# Asymmetric Xanthene with Noncovalent Conformation Locks to Attain High Fluorescence >1500 nm

**DOI:** 10.1021/jacs.5c23313

**Published:** 2026-04-21

**Authors:** Partha Chowdhury, Hsiu-Feng Lu, Hsin-Ting Huang, Meng-Huan Liu, Yen-Chang Chen, Kai-Min Chan, Syue-Liang Lin, Ricardas Rotomskis, Simona Steponkiene, Tung-Kung Wu, Yu-Fen Huang, Chao-Ping Hsu, Yang-Hsiang Chan

**Affiliations:** † Department of Applied Chemistry, 34914 National Yang Ming Chiao Tung University, Hsinchu 300, Taiwan, R.O.C.; ‡ Institute of Chemistry, Academia Sinica, Taipei 115, Taiwan, R.O.C.; § Department of Biomedical Engineering and Environmental Sciences, 34881 National Tsing Hua University, Hsinchu 300, Taiwan, R.O.C.; ∥ Biomedical Department of Biotechnology and Laboratory Science in Medicine, National Yang Ming Chiao Tung University, Taipei 112, Taiwan, R.O.C.; ⊥ Biomedical Physics Laboratory of National Cancer Institute, Baublio 3B, LT-08406 Vilnius, Lithuania; # Department of Biological Science, College of Engineering Bioscience, Center for Emergent Functional Matter Science, National Yang Ming Chiao Tung University, Hsinchu 300, Taiwan, R.O.C.; ∇ Institute of Analytical and Environmental Sciences, National Tsing Hua University, Hsinchu 300, Taiwan, R.O.C.; ○ School of Pharmacy, College of Pharmacy, Kaohsiung Medical University, Kaohsiung 807, Taiwan, R.O.C.; ◆ Center for Emergent Functional Matter Science, National Yang Ming Chiao Tung University, Hsinchu 300, Taiwan, R.O.C.; ¶ Department of Medicinal and Applied Chemistry, Kaohsiung Medical University, Kaohsiung 807, Taiwan, R.O.C.

## Abstract

Fluorescence imaging in the NIR-IIb window (1500–1700 nm) enables unparalleled tissue penetration and contrast, yet efficient organic fluorophores >1500 nm remain extremely scarce due to intrinsic trade-offs between emission wavelength and brightness in symmetric molecular designs. To date, molecular scaffolds predominantly adopt either symmetric planar or twisted frameworks, which can individually optimize the long emission wavelength (λ_em_) or high quantum yield (ϕ) but fail to overcome this fundamental optical trade-off simultaneously. In this study, we propose a rational asymmetric design that integrates planar ACQ units with twisted AIE rotors within a single molecular scaffold, uniting the complementary advantages of long λ_em_, a high molar extinction coefficient (ε), and elevated ϕ. We further introduce the concept of intermolecular noncovalent conformational locks (NoCLs) that can precisely tune the photophysical properties within nanoparticles. To validate this concept, we design and synthesize a systematic library of asymmetric dyes (**T-X-J**, **T-X-BJ**, **J-X-BJ**, and **T-Si-BJ**) by combining heteroatom-substituted xanthene cores (O, Si) with structurally diverse electron-donating groups (**T**, **J**, **BJ**), alongside symmetric analogues (**T-X-T**, **J-X-J**, **BJ-X-BJ**, **T-Si-T**, and **BJ-Si-BJ**) as controls. Embedding these dyes into a heteroatom-rich polymer matrix (**Pttc-TTQ**) establishes intermolecular noncovalent conformational locks (C–H···π and C–H···O) for enabling precise regulation of dihedral angles and significantly enhancing ϕ in nanoparticles. Notably, **T-Si-BJ** exhibits emission >1500 nm in CH_2_Cl_2_, representing the first asymmetric organic dye with emission maxima in the NIR-IIb window, and demonstrates the brightest (14.1 M^–1^ cm^–1^) nanoparticles with emission maxima at 1320 nm and tail signal extending into the NIR-IIb region among the reported optical trade-off fluorophores in H_2_O (Scheme 1). **T-Si-BJ** was further prepared as polymer dots (Pdots) for high-contrast, deep-tissue vascular imaging and AI-assisted resolution of hindlimb vasculature. This study provides a synthetically versatile and structurally diverse platform for NIR-IIb fluorophores, demonstrating that asymmetric donor–acceptor–donor scaffolds coupled with polymer-mediated geometry control can simultaneously optimize the emission wavelength and brightness.

## Graphical abstract

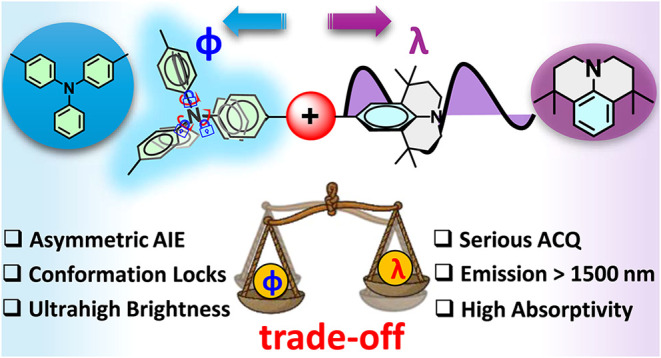

## Introduction

Fluorescence bioimaging in the shortwave infrared (SWIR, 1000–3000 nm) region offers remarkable promise for high-contrast deep-tissue visualization.^1–5^ However, photon scattering within biological tissues continues to restrict imaging depth and resolution, posing a fundamental barrier to high-fidelity *in vivo* studies.^6,7^ Extending both excitation and emission into the second near-infrared region (NIR-II, 1000–1700 nm) provides an effective route to overcome this limitation, as reduced scattering and autofluorescence enable deeper tissue penetration and sharper image contrast.^8^ In particular, NIR-II subwindows NIR-IIx (1400–1500 nm)^9^ and NIR-IIb (1500–1700 nm)^10^ are considered the best imaging windows for achieving superior imaging contrast and clarity.^11,12^ Consequently, fluorophores emitting beyond 1500 nm are key to advancing deep-tissue bioimaging.

Promoting emission wavelength >1500 nm, currently reported fluorophores primarily rely on metal-based nanoparticles.^13–16^ Despite their optical advantages, concerns regarding long-term toxicity hinder their use for *in vivo* imaging.^17^ Organic fluorophores, in contrast, offer intrinsic advantages including biodegradability, biocompatibility, and synthetic tunability, which make them highly attractive for NIR-IIb applications.^18^ Despite significant progress with emitters >1200 nm,^1,19^ efficient organic fluorescence beyond 1500 nm remains scarce, with SiRos1500 being a notable exception.^20^

To date, organic fluorophores emitting beyond 1200 nm have predominantly adopted symmetric molecular scaffolds terminated by either two planar or two twisted chromophores. Such a design has recently been proved to possess aggregation-caused quenching (ACQ), which occurs due to strong intermolecular π–π interactions, particularly in planar π-conjugated systems that lead to nonradiative decay pathways in the aqueous medium. Moreover, these fluorophores also exhibit undesired H-aggregation in aqueous solutions which results in emission quenching by several-fold.^20–23^ Conversely, twisted chromophores in a symmetric scaffold were designed to mitigate π–π interactions by restricting intramolecular motion, thereby achieving high quantum yield (ϕ) in aqueous medium which are also known as aggregation-induced emission (AIE).^24^ From a photophysical standpoint, planar chromophores possess large molar extinction coefficients (ε) and yield red-shifted emission wavelengths (λ_em_) through excitonic coupling,^25^ while twisted chromophores display smaller ε and blue-shifted emission due to increased dihedral angles upon aggregation.^26^ Hence, symmetric AIE or ACQ scaffolds excel individually in brightness or emission wavelength but fail to optimize both simultaneously. This intrinsic limitation in symmetric scaffolds significantly restricts the molecular diversity and hinders the development of next-generation organic NIR-II fluorophores.

Though scanty, only a handful of design strategies have been reported sporadically to address this setback. The very first approach, pioneered by Tang et al., ingeniously combined AIE and ACQ to develop an asymmetric scaffold that achieved longer emission and higher brightness as compared to their symmetric AIE or ACQgens (**pNIR-4**, Scheme 1).^27^ Subsequently, this design concept was recently implemented in hemicyanine core (**DPM-HD1**-**HD3**, Scheme 1), wherein a planar xanthene unit (ACQ) was coupled with a structurally twisted xanthene analogue (AIE) to achieve long emission and superior brightness compared with their AIE or ACQ dyes.^28^ This strategy seems to hold great promise to solve the intrinsic limits of symmetric AIE or ACQ scaffolds. Despite these advances, current asymmetric designs fall short of achieving target absorption and emission regimes (Scheme 1, upper panel), probably due to their intrinsic donor–acceptor strength within donor–acceptor–donor (D–A–D) or D–π–A backbones. Therefore, extending this synergistic design into the NIR-IIb window remains a longstanding challenge. Yet, it holds the key to unlock the longer emissive organic emitters capable of deep-tissue imaging beyond 1500 nm.

**Scheme 1:**
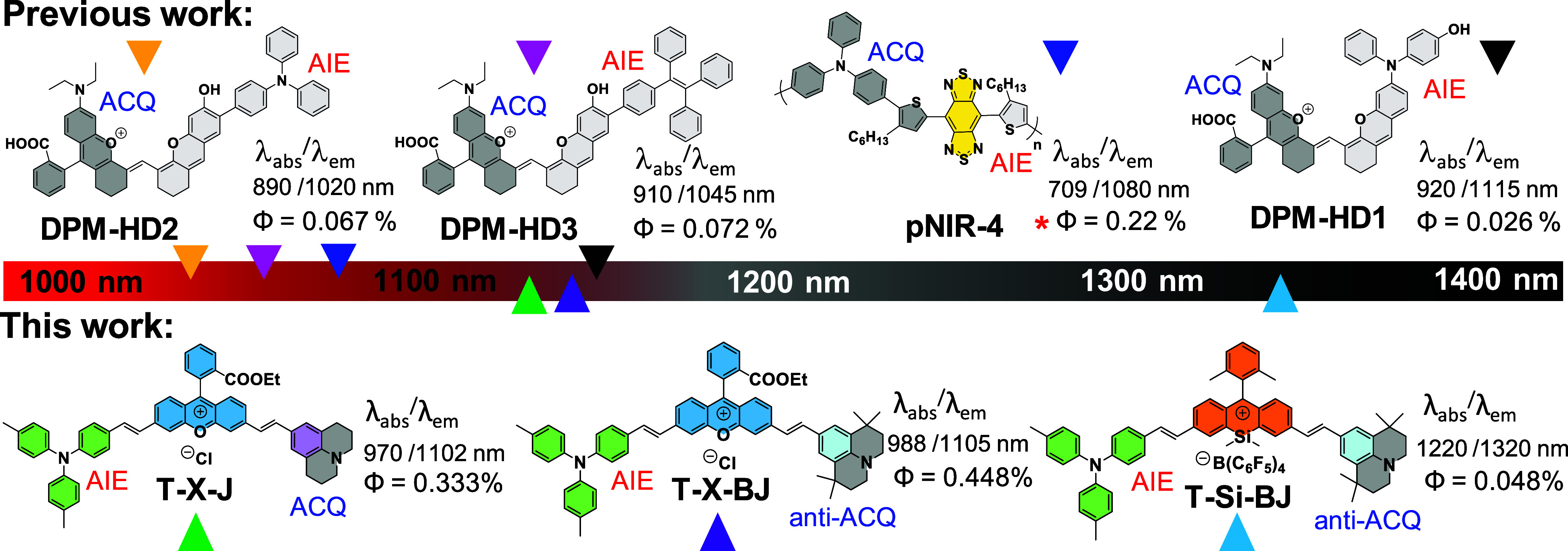
Emission Wavelengths and Quantum Yields of the Existing Asymmetric AIEgens with Emission <1200 nm and the Asymmetric AIEgens in This Work with Emission >1300 nm, Measured in Solid State or H_2_O (e.g., Pdots Assembled with Polymer Matrix)^a^

a Their absorption and emission peak wavelengths and quantum yield in the reported solvents can refer to pNIR-4,^27^ DPM-HD1,^28^ DPM-HD2,^28^ and DPM-HD3.^28^ Note: The quantum yield (ϕ) of pNIR-4 was originally measured using IR-26 as a reference with a ϕ of 0.5%. However, the widely accepted ϕ of IR-26 in DCE is 0.05%. Accordingly, the ϕ of pNIR-4 has been recalculated to be ten times lower than the previously reported value (marked as * in Scheme 1).

To address this challenge, we propose a rational molecular design that couples the high ε and long λ_em_ of ACQ with the high ϕ of the AIE system for NIR-IIb emission. We hypothesize that structural flexibility and rigidity of a donor embedded in a molecular backbone can produce twisted (AIE-type) and planar (ACQ-type) conformations (Scheme 2A), thereby constructing a strong electron-accepting core terminated with flexible and rigid donors yields an asymmetric framework, exhibiting both AIE and ACQ simultaneously (Scheme 2B). Such a dual-function framework effectively unites the complementary advantages of ACQ (longer λ_em_ and high ε) and AIE (high ϕ) for future development of NIR-IIb dyes.^29^

**Scheme 2:**
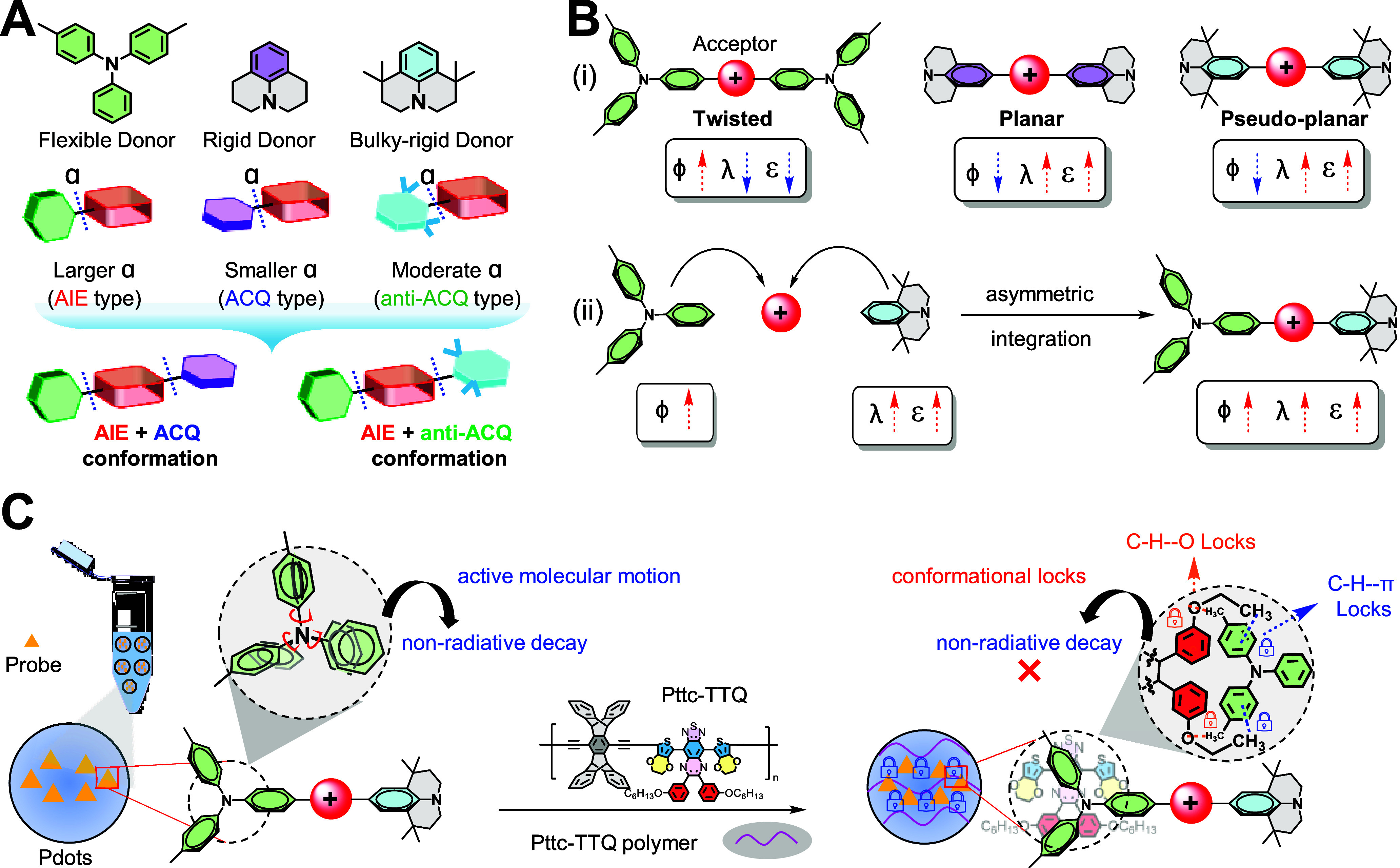
Representation of the Asymmetric Design Strategy^a^

a (A) Dye geometry in aggregation when flexible triphenylamine (**T**) unit (molecular rotor), rigid julolidine (**J**) unit, and bulky-rigid tetramethyl julolidine (**BJ**) unit are attached to the molecular backbone. (B) Comparison of (i) symmetric and (ii) asymmetric design strategies, where λ, ε, and ϕ represent absorption/emission wavelength, molar extinction coefficient, and quantum yield, respectively. (C) Illustration of intermolecular noncovalent conformation locks between twisted triphenylamine unit and **TTQ** unit of the **Pttc-TTQ** polymer to improve emission yield in the nanoparticle form.

We herein design a xanthene-based emissive core (electron acceptor) terminated by strong electron-donating groups to achieve NIR-IIb emissive windows. To further red-shift both the absorption and emission wavelengths, we substituted the central heteroatom of this xanthene core, thereby achieving highly emissive NIR-IIb fluorophores. To induce the desired molecular geometry, we next sought to finely regulate the dihedral angles between the donor and the acceptor in our molecular framework. Recent works have proved that engineering alkyl chains on symmetric AIE-based NIR-II molecules by tuning alkyl length, positions, and topology has offered an intuitive strategy to regulate molecular conformations and the packing behavior of aggregated materials.^10,27,30^ This design has successfully achieved high NIR-IIb emission in the nanoparticle state.^10^ However, these studies mostly rely on benzobisthiadiazole-based acceptors, while the dihedral angles within the D–A–D framework can vary substantially depending on the specific acceptor structure. As a result, the mechanisms underlying AIE or ACQ behavior in these systems tend to be case-dependent and somewhat of consequentialism, which are beyond the scope of the present work.^27,31^ We thus propose a more universal and facile strategy to regulate the extent of dihedral angle for modulating the molecular geometry and the emission wavelength by introducing noncovalent conformation locks (NoCLs) between asymmetric NIR-II dyes and polymer matrix in the nanoparticle form (Scheme 2C).^32–38^ Specifically, we implanted intermolecular NoCLs between dyes and polymers by using C–H···π and C–H···O noncovalent interactions that can better manipulate the extent of dihedral angles in the D–A–D scaffold and thereon tune their optical properties.

To prove our proposed concept, we strategically design and synthesize a series of asymmetric heteroatom-substituted xanthenes, comprising oxygen- and silicon-centered cores (hereafter denoted as **X** and **Si**, respectively) terminated with structurally flexible and rigid electron-donating end groups (Scheme 2A), 4-methyl-*N*-phenyl-N-(p-tolyl)­aniline (**T**) as AIE rotor, julolidine (**J**) as rigid ACQ unit, and tetramethyl julolidine (**BJ**) as sterically hindered rigid anti-ACQ unit,^39,40^ yielding **T-X-J**, **T-X-BJ**, **J-X-BJ**, and **T-Si-BJ** (see Scheme 3, asymmetric). For comprehension, symmetric xanthenes **T-X-T**, **J-X-J**, **BJ-X-BJ**, **T-Si-T**, and **BJ-Si-BJ** were also prepared (see Scheme 3, symmetric). As a result, the freely rotating **T** when embedded in a backbone induces a large dihedral angle, whereas the sterically rigid fused-ring groups **J** and **BJ** enforce small to medium dihedral angles, respectively. Thus, integrating a large dihedral angle of **T** with **J** or **BJ** yields twisted with planar and pseudoplanar geometry, which in turn form AIE (**T**) + ACQ (**J**) or AIE (**T**) + anti-ACQ (**BJ**) frameworks within a single scaffold, respectively. To further regulate the dihedral angle of asymmetric dyes in the nanoparticle form, we then design a polymer matrix, **Pttc-TTQ** (Scheme 2C), capable of forming intermolecular NoCLs with the dye molecules. Conceptually, the **Pttc** segment functions as an inert, bulky unit that suppresses dye aggregation, whereas the heteroatom-rich **TTQ** segment serves as the active NoCL-forming unit. Taking advantage of the intermolecular NoCLs from **TTQ**, we assume to harness the active molecular motion of the **T** unit for inhibiting the radiationless decay of the asymmetric dyes in the nanoparticle form (Scheme 2C).^41,42^

**Scheme 3:**
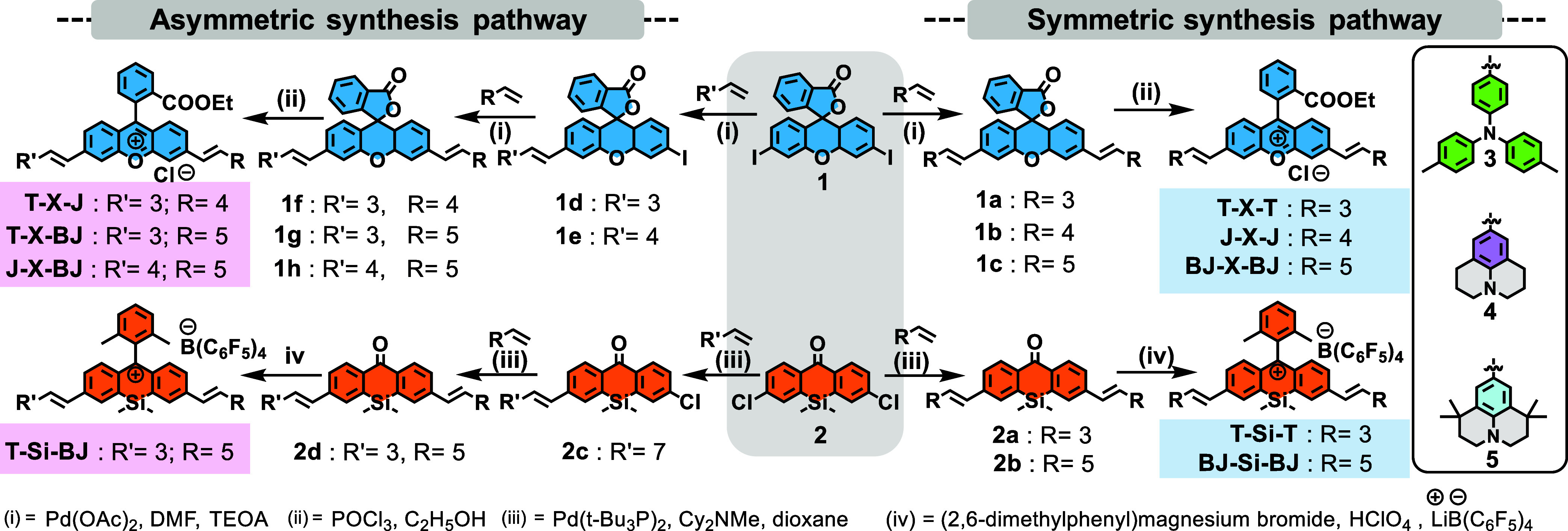
Reaction Scheme for the Synthetic Routes for Symmetric and Asymmetric Xanthenes Synthesized in This Study^a^

a See the text and Supporting Information for the details of synthesis and characterization.

Subsequently, we employed a combination of steady-state spectroscopy and computational analysis to validate the proposed concept. These studies revealed that asymmetric xanthenes in a nanoparticle, when embedded within the polymer matrix, exhibit markedly enhanced optical performance enabled by the formation of C–H···π and C–H···O intermolecular conformational locks. These NoCLs effectively regulate the molecular geometry, emission wavelength, and overall brightness within the aggregated state. Notably, **T-Si-BJ** demonstrated the highest brightness among all xanthenes investigated and represents the longest-wavelength emissive asymmetric AIEgen reported to date (Scheme 1). The proof-of-concept **T-Si-BJ** nanoparticle was then used for acquiring high-contrast deep-tissue vascular imaging at >1500 nm, along with AI-assisted simulations to achieve exceptionally high-resolution hindlimb visualization.

## Results and Discussion

### Synthesis and Characterization

Scheme 3 outlines the synthetic strategies employed to prepare symmetric and asymmetric heteroatom-substituted xanthenes. Two distinct synthetic approaches were followed, one for symmetric and another for asymmetric xanthenes derivatives. For the synthesis of symmetric xanthenes, compounds **1** and **2** were first prepared as key precursors. Compound **1** was synthesized via the condensation of 3-iodophenol with phthalic anhydride in methanesulfonic acid (see Scheme S1 in the Supporting Information), and Compound **2** was synthesized as previously reported^43^ starting from 2-bromo-4-chloro-1-iodobenzene and 2-bromo-4-chlorobenzaldehyde, followed by oxidation with KMnO_4_ to obtain the corresponding ketone suitable for further functionalization. The donor alkene moieties of compounds **3**, **4**, and **5** were synthesized using the Wittig reaction, as reported in the previous literature.^21^ C–C coupling reactions were then performed between the halogenated precursors (compounds **1** and **2**) and alkenes of **3, 4**, and **5** using palladium catalysis. These reactions yielded symmetric oxygen-containing xanthenes (**1a–1c**) and symmetric silicon-containing xanthenes (**2a**, **2b**). Asymmetric xanthenes were synthesized by Heck reaction using a palladium catalyst in two steps. By controlling the molar ratio, we first C–C coupled the alkene of compounds **3** and **4** with compound **1** and the alkene of compound **3** with compound **2** to prepare the precursor compounds **1d**, **1e**, and **2c**, respectively, that extend one side of the xanthene backbone. Followed by the second C–C coupling, compound **1d** reacted with the alkene of compounds **4** and **5** to yield asymmetric oxygen-xanthenes compounds **1f** and **1g**, while compound **1h** was prepared by the alkene of compound **5** with compound **1e**. Similarly, asymmetric silicon-xanthene compound 2**d** was prepared from compound **2c** and the alkene of compound **5** by using a palladium catalyst. Next, lactone-containing symmetric and asymmetric oxygen-xanthenes (**1a**–**1c**, **1f**–**1h**) were converted into ring-opened derivatives through esterification with ethanol in the presence of POCl_3_, yielding the final products: **T-X-T**, **J-X-J**, and **BJ-X-BJ** as symmetric xanthenes, and **T-X-J**, **J-X-BJ**, and **T-X-BJ** as asymmetric xanthenes, respectively. In the case of silicon-based xanthenes, the final dyes (**T-Si-T**, **BJ-Si-BJ**, and **T-Si-BJ**) were synthesized in a one-pot process. Compounds **2a**, **2b**, and **2d** were subjected to a Grignard reaction using 2,6-dimethylphenylmagnesium bromide, followed by acidic treatment with 2 M HClO_4_. To stabilize the cation and prevent it from nucleophilic decomposition in a polar environment, a counterion-pairing strategy was employed using lithium tetrakis­(pentafluorophenyl)­borate ethyl ether. The chemical structures of synthesized dyes are summarized in Figure 1A, and their photophysical properties in CH_2_Cl_2_ are shown in Figure 1B.

**Figure 1:**
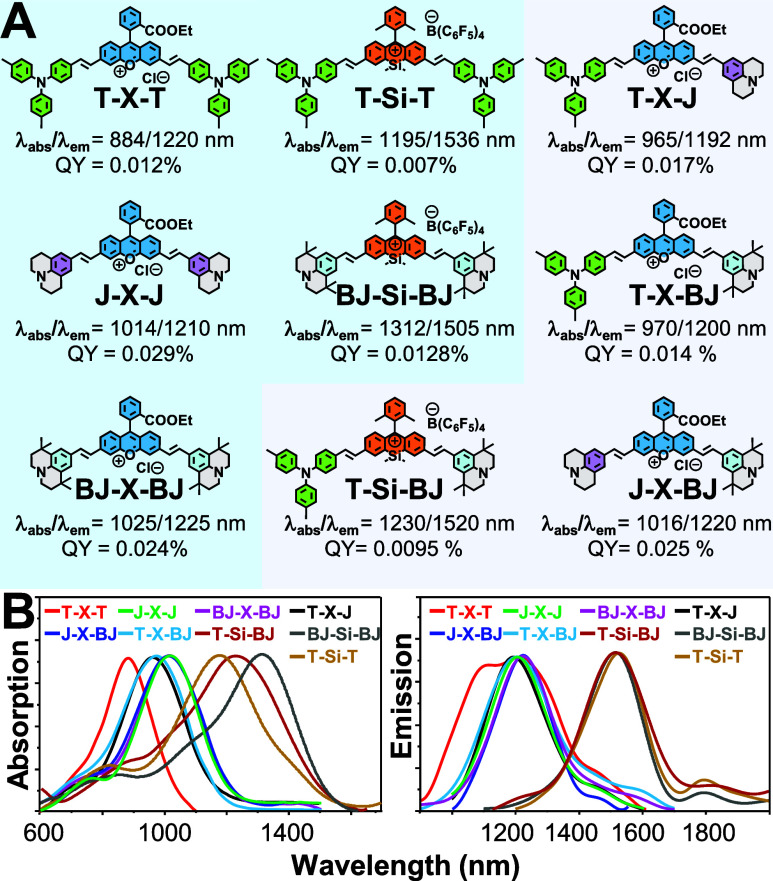
(A) Chemical structures of symmetric and asymmetric NIR-II xanthenes synthesized in this work. (B) Their corresponding absorption/emission wavelengths and relative ϕ measured in CH_2_Cl_2_ without polymer (Reference, IR-1061; ϕ = 0.59% in CH_2_Cl_2_).^44^.

### Steady-State Spectroscopy and Computational Approach

Absorption spectroscopy reveals that **T**-terminated xanthenes exhibit shorter absorption maxima (Figure 1B) compared to those of **J** and **BJ** analogues. This trend might arise from the weaker electron-donating ability of the **T** unit in the ground state, consistent with our computed HOMO energy levels (Figure 2A). The reduced electron-donating strength of **T** originates from the limited availability of the nitrogen lone pair in the D–A–D scaffold, as it is shared with the two p-tolyl units (Figure 2B). In the D_1_–A–D_2_ system, A is the electron acceptor, and D_1_ and D_2_ are both electron donors. Therefore, upon excitation, there is a charge transfer from both D_1_ and D_2_ to A. In Figure 2B, HOMO shows that the electron density is primarily delocalized on the D_1_–A–D_2_ framework, while LUMO indicates that electrons concentrate toward the center (A) of the dye, with minimal electrons distributed at the donor sites of the D_1_ and D_2_ parts. Thus, in the excited state, however, intramolecular charge transfer (ICT) leads all xanthenes to emit within a similar spectral window. Upon substituting the central heteroatom “O” with “Si”, both absorption and emission were found to be red-shifted irrespective of terminal units. Accordingly, **X**-derivatives exhibit absorption in the range 900–1000 nm and emission primarily located at 1200 nm. In contrast, **Si**-derivatives show absorption at 1200–1300 nm and emit at wavelengths >1500 nm with a tail elongated over 1800 nm (Figure 1B and Table 1). To further elucidate these observations, computational analyses were conducted on both O-Xanthenes and Si-Xanthenes. Geometry optimizations and frequency calculations were performed using the B3LYP-D3/6-31G­(d) level of theory to ensure the absence of imaginary frequencies. Using symmetric O-Xanthenes as representative models, vertical excitation energies were calculated via time-dependent density functional theory (TDDFT) employing nine different functionals with the 6-31G­(d) basis set (Table S1). However, the results obtained from the SOS-CIS­(D) method showed the best agreement with experimental data, as shown in Table 1. Consequently, theoretical optical properties were derived based on the SOS-CIS­(D) results. The lower excitation energies observed for Si-Xanthene dyes are consistent with the experimental results. Inspired by a previous study,^45^ where the low-lying LUMO was attributed to the conjugation between the σ* orbital of the SiMe_2_ group and the π* orbital of the diene fragment in a silole ring, we examined the HOMO and LUMO energies, as well as the corresponding HOMO–LUMO gaps (Figure 2A). The results clearly show that the LUMO energies of Si-Xanthenes are lower than those of O-Xanthenes. Consequently, the smaller HOMO–LUMO energy gap contributes to the lower excitation energies, leading to red-shifted absorption wavelengths. Furthermore, the LUMO isocontour surfaces depicted in Figure 2B reveal that the LUMO of Si-Xanthene extends over the methyl groups of the SiMe_2_ unit, confirming the presence of σ*−π* conjugation and the associated stabilization effect.

**Figure 2:**
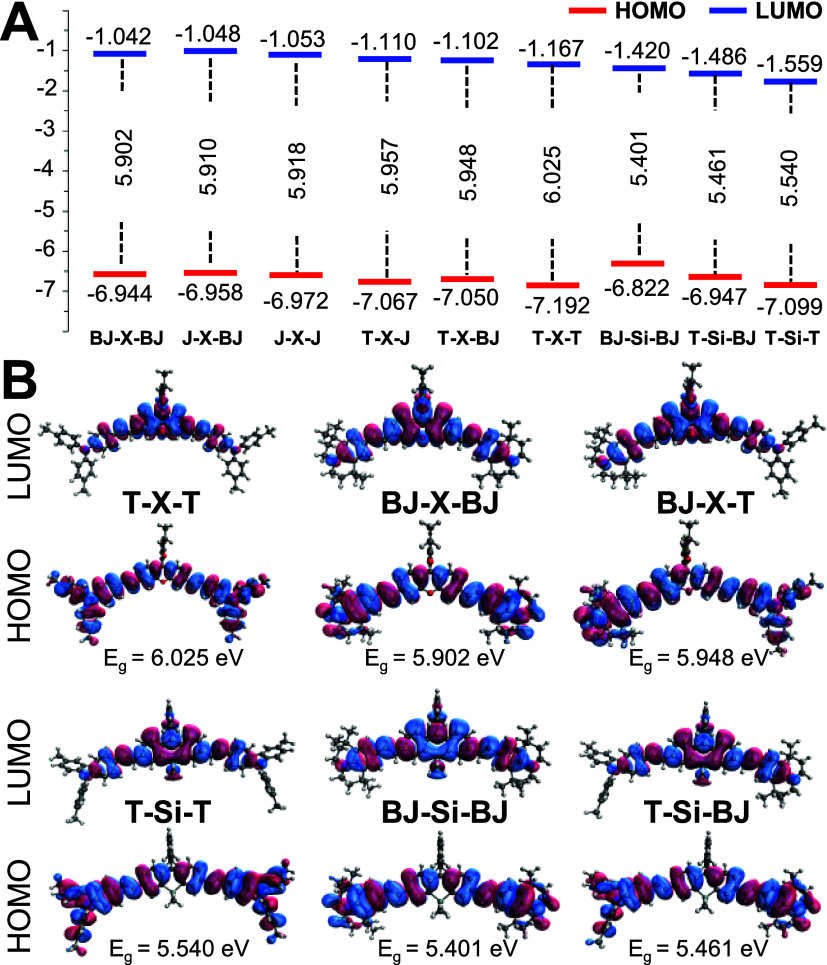
(A) Molecular orbital energy diagram of oxygen and silicon substituted xanthenes **T-X-T**, **J-X-J**, **BJ-Si-BJ**, **T-X-BJ**, **T-X-J**, **T-Si-T**, **BJ-Si-BJ**, and **T-Si-BJ**, calculated at the HF (CPCM, CH_2_Cl_2_)/DZV level. (B) Isocontour surfaces for HOMO and LUMO of **T-X-T**, **T-X-BJ**, **BJ-X-BJ**, **T-Si-T**, **T-Si-BJ**, and **BJ-Si-BJ** at the HF (CPCM, CH_2_Cl_2_)/DZV level.

**Table 1:** Photophysical Properties of Experimental Results and the Calculated Vertical Excitation Data (*E*_abs_ in eV and λ_abs_ in nm) at the SOS-CIS­(D) (CH_2_Cl_2_, CPCM) /VDZ of Symmetric and Asymmetric Xanthenes in CH_2_Cl_2_ without Polymer

**experimental data**					**SOS-CIS(D)(CH**_**2**_ **Cl**_**2**_, **CPCM) /VDZ**		
**dye**	λ_max_^abs^ (nm)	λ_max_^em^ (nm)	ε_max_ (M^–1^ cm^–1^)	Φ (%)	brightness (M^–1^ cm^–1^)	*E*_abs_	λ_abs_ (nm)
**T-X-T**	884	1220	0.605 × 10^5^	0.012	7.26	1.741	712
**T-X-J**	965	1192	1.720 × 10^5^	0.017	29.24	1.551	800
**T-X-BJ**	970	1200	1.680 × 10^5^	0.014	23.52	1.522	815
**J-X-J**	1014	1210	2.190 × 10^5^	0.029	63.51	1.452	854
**J-X-BJ**	1016	1220	1.990 × 10^5^	0.025	49.75	1.434	865
**BJ-X-BJ**	1025	1225	2.070 × 10^5^	0.024	49.68	1.418	875
**T-Si-T**	1195	1536	0.420 × 10^5^	0.007	1.680	1.136	1091
**T-Si-BJ**	1230	1520	0.910 × 10^5^	0.009	4.823	0.963	1288
**BJ-Si-BJ**	1312	1505	1.390 × 10^5^	0.012	10.00	0.856	1449

To further justify our molecular designs, we then measured their ε and ϕ values as listed in Table 1. The results reveal that **T**-containing xanthenes exhibit lower ε and ϕ than **J** or **BJ** in CH_2_Cl_2_, in which **T-X-T**, **T-X-J**, **T-X-BJ**, **T-Si-T**, and **T-Si-BJ** exhibit very low ϕ of 0.012, 0.017, 0.014, 0.007, 0.009%, and lower ε of 60,500, 172,000, 168,000, 42,000, and 91,000 M^–1^ cm^–1^, respectively. In contrast, **J-** and **BJ**-containing xanthenes, **J-X-J**, **BJ-X-BJ**, **J-X-BJ**, and **BJ-Si-BJ**, show relatively higher ϕ of 0.029, 0.024, 0.025, and 0.012%, and high ε of 219,000, 207,000, 199,000, and 139,000 M^–1^ cm^–1^, respectively, in CH_2_Cl_2_. Notably, asymmetric xanthenes **T-X-J**, **T-X-BJ**, and **T-Si-BJ** exhibit ε and ϕ values between those of their symmetric analogues **T-X-T**, **J-X-J**, **BJ-X-BJ**, **T-Si-T**, and **BJ-Si-BJ**, in CH_2_Cl_2_, which validates our design strategy in terms of structurally twisted (AIE-type), planar (ACQ-type), and their combination twisted+planar (AIE+ACQ-type) frameworks within a single scaffold. Geometry optimizations by DFT calculations were also performed, showing that for xanthenes containing **T**, the *p*-tolyl rings exhibit rotational degrees of freedom of approximately ±23.5 and ±24.3° relative to the nitrogen center (Figure S1). This conformational flexibility facilitates nonradiative energy dissipation and thus leads to lower ϕ. In contrast, due to the absence of rotatable phenyl units in **J** and **BJ**, such nonradiative loss is negligible, resulting in relatively higher ϕ and ε.

Overall, these results demonstrate that both the terminal donor units and the central heteroatom play decisive roles in governing the optical behavior of the xanthene dyes. We then studied their aggregation mechanisms underlying the interactions among molecules.

### Aggregation Behavior, AIE, ACQ, and Anti-ACQ Properties

Aggregation-induced photophysical behaviors of the symmetric and asymmetric xanthenes were studied from both theoretical and experimental points of view. At the molecular level, we performed structural optimizations for all oxygen-xanthene derivatives with a zero-point energy correction and calculated the BSSE-corrected binding energy of xanthene dimers at the M06-2*X*/6-31+G*//B3LYP-D3/6-31G* level. Additionally, we calculated the dihedral angles located at C = C relative to donors (denoted as α1 and α2) and C = C to the xanthene core (denoted as β1 and β2), as summarized in Figure 3, with all the dihedral angles listed in Table 2. The binding energy of the dimers explains the stability of the two monomer units when it comes to their proximity, revealing that the flexible **T**-terminated xanthenes form the most stable dimers, followed by **BJ**- and **J**-terminated systems (Figure 3).

**Figure 3:**
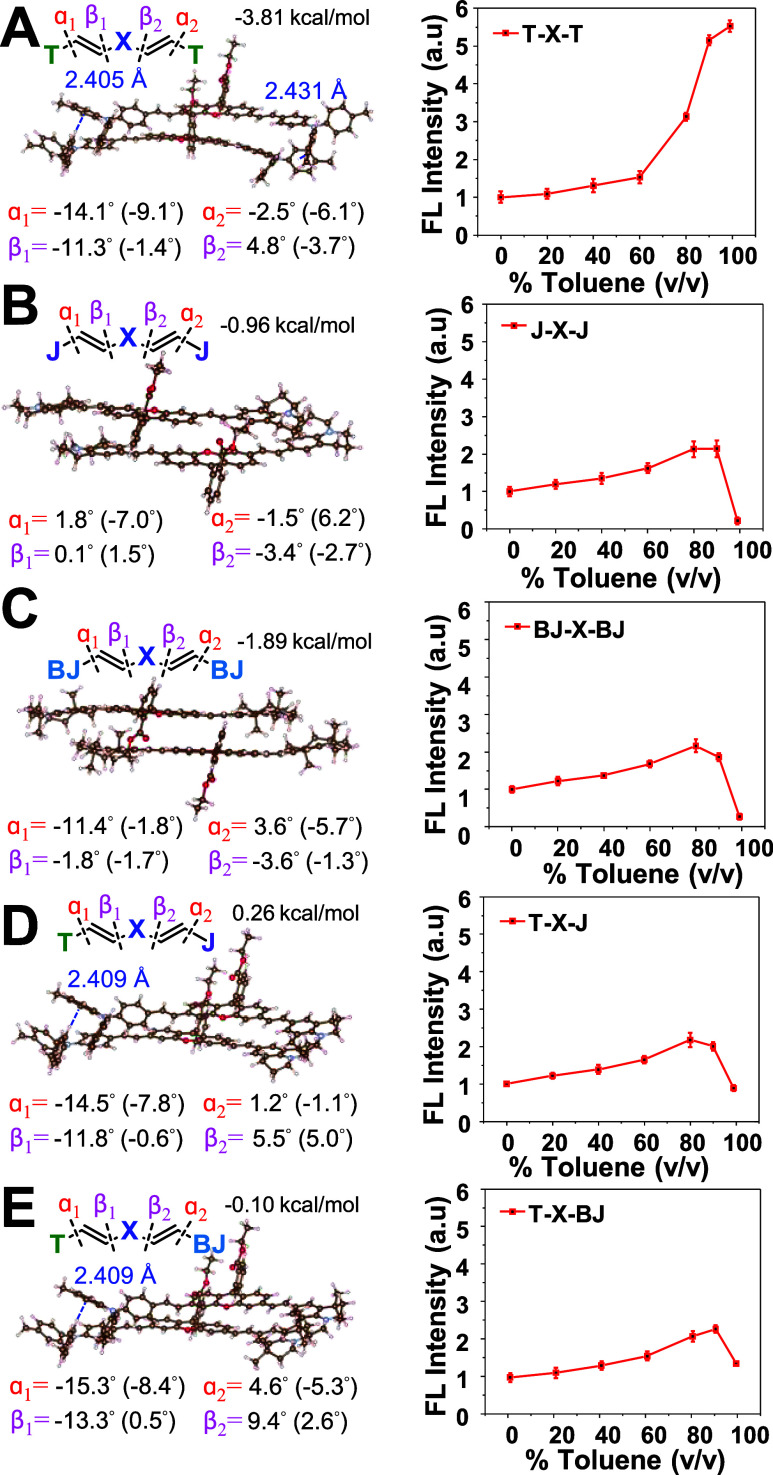
Theoretical and experimental calculations to evaluate the aggregation behaviors of (A) **T-X-T**, (B) **J-X-J**, (C**) BJ-X-BJ**, (D**) T-X-J**, and (E) **T-X-BJ** dyes. The left column depicts the dihedral angles of the optimized geometry at the ground state (S_0_) of symmetric and asymmetric O-xanthenes and shows the optimized structures, Zero-point energy corrections, and BSSE-corrected binding energy of low-energy Xanthene dimers at the M06-2*X*/6-31+G*//B3LYP-D3/6-31G* level. Brown, light blue, red, and white represent carbon, nitrogen, oxygen, and hydrogen atoms, respectively. The blue dashed line represents the C–H···π force. The right column describes fluorescence variation with % toluene (v/v) in DMSO/toluene mixture (standard deviation *n* = 3).

**Table 2:** Dihedral Angle of Linker (C = C) Relative to Chromophore (α1 and α2) or Xanthene Core (β1 and β2) of All Xanthene Monomers and Low-Energy Xanthene Dimers^a^

dye	packing model	α1	β1	β2	α2
T-X-T	monomer	1.5	0.2	–0.2	–1.5
	dimer	–14.1(−9.1)	–11.3(−1.4)	4.8(−3.7)	–2.5(−6.1)
J-X-J	monomer	0.2	0.1	–0.3	–0.1
	dimer	1.8(−7.0)	0.1(1.5)	–3.4(−2.7)	–1.5(6.2)
BJ-X-BJ	monomer	–0.1	–0.4	–0.1	–0.6
	dimer	–11.4(−1.8)	–1.8(−1.7)	–3.6(−1.3)	3.6(−5.7)
J-X-BJ	monomer	0.2	0.1	–0.2	–0.8
	dimer	1.5(−6.5)	–0.7(1.8)	–1.0(−2.9)	–0.3(5.9)
T-X-J	monomer	–1.4	0.0	0.6	0.5
	dimer	–14.5(−7.8)	–11.8(0.6)	5.5(5.0)	1.2(−1.1)
T-X-BJ	monomer	–1.5	–0.2	–0.1	–0.8
	dimer	–15.3(−8.4)	–13.1(0.5)	9.4(2.6)	4.6(−5.3)

a For dimers, the values in parentheses represent the dihedral angles of the lower-layer molecules, while the values outside brackets are those in the upper-layer molecules, as shown in Figure 3.

Following calculations found that **T**-terminated xanthenes displayed large α1 and β1 dihedral angles, which indicate that **T** groups introduce substantial twisting within the D–A–D scaffold and promote a highly twisted aggregated geometry. On the other hand, **J**-terminated xanthenes exhibited very small α1 and β1 angles, giving rise to a nearly planar framework. Interestingly, **BJ**-terminated xanthenes showed selectively twisted α1 angles due to steric hindrance from the 4-methyl substituents while β1 remained relatively small, suggesting that **BJ** induces a pseudoplanar geometry with moderate distortion. As a result, **T-X-T** exhibited the most twisted geometry with the large dihedral angles α1 and β1 of −14.1° (−9.1°) and 11.3° (−1.4°) at one end and α2 and β2 of −2.5° (−6.1°) and 4.8° (−3.7°) in dimers (Figure 3A). Note that the values in parentheses represent the dihedral angles of the lower-layer molecules, and the values outside brackets are those in the upper-layer molecules. This twisted geometry of the **T-X-T** originates from the two C–H···π interaction with a hydrogen of tolyl groups attached to another phenyl as acceptor, with the bond distances from H to the center of the benzene ring being 2.405 Å and 2.431 Å, resulting in a larger dihedral angle. In contrast, **J-X-J** resulted in a small dihedral angle α1 and β1 of 1.8° (−7.0°) and 0.1° (1.5°) at one end and α2 and β2 of −1.5° (6.2°) and −3.4° (−2.7°) at another, in dimers (Figure 3B). In the case of **BJ-X-BJ**, 4-methyl units twist α1 and α2 at −11.4° (−1.8°) and 3.6° (−5.7°), whereas β1 and β2 showed small dihedral angles at −1.8° (−1.7°) and −3.6° (−1.3°), respectively, in dimer (Figure 3C).

Interestingly, by introducing twisted **T** in one end and planar **J** on the other end of the xanthene core, **T-X-J** exhibited the combination of large and small dihedral angles in a D–A–D scaffold at α1 and β1 of −14.5° (−7.8°) and −11.8° (−0.6°), whereas α2 and β2 of 1.2° (−1.1°) and 5.5° (5.0°), respectively, in dimer (Figure 3D). Thereby, **T-X-J** adopted asymmetric twisted+planar geometry in a single scaffold. Similarly, the xanthene core terminated by **T** at one end and **BJ** on the other displayed an integrated large and moderate dihedral angle in a D–A–D scaffold at α1 and β1 of −15.3° (−8.4°) and −13.3° (0.5°), whereas α2 and β2 were 4.6° (−5.3°) and 9.4° (2.6°), respectively, in dimer. Due to the larger α2 and β2 of **BJ** compared to **J**, **T-X-BJ** adopted twisted+pseudoplanar geometry at aggregation (Figure 3E), whereas **J-X-BJ** showed a planar geometry designed as a control asymmetric dye to evaluate the right combination strategy in this study (Figure S2). Building on this, we constructed an experimental aggregate model to further confirm our design strategy, validated by the above computational analysis.

Given that the compounds are soluble in DMSO but insoluble in toluene, gradual addition of toluene effectively induces molecular aggregation and thus allows us to probe the relationship among molecular geometry, aggregation state, and emission response. Accordingly, we measured the emission properties of the studied compounds at varying toluene fractions in DMSO and at 99% toluene fraction (right columns in Figure 3A–E) to mimic a highly aggregated state. Computational dihedral angle calculations suggest that **T**-containing derivatives adopt a twisted geometry, which could reduce intermolecular π–π stacking and thus lead to an increase in the emission intensity upon aggregation. In contrast, **J** derivatives adopt a highly planar conformation, making them more susceptible to π–π stacking and consequently expected to exhibit strong emission quenching upon aggregation. Meanwhile, the **BJ** derivative, which induces a pseudoplanar geometry, is anticipated to exhibit moderate quenching behavior because its structure partially resists π–π stacking. Thus, **T-X-T** exhibited very high emission enhancement with a 5.6-fold increase in emission intensity at 99% toluene fraction in DMSO. This enhancement is attributed to the presence of two C–H···π interactions between neighboring **T** units as shown in Figure 3A. On the other hand, **J-X-J** and **BJ-X-BJ** display severe quenching upon aggregation with a reduction of initial intensity to 4.5-fold and 3.6-fold, respectively, at 99% toluene fraction in DMSO. Consistent with the dihedral angle analysis, **BJ-X-BJ** showed resistance to ACQ due to 8-methyl units of **BJ** as compared to **J**, confirming the anti-ACQ character of the **BJ** moiety (Figure 3B,C).

Next, we investigated the aggregation behaviors of asymmetric dyes in a DMSO/toluene mixture. Computational studies reveal the existence of combinatory geometries in our asymmetric dyes. From the experimental results, we found that there is significant dominance of one geometry over another. In the case of **T-X-J**, at 99% toluene fraction in DMSO, the resulting fluorescence intensity neither enhanced as high as **T-X-T** nor quenched as much as **J-X-J**, but it fell in between them. This proves the existence of dual AIE+ACQ geometry in a single scaffold. However, the dominant ACQ from **J** slightly quenched the initial intensity of **T-X-J** by 1.1-fold in aggregation (Figure 3D). Interestingly, in **T-X-BJ**, we found the dominant AIE character of the **T** unit over the anti-ACQ of the **BJ** unit at 99% of toluene fraction in DMSO. In addition, the resulting emission intensity fell between **T-X-T** and **BJ-X-BJ** with a dominant AIE enhancement by 1.4-fold of its initial intensity (Figure 3E). This result again proved the existence of dual AIE+anti-ACQ geometry in a single scaffold. As a control asymmetric dye, **J-X-BJ** was found to be severely quenched like **J-X-J** or **BJ-X-BJ** in the aggregation state (Figure S3), which again highlights the significance of the right molecular combination in modulating emission performance at aggregation.

Further substituting “O” with “Si” atom in xanthene cores can red-shift their emission wavelength while retaining similar properties, confirming the universality of our design. As shown in Figure S4, **T-Si-T** and **T-Si-BJ** exhibited prominent AIE with enhanced intensity by 3-fold and 1.25-fold, whereas **BJ-Si-BJ** showed ACQ by 1.17-fold at 99% toluene fraction in DMSO. Taken together, the theoretical and experimental analyses established clear relationships between geometry and emission intensity across the symmetric and asymmetric xanthene dyes. The dihedral angle-driven geometries, ranging from twisted (**T**), planar (**J**), and pseudoplanar (**BJ**) motifs, govern the balance between π–π interactions and conformational restriction in the aggregated state. These structural features ultimately dictate whether a dye exhibits AIE, ACQ, or anti-ACQ behavior, and asymmetric scaffolds enable the coexistence of dual photophysical responses within a single molecule. We then explore how these aggregation-dependent properties can be further manipulated by a strategic interaction between dye and polymer matrix in aggregates.

### Polymer-Induced Noncovalent Conformation Lock (PI-NoCL) in Aggregates

We then employ a polymer matrix to induce intermolecular interactions with dye molecules and thereon regulate the dihedral angle, dye packing, molecular geometry, and emission yield in the aggregated state. Our design aims to (1) suppress the low-frequency torsional motion of the **T** tolyl group (the dominant nonradiative pathway) by exploiting noncovalent interactions (dipole–dipole alignment, C–H···O hydrogen bonds, and C–H···π interactions) between dye and polymer to restrict active molecular motion and conformationally lock the twisted geometry; (2) achieve site-specific conformational locking through strategic polymer design that preferentially immobilizes the **T** units in asymmetric xanthene dyes; (3) reduce random dye aggregation by incorporating sterically hindered, nonpolar segments act as anti-ACQ units. To realize this concept, we designed and synthesized a series of alternating copolymers composed of two distinct monomer units: **Pttc-TTQ**, **PFC**_**8**_ **-TTQ**, and **Pttc-PFC**_**8**_ (Figure 4A). These polymers were employed to elucidate the role of individual monomer units in achieving PI-NoCL in the aggregated state. In this design, the **Pttc** unit functions as an anti-ACQ component that mitigates random dye aggregation, whereas the **TTQ** unit serves as the NoCL element responsible for locking active molecular motions. **TTQ** unit is enriched with heteroatoms for the formation of multiple noncovalent interactions with the tolyl moiety, which in turn can restrict the conformational flexibility.^46,47^ On the other hand, **Pttc** is a sterically hindered and inert unit that suppresses random aggregation while allowing site-specific interactions between the tolyl group and **TTQ**. For comprehension, we also designed and synthesized **PFC**_**8**_ **-TTQ** by replacing sterically hindered **Pttc** with a relatively planar **PFC**_**8**_ unit and **Pttc-PFC**_**8**_ by replacing the heteroatom-rich **TTQ** unit with the **PFC**_**8**_ unit (devoid of heteroatom).

**Figure 4:**
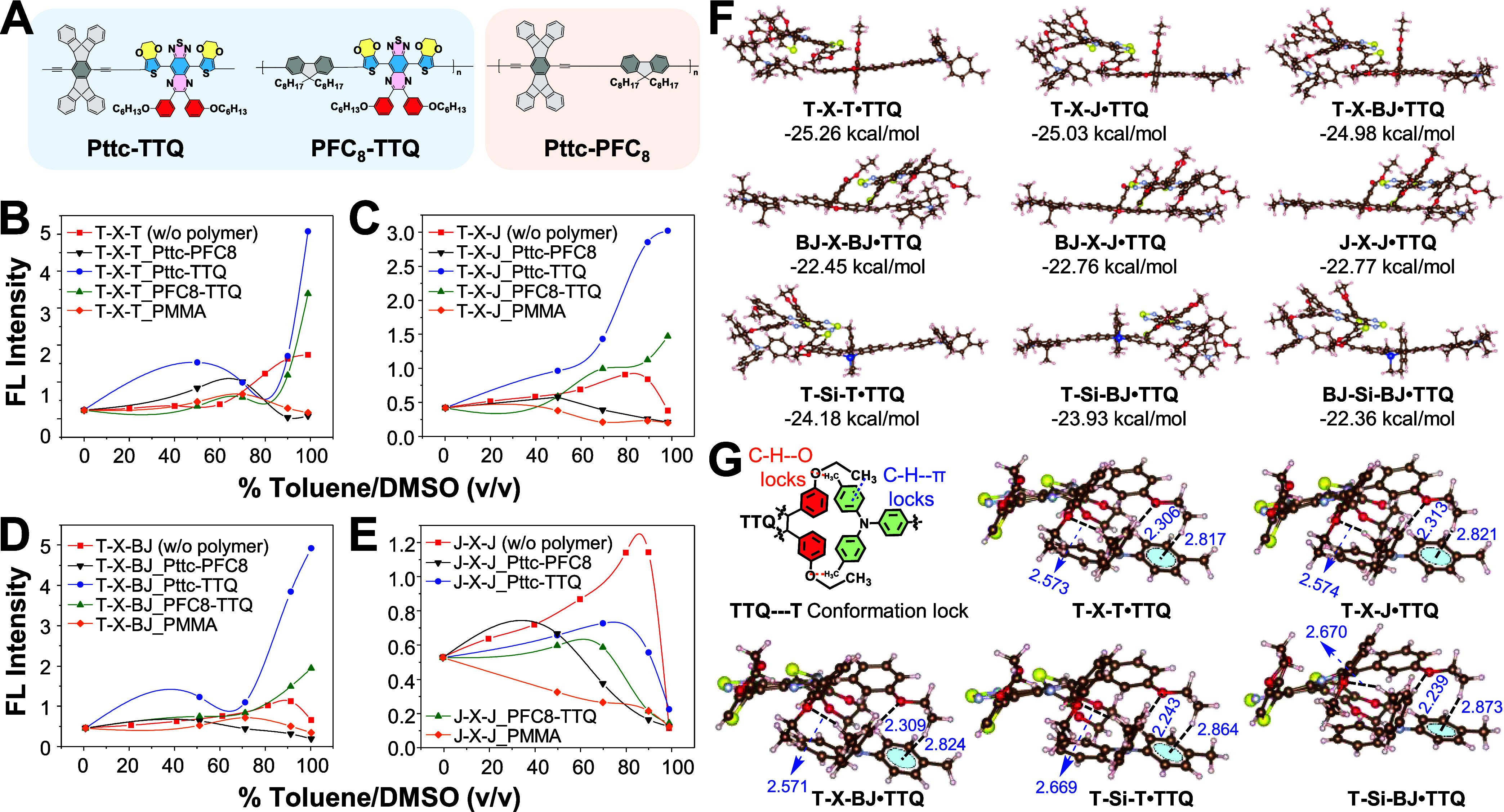
(A) Chemical structures of polymers used for PI-NoCL interactions with **T-X-T**, **T-X-J**, **T-X-BJ**, **and J-X-J**. (B-E) Change of fluorescence intensity with the increase in toluene fraction in DMSO for **T-X-T**, **T-X-J**, **T-X-BJ**, and **J-X-J** with and without polymer interaction. (F) Optimized structures and BSSE-corrected binding energy of low-energy **Xanthene·TTQ** complexes at the M06-2*X*/6-31G*­(0 K)/6-31G* level. (G) Illustration of partial structures of low-energy **Xanthene·TTQ** complexes. Brown, light blue, red, yellow, and white are carbon, nitrogen, oxygen, sulfur, and hydrogen atoms, respectively. The values in the G are the distances of two C–H­(of **T**)···O­(**TTQ**) and C–H­(**TTQ**)···π­(of **T**) with units in Å.

First, we experimentally mimicked the aggregation state using a DMSO/toluene mixture by choosing **T**-containing xanthene as the model dye because it contains the freely rotating tolyl units and **J-X-J** as the control dye. Later, we analyzed how xanthene dyes behave in the presence and absence of polymers, including a control polymer PMMA. As shown in Figure 4B–E, we found that **T-X-T** packed with heteroatom-rich polymer, **Pttc-TTQ** and **PFC**_**8**_ **-TTQ**, exhibited enhanced emission intensity of 15-fold and 12-fold, respectively, at 99% toluene fraction in DMSO, 2–3 times higher than **T-X-T** dye aggregate (5.6-fold) without polymer. In contrast, **T-X-T** with the **Pttc-PFC**_**8**_ polymer (devoid of heteroatom) showed an emission decrease by 1.8-fold (see Figure 4B). The results indicate that the **TTQ** unit somehow interacted with **T-X-T** in the aggregated state. Furthermore, we performed the same experiments with the asymmetric xanthenes, **T-X-J** and **T-X-BJ**, and found that asymmetric xanthenes, which were initially nonemissive in aggregates, exhibited a sharp emission increase after interacting with **Pttc-TTQ** and **PFC**_**8**_ **-TTQ** polymers (Figure 4C,D). As such, **T-X-J** and **T-X-BJ** showed 7.39- and 10-fold enhancement with **Pttc-TTQ** polymer, and 3.5- and 4-fold enhancement with **PFC**_**8**_ **-TTQ** polymer, respectively, in aggregates. On the contrary, as shown in Figure 4E, no such polymer-induced emission in **J-X-J** could be observed, where a sharp drop in emission intensity appeared in aggregation with or without polymers. The slight reduction of ACQ of **J-X-J** with polymer compared with dye alone might be attributed to the anti-ACQ effect of the polymer itself. We also examined an additional control polymer lacking specific functional groups (PMMA), and no enhancement effect was detected. These results motivate us to understand the detailed interactions between dyes and polymers both experimentally and computationally.

### Experimental and Computational Approach

To provide direct experimental support for the proposed noncovalent conformational locking between **TTQ** and **T** units, we performed 2D ROESY (Figure S5) and temperature-dependent ^1^H NMR analyses, together with appropriate control experiments. In the **T-X-T·TTQ** system, the 2D ROESY spectrum shows distinct cross-peaks between the aliphatic methyl protons (∼2.34 ppm) and aromatic protons (∼7.07–7.13 ppm), indicating through-space intermolecular proximity within ∼3–5 Å, consistent with C–H···π contacts. These correlations are absent in the single-component **T-X-T** control 2D NOESY (Figure S6), confirming that the observed short-range interactions arise from **TTQ** incorporation rather than intrinsic intrachain folding or nonspecific self-association. Complementary variable-temperature ^1^H NMR (Figure S7) further reveals a reversible and site-specific chemical-shift change of the tolyl −CH_3_ protons only in the **T-X-T+Pttc-TTQ** coprecipitated dye, whereas a negligible variation is observed for **T-X-T** alone. The downfield shift at lower temperature and upfield shift upon heating are consistent with thermally modulated weak C–H···O interactions and associated noncovalent conformational locks. Next, to get a deeper understanding of the interactions, we proposed a computational model that explains the interaction between the **T** and **TTQ** units as described below.

The **Pttc** segment in the **Pttc–TTQ** polymer serves primarily as an inert scaffold. Therefore, to streamline the theoretical analysis, our discussion focuses exclusively on the stacking interactions between the **TTQ** unit and the donor moieties (**T**, **J**, and **BJ**). For computational efficiency, the alkoxy substituent OC_2_H_5_ was used in place of OC_6_H_13_ on the **TTQ** unit, as this substitution has a negligible influence on the electronic structure or stacking behavior. The optimized structure and corresponding dipole moment of **TTQ** are illustrated in Figure S8. The stacking configurations between **TTQ** and the donor units were modeled by aligning the dipole moment of **TTQ** either parallel or antiparallel to the top or bottom face of the donor. Using the **T-X-T·TTQ** complex as a representative example, four distinct stacking modes are possible. In comparison, the asymmetric **T-X-J·TTQ** complex allows for eight unique stacking arrangements. The optimized geometries of these complexes are presented in Figure S9. The lowest-energy configuration of the **Xanthene·TTQ** complex is shown in Figure 4F, which serves as a model to elucidate the structural adaptation of the xanthene framework within the aggregation. This configuration provides key insights into how xanthene dye as a donor and **TTQ** as an acceptor stacking modulate the molecular conformation and, consequently, the emission behavior in the aggregated state.

The results indicate that **TTQ** stacking on **T** is significantly more stable than that on **J** or **BJ** (Figure 4F). This enhanced stability arises when the negative end of the **TTQ** dipole moment is oriented toward the xanthene core, suggesting a favorable interaction between **TTQ** and **T**-containing xanthenes. To better elucidate the structural influence of **TTQ** on **T** within the **Xanthene·TTQ** complex, **TTQ** was incorporated into various configurations, **T-X-T**, **T-X-J**, and **T-X-BJ**, and both **T** and **TTQ** were subsequently extracted for structural analysis (Figure 4G). The results indicate that **TTQ** contains two ether oxygen atoms that act as hydrogen bond acceptors, forming two C–H···O hydrogen bonds with the tolyl hydrogens of **T**. Additionally, the methyl hydrogen of **TTQ** participates in a C–H···π interaction with another tolyl group of **T**. For instance, in the **TXT·TTQ** complex shown in Figure 4G, the observed distances are 2.306, 2.573, and 2.817 Å, respectively. These interactions effectively restrict the rotational freedom of the phenyl group in **T** and, thus, enhance the emission intensity in aggregates. Such conformation locks were also observed in both **T-X-J** and **T-X-BJ**, which are selective to the **T** unit in asymmetric xanthenes. Consequently, they exhibited two C–H···O hydrogen bonds and one C–H···π interaction at distances 2.574, 2.313, 2.821 and 2.571, 2.309, 2.824 Å, respectively, aligning with the moderate enhancement of emission intensity compared to **T-X-T** observed in the experimental result. In contrast, no such polymer interactions were observed in the case of **J**- or **BJ**-containing xanthenes, which further validates our experimental observations. Together with theoretical calculations and comparative polymer controls (**Pttc-PFC**_**8**_ and **PMMA**), these spectroscopic results provide experimental evidence of persistent short-range intermolecular contacts in Pdots. While additional matrix rigidification effects cannot be excluded, the data support that recurrent C–H···π/C–H···O interactions contribute to conformational locking and reduced nonradiative relaxation, thereby enhancing fluorescence brightness. Leveraging these well-defined dye–polymer interactions, we next investigated the influence of NoCLs on the photophysical properties of the xanthene dyes in nanoparticles.

### Preparation of Nanoparticles and Their Optical Properties

We next prepared polymer dots (Pdots) for all xanthene dyes using **Pttc-TTQ** and DSPE-PEG, as illustrated in Figure 5A. The solid-state environment within the Pdots is expected to further strengthen the intermolecular interactions observed in the DMSO/toluene system. We subsequently investigated the photophysical properties of the xanthene Pdots in aqueous medium and summarized the influence of **Pttc-TTQ** interactions on the photophysical properties of both symmetric and asymmetric xanthenes (see Table 3).

**Figure 5:**
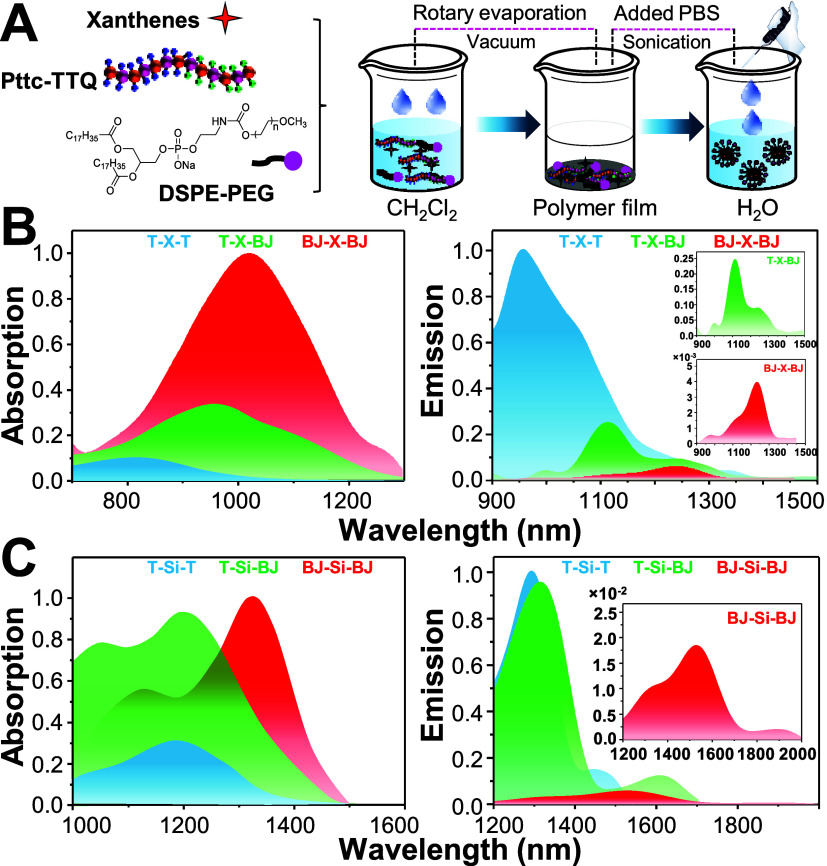
(A) Schematic of preparation of polymer dots (Pdots) by the miniemulsion method. (B) Absorption and emission profiles of symmetric and asymmetric **T-X-T**, **T-X-BJ**, and **BJ-X-BJ** as Pdots in aqueous medium. (C) Absorption and emission profiles of **T-Si-T**, **T-Si-BJ**, and **BJ-Si-BJ** as Pdots in aqueous medium.

**Table 3:** Photophysical Properties of Symmetric and Asymmetric Xanthene Pdots Assembled with Pttc-TTQ/mPEG-DSPE in Aqueous Medium and the Dihedral Angle of Linker (C = C) Relative to Donor (**T**, **J**, and **BJ**) (α1 and β2) or Xanthene Core (α2 and β1) of All Low-Energy **Xanthene·TTQ** Complexes

**experimental results**						**computational analysis**			
**dye**	λ_max_^abs^ (nm)	λ_max_^em^ (nm)	ε_max_ × 10^5^ (M^–1^ cm^–1^)	Φ (%)	brightness (M^–1^ cm^–1^)	α1	β1	β2	α2
**T-X-T.TTQ**	826	960	0.003	2.523	7.56	–23.4	–19.9	–0.6	0.9
**T-X-J.TTQ**	960	1102	0.013	0.333	4.33	–23.9	–20.0	–0.2	–0.2
**T-X-BJ.TTQ**	968	1105	0.011	0.448	4.93	–23.9	–19.8	–0.4	–1.0
**J-X-J.TTQ**	1014	1220	0.053	0.004	0.21	0.5	0.9	7.8	–3.1
**J-X-BJ.TTQ**	1017	1220	0.047	0.008	0.37	–1.0	–0.1	7.9	–3.0
**BJ-X-BJ.TTQ**	1025	1240	0.044	0.011	0.48	–0.3	–0.4	9.6	2.7
**T-Si-T.TTQ**	1190	1290	0.110	0.050	5.57	–20.5	–14.5	1.0	1.5
**T-Si-BJ.TTQ**	1220	1320	0.290	0.048	14.1	–20.7	–14.9	1.2	–0.5
**BJ-Si-BJ.TTQ**	1360	1555	0.310	0.001	0.34	0.0	1.4	9.2	9.8

Interestingly, we found that **T-X-T** Pdots exhibited an exceptionally high ϕ of 2.523% in aqueous solution, which is >200 times higher than **T-X-T** in CH_2_Cl_2_ (ϕ = 0.012%) (Table S2). However, it showed a substantial blue shift in both absorption and emission wavelengths from 884 and 1220 nm in CH_2_Cl_2_ to 850 and 960 nm in H_2_O (Figure 5B), along with very low molar absorptivity from 60,500 to 300 M^–1^ cm^–1^. Computational analysis revealed that when **T-X-T** interacts with **Pttc-TTQ** polymer in Pdots, the dihedral angle from **T** to C = C and C = C to xanthene core conformationally twisted more than **T-X-T** in CH_2_Cl_2_ and aggregates, and the resulting dihedral angles α_1_ and β_1_ twisted from −14.1 and 11.3° to −23.4 and −19.9°, which in turn disrupted the π-delocalization from donor **T** to the xanthene core and blue-shifted their absorption and emission wavelengths (Table 3 and Figure S10). The distortions in α_1_ and β_1_ originate from the combined effects of the two C–H···O hydrogen bonds and the single C–H···π interaction, which together lock the conformation and prevent free rotation. Likewise, the ε drops significantly due to the twisted geometry. Therefore, the beneficial NoCLs in **T-X-T** indeed increase the ϕ significantly but blue-shift λ_abs_, λ_em_, and low ε. We further performed quantitative kinetic analysis to examine the fluorescence quantum yield enhancement of dye **T-X-T** upon aggregation in Pdots. The calculations are based on experimentally measured fluorescence quantum yields and time-resolved fluorescence lifetimes, enabling the extraction of radiative (*k*_r_) and nonradiative (*k*_nr_) decay rate constants. As shown in Figure S11 and Table S3, in the solution state (CH_2_Cl_2_), **T-X-T** shows an extremely low quantum yield (ϕ = 0.012%) and an amplitude-weighted average lifetime (⟨τ ⟩) of 1.6 ± 0.4 ps with *k*_r_ = 8.1 × 10^7^ s^–1^ and a very large *k*_nr_ = 6.8 × 10^11^ s^–1^, mainly because the tolyl unit in the **T-X-T** segment possesses active rotational freedom, enabling strong intramolecular motion and efficient nonradiative relaxation. Upon interaction with the **TTQ** unit, **T-X-T** Pdots exhibit a fast component (τ_1_) of 2.1 ± 0.1 ps and a slow component (τ_2_) of 25.3 ± 0.7 ps. With the slow component lifetime, the *k*_r_ was estimated to be (1.00 ± 0.05) × 10^9^ s^–1^, about 12 times larger than that of **T-X-T** in solvent (8.1 × 10^7^ s^–1^), while *k*_nr_ is (3.8 ± 0.1) × 10^10^ s^–1^, about 18 times slower than that in solvent (6.8 × 10^11^ s^–1^). This latter result indicates that the increase of ϕ is significantly contributed by the suppression of nonradiative processes via conformational locking, whereas for **T-X-T**, when prepared with PMMA as Pdots, the lifetime was found to be less than 1 ps, which exhibits similar nonradiative decay like **T-X-T** in CH_2_Cl_2_, due to the absence of such noncovalent interactions. Overall, **TTQ**-induced conformation locks suppress rotationally driven nonradiative loss via structural locking, as quantitatively reflected in the decrease in *k*_nr_.

In contrast, symmetric ACQ-based **J-X-J** and **BJ-X-BJ** Pdots displayed extremely low ϕ values of 0.004% and 0.011%, respectively, consistent with the aggregation behavior observed in DMSO/toluene due to severe ACQ in aqueous medium as compared to in CH_2_Cl_2_ (**J-X-J**, ϕ = 0.029%; **BJ-X-BJ**, ϕ = 0.024%). However, owing to their planar geometry (Table 3), **J-X-J** and **BJ-X-BJ** Pdots exhibit longer λ_abs_, λ_em_, and higher ε values as compared to **T-X-T** Pdots. Overall, symmetric AIE (**T-X-T**) Pdots appear to be the brightest but shortest emission, which makes it less useful for the NIR-IIb imaging. Although symmteric ACQ (**J-X-J**) or anti-ACQ (**BJ-X-BJ**) Pdots display the longest optical wavelength and high ε, they might not be bright enough for *in vivo* applications. This finding emphasizes the inherent limitation of symmetric AIE or ACQ xanthene dyes, which strive to simultaneously achieve the optimal absorption and emission wavelengths and brightness.

To circumvent this dilemma, asymmetric Pdots **T-X-J** and **T-X-BJ** showed a phenomenal optical trade-off between the optical wavelength and brightness. Taking advantage of their asymmetric structure, **TTQ** can interact selectively with the **T** unit to enhance the ϕ, whereas planar **J** or **BJ** can contribute to longer λ_abs_, λ_em_, and higher ε. Computational data revealed that **TTQ** interacts selectively with the **T** unit to alter the dihedral angle at one end of the **T-X-J** and **T-X-BJ** xanthene dyes and thus twists their conformation asymmetrically in the xanthene backbone. As a result, **T-X-J** and **T-X-BJ** Pdots exhibited twisted dihedral angles α_1_ and β_1_ of −23.9°, −20.0°, and −23.9°, −19.8°, respectively, at the **T** end, and very planar geometry with dihedral angles α_2_ and β_2_ of −0.2°, −0.2°, and −0.4°, −1.0°, respectively, at the **J**/**BJ** end. Besides, **T-X-J** and **T-X-BJ** exhibited ϕ of 0.333 and 0.448% in aqueous medium which is 20–30 times higher in CH_2_Cl_2_ (**T-X-J**, ϕ = 0.017%; **T-X-BJ**, ϕ = 0.014%). Unlike **T-X-T**, **T-X-J** and **T-X-BJ** Pdots showed balanced λ_abs_, λ_em_, and ε of 960 nm, 1102 nm, and 1300 M^–1^ cm^–1^, and 968, 1105, and 1100 M^–1^ cm^–1^, respectively (Figure 5B and Table 3). In general, asymmetric xanthenes solved the trade-off between wavelength and brightness and highlighted the importance of asymmetric AIE+ACQ and AIE+anti-ACQ design using PI-NoCL strategy. However, the current optical wavelength is still far from achieving NIR-IIb windows; thus, we further extend the absorption and emission wavelength by replacing “O” with “Si” in the xanthene core.

In CH_2_Cl_2_, Si-xanthenes exhibited notable red-shifted excitation >1200 nm and emission >1500 nm which makes them promising candidates for deep-tissue NIR-IIb imaging. We applied the PI-NoCL strategy to Si-substituted xanthenes to examine the universality of our design. As shown in Figure 5C, **T-Si-T** Pdots displayed the similar behaviors with its O-based analogous **T-X-T** with a emission maximum at 1290 nm and a moderate brightness of 5.57 M^–1^ cm^–1^. Correspondingly, **BJ-Si-BJ** Pdots suffered from severe π–π quenching with a weak emission peak at 1555 nm but possessed minimal brightness (0.34 M^–1^ cm^–1^), closely resembling the trend observed for **BJ-X-BJ**. In contrast, asymmetric **T-Si-BJ** Pdots, which synergistically combines efficient **Pttc–TTQ** interaction via the **T** unit and enhanced conjugation from the **BJ** unit achieved the most favorable trade-off across all studied xanthene systems with extended excitation and emission at 1220 and 1320 nm, respectively, along with the highest brightness recorded among all xanthene-based Pdots (14.1 M^–1^ cm^–1^). To the best of our knowledge, **T-Si-BJ** is considered the longest and brightest NIR-IIb asymmetric dye reported to date.

### *In Vivo* Whole-Body Imaging in Mice Using Pdots

To elucidate the importance of our trade-off design, we systematically compared the real-time brightness of symmetric and asymmetric xanthene Pdots under 1064 nm excitation using different long-pass filters (LPFs). Representative O-xanthene Pdots (**T-X-T**, **BJ-X-BJ**, and **T-X-BJ**) and Si-xanthene Pdots (**T-Si-T**, **BJ-Si-BJ**, and **T-Si-BJ**) were selected for detailed evaluation. At identical concentrations and excitation conditions, **T-X-BJ** exhibited superior real-time brightness relative to those of both **T-X-T** and **BJ-X-BJ** at 1300 and 1400 nm LPFs (Figure 6A,B). Although **T-X-T** displayed the highest experimental brightness among the O-xanthenes, its negligible emission beyond 1300 nm resulted in diminished real-time brightness, consistent with its steady-state emission profile (Figure 5B). In contrast, **BJ-X-BJ** showed moderate brightness under 1300 nm LPF owing to its longer emission wavelength. However, its signal declined sharply at 1400 nm as a result of its very low ϕ within this detection window, further validating the experimental wavelength and brightness. By comparison, the asymmetric **T-X-BJ** Pdots leveraged both a high ε at 1064 nm excitation and a moderate ϕ to enable strong brightness at 1300 nm but only moderate intensity at 1400 nm. Despite its favorable performance within the O-xanthene series, **T-X-BJ** exhibited very weak emission beyond 1400 nm, limiting its applicability for deep-tissue *in vivo* imaging.

**Figure 6:**
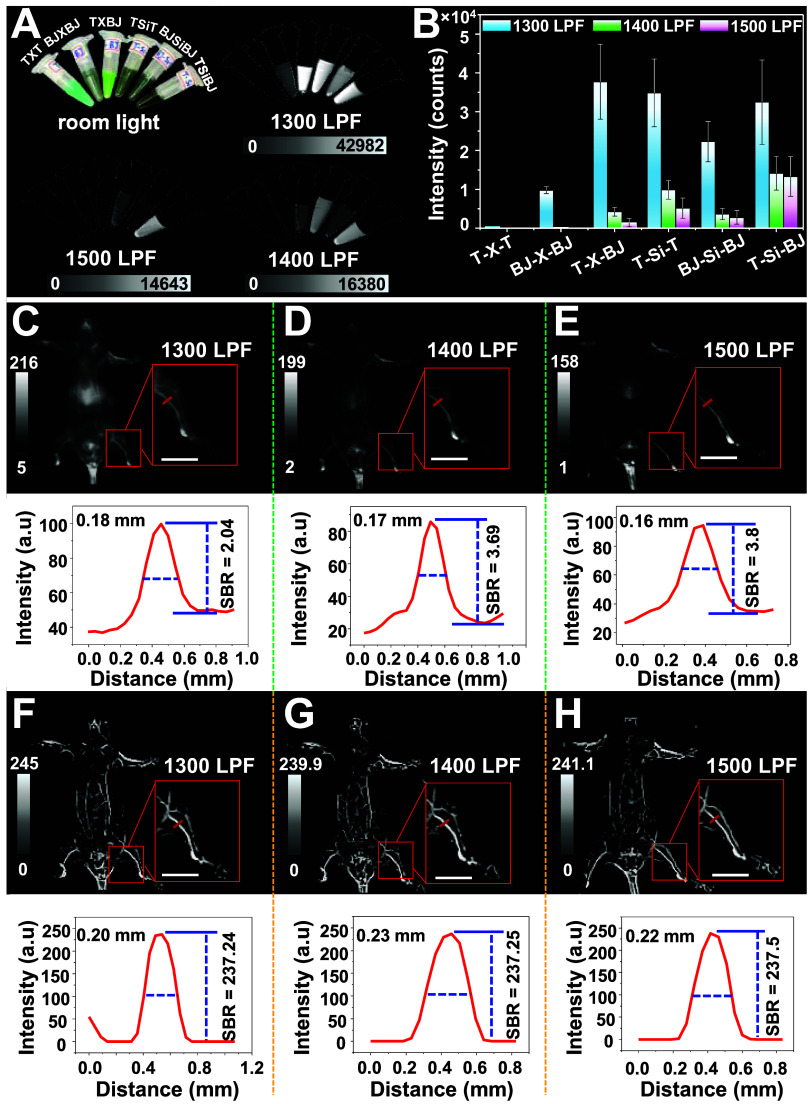
Comparison of (A) **T-X-T, BJ-X-BJ**, **T-X-BJ**, **T-Si-T**, **BJ-Si-BJ**, and **T-Si-BJ** Pdots assembled with Pttc-TTQ/mPEG-DSPE in water at the same concentration of 5 mg/mL under irradiation of a 1064 nm laser (100 mW cm^–2^) with different LPFs. (B) Mean fluorescence intensities of **T-X-T, BJ-X-BJ**, **T-X-BJ**, **T-Si-T**, **BJ-Si-BJ**, and **T-Si-BJ** Pdots in (A). (C-E) Whole-body fluorescence imaging of vascular structures in mice at the supine position injected by **T-Si-BJ** Pdots with 1300, 1400, and 1500 nm LPFs, respectively (upper panels) and their corresponding cross-sectional intensities along the red lines (bottom panel). (F–H) AI-enhanced NIR-II images based on the original images in (C-E) and their corresponding cross-sectional intensities along the red lines in (F–H) at various LPFs (bottom panel). The scale bars are 5 mm. Note that the images were obtained from the same mouse under identical acquisition settings and comparable time points, except for the use of different long-pass filters.

In comparison, the **Si**-series demonstrated markedly enhanced brightness across the 1300–1500 nm range owing to their red-shifted emission maxima (Figure 5C). However, **BJ-Si-BJ** displayed substantial ACQ, yielding relatively weak emission beyond 1400 nm, despite its emission peak at 1555 nm. Conversely, AIE active **T-Si-T** retained strong intensity under 1400 nm LPF and moderate brightness at 1500 nm as reflected in both its spectral and real-time brightness profiles (Figures 5C and 6B). The combination of enhanced brightness and an extended emission wavelength in **T-Si-BJ** culminates in exceptional real-time brightness across all LPFs, highlighting the efficacy of our molecular design strategy in achieving robust brightness within the NIR-IIb window. Based on these results, **T-Si-BJ** Pdots was selected as the brightest asymmetric dye for subsequent in vivo deep-tissue imaging studies.

Preceding *in vivo* experiments, the cytotoxicity and photostability of the **T-Si-BJ** Pdots were systematically evaluated. *In vitro* cytotoxicity assay (Figure S12) demonstrated negligible cellular toxicity, indicating excellent biocompatibility. Next, **T-Si-BJ** Pdots were intravenously administered through tail vein injection in live mice, followed by real-time whole-body imaging under a 1064 nm laser excitation. Within 5 min postinjection, Pdots exhibited rapid systemic distribution for immediate visualization of the vascular network (Figure 6C–E). To further evaluate imaging performance, the signal-to-background ratio (SBR) of **T-Si-BJ** Pdots was quantified from vascular structures under varying LPFs. The results showed the superior imaging capability of the NIR-IIb region compared with NIR-IIa or NIR-IIx windows, which is essential for achieving optimal image contrast. Specifically, the SBR measured at 1300 nm LPF was 2.04, likely influenced by background scattering and autofluorescence (Figure 6C). In contrast, the SBR increased significantly to 3.69 and 3.80 under 1400 and 1500 nm LPFs, respectively (see Figure 6D–E), highlighting the enhanced imaging contrast attainable in the NIR-IIb window. While the 1500 nm filter improved the SBR ratio, the lower brightness of **T-Si-BJ** Pdots beyond 1500 nm degraded the image clarity, demonstrating a key challenge that demands further enhancement of SBR for high-contrast deep-tissue imaging. Thus, we utilized AI-driven imaging to enhance the SBR of blood vessels in mice.

### AI-Powered Enhancement of Vascular Detail in Mice Imaging

To address the aforementioned challenge, we employed AI-driven image processing to enhance the quality of the in vivo NIR-IIb imaging. The images presented in Figure 6F (1300 nm LPF), 6 **G** (1400 nm LPF), and 6 **H** (1500 nm LPF) were processed using a diffusion model followed by AI-based enhancement. The resulting outputs, denoised using a trained diffusion model-based HDR (DMHDR),^48^ exhibit significantly improved vascular brightness and contrast while preserving the anatomical integrity of the structures. The combined use of the diffusion model and AI-driven vessel-enhancement algorithms markedly improved imaging resolution, contrast, and SBR. Quantitative analysis of fluorescence intensity profiles (Figure 6F–H, bottom panels) revealed a progressive enhancement of image quality across increasing LPFs. In unprocessed images, the brightness of hindlimb blood vessels declined from 1300 to 1500 nm LPF (Figure 6C–E). In contrast, AI-processed images sharply enhanced these vessels, underscoring the importance of AI-assisted imaging for overcoming weak signals in longer emissive windows. At 1500 nm LPF, vessel structures were clearly delineated with an SBR of 237.5. It should be noted that the high SBR values in Figure 6F–H represent the contrast ratio resulting from algorithmic background suppression and should be distinguished from the intrinsic optical SBR of the probe shown in Figure 6C–E. It is also worth mentioning that this substantial increase in SBR is a mathematical consequence of algorithmic background suppression (where the denominator *I*_background_ approaches zero) rather than a physical amplification of the fluorescence photon count. Accordingly, the AI-enhanced NIR-IIb images provide high-contrast, referential vascular maps that support structural interpretation while remaining computational reconstructions of the original raw data. This demonstrates the potential of AI-enhanced NIR-IIb imaging for extracting high-contrast vascular maps from low-dose organic probes.

## Conclusion

To summarize the above results and discussion, we propose the expansion of conventional symmetric ACQ or AIEgens to asymmetric xanthene-based AIEgens for broadening the synthetic diversity and promoting emission in the NIR-IIb window. The concept lies in embedding the planar and twisted chromophores in a single scaffold for unifying the complementary advantages of long λ_em_, high ε, and elevated ϕ. Bearing this aim, asymmetric xanthenes (**T-X-J**, **T-X-BJ**, and **T-Si-BJ**) were designed and synthesized. Moreover, embedding these dyes into a heteroatom-rich polymer matrix (**Pttc-TTQ**) establishes intermolecular NoCLs and thus enables the precise control of dihedral angles to significantly enhance ϕ in Pdots. The proof-of-concept is given experimentally by balancing long λ_em_, high ε from the **J** or **BJ** unit, and ultrahigh ϕ from **T** unit interacting with the **TTQ** matrix and computationally by assessing a smaller dihedral angle generated from planar and pseudoplanar **J** or **BJ** units and larger dihedral angle from twisted **T** unit due to C–H···π and C–H···O intermolecular NoCLs with the **TTQ** matrix. The superior optical wavelength of **T-Si-BJ** over **T-X-J** and **T-X-BJ** is attributed to “Si” incorporation in a xanthene scaffold that significantly lowers the LUMO by extending σ*−π* conjugation over SiMe_2_ unit. Conceptually, asymmetric xanthenes outperformed their symmetric analogues (**T-X-T**, **J-X-J**, **BJ-X-BJ**, **T-Si-T**, and **BJ-Si-BJ**) in this study, owing to their balanced optical wavelength and ultrahigh brightness, manifesting the versatility of asymmetric derivatives. **T-Si-BJ** was further prepared as Pdots to successfully illustrate its importance in obtaining high-contrast deep-tissue imaging in the NIR-IIb window, far reaching the NIR-IIb emitters in terms of synthetic diversity. We contend that the universality of this molecular design renders it broadly transferable to other scaffolds, and future efforts will aim to optimize noncovalent conformational locking by systematically screening broader donor-polymer pairs with tailored steric size, peripheral flexibility, and electronic density, thus charting a new course for the development of organic NIR-IIb and potentially NIR-III emissive systems for biomedical imaging and hence medical applications.

## Supplementary Material



## Notes

Details of experimental section, including synthetic procedures, characterization, computational approach, and photophysical measurements (PDF)
