# Supplementary material for: Asymmetric Xanthene with Noncovalent Conformation Locks to Attain High Fluorescence >1500 nm
Source: J Am Chem Soc. 2026 Apr 21;148(17):17764–78. doi: 10.1021/jacs.5c23313 (PMC13154214; doi:10.1021/jacs.5c23313)
Supplement: Supplementary file 1 [file ja5c23313_si_001.pdf]

# Asymmetric Xanthene with Noncovalent Conformation Locks to Attain High Fluorescence > 1500 nm

*Partha Chowdhury,[a†] Hsiu-Feng Lu,[b†] Hsin-Ting Huang,[a] Meng-Huan Liu,[a] Yen-Chang Chen,[a] Kai-Min Chan[c], Syue-Liang Lin,[d] Ricardas Rotomskis,[e] Simona Steponkiene,[e] Tung-Kung Wu,[f] Yu-Fen Huang,[c,g,h] Chao-Ping Hsu,[b]* and Yang-Hsiang Chan[a,j,k]**

[a]*Department of Applied Chemistry, National Yang Ming Chiao Tung University, Hsinchu, 300, Taiwan, R.O.C.*

[b]*Institute of Chemistry, Academia Sinica, Taipei, 115, Taiwan, R.O.C.*

[c]*Department of Biomedical Engineering and Environmental Sciences, National Tsing Hua University, Hsinchu, 300, Taiwan, R.O.C.*

[d]*Biomedical Department of Biotechnology and Laboratory Science in Medicine, National Yang Ming Chiao Tung University, Taipei, Taiwan*

[e]*Biomedical Physics Laboratory of National Cancer Institute, Baublio 3B, LT-08406, Vilnius, Lithuania*

[f]*Department of Biological Science, College of Engineering Bioscience, Center for Emergent Functional Matter Science, National Yang Ming Chiao Tung University, Hsinchu, 300, Taiwan, R.O.C.*

[g]*Institute of Analytical and Environmental Sciences, National Tsing Hua University, Hsinchu, 300, Taiwan, R.O.C.*

[h]*School of Pharmacy, College of Pharmacy, Kaohsiung Medical University, Kaohsiung 807, Taiwan, R.O.C.*

[i]*Center for Emergent Functional Matter Science, National Yang Ming Chiao Tung University, Hsinchu, 300, Taiwan, R.O.C.*

[j]*Department of Medicinal and Applied Chemistry, Kaohsiung Medical University, Kaohsiung, 807, Taiwan, R.O.C.*

[†]*Authors contributed equally to this work.*

## Supporting Information

# Table of Contents

**Experimental Procedures** ..... S3
Scheme S1 ..... S3
Scheme S2 ..... S6
Scheme S3 ..... S11
Scheme S4 ..... S14
Figure S1 ..... S19
Figure S2 ..... S19
Figure S3 ..... S20
Figure S4 ..... S20
Figure S5 ..... S21
Figure S6 ..... S21
Figure S7 ..... S22
Figure S8 ..... S23
Figure S9 ..... S23
Figure S10 ..... S24
Figure S11 ..... S24
Figure S12 ..... S25
Figure S13-S21 ..... S26-S30
Figure S22 ..... S30
Figure S23 ..... S30
Figure S24-S26 ..... S31-S32
Figure S27-S35 ..... S32-S36
Figure S36-S39 ..... S37-S38
Figure S40 ..... S39
Figure S41 ..... S39
Figure S42 ..... S40
Table S1 ..... S41
Table S2 ..... S41
Table S3 ..... S42
Table S4 ..... S43
Table S5 ..... S43
Table S6 ..... S44
Table S7 ..... S44
**Appendix ($^{1}$H-NMR, $^{13}$C-NMR, HR-Mass, and ESI-HRMS)** ..... S45-S83
**References** ..... S83

## Experimental Procedures

**Materials.** The chemicals used in the experiments were purchased from Alfa Aesar, Sigma-Aldrich, TCI, and Acros. All chemicals were used as received unless described otherwise. DSPE-based lipid mPEG-DPSE ($M_w$=2000) is obtained from Laysan Bio, Inc. High-purity water (18.2 MΩ•cm) was used throughout the experiment. All $^1$HNMR and $^{13}$CNMR spectra were recorded on Agilent 400-MR DD2, Agilent-VARIAN Vnmrs-600, and JEOL JNM-ECZ400S/L1 spectrometers. Compounds (**3-5**),[1] **Pttc**[2], **PFC$_8$Br**,[3] **TTQ**,[2] were synthesized as reported before.

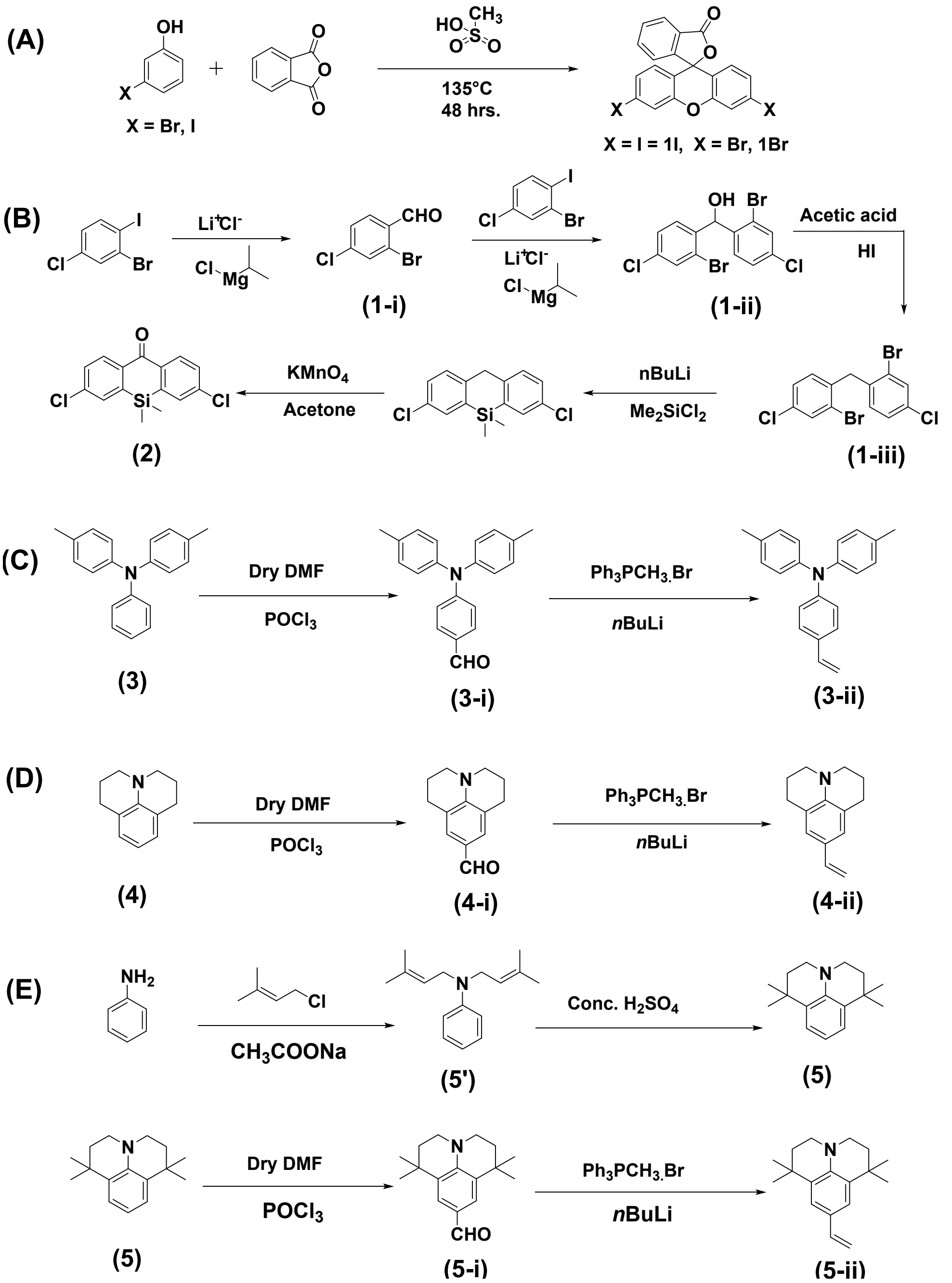

**Scheme S1**: Synthetic routes for donors and acceptors used in this study. (A, B) Oxygen and silicon-based xanthene. (C-E) Alkenes of nitrogen-based flexible and rigid donors.

*Synthesis of 3',6'-diiodo-3H-spiro[isobenzofuran-1,9'-xanthen]-3-one,* **[1 (I/Br)]**. In a 250 mL round-bottom flask, 3-iodophenol (5.5 g, 25 mmol), phthalic anhydride (1.9 g, 12.5 mmol), and methanesulfonic acid (12.5 mL) were added. The reaction mixture was heated to 135 °C under an air atmosphere and stirred continuously for 48 h. Upon completion, the reaction mixture was allowed to cool to room temperature and then poured into 600 mL of ice water with vigorous stirring. The resulting dark purple mixture was stirred for an additional 20 min, and the precipitated solid was collected by filtration. The crude solid was dissolved in dichloromethane and purified by flash column chromatography using DCM to afford the desired product as a white solid (1.7 g, 25% yield). *Compound 1I*: $^1$H NMR (400 MHz, $CDCl_3$) δ 8.04 (d, *J* = 7.8 Hz, 1H), 7.71 – 7.63 (m, 4H), 7.38 (d, *J* = 10.1 Hz, 2H), 7.11 (d, *J* = 7.5 Hz, 1H), 6.54 (d, *J* = 8.3 Hz, 2H). **Compoud 1Br**: $^1$H NMR (400 MHz, $CDCl_3$) δ 8.04 (d, *J* = 6.6 Hz, 1H), 7.75 – 7.60 (m, 2H), 7.49 (s, 2H), 7.19 (d, *J* = 8.6 Hz, 2H), 7.13 (d, *J* = 7.6 Hz, 1H), 6.70 (d, *J* = 8.4 Hz, 2H).

*Synthesis of 2-bromo-4-chlorobenzaldehyde,* **[1-i]**[4]. In a dry two-neck round-bottom flask, 2-bromo-4-chloro-1-iodobenzene (4.88 g, 15.4 mmol) was added, and the vessel was subjected to vacuum/nitrogen 3 times. Dry THF (50 mL) was added to the mixture and cooled to –78 °C. A solution of iPrMgCl·LiCl in THF (2.23 M, 6.89 mL, 15.4 mmol) was added dropwise, and stirred at –78 °C for 2 h, followed by the addition of dry DMF (3.5 mL, 15 mmol). The mixture was stirred at room temperature for 12 h, then quenched with saturated $NH_4Cl$. The mixture was extracted with DCM and brine, the organic layer was dried over $MgSO_4$, filtered, and concentrated under reduced pressure. The crude material was purified by silica gel column chromatography (DCM: hexane = 1:1), affording a light yellow solid (3.3 g, 95% yield). $^1$H NMR (400 MHz, $CDCl_3$) δ 10.30 (s, 1H), 7.86 (d, *J* = 8.4 Hz, 1H), 7.68 (d, *J* = 2.0 Hz, 1H), 7.43 (d, *J* = 6.5 Hz, 1H).

*Synthesis of bis(2-bromo-4-chlorophenyl)methanol,* **[1-ii]**[4]. In a dry two-neck round-bottom flask, 2-bromo-4-chloro-1-iodobenzene (4.68 g, 14.7 mmol) was added. the vessel was subjected to vacuum/nitrogen 3 times. Anhydrous THF (100 mL) was added, and the solution was cooled to –78 °C. A solution of iPrMgCl·LiCl in THF (2.23 M, 6.6 mL, 14.7 mmol) was added dropwise. After stirring at –78 °C for 2 h, a solution of compound **2** (3.2 g, 14.7 mmol) in dry THF (20 mL) was added. The reaction mixture was stirred at room temperature for 12 h. The reaction was concentrated under reduced pressure, and the residue was treated with diethyl ether and saturated aqueous $NH_4Cl$. The aqueous layer was extracted with $Et_2O$, and the combined organic layers were washed with brine, dried over anhydrous $MgSO_4$, filtered, and concentrated. The crude product was purified by silica gel column chromatography (100% DCM) to afford a white solid (5.1 g, 85% yield). $^1$H NMR (400 MHz, $(CD_3)_2CO$) δ 7.67 (d, *J* = 2.0 Hz, 2H), 7.46 - 7.38 (m, 4H), 6.28 (d, *J* = 5.2 Hz, 1H), 5.38 (d, *J* = 5.1 Hz, 1H).

*Synthesis of bis(2-bromo-4-chlorophenyl)methane,* **[1-iii]**[5]. In a round-bottom flask, compound **1-ii** (5.1 g, 12.5 mmol) was dissolved in acetic acid (40 mL), and hydroiodic acid (57 wt% in water, 7 g, 30 mmol) was added. The reaction mixture was heated to 125 °C and refluxed with stirring for 3 h. After cooling to room temperature, the mixture was treated with saturated aqueous $Na_2SO_3$, then extracted with ethyl acetate. The combined organic layers were neutralized with KOH, washed with saturated $NaHCO_3$, then brine, dried over anhydrous

$MgSO_4$, filtered, and concentrated under reduced pressure. The crude product was purified by silica gel column chromatography using 100% hexane to yield a white solid (4.1 g, 85% yield). $^1H$ NMR (400 MHz, $CDCl_3$) δ 7.62 (s, 2H), 7.21 (d, $J$ = 8.3 Hz, 2H), 6.90 (d, $J$ = 8.3 Hz, 2H), 4.11 (s, 2H).

*Synthesis of 3,7-dichloro-5,5-dimethyl-5,10-dihydrodibenzo[b,e]siline.* In a dry two-neck flask, compound **1-iii** (1.96 g, 4.97 mmol) was dissolved in dry $Et_2O$ (20 mL) under nitrogen. At 0 °C, n-BuLi (1.6 M in hexanes, 6.40 mL, 10.4 mmol) was added dropwise. After stirring for 2 h at 0 °C, the mixture was cooled to –78 °C, and $Me_2SiCl_2$ (0.72 mL, 5.97 mmol) was added. The reaction was warmed to room temperature and stirred for 2 h. It was quenched with saturated $NH_4Cl$, and the aqueous layer was extracted with $Et_2O$. The combined organics were washed with brine, dried ($MgSO_4$), filtered, and concentrated. Proceed to the next step without further purification.

*Synthesis of 3,7-dichloro-5,5-dimethyldibenzo[b,e]silin-10(5H)-one,* **[2]**[5]. 3,7-dichloro-5,5-dimethyl-5,10-dihydrodibenzo[b,e]siline was dissolved in acetone (40 mL) and purged with nitrogen for 10 min. $KMnO_4$ (1.62 g, 10.2 mmol) was added, and the mixture was stirred at room temperature for 90 min. The reaction mixture was then poured directly onto a thick silica gel layer and eluted with 100% DCM. The crude product was purified by silica gel column chromatography (DCM/hexane=1:5) to afford a white crystal (0.955 g, 37% yield). $^1H$ NMR (600 MHz, $CDCl_3$) δ 8.36 (d, $J$ = 8.1 Hz, 2H), 7.61 (d, $J$ = 2.8 Hz, 2H), 7.54 (dd, $J$ = 8.5, 2.2 Hz, 2H), 0.52 (s, 6H).

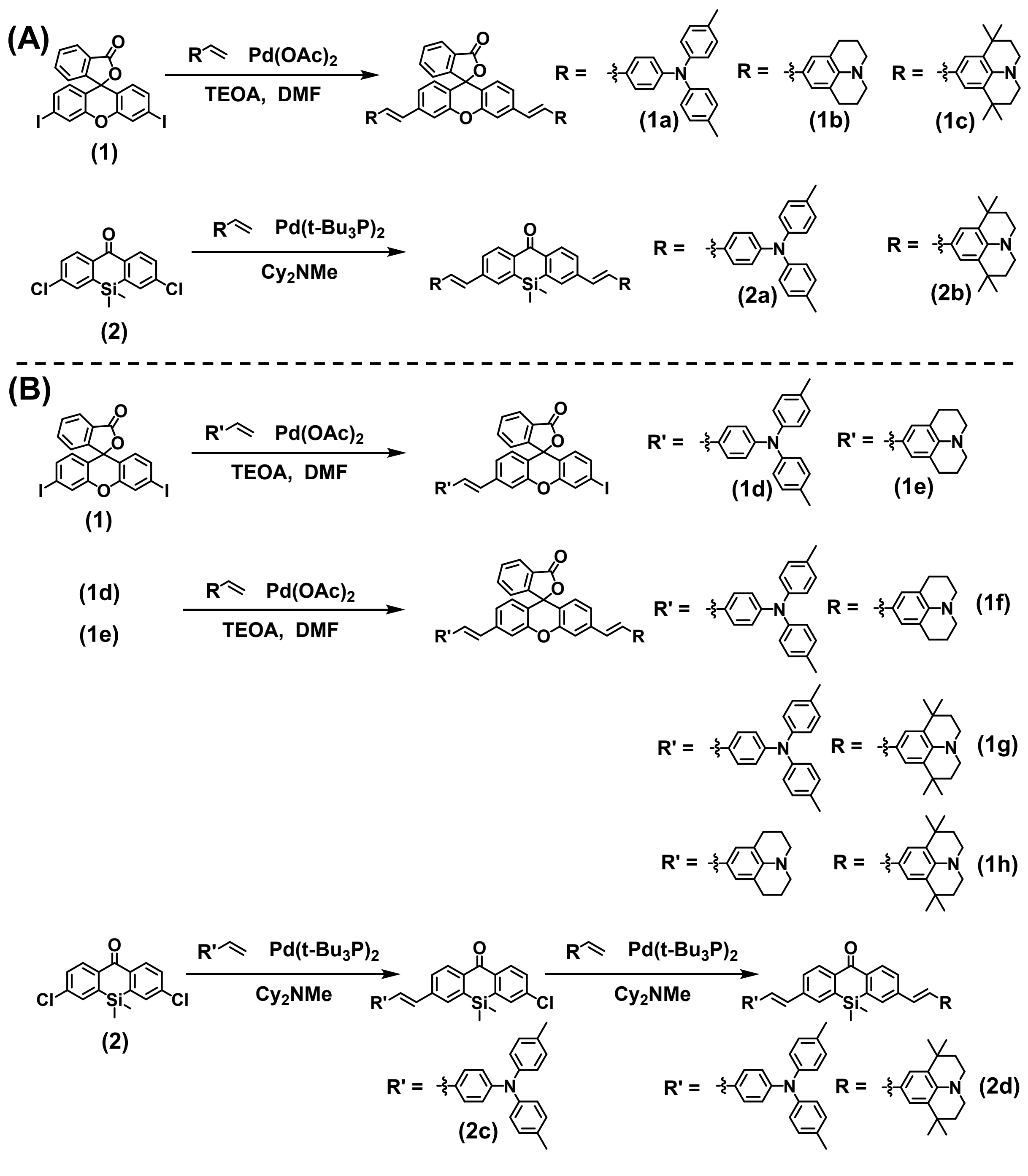


**Scheme S2**: Synthetic routes for the synthesis of symmetric and asymmetric xanthenes in this study. (A) Symmetric xanthenes, (B) Asymmetric xanthenes.

**Synthesis of Symmetric Xanthenes:**

*Synthesis of 3',6'-bis((E)-4-(di-p-tolylamino)styryl)-3H-spiro[isobenzofuran-1,9'-xanthen]-3-one,* **[1a]**. In a two-neck round-bottom flask, compound **3-ii** (600 mg, 2 mmol), compound **1** (550 mg, 0.99 mmol), triethanolamine (2.0 mL), and $Pd(OAc)_2$ (4 mg, 0.018 mmol) were added sequentially. Anhydrous DMF (10 mL) was then added to the mixture and heated to reflux at 130 °C for 12 hours. Upon completion of the reaction (as monitored by TLC), the mixture was allowed to cool to room temperature and extracted with DCM and brine solution. The organic layer was separated, dried over anhydrous $MgSO_4$, and filtered. The solvent was removed under

reduced pressure to afford a crude product, which was purified by silica gel column chromatography using hexane/DCM (1:1) as the eluent. The purified product was obtained as a yellow solid (202 mg, 36% yield). $^{1}$H NMR (400 MHz, $CDCl_3$) δ 8.05 (d, *J* = 8.0 Hz, 1H), 7.70 – 7.60 (m, 2H), 7.39 – 7.33 (m, 6H), 7.23 – 6.90 (m, 29H), 6.78 (d, *J* = 8.2 Hz, 2H), 2.32 (s, 12H). $^{13}$C NMR (101 MHz, $CDCl_3$) δ 169.56, 151.54, 148.30, 144.90, 140.59, 135.09, 132.96, 130.50, 129.94, 129.66, 128.15, 127.52, 125.14, 124.92, 124.75, 123.90, 121.97, 121.61, 117.13, 114.24, 82.63, 20.83. HRMS (FD, $[M]^+$) for $C_{64}H_{50}N_2O_3$ calcd. 894.38269, found: 894.38269.

*Synthesis of 3',6'-bis((E)-2-(2,3,6,7-tetrahydro-1H,5H-pyrido[3,2,1-ij]quinolin-9-yl)vinyl)-3H-spiro[isobenzofuran-1,9'-xanthen]-3-one,* **[1b]**. In a two-neck flask under nitrogen (Schlenk line), compound **4-ii** (550 mg, 2.7 mmol), compound **1** (500 mg, 0.9 mmol), triethanolamine (2.0 mL), and $Pd(OAc)_2$ (4 mg, 0.018 mmol) were added, followed by anhydrous DMF (10 mL). The mixture was refluxed at 130 °C for 12 h. After cooling, the reaction was extracted with DCM and a saturated NaCl solution. The organic layer was dried over anhydrous $MgSO_4$, filtered, and concentrated under reduced pressure. The crude product was purified by silica gel column chromatography (hexane/DCM, 1:1). Yellow solid was obtained after purification (216 mg, 43% yield). $^{1}$H NMR (400 MHz, $CDCl_3$) δ 8.04 (d, *J* = 9.7 Hz, 1H), 7.68 – 7.59 (m, 2H), 7.34 (d, *J* = 1.6 Hz, 2H), 7.19 – 7.09 (m, 3H), 7.03 (s, 6H), 6.84 – 6.72 (m, 4H), 3.20 – 3.15 (m, 8H), 2.77 (t, *J* = 6.4 Hz, 8H), 2.02 – 1.95 (m, 8H).

*Synthesis of 3',6'-bis((E)-2-(1,1,7,7-tetramethyl-2,3,6,7-tetrahydro-1H,5H-pyrido[3,2,1-ij]quinolin-9-yl)vinyl)-3H-spiro[isobenzofuran-1,9'-xanthen]-3-one,* **[1c]**. In a two-neck flask under a nitrogen atmosphere, compound **5-ii** (400 mg, 1.56 mmol), compound **1** (400 mg, 0.73 mmol), triethanolamine (2.0 mL), and $Pd(OAc)_2$ (4 mg, 0.018 mmol) were added, followed by anhydrous DMF (10 mL). The reaction mixture was heated to reflux at 130 °C for 12 h. After cooling to room temperature, the mixture was extracted with dichloromethane (DCM) and brine 5 times. The organic layer was separated, dried over anhydrous $MgSO_4$, filtered, and concentrated under reduced pressure. The crude product was purified by silica gel column chromatography using hexane/DCM (1:1) as the eluent. The product was obtained as a yellow solid (243mg, 60% yield). $^{1}$H NMR (400 MHz, $CDCl_3$) δ 8.04 (d, *J* = 9.7 Hz, 1H), 7.69 – 7.60 (m, 2H), 7.37 (s, 2H), 7.22 (s, 4H), 7.14 (d, *J* = 8.3 Hz, 2H), 7.07 (d, *J* = 16.2 Hz, 2H), 6.86 – 6.71 (m, 4H), 3.24 – 3.18 (m, 8H), 1.77 (t, *J* = 6.0 Hz, 8H), 1.33 (s, 24H). $^{13}$C NMR (101 MHz, $CDCl_3$) δ 169.60, 153.48, 151.59, 141.31, 140.80, 134.97, 131.86, 130.24, 129.58, 127.94, 126.40, 124.96, 123.92, 123.52, 122.72, 121.32, 121.19, 116.23, 113.65, 83.01, 53.37, 46.66, 36.51, 32.21, 30.98. HRMS (FD, $[M]^+$) for $C_{56}H_{58}N_2O_3$ calcd. 806.44420, found: 806.44420.

*Synthesis of 3,7-Bis((E)-4-(di-p-tolylamino)styryl)-5,5-dimethyl-5,5a,9a,10a-tetrahydrodibenzo[b,e]silin-10(4aH)-one,* **[2a]**. In a pressure tube purged with nitrogen three times, compound **2** (50 mg, 0.16 mmol), compound **3-ii** (100 mg, 0.33 mmol), $Pd(tBu_3P)_2$ (5 mg), $Cy_2NMe$ (2 mL), and dioxane (2.7 mL) were added. The reaction mixture was heated to 120 °C and stirred for 12 hours. After cooling, the mixture was extracted with DCM and saturated NaCl solution. The organic layer was dried over anhydrous $MgSO_4$, filtered, and concentrated under reduced pressure. The crude product was purified by column chromatography (hexane/DCM = 1:1) to yield the desired compound as an orange solid (22 mg,

44% yield). $^1$H NMR (600 MHz, $CDCl_3$) δ 8.42 (d, $J$ = 8.8 Hz, 2H), 7.70 – 7.71 (m, 4H), 7.40 (d, $J$ = 8.7 Hz, 4H), 7.21 (d, $J$ = 16.2 Hz, 2H), 7.09 (d, $J$ = 8.3 Hz, 8H), 7.06 – 7.00 (m, 14H), 2.33 (s, 12H), 0.56 (s, 6H). $^{13}$C NMR (101 MHz, $CDCl_3$) δ 162.84, 144.96, 138.15, 133.17, 130.08, 129.05, 127.72, 125.12, 121.91, 114.58, 46.17, 37.18, 32.83, 32.02, 29.79, 29.46, 27.18, 26.85, 22.79, 20.95, 19.82, 14.22, 8.70. HRMS (FD, $[M]^+$) for $C_{59}H_{52}N_2OSi$ calcd. 832.38434, found: 832.38434.

*Synthesis of 5,5-Dimethyl-3,7-bis((E)-2-(1,1,7,7-tetramethyl-1,2,3,5,6,7-hexahydropyrido[3,2,1-ij]quinolin-9-yl)vinyl)-5,5a,9a,10a-tetrahydrodibenzo[b,e]silin-10(4aH)-one,* **[2b]**. In a very dry pressure tube, compound **2** (50 mg, 0.16 mmol), compound **5-ii** (200 mg, 0.78 mmol), $Pd(tBu_3P)_2$ (5 mg), $Cy_2NMe$ (2 mL), and dioxane (2.7 mL) were added. The reaction mixture was heated to 120 °C and stirred for 12 h. After cooling, the mixture was extracted with DCM and saturated NaCl solution. The organic layer was dried over anhydrous $MgSO_4$, filtered, and concentrated under reduced pressure. The crude product was purified by column chromatography (hexane/DCM = 1:2) to afford the desired compound as a red-orange solid (30 mg, 60% yield). $^1$H NMR (600 MHz, $CDCl_3$) δ 8.40 (d, $J$ = 8.8 Hz, 2H), 7.69 – 7.70 (m, 4H), 7.27 (s, 4H), 7.20 (d, $J$ = 16.1 Hz, 2H), 6.93 (d, $J$ = 16.1 Hz, 2H), 3.25 – 3.20 (m, 8H), 1.80 – 1.77 (m, 8H), 1.34 (s, 24H), 0.56 (s, 6H). $^{13}$C NMR (101 MHz, $CDCl_3$) δ 186.72, 141.47, 140.97, 139.33, 138.86, 132.53, 130.76, 130.27, 130.03, 126.77, 123.70, 122.91, 121.70, 46.71, 36.52, 32.25, 30.99, 29.66. HRMS (FD, $[M]^+$) for $C_{51}H_{60}N_2OSi$ calcd. 744.44694, found: 744.44694.

**Synthesis of Asymmetric Xanthenes:**

*Synthesis of (E)-3'-(4-(di-p-tolylamino)styryl)-6'-iodo-3H-spiro[isobenzofuran-1,9'-xanthen]-3-one,* **[1d]**. Under a nitrogen atmosphere, Compound **1** (200 mg, 0.36 mmol), $Pd(OAc)_2$ (4 mg), and triethanolamine (TEOA, 2.0 mL) were added to a round-bottom flask, followed by dry DMF (30 mL). Compound **3-ii** (50 mg, 0.16 mmol) was dissolved in an additional 5 mL of dry DMF and placed in an additional funnel connected to the flask. The reaction mixture was refluxed at 130 °C for 10 minutes, after which the funnel was opened to allow the dropwise addition of compound **3-ii**. The reaction was then stirred at reflux for an additional 12 hours. Upon completion, the mixture was extracted with DCM and brine. The organic layer was separated, dried over anhydrous $MgSO_4$, filtered, and concentrated under reduced pressure. The crude product was purified by silica gel column chromatography using hexane/DCM (1:1) as the eluent, affording a yellow dye (48 mg, 41% yield). $^1$H NMR (400 MHz, $CDCl_3$) δ 8.05 (d, $J$ = 7.8 Hz, 1H), 7.73 – 7.60 (m, 2H), 7.50 (d, $J$ = 1.8 Hz, 1H), 7.41 – 7.32 (m, 3H), 7.15 – 7.18 (m, 3H), 7.08 (d, $J$ = 7.9 Hz, 5H), 7.04 – 6.90 (m, 7H), 6.77 (d, $J$ = 8.3 Hz, 1H), 6.70 (d, $J$ = 8.4 Hz, 1H), 2.32 (s, 6H). $^{13}$C NMR (101 MHz, $CDCl_3$) δ 168.96, 152.80, 150.62, 148.06, 144.54, 140.54, 134.93, 132.69, 130.43, 129.63, 128.96, 127.71, 127.22, 125.98, 124.63, 124.14, 123.44, 121.56, 118.50, 116.48, 113.87, 95.05, 81.59, 20.51. HRMS (FD, $[M]^+$) for $C_{42}H_{30}INO_3$ calcd. 723.12649, found: 723.12608.

*Synthesis of (E)-3'-iodo-6'-(2-(2,3,6,7-tetrahydro-1H,5H-pyrido[3,2,1-ij]quinolin-9-yl)vinyl)-3H-spiro[isobenzofuran-1,9'-xanthen]-3-one,* **[1e]**. In a three-neck flask under nitrogen, compound **1** (200 mg, 0.36 mmol), triethanolamine (2.0 mL), and $Pd(OAc)_2$ (4 mg, 0.018 mmol)

were combined with dry DMF (30 mL). Compound **4-ii** (50 mg, 0.25 mmol), dissolved in an additional 5 mL of dry DMF, was placed in a dropping funnel. The reaction mixture was heated to reflux at 130 °C for 10 minutes before the dropwise addition of compound **4-ii**. Reflux was continued for 12 hours. After completion, the reaction mixture was extracted with DCM and brine. The organic layer was separated, dried over anhydrous $MgSO_4$, filtered, and concentrated under reduced pressure. The crude product was purified by silica gel column chromatography using hexane/DCM (1:1) as the eluent. The product was obtained as a light yellow solid (65mg, 41% yield). $^1H$ NMR (400 MHz, $CDCl_3$) δ 8.04 (d, *J* = 7.3 Hz, 1H), 7.71 – 7.58 (m, 2H), 7.49 (s, 1H), 7.32 (s, 1H), 7.19 – 7.10 (m, 3H), 7.00 (d, *J* = 17.5 Hz, 3H), 6.80 (d, *J* = 16.1 Hz, 1H), 6.76 – 6.66 (m, 2H), 3.18 (t, *J* = 5.7 Hz, 4H), 2.77 (t, *J* = 6.5 Hz, 4H), 1.97 (p, *J* = 6.1 Hz, 4H). $^{13}C$ NMR (101 MHz, $CDCl_3$) δ 169.55, 153.57, 151.39, 151.32, 143.14, 141.38, 135.05, 131.38, 130.63, 130.54, 129.69, 127.97, 127.95, 126.27, 125.71, 125.08, 123.94, 123.89, 123.68, 123.54, 121.62, 121.41, 121.32, 118.93, 117.16, 116.24, 113.65, 82.65, 77.32, 77.00, 76.68, 49.94, 27.71, 21.91. HRMS (FD, $[M]^+$) for $C_{34}H_{26}BrNO_3$ calcd. 575.11015, found: 575.11064.

*Synthesis of 3'-((E)-4-(di-p-tolylamino)styryl)-6'-((E)-2-(2,3,6,7-tetrahydro-1H,5H-pyrido[3,2,1-ij]quinolin-9-yl)vinyl)-3H-spiro[isobenzofuran-1,9'-xanthen]-3-one,* **[1f]**. In a two-neck flask under a nitrogen atmosphere, compound **1d** (300 mg, 0.41 mmol) and compound **4-ii** (200 mg, 1 mmol) were added, along with Triethanolamine (2 mL) and Pd(OAc)2 (4 mg, 0.018 mmol). After that, dry DMF (10 mL) was added, and the mixture was refluxed at 130°C for 12 hours. After completion of the reaction, the mixture was extracted with DCM and brine solution. The organic layer was collected and dried over anhydrous $MgSO_4$ to remove water. After filtration, the solvent was removed under reduced pressure. The crude product was purified by silica gel column chromatography using hexane/DCM (1:2) as the eluent, yielding a yellow product (120 mg, 36% yield). $^1H$ NMR (400 MHz, $CDCl_3$) δ 8.04 (d, *J* = 5.6 Hz, 1H), 7.68 – 7.60 (m, 2H), 7.39 – 7.32 (m, 4H), 7.22 – 7.15 (m, 2H), 7.12 (d, *J* = 7.7 Hz, 2H), 7.08 (d, *J* = 8.0 Hz, 4H), 7.03 – 6.98 (m, 9H), 6.94 (d, *J* = 15.9 Hz, 1H), 6.81 (d, *J* = 16.2 Hz, 1H), 6.73 – 6.79 (m, 2H), 3.23 – 3.14 (m, 4H), 2.77 (t, *J* = 6.4 Hz, 4H), 2.32 (s, 6H), 1.98 (dt, *J* = 12.0, 6.4 Hz, 4H). $^{13}C$ NMR (101 MHz, $CDCl_3$) δ 169.59, 153.52, 151.62, 151.54, 148.28, 144.92, 143.14, 141.36, 140.52, 135.03, 132.94, 131.36, 130.43, 129.94, 129.73, 129.68, 128.15, 128.01, 127.51, 126.39, 125.72, 125.08, 124.91, 124.83, 123.99, 123.92, 122.00, 121.68, 121.53, 121.33, 117.23, 116.28, 114.25, 113.70, 82.81, 49.96, 29.70, 27.72, 21.92, 20.83. HRMS (FD, $[M]^+$) for $C_{56}H_{46}N_2O_3$ calcd. 794.35139, found: 794.35085.

*Synthesis of 3'-((E)-4-(di-p-tolylamino)styryl)-6'-((E)-2-(1,1,7,7-tetramethyl-2,3,6,7-tetrahydro-1H,5H-pyrido[3,2,1-ij]quinolin-9-yl)vinyl)-3H-spiro[isobenzofuran-1,9'-xanthen]-3-one,* **[1g].** In a two-neck flask under a nitrogen atmosphere, compound **1d** (200 mg, 0.27 mmol), compound **5-ii** (200 mg, 0.78 mmol), triethanolamine (2.0 mL), and $Pd(OAc)_2$ (4 mg, 0.018 mmol) were added. Anhydrous DMF (10 mL) was added to the mixture and refluxed at 130 °C for 12 hours. After completion, the mixture was cooled to room temperature and extracted with DCM and brine. The organic phase was separated, dried over anhydrous $MgSO_4$, filtered, and concentrated under reduced pressure. The crude product was purified by silica gel column chromatography using hexane/DCM (1:2) as the eluent, affording the desired compound as a yellow solid (144 mg, 60% yield). $^1H$ NMR (400 MHz, $CDCl_3$) δ 8.05 (d, *J* =

7.4 Hz, 1H), 7.61 – 7.69 (m, 2H), 7.40 – 7.33 (m, 4H), 7.26 (s, 1H), 7.23 (s, 2H), 7.20 (d, *J* = 7.7 Hz, 1H), 7.15 (d, *J* = 8.4 Hz, 2H), 7.10 – 6.96 (m, 13H), 6.87 – 6.73 (m, 4H), 3.22 (t, *J* = 6.0 Hz, 4H), 2.32 (s, 6H), 1.80 (s, 4H), 1.33 (s, 12H). $^{13}$C NMR (101 MHz, $CDCl_3$) δ 169.62, 153.51, 151.62, 151.56, 148.27, 144.92, 141.36, 140.51, 135.06, 132.94, 131.87, 130.42, 129.94, 129.70, 128.14, 127.99, 127.51, 126.38, 125.10, 124.90, 124.82, 123.93, 122.90, 121.99, 121.49, 117.22, 114.28, 113.73, 82.84, 77.32, 77.00, 76.68, 36.46, 32.32, 31.57, 31.07, 25.26, 22.64, 20.83, 14.11. HRMS (FD, $[M]^+$) for $C_{60}H_{54}N_2O_3$ calcd. 850.41399, found: 850.41480.

*Synthesis of 3'-((E)-2-(2,3,6,7-tetrahydro-1H,5H-pyrido[3,2,1-ij]quinolin-9-yl)vinyl)-6'-((E)-2-(1,1,7,7-tetramethyl-2,3,6,7-tetrahydro-1H,5H-pyrido[3,2,1-ij]quinolin-9-yl)vinyl)-3H-spiro[isobenzofuran-1,9'-xanthen]-3-one,* **[1h]**. In a two-neck flask under a nitrogen atmosphere, compound **1e** (200 mg, 0.32 mmol), compound **5-ii** (300 mg, 1.17 mmol), triethanolamine (2.0 mL), and $Pd(OAc)_2$ (4 mg, 0.018 mmol) were added. Anhydrous DMF (10 mL) was introduced, and the reaction mixture was heated to reflux at 130 °C for 12 hours. Upon completion, the mixture was cooled to room temperature and extracted with DCM and brine solution. The organic phase was separated, dried over anhydrous $MgSO_4$, filtered, and concentrated under reduced pressure. The crude product was purified by silica gel column chromatography using hexane/DCM (1:2) as the eluent. The product was obtained as a yellow solid (110 mg, 45% yield). $^1$H NMR (400 MHz, $CDCl_3$) δ 8.04 (d, *J* = 6.7 Hz, 1H), 7.70 – 7.59 (m, 2H), 7.36 (d, *J* = 16.8 Hz, 2H), 7.25 – 6.97 (m, 10H), 6.84 (d, *J* = 16.3 Hz, 2H), 6.74 (d, *J* = 8.2 Hz, 2H), 3.18 – 3.23 (m, 8H), 2.79 (t, *J* = 6.5 Hz, 4H), 2.02 (s, 4H), 1.79 (s, 4H), 1.33 (s, 12H). $^{13}$C NMR (101 MHz, $CDCl_3$) δ 169.75, 153.57, 151.64, 143.11, 141.40, 140.86, 140.24, 135.01, 131.94, 131.27, 130.35, 129.62, 128.00, 127.16, 126.46, 125.72, 124.61, 122.80, 121.78, 121.38, 119.88, 116.37, 113.75, 112.12, 83.12, 71.86, 56.75, 49.96, 46.75, 36.61, 32.28, 31.04, 29.68, 27.70, 21.92, 14.10. HRMS (FD, $[M]^+$) for $C_{52}H_{50}N_2O_3$ calcd. 750.38269, found: 750.39007.

*Synthesis of (E)-3-chloro-7-(4-(di-p-tolylamino)styryl)-5,5-dimethyldibenzo[b,e]silin-10(5H)-one,* **[2c]**. In a pressure tube, compound **2** (100 mg, 0.32 mmol), $Pd(tBu_3P)_2$ (10 mg), $Cy_2NMe$ (2 mL), and dioxane (10 mL) were added. The mixture was subjected to a vacuum for 15 minutes, followed by three purges with nitrogen. In a separate round-bottom flask, compound **3-ii** (30 mg, 0.10 mmol) was dissolved in 10 mL of dioxane. This solution was added dropwise to the pressure tube over 10 minutes. The reaction mixture was then heated to 120 °C and stirred at that temperature for 5 hours. After completion, the reaction was allowed to cool to room temperature. The mixture was extracted with DCM and brine. The organic layer was separated, dried over anhydrous $MgSO_4$, and concentrated under reduced pressure. The crude product was purified by column chromatography using hexane/DCM (1:2) as the eluent to afford the pure product as a light orange solid (30 mg, 52% yield). $^1$H NMR (400 MHz, $CDCl_3$) δ 8.40 (d, *J* = 6.4 Hz, 1H), 8.38 (d, *J* = 6.7 Hz, 1H), 7.75 – 7.66 (m, 2H), 7.62 (d, *J* = 2.2 Hz, 1H), 7.53 (dd, *J* = 8.6, 2.2 Hz, 1H), 7.39 (d, *J* = 8.7 Hz, 2H), 7.21 (d, *J* = 16.3 Hz, 1H), 7.13 – 6.97 (m, 11H), 2.33 (s, 6H), 0.54 (s, 6H). $^{13}$C NMR (101 MHz, $CDCl_3$) δ 186.05, 148.21, 144.51, 140.94, 140.83, 138.61, 132.82, 132.44, 131.21, 131.10, 130.89, 129.98, 129.67, 129.20, 127.34,

127.07, 124.75, 124.54, 121.40, 53.10, 20.53. HRMS (FD, $[M]^+$) for $C_{37}H_{32}ClNOSi$ calcd. 569.19362, found: 569.19380.

*Synthesis of 3-((E)-4-(di-p-tolylamino)styryl)-5,5-dimethyl-7-((E)-2-(1,1,7,7-tetramethyl-2,3,6,7-tetrahydro-1H,5H-pyrido[3,2,1-ij]quinolin-9-yl)vinyl)dibenzo[b,e]silin-10(5H)-one,* **[2d]**. In a pressure tube, compound **2c** (50 mg, 0.08 mmol), compound **5-ii** (100 mg, 0.39 mmol), $Pd(tBu_3P)_2$ (10 mg), $Cy_2NMe$ (2 mL) were added. The mixture was subjected to a vacuum for 15 minutes, followed by three purges with nitrogen. 4 ml of dioxane was added to the mixture and heated at 120 °C for 12 hours. After completion, the reaction was allowed to cool to room temperature. The mixture was extracted with DCM and brine. The organic layer was separated, dried over anhydrous $MgSO_4$, and concentrated under reduced pressure. The crude product was purified by column chromatography using hexane/DCM (1:2) as the eluent to afford the pure product as a light orange solid (40 mg, 63% yield). $^1H$ NMR (400 MHz, $CDCl_3$) δ 8.39 – 8.43 (m, 2H), 7.72 – 7.68 (m, 4H), 7.40 (d, $J$ = 8.7 Hz, 2H), 7.27 (s, 2H), 7.18 (s, 2H), 7.11 – 7.01 (m, 11H), 7.00 – 6.90 (m, 2H), 3.25 – 3.20 (m, 4H), 2.33 (s, 6H), 1.81 – 1.74 (m, 4H), 1.34 (s, 12H), 0.56 (s, 6H). $^{13}C$ NMR (101 MHz, $CDCl_3$) δ 186.77, 161.69, 148.37, 144.89, 140.61, 139.28, 133.04, 130.79, 129.96, 127.60, 127.17, 125.00, 123.68, 122.95, 121.85, 121.66, 46.75, 36.53, 32.29, 31.00, 29.69, 20.84. HRMS (FD, $[M]^+$) for $C_{55}H_{56}N_2OSi$ calcd. 788.41564, found: 788.41613.

**Synthesis of symmetric and asymmetric xanthene dyes in the NIR-II window:**

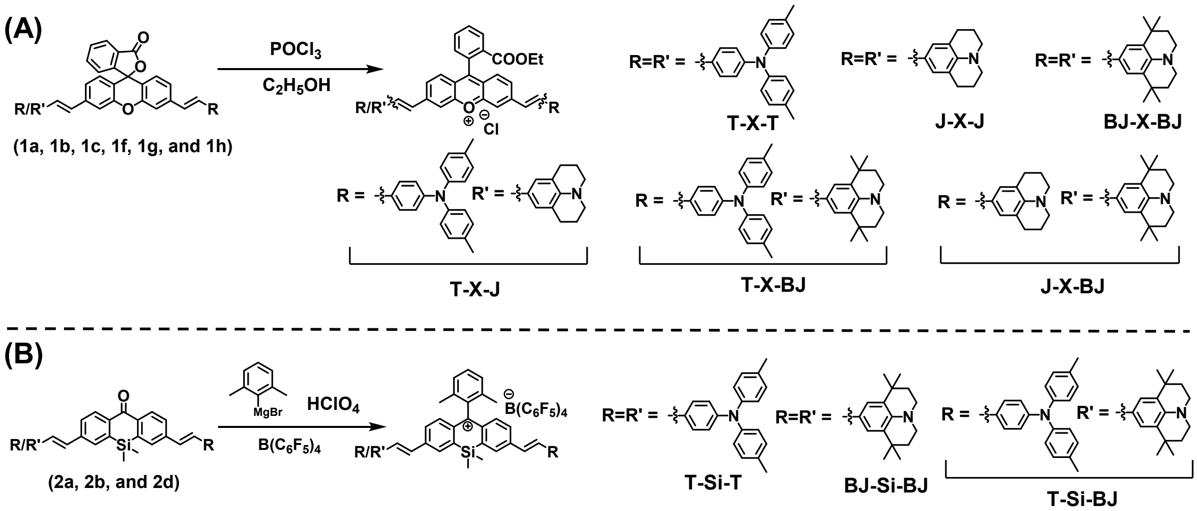


**Scheme S3:** Synthetic routes of heteroatom-substituted symmetric and asymmetric NIR-II xanthenes. (A) Ring opening reaction of lactones using ethanol to make NIR-II xanthenes. (B) Grignard followed by a counterion strategy to synthesize silocon-substituted NIR-IIb xanthenes.

General strategy to synthesize the ring-opening product of symmetric and asymmetric lactones (**T-X-T**, **J-X-J**, **BJ-X-BJ**, **T-X-J**, **T-X-BJ**, and **J-X-BJ**):

In a two-neck round-bottom flask, 0.5 mmol of lactones (**1a**, **1b**, **1c**, **1f**, **1g**, and **1h**) were added. The flask was subjected to vacuum for 30 minutes, followed by purging with nitrogen for 15 minutes. A solution of phosphorus oxychloride ($POCl_3$, 1 mL) in 1,2-dichloroethane (10 mL) was then added, and the reaction mixture was refluxed for 4 hours. After completion, the solvent was removed under reduced pressure. The resulting residue was treated with 10 mL of dry ethanol and refluxed for 24 hours. Reaction progress was monitored by TLC. Upon cooling to room temperature, ethanol was evaporated under reduced pressure. The crude mixture was extracted with DCM and brine. The combined organic layers were dried over anhydrous $MgSO_4$, filtered, and concentrated under reduced pressure. The crude product was purified by column chromatography using DCM/methanol (100:2, v/v), followed by reprecipitation from diethyl ether to afford the final product.

**T-X-T** was obtained as a green solid (80 mg, 17% yield). $^1H$ NMR (400 MHz, $CDCl_3$) δ 8.38 (d, *J* = 7.7 Hz, 1H), 8.05 (s, 2H), 7.84 – 7.79 (m, 2H), 7.71 (d, *J* = 15.7 Hz, 2H), 7.51 (d, *J* = 8.2 Hz, 2H), 7.39 (d, *J* = 8.8 Hz, 1H), 7.20 (d, *J* = 15.7 Hz, 2H), 7.13 (d, *J* = 8.0 Hz, 8H), 7.06 (d, *J* = 7.9 Hz, 10H), 7.01 (d, *J* = 7.6 Hz, 2H), 6.96 (d, *J* = 8.7 Hz, 4H), 4.04 (q, *J* = 7.0 Hz, 2H), 2.35 (s, 12H), 1.07 (t, *J* = 7.2 Hz, 3H). $^{13}C$ NMR (101 MHz, $CDCl_3$) δ 164.32, 156.75, 152.27, 150.55, 144.55, 143.42, 140.61, 134.33, 133.34, 132.71, 130.01, 129.71, 127.92, 127.46, 126.80, 125.77, 125.13, 124.76, 122.94, 122.48, 121.83, 121.16, 119.88, 114.01, 82.88, 62.38, 32.69, 30.48, 23.54, 21.82, 14.80. HRMS (FD, $[M]^+$) for $C_{66}H_{55}N_2O_3^+$ calcd. 923.42182, found: 923.42087.

**J-X-J** was obtained as a brown solid (130 mg, 36% yield). $^1H$ NMR (400 MHz, $CDCl_3$) δ 8.36 (d, *J* = 7.8 Hz, 1H), 7.89 – 7.79 (m, 2H), 7.71 (d, *J* = 1.6 Hz, 2H), 7.60 (s, 1H), 7.53 (d, *J* = 8.7 Hz, 1H), 7.39 (d, *J* = 7.4 Hz, 1H), 7.19 – 7.16 (m, 6H), 7.06 – 6.96 (m, 4H), 4.06 (q, *J* = 7.1 Hz, 2H), 3.34 (t, *J* = 5.8 Hz, 8H), 2.79 (t, *J* = 6.3 Hz, 8H), 2.01 – 1.96 (m, 8H), 1.07 (t, *J* = 7.2 Hz, 3H). $^{13}C$ NMR (101 MHz, $CDCl_3$) δ 151.17, 138.49, 129.60, 125.82, 125.56, 124.72, 122.87, 121.47, 121.44, 50.08, 29.80, 29.42, 27.80, 27.30, 22.78, 22.09, 22.01, 14.43, 14.22.

**BJ-X-BJ** was obtained as a brown solid (70 mg, 16% yield). $^1H$ NMR (400 MHz, $CDCl_3$) δ 8.37 (d, *J* = 7.9 Hz, 1H), 7.95 – 7.87 (m, 1H), 7.86 – 7.78 (m, 1H), 7.73 (s, 2H), 7.66 – 7.58 (m, 4H), 7.39 (s, 4H), 7.19 (d, *J* = 8.8 Hz, 2H), 7.03 (d, *J* = 15.5 Hz, 2H), 4.06 (q, *J* = 7.1 Hz, 2H), 3.38 (t, *J* = 6.2 Hz, 8H), 1.80 – 1.76 (m, 9H), 1.34 (s, 25H), 1.06 (t, *J* = 7.1 Hz, 4H). $^{13}C$ NMR (101 MHz, $CDCl_3$) δ 164.16, 159.19, 155.79, 151.17, 143.51, 141.33, 132.78, 130.97, 130.38, 129.83, 129.37, 128.68, 127.46, 125.84, 125.05, 122.50, 122.33, 121.75, 119.50, 113.37, 111.42, 61.90, 47.35, 47.09, 37.04, 36.00, 32.75, 32.63, 31.51, 30.56, 29.80, 27.72, 26.07, 23.23, 14.74, 14.42. HRMS (FD, $[M]^+$) for $C_{58}H_{63}N_2O_3^+$ calcd. 835.48332, found: 835.48338.

**T-X-J** was obtained as a dark green solid (117 mg, 28% yield). $^1H$ NMR (400 MHz, $CDCl_3$) δ 8.36 (d, *J* = 7.7 Hz, 1H), 7.94 – 7.66 (m, 7H), 7.65 – 7.61 (m, 1H), 7.47 (d, *J* = 8.9 Hz, 4H), 7.28 (s, 2H), 7.12 (d, *J* = 8.3 Hz, 5H), 7.05 (d, *J* = 8.4 Hz, 5H), 6.97 (d, *J* = 8.3 Hz, 4H), 4.05 (q, *J* = 7.1 Hz, 2H), 3.38 (t, *J* = 5.8 Hz, 4H), 2.79 (t, *J* = 6.5 Hz, 4H), 2.34 (s, 6H), 2.02 – 1.97 (m, 5H), 1.06 (t, *J* = 7.1 Hz, 3H). $^{13}C$ NMR (101 MHz, $CDCl_3$) δ 163.96, 152.85, 144.06, 134.20, 131.49, 130.17, 125.73, 124.87, 122.22, 120.42, 115.19, 114.07, 92.65, 61.75, 57.05, 50.57,

38.30, 27.59, 21.22, 20.92, 13.79. HRMS (FD, $[M]^+$) for $C_{58}H_{51}N_2O_3^+$ calcd. 824.39834, found: 824.40007.

**T-X-BJ** was obtained as a dark green solid (140 mg, 31% yield). $^1$H NMR (400 MHz, $CDCl_3$) δ 8.36 (d, $J$ = 9.3 Hz, 1H), 7.91 (t, $J$ = 6.9 Hz, 1H), 7.86 – 7.77 (m, 2H), 7.74 (d, $J$ = 4.0 Hz, 1H), 7.65 (dd, $J$ = 21.0, 8.9 Hz, 2H), 7.54 (d, $J$ = 16.1 Hz, 1H), 7.46 (d, $J$ = 9.3 Hz, 4H), 7.21 (d, $J$ = 8.4 Hz, 2H), 7.12 (d, $J$ = 8.2 Hz, 5H), 7.09 – 7.01 (m, 5H), 6.97 (d, $J$ = 8.8 Hz, 2H), 4.05 (q, $J$ = 7.1 Hz, 2H), 3.43 (t, $J$ = 6.3 Hz, 4H), 2.34 (s, 6H), 1.78 (t, $J$ = 6.3 Hz, 4H), 1.35 (s, 12H), 1.06 (t, $J$ = 7.1 Hz, 3H). $^{13}$C NMR (101 MHz, $CDCl_3$) δ 168.97, 153.06, 147.89, 144.56, 143.84, 143.69, 141.61, 133.96, 133.11, 132.69, 131.28, 131.15, 130.71, 129.95, 129.71, 129.46, 129.11, 127.92, 127.77, 127.31, 126.39, 125.57, 125.35, 124.90, 124.75, 123.78, 122.68, 122.05, 121.86, 121.35, 120.27, 119.75, 113.63, 62.24, 47.90, 47.66, 36.29, 36.04, 33.07, 32.94, 32.92, 31.82, 30.86, 30.63, 21.80, 21.71, 14.74. HRMS (FD, $[M]^+$) for $C_{62}H_{59}N_2O_3^+$ calcd. 879.45202, found: 879.45162.

**J-X-BJ** was obtained as a brown solid (100 mg, 25% yield). $^1$H NMR (400 MHz, $CDCl_3$) δ 8.36 (d, $J$ = 9.2 Hz, 1H), 7.94 – 7.76 (m, 2H), 7.69 (d, $J$ = 13.1 Hz, 2H), 7.57 – 7.60 (m, 4H), 7.39 (d, $J$ = 9.0 Hz, 3H), 7.20 – 7.13 (m, 4H), 7.01 (d, $J$ = 15.6 Hz, 2H), 4.06 (q, $J$ = 7.1 Hz, 2H), 3.41 – 3.35 (m, 4H), 3.32 (t, $J$ = 5.8 Hz, 4H), 2.77 (t, $J$ = 6.3 Hz, 4H), 2.01 – 1.96 (m, 4H), 1.79 – 1.75 (m, 4H), 1.33 (s, 12H), 1.06 (t, $J$ = 7.1 Hz, 3H). $^{13}$C NMR (151 MHz, $CDCl_3$) δ 165.00, 156.54, 151.64, 146.44, 144.17, 141.57, 134.97, 133.31, 131.51, 130.92, 130.33, 128.83, 128.00, 125.50, 122.78, 121.80, 113.66, 111.81, 61.72, 50.30, 49.98, 47.03, 36.61, 35.55, 32.16, 31.03, 30.07, 29.68, 21.92, 21.36, 13.77. HRMS (FD, $[M]^+$) for $C_{54}H_{55}N_2O_3^+$ calcd. 779.42072, found: 779.42106.

General procedure for synthesis of **T-Si-T**, **BJ-Si-BJ**, and **T-Si-BJ**:

In a pressure tube, 0.1 mmol of ketones (compounds **2a**, **2b**, and **2d**) were added and subjected to vacuum/$N_2$ for 10 min, followed by the addition of 3 mL dry THF. Under a nitrogen atmosphere, 0.2 mmol of 1,3-dimethylphenylmagnesium bromide was added dropwise over 10 minutes to the ketone. The reaction was stirred for 30 min at room temperature. The color of the solution was changed from orange to dark red (reaction time was controlled by TLC). After 30 minutes, the THF was evaporated under vacuum and quenched with 0.1 mL of water. The resulting mixture was extracted using DCM and water. The organic phase was dried over $MgSO_4$ and passed through a thick cotton pad using DCM. Next, 20 ml of 2M $HClO_4$ was added to that DCM mixture and extracted 5 times with water. The resulting mixture contains $ClO_4$ anion as a counterion.

To stabilize the cation and prevent it from nucleophilic decomposition in a polar environment, a counterion-pairing strategy was employed using lithium tetrakis(pentafluorophenyl)borate ethyl ether. 0.1 mmol of $ClO_4$ based dye was dissolved in 10 mL ACN, and 0.1 mmol of lithium tetrakis(pentafluorophenyl)borate ethyl ether was added to it, and the solution was stirred for 15 min, followed by extraction with water to obtain lithium tetrakis(pentafluorophenyl)borate counterion-based **T-Si-T**, **BJ-Si-BJ**, and **T-Si-BJ** as the final compounds.

**T-Si-T** was obtained as a purple solid (40 mg, 48% yield). $^1$H NMR (600 MHz, $CD_2Cl_2$) δ 7.52 (s, 4H), 7.39 (t, *J* = 7.6 Hz, 2H), 7.17 (d, *J* = 8.1 Hz, 12H), 7.08 – 7.04 (m, 12H), 6.92 (d, *J* = 8.4 Hz, 7H), 2.34 (s, 13H), 1.99 (s, 6H), 0.66 (s, 6H). $^{13}$C NMR (101 MHz, $CDCl_3$) δ 184.38, 142.30, 134.70, 129.61, 128.32, 125.49, 106.75, 77.00, 76.79, 76.58, 21.35, 20.33. $^{19}$F NMR (376 MHz, $CDCl_3$) δ -132.43, -163.16, -163.21, -163.27, -166.78. HRMS (ESI, $[M]^+$) for $C_{67}H_{61}N_2Si^+$ calcd. 921.4599, found: 921.4599.

**BJ-Si-BJ** was obtained as a dark brown solid (30 mg, 40% yield). $^1$H NMR (600 MHz, $CD_2Cl_2$) δ 7.84 (s, 2H), 7.55 (s, 2H), 7.41 (s, 4H), 7.33 (q, *J* = 10.3 Hz, 2H), 7.29 – 7.14 (m, 4H), 6.99 (s, 4H), 3.54 (t, *J* = 100.1 Hz, 8H), 2.01 (s, 6H), 1.78 (t, *J* = 6.1 Hz, 8H), 1.32 (s, 24H), 0.63 (s, 6H). $^{13}$C NMR (101 MHz, $CDCl_3$) δ 77.53, 77.32, 77.11, 48.33, 36.04, 33.00, 30.66, 30.49, 20.79, 2.10. The $^{13}$C NMR spectrum could barely be obtained because the high sample concentration required for a reasonable acquisition time led to poor spectral resolution, likely resulting from dye aggregation.[4, 6] $^{19}$F NMR (376 MHz, $CDCl_3$) δ -132.39, -162.88, -166.63. HRMS (ESI, $[M]^+$) for $C_{59}H_{69}N_2Si^+$ calcd. 833.5225, found: 833.5225.

**T-Si-BJ** was obtained as a teal green solid (25 mg, 32% yield). $^1$H NMR (600 MHz, $CD_2Cl_2$) δ 7.85 (d, *J* = 8.7 Hz, 3H), 7.56 (s, 2H), 7.39 (d, *J* = 8.8 Hz, 4H), 7.34 (d, *J* = 15.3 Hz, 2H), 7.22 (d, *J* = 7.6 Hz, 3H), 7.13 – 7.07 (m, 6H), 7.02 – 6.98 (m, 7H), 6.93 (d, *J* = 8.7 Hz, 4H), 3.66 (t, *J* = 6.1 Hz, 4H), 2.31 (s, 6H), 2.00 (s, 6H), 1.86 – 1.83 (m, 4H), 1.35 (s, 12H), 0.61 (s, 6H). $^{13}$C NMR (101 MHz, $CDCl_3$) δ 186.87, 172.31, 148.48, 144.99, 133.14, 131.31, 131.04, 130.90, 130.41, 130.19, 130.07, 127.70, 127.28, 126.95, 125.11, 123.05, 121.95, 121.76, 46.85, 36.64, 32.39, 31.11, 20.95. $^{13}$C NMR exhibits poor resolution.[4, 6] $^{19}$F NMR (376 MHz, $CDCl_3$) δ -132.76, -162.28, -166.29. HRMS (ESI, $[M]^+$) for $C_{63}H_{65}N_2Si^+$ calcd. 877.4912, found: 877.4912.

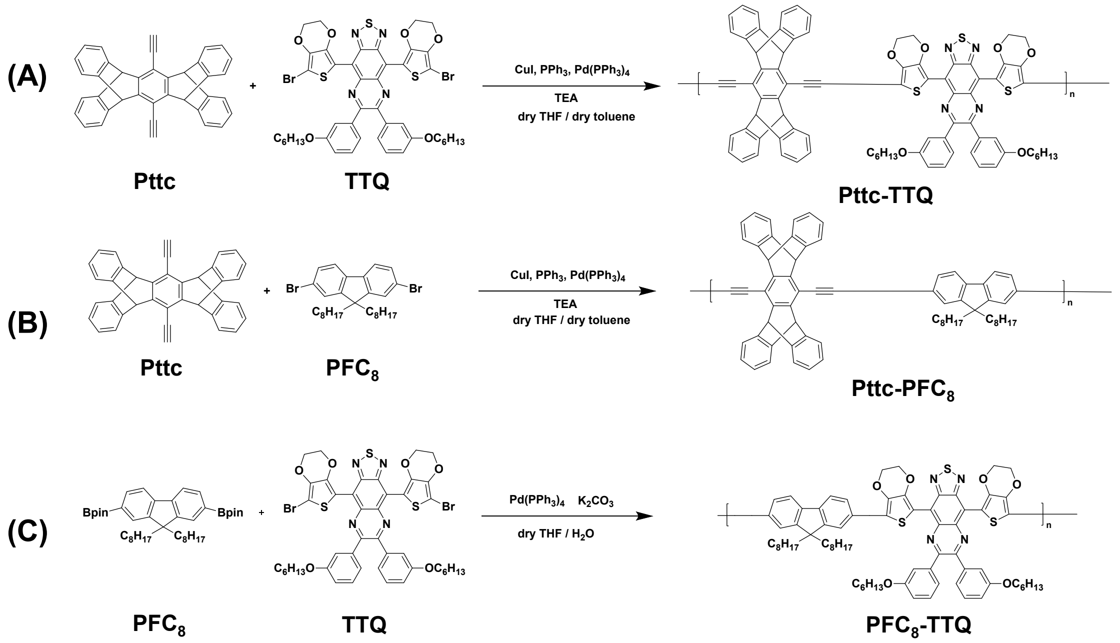


**Scheme S4**: Synthetic routes for the synthesis of **Pttc-TTQ**, **Pttc-PFC$_8$**, and **PFC$_8$-TTQ**.

*General Procedure for the Synthesis of Pttc–TTQ Polymer*

The conjugated polymers were synthesized via a Sonogashira polycondensation reaction, as illustrated in Scheme S4. In a typical procedure, Pttc (59.8 mg, 0.125 mmol), TTQ (122 mg, 0.125 mmol), CuI (2 mg), $PPh_3$ (8 mg), and $Pd(PPh_3)_2Cl_2$ (5 mg) were combined in 5 mL of dry triethylamine and tetrahydrofuran (THF). The mixture was stirred under an inert atmosphere at 65 °C for 54 h to promote cross-coupling polymerization. Subsequently, phenylacetylene (200 μL) was introduced, and the reaction continued for an additional hour, followed by the addition of bromobenzene (200 μL) to terminate the polymer chain ends. The crude polymer was purified by repeated precipitation from cold methanol/acetone and thoroughly washed with excess cold acetone/methanol. The purified material was dissolved in dichloromethane ($CH_2Cl_2$) and extracted three times with brine. After drying and solvent removal, 70–80 mg of polymer was obtained. The resulting Pttc-TTQ polymer exhibited number-average molecular weight (Mn) of 23,770, weight-average molecular weight (Mw) of 40,981, and a polydispersity index (PDI) of 1.72.

*General Procedure for the Synthesis of Pttc–$PFC_8$ Polymers*

The Pttc–PFC8 polymer was synthesized through a palladium-catalyzed coupling reaction conducted under an inert nitrogen atmosphere. In a high-pressure reaction tube, Pttc (14.4 mg, 0.03 mmol), $PFC_8Br$ (16.4 mg, 0.03 mmol), $Pd(PPh_3)_4$ (1.16 mg, 0.001 mmol), CuI (0.6 mg, 0.003 mmol), and triphenylphosphine (2.1 mg, 0.008 mmol) were charged sequentially. The tube was then subjected to three evacuation–nitrogen backfill cycles to ensure complete removal of oxygen. Separately, a solvent mixture composed of anhydrous THF (0.8 mL), toluene (0.26 mL), and triethylamine (0.5 mL) was degassed using the same three-cycle evacuation–backfill procedure, followed by continuous nitrogen purging for 30 min. This degassed solvent system was subsequently transferred into the reaction tube under a nitrogen atmosphere. The mixture was heated to 95 °C and stirred for 72 h, allowing polymerization to proceed to completion. After this period, phenylacetylene (50 μL) was added to cap the terminal alkyne groups, and the reaction was maintained for 1 h. A second end-capping step was then carried out by adding bromobenzene (50 μL) and stirring for an additional 1 h. Following completion, the reaction mixture was cooled to room temperature and poured into an ice-cold methanol/acetone mixture (50 mL:25 mL), which induced polymer precipitation. The suspension was stirred for 1 h, and the resulting solid was collected by vacuum filtration. The crude polymer was washed thoroughly with acetone to remove low-molecular-weight by-products and further purified via DCM/$H_2O$ extraction. Final purification was achieved by reprecipitation from DCM/MeOH, yielding a dark green to light green solid, corresponding to the pure Pttc-$PFC_8$ polymer.

*General Procedure for the Synthesis of $PFC_8$-TTQ Polymer*

The $PFC_8$-TTQ polymer was synthesized via a palladium-catalyzed polycondensation reaction under an inert nitrogen atmosphere. In a high-pressure reaction tube, compound $PFC_8$-Bpin (11.6 mg, 0.018 mmol), compound TTQ (29.4 mg, 0.03 mmol), $K_2CO_3$ (20.7 mg, 0.15 mmol)

and $Pd(PPh_3)_4$ (1.16 mg, 0.001 mmol) were added sequentially. The reaction tube was subjected to three consecutive evacuation–nitrogen backfill cycles to ensure complete removal of oxygen. Subsequently, anhydrous THF (0.5 mL) and $H_2O$ (0.15 mL) were introduced into the tube under nitrogen. The reaction mixture was then heated to 120 °C and stirred for 72 h, allowing the polymerization to proceed. After cooling down, the mixture was extracted with dichloromethane. The organic phase was dried over anhydrous $Na_2SO_4$ and filtered. After removing the solvent from the filtrate, the crude polymer was precipitated from dichloromethane solution in methanol to give polymer PFC$_8$-TTQ as a yellow solid (25 mg). The solid product was collected by vacuum filtration, thoroughly washed with acetone to remove residual low-molecular-weight species.

*Sample preparation for 2D NMR Measurement*

Equimolar amounts of **T-X-T** and **Pttc-TTQ** were mixed in a minimal amount of $CH_2Cl_2$ and reprecipitated in $CH_2Cl_2$/ether overnight to remove any further impurities. The resulting solid was then dissolved in $CDCl_3$ to make a homogeneous solution suitable for NMR experiment. The solution was mixed well to ensure complete dissolution and uniform mixing before the sample was set for solution-state ROESY measurement.

*Preparation of Pdots*

The fabrication of Pdots was carried out by thoroughly blending 900 μL of a semiconducting polymer solution containing Pttc-TTQ (0.4 mg), 4.0 mg of mPEG-DSPE-2000, and 0.7 mg of symmetric and asymmetric xanthene dyes dissolved in 10 mL of DCM. The resulting polymer solution in DCM was removed under reduced pressure at room temperature, followed by 2 hours under vacuum to make a fine film. The film was then dissolved in 4 mL PBS solution and sonicated for 40 minutes. The obtained Pdots dispersion was subsequently filtered through a 0.2 μm cellulose acetate syringe filter to yield a clear and uniform suspension ready for use. The freshly prepared Pdots remained optically stable for at least two weeks when stored in the dark at room temperature.

*Characterization of Pdots*

The average particle size of the Pdots was analyzed using dynamic light scattering (DLS) and transmission electron microscopy (TEM). TEM imaging was performed with a JEOL 2100 transmission electron microscope operated at 200 kV. For TEM sample preparation, a drop of the aqueous Pdots solution was deposited onto a carbon-coated copper grid and allowed to dry naturally at room temperature.

The optical absorption spectra of the Pdots were recorded using UV–Vis spectrophotometry (Dynamica Halo DB20S, Dynamica Scientific) in the range of 600–1100 nm and a BWTek Sol 1.7 spectrometer (Sensors Unlimited, Inc.) for the 900–1700 nm region. Fluorescence spectra

were acquired with a FS5 spectrofluorometer (Edinburgh Instruments Ltd., UK) using a 980 nm excitation laser.

*Determination of Fluorescence Quantum Yields*

The fluorescence quantum yields (QY) of the polymers and monomers were determined relative to the IR-1061 dye (QY = 0.59%, reported as absolute QY)[7] as a standard. The QY was calculated using the following equation:

$$QY_s = QY_r \times (\frac{K_s}{K_r}) \times (\frac{n_s}{n_r})^2$$

where subscripts s and r denote the sample and reference, respectively, K represents the slope of the integrated fluorescence intensity versus absorbance (obtained by linear fitting over at least five data points), and n is the refractive index of the solvent. To minimize self-quenching and reabsorption or re-emission effects, the maximum absorbance of each sample was maintained below 0.1 during measurements. The optical parameters used were as follows: Oxygen-xanthene derivatives were compared with IR-1061 under 808 nm excitation, with emission collected from 900 to 1600 nm, and silicon-xanthene derivatives were compared with IR-1061 under 1064 nm excitation, with emission collected from 1200 to 2000 nm.

*MTT Assay*

The cytotoxicity of the Pdots was assessed on SKOV-3 cells using the MTT assay, which measures cell viability via 3-(4,5-dimethylthiazol-2-yl)-2,5-diphenyltetrazolium bromide reduction. HeLa cells were seeded into 24-well culture plates and exposed to varying Pdots concentrations (5, 10, and 20 μg/mL) for incubation periods of 6, 12, and 24 hours. After treatment, 20 μL of MTT solution (5 mg/mL) was added to each well, followed by incubation for an additional 4 hours at 37°C to allow for MTT reduction. The medium was then carefully removed, and 300 μL of DMSO was introduced to dissolve the resulting formazan crystals. Absorbance was measured at 570 nm using a BioTek ELx800 microplate reader. Control wells containing only culture medium (without Pdots) were used as the reference for cell viability determination.

*In Vivo Fluorescence Imaging of Mouse Blood Vasculature Using Pdots*

All animal procedures were carried out according to the guidelines and protocols approved by the Institutional Animal Care and Use Committee (IACUC #1100509) at NYCU. Five-week-old female nude mice (BALB/cAnN.Cg-Foxn1nu/CrlNarl) were obtained from the National Laboratory Animal Center and housed in a specific pathogen-free facility maintained at 24 °C under a 12-hour light/dark cycle. Before imaging, groups of five mice (n=5) were anesthetized using 2% isoflurane administered through a rodent ventilator system. Each animal received an intravenous injection of 100–200 μL of Pdots solution (5.5 mg/mL) via the tail vein.

Fluorescence imaging in vivo was performed using a custom-built near-infrared II (NIR-II) imaging system equipped with a 1064 nm excitation laser and an InGaAs camera (Ninox 640 SU, Raptor Photonics). Emission signals were collected through a 1200–1400 nm long-pass filter (Thorlabs). The imaging setup maintained the camera at −80 °C, with a 10 MHz analog-to-digital conversion rate, high gain, and exposure times between 500 and 1500 ms. During image acquisition, the laser power density was controlled between 20 and 100 mW $cm^{-2}$.

*Inference for Diffusion-Model-based HDR (DMHDR) Rendering.*

All experiments were executed on a workstation equipped with an Intel Core i9-14900K (24 threads, 6 P-cores @ 6.0 GHz), an NVIDIA RTX 5090 GPU with 32 GB GDDR7, and 96 GB of DDR5-5600 system memory. CUDA 12.8 and PyTorch 2.7.1 were compiled with cuDNN and TensorRT-LLM acceleration. Each NIR-II fluorescence frame (512 × 512 px) was first normalized to floating-point radiance; a small Gaussian window produced a mean-subtracted contrast-normalized edge map, while a broader Gaussian blur supplied a luma prior. These two conditioning images were fed, without any parameter tuning, into a pre-trained structure-preserving diffusion model; inference employed a deterministic 20-step DDIM sampler, and from mid-sampling onward the original edge map was re-injected at every iteration to prevent structural drift. To remain within 22 GB of GPU memory, each full frame was processed as overlapping 512 × 512 tiles that were sequentially generated (~32 ms per tile) and seamlessly blended. The resulting luminance was rescaled back to the original dynamic range, and final outputs were stored as PNG files for quantitative analysis alongside sRGB previews for publication, with no additional tone-mapping or sharpening applied so that all visual improvements derive solely from the diffusion process.

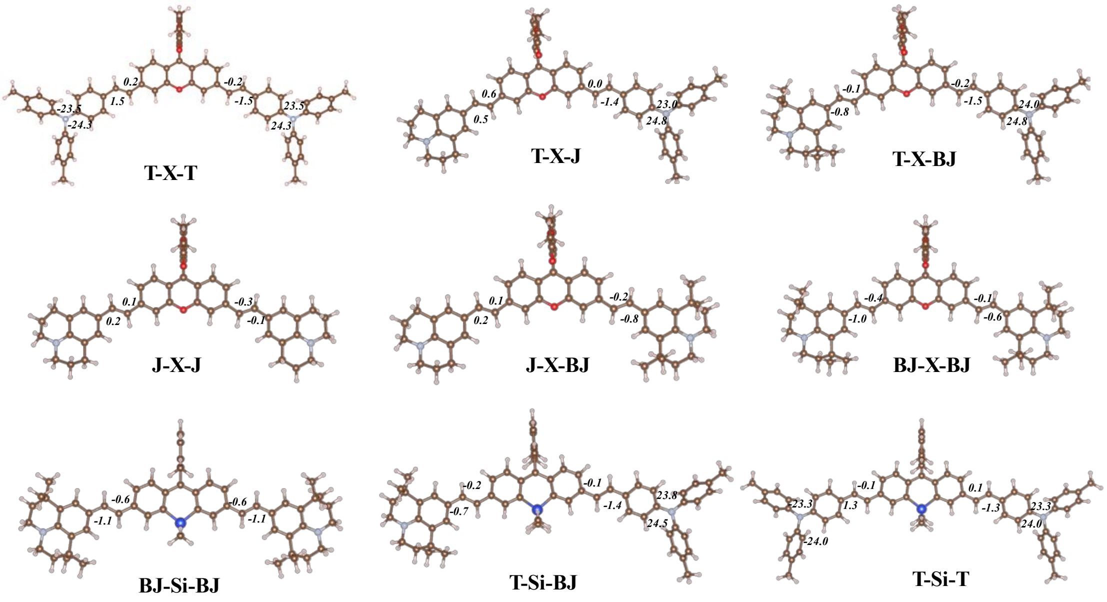


**Figure S1.** The optimized structure of all Xanthenes at the theoretical level of B3LYP-D3/6-31G(d). The values marked are important dihedral parameters. Color in brown, light blue, red, dark blue and white are carbon, nitrogen, oxygen, silicon and hydrogen, respectively.

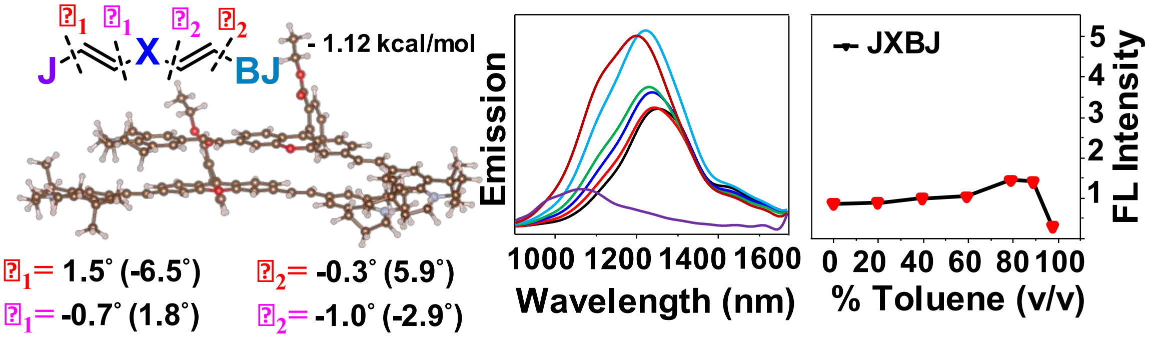


**Figure S2.** Theoretical and experimental calculations to evaluate the aggregation behaviors of the studied dyes. The first column depicts the dihedral angles of the optimized geometry at ground state ($S_0$) of J-X-BJ (The dihedral angle of the linker (C=C) relative to chromophore (□1 and □2) or molecular core (□1 and □2)) and shows the optimized structures, Zero-point energy (ZPE) corrections, and BSSE-corrected binding energy of low-energy Xanthene dimers at the M06-2X/6-31+G*//B3LYP-D3/6-31G* level. Color in brown, light blue, red, and white are carbon, nitrogen, oxygen, and hydrogen, respectively. The second column represents emission profiles as the fraction of toluene increases in DMSO. Color in black, red, blue, green, sky blue, brown, and purple represent 0%, 20%, 40%, 60%, 80%, 90%, and 99% toluene fraction (v/v), respectively, in DMSO. The Third column describes FL intensity variation with % toluene.

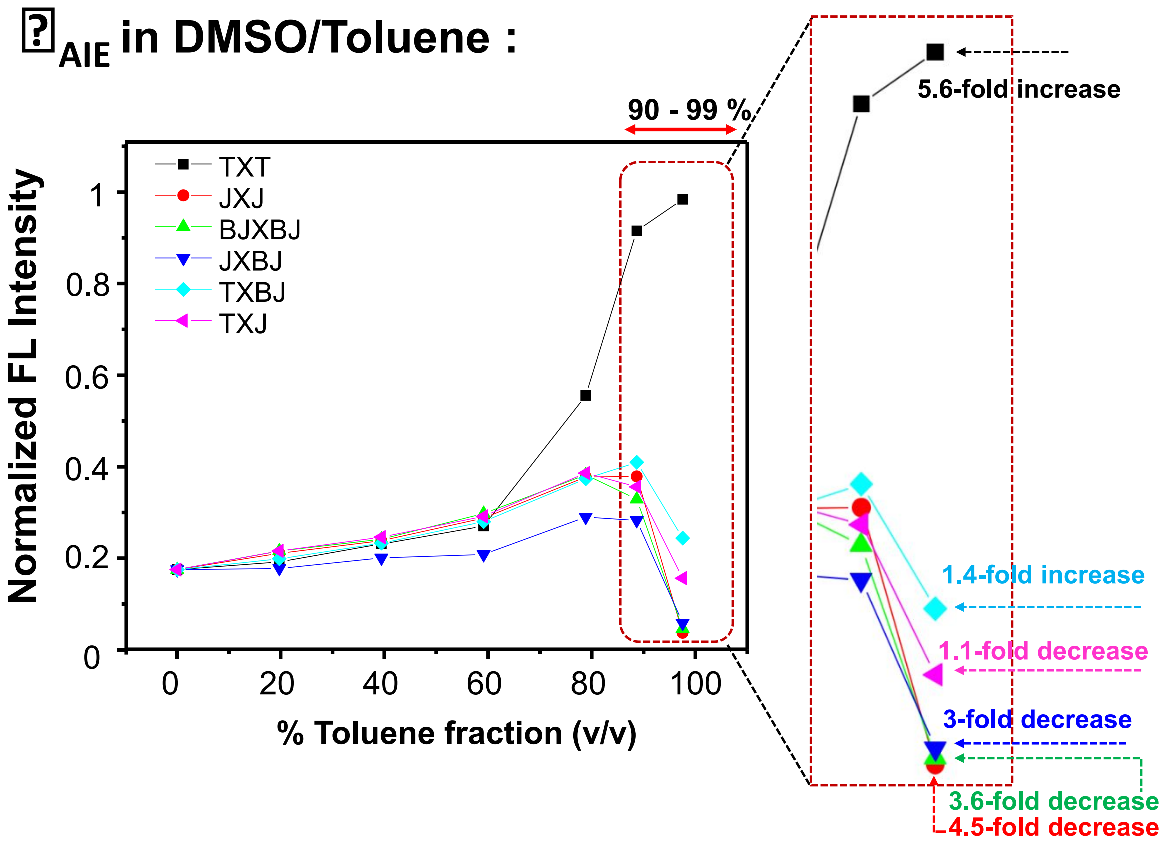


**Figure S3.** Aggregation behavior of oxygen-substituted xanthenes in DMSO at different toluene fractions, illustrated by the change in fluorescence intensity with increasing toluene content.

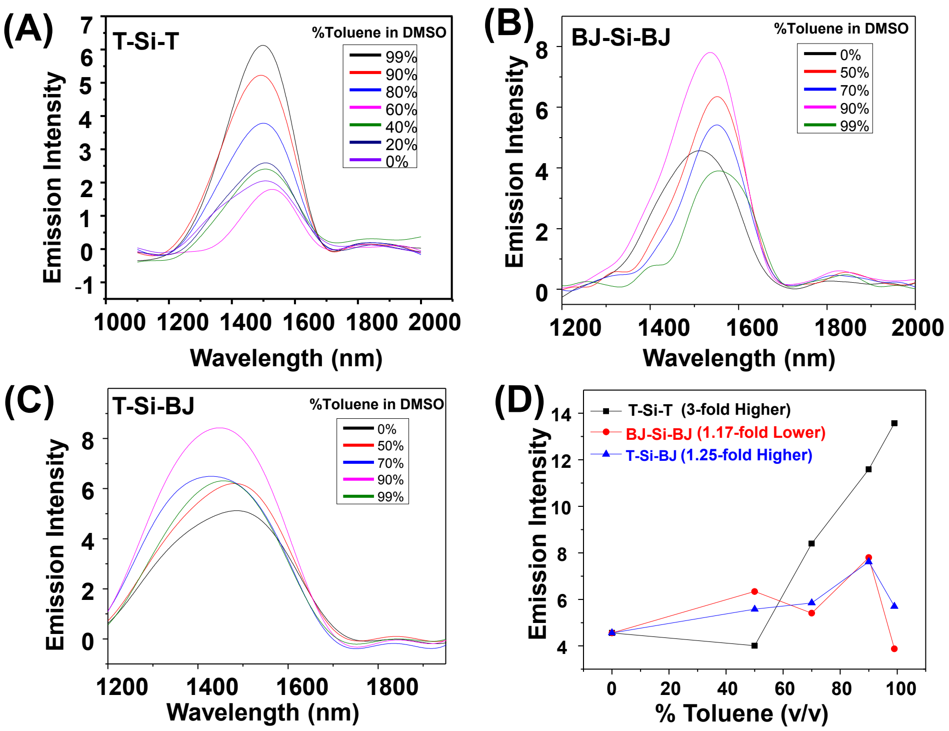


**Figure S4.** Illustration of the aggregation behavior of silicon-substituted xanthenes. (A-C) Emission profiles at different toluene fractions in DMSO, while panel (D) compares the fluorescence intensity variations of **T-Si-T**, **BJ-Si-BJ**, and **T-Si-BJ**.

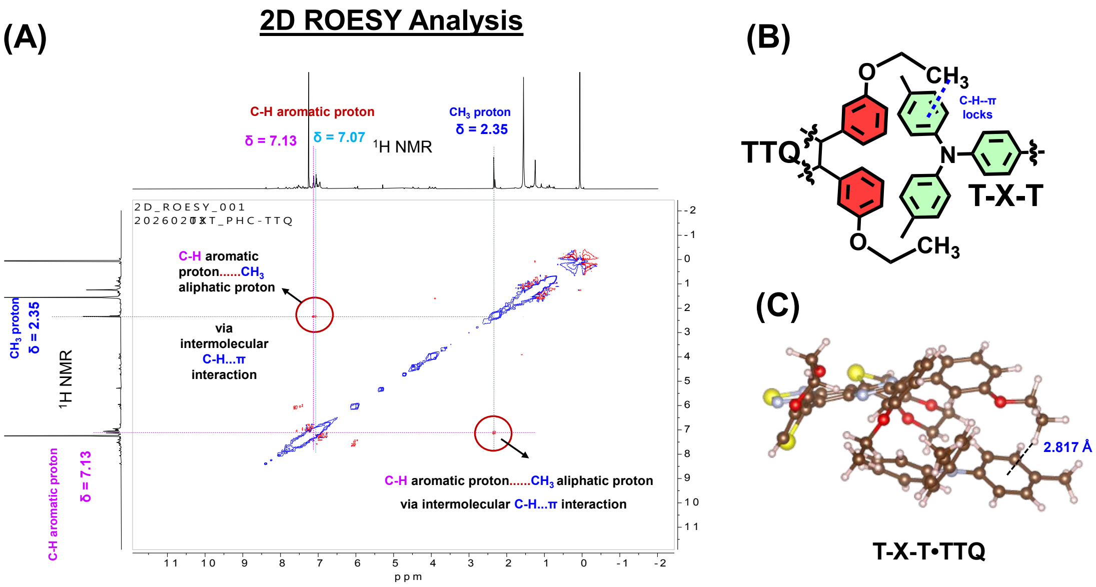


**Figure S5.** (A) 2D ROESY ¹H NMR of **T-X-T+Pttc-TTQ** co-precipitated dye, which defines through-space C-H...π intermolecular noncovalent conformation locks. (B) Schematic interaction between aliphatic $CH_3$ and aromatic C-H proton via C-H...π locks. (C) Proposed model showcasing partial structures of low-energy **T-X-T•TTQ** complexes at the M06 2X/6-31G*(0 K)/6-31G* level.

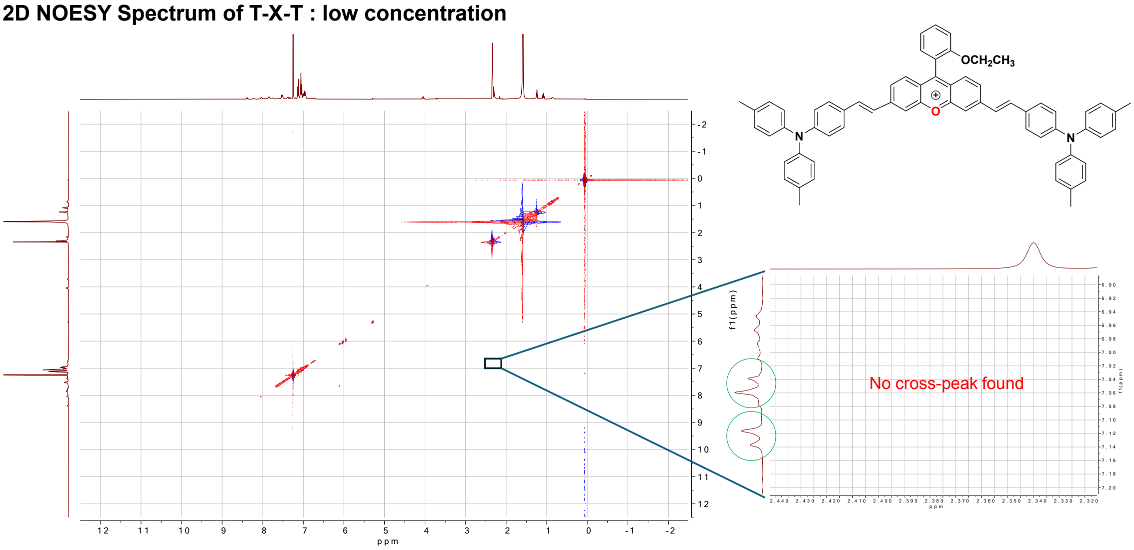


**Figure S6.** 2D NOESY ¹H NMR of **T-X-T** control dye, which defines the absence of through-space C-H...π intermolecular noncovalent conformation locks.

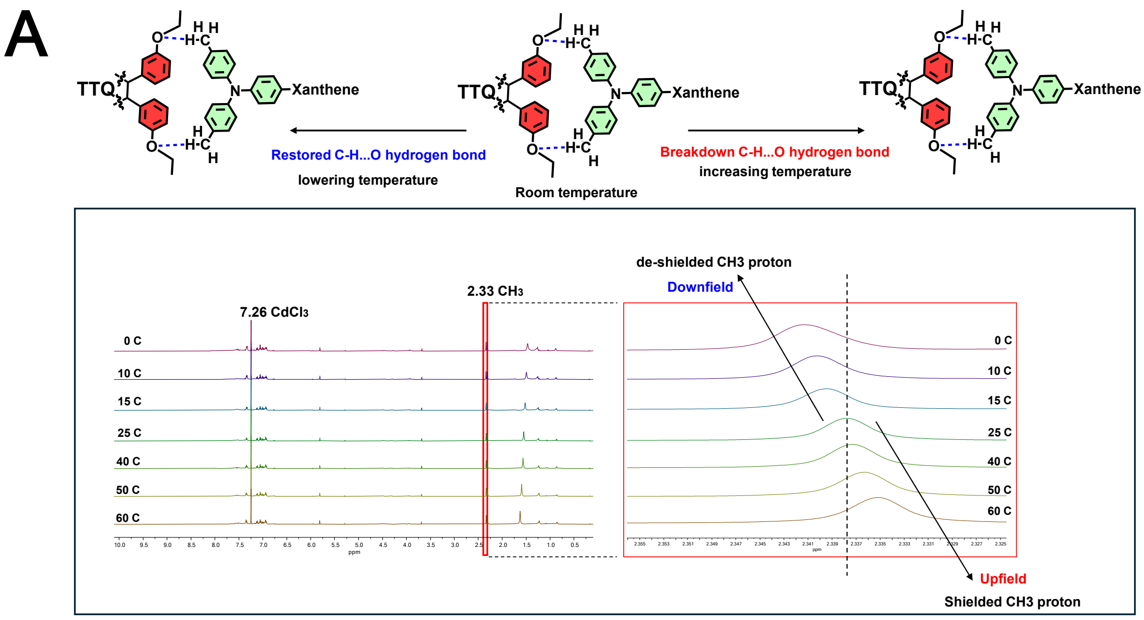


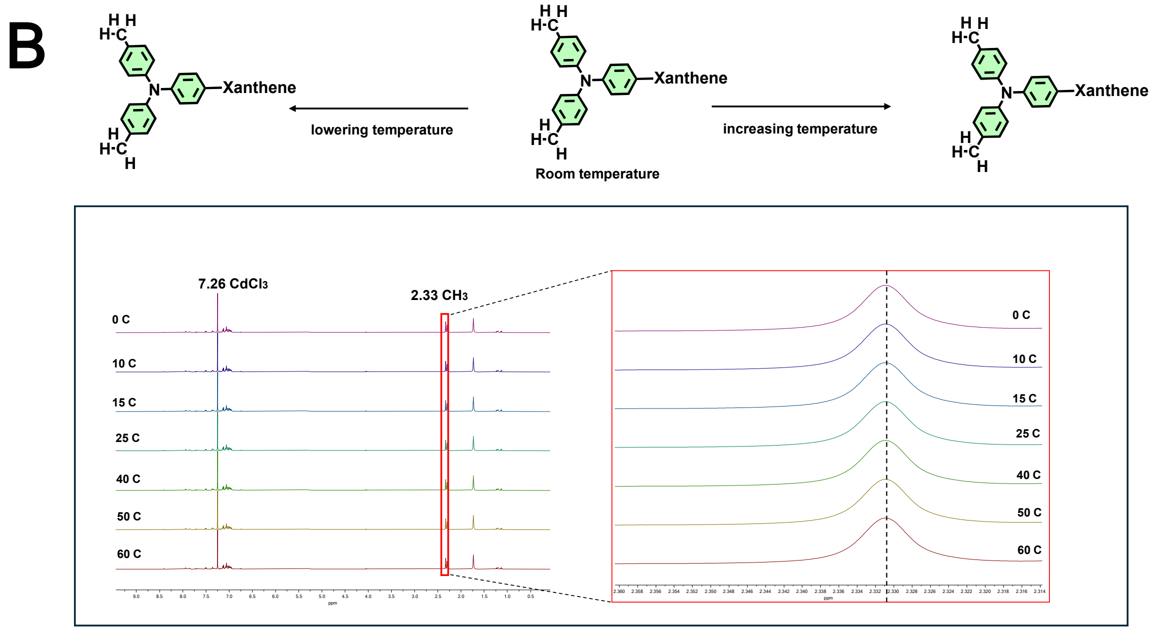


**Figure S7.** Temperature-dependent (0°C to 60°C) $^{1}$H NMR chemical shift of (A) T-X-T+Pttc-TTQ dye dissolved in $CDCl_3$ solvent. (B) T-X-T control dye dissolved in $CDCl_3$ solvent.

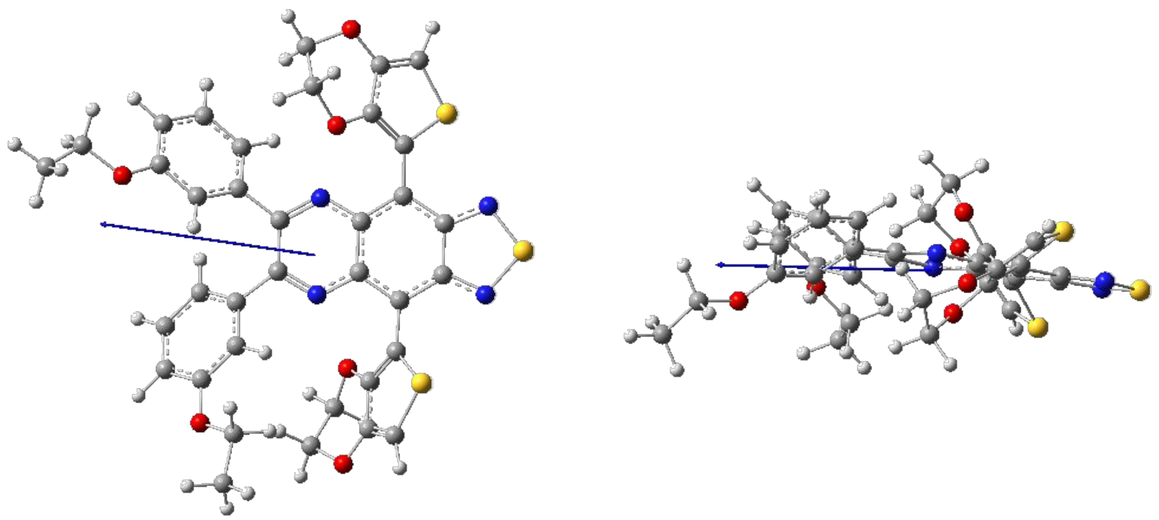

(a) top-view (b) side-view

**Figure S8.** The optimized structure of **TTQ** at the theoretical level of B3LYP-D3/6-31G(d). The arrow is the dipole moment from the negative charge to the positive charge. Color in brown, light blue, red, dark blue, and white are carbon, nitrogen, oxygen, silicon, and hydrogen, respectively.

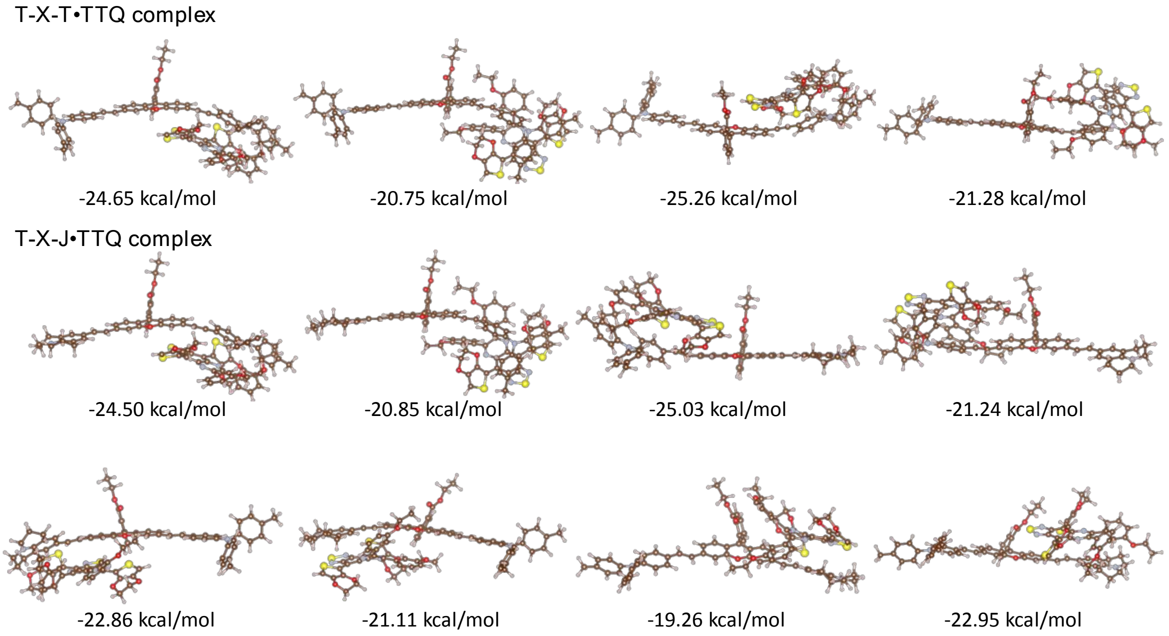


**Figure S9.** The optimized structure of all **T-X-T•TTQ** and **T-X-J•TTQ** complexes at the theoretical level of B3LYP-D3/6-31G(d). Color in brown, light blue, red, yellow, blue and white are carbon, nitrogen, oxygen, sulfur, silicon and hydrogen, respectively.

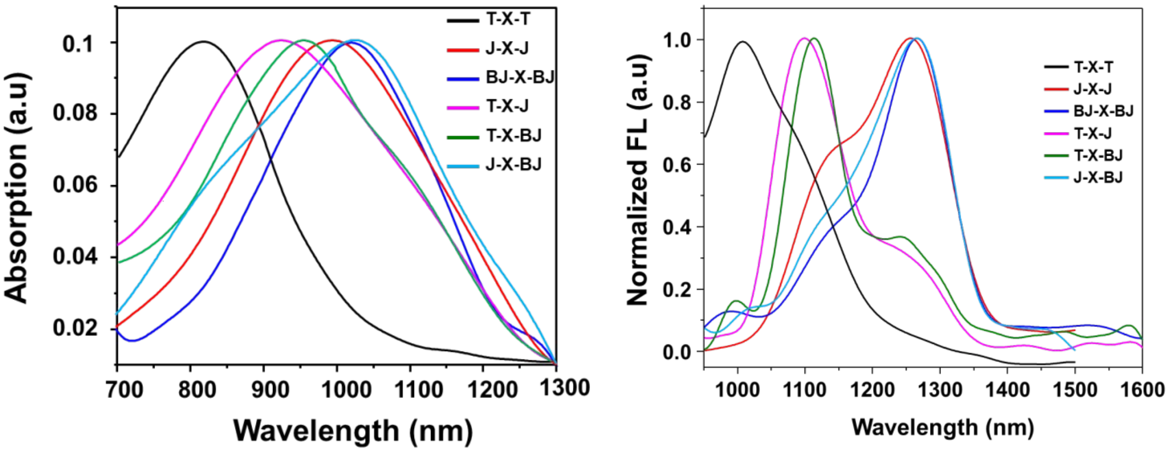


**Figure S10.** The absorption and emission profile of **T-X-T, J-X-J, BJ-X-BJ, T-X-J, T-X-BJ, J-X-BJ** as Pdots assembled with Pttc-TTQ/mPEG-DSPE in aqueous medium.

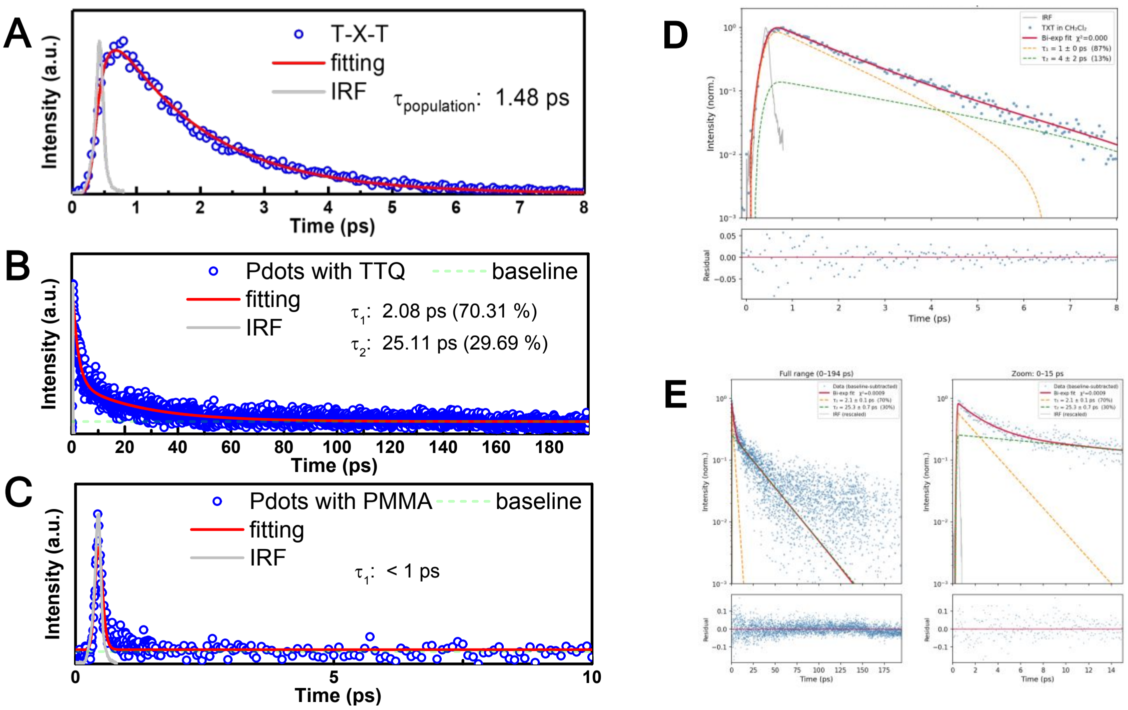


**Figure S11**. Ultrafast fluorescence up-conversion measurements of **T-X-T** in $CH_2Cl_2$ solution and aggregate states using a 35-fs laser system. The instrument response function (IRF) was ~150 fs. (A) **T-X-T** in $CH_2Cl_2$ was monitored at 1200 nm, and (B) aggregated **T-X-T_Pttc-TTQ** Pdots was monitored at 1000 nm. (C) aggregated **T-X-T_PMMA** Pdots was monitored at 1200 nm. Bi-exponential decay model, which numerically convolved with a Gaussian instrument response function (IRF) using the Levenberg-Marquardt algorithm (D) **T-X-T** in $CH_2Cl_2$ (E) **T-X-T_Pttc-TTQ** in Pdots.

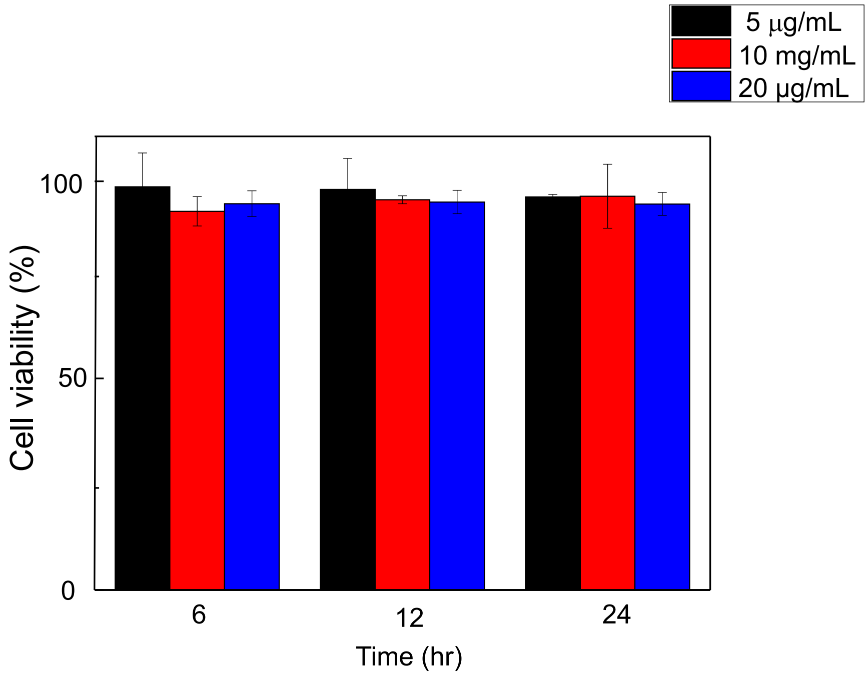


**Figure S12.** The cytotoxicity of **T-Si-BJ** Pdots assembled with Pttc-TTQ/mPEG-DSPE toward HeLa cells was assessed using the MTT assay. Cells were treated with varying concentrations of the Pdots (5-20 µg/mL) and incubated for different durations ranging from 6 to 24 hours. Each experimental condition was analyzed with a sample size of five (n = 5).

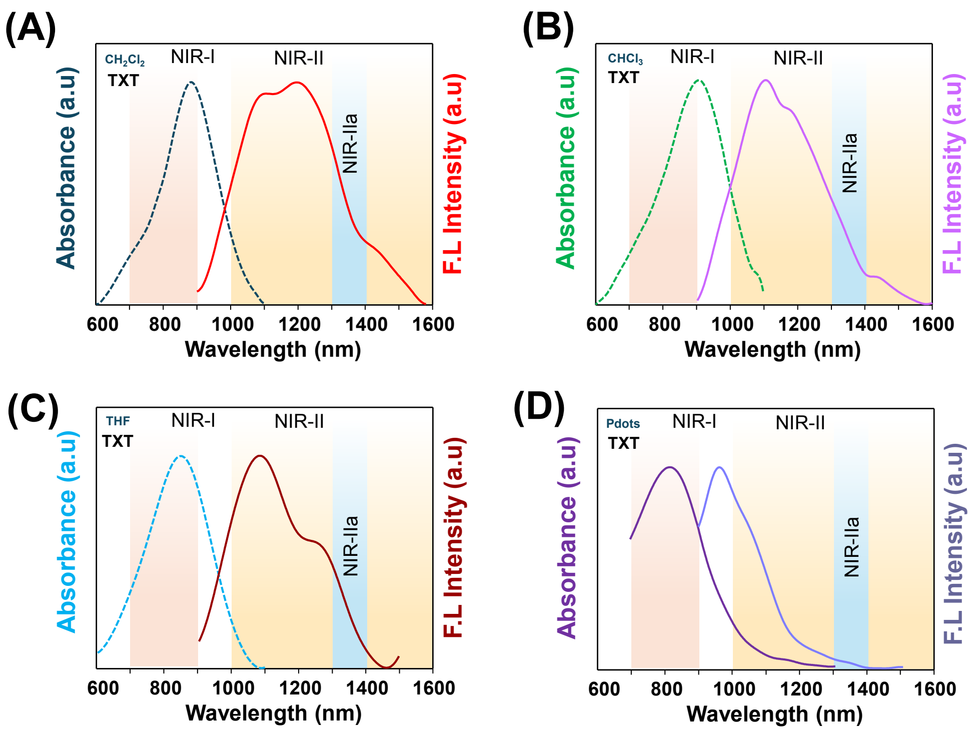


**Figure S13.** Absorption and emission spectra of **T-X-T** measured in (A) $CH_2Cl_2$ without polymer, (B) $CHCl_3$ without polymer, (C) THF without polymer, and (D) $H_2O$ (Pdots assembled with Pttc-TTQ/mPEG-DSPE) at concentration of 5 µM.

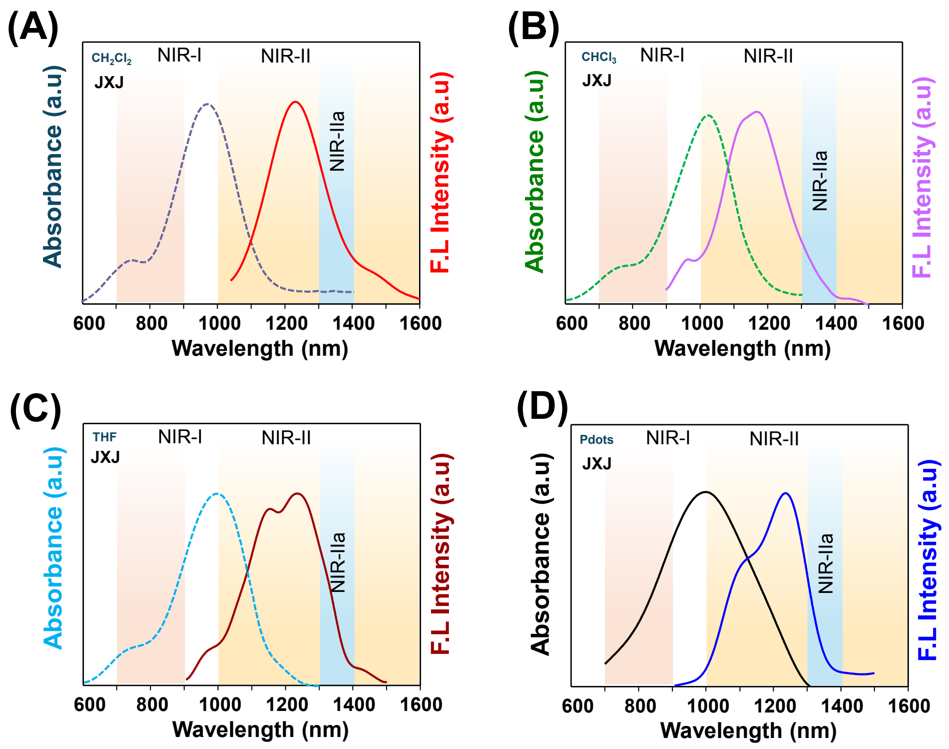


**Figure S14.** Absorption and emission spectra of **J-X-J** measured in (A) $CH_2Cl_2$ without polymer, (B) $CHCl_3$ without polymer, (C) THF without polymer, and (D) $H_2O$ (Pdots assembled with Pttc-TTQ/mPEG-DSPE) at concentration of 5 μM.

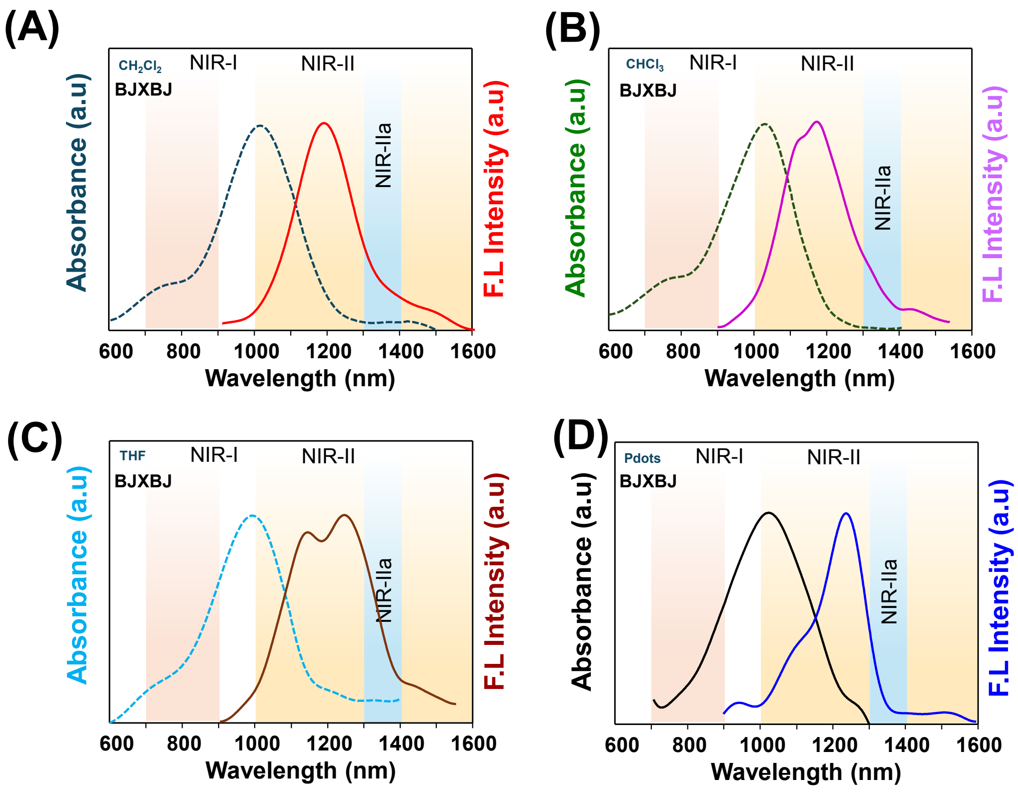


**Figure S15.** Absorption and emission spectra of **BJ-X-BJ** measured in (A) $CH_2Cl_2$ without polymer, (B) $CHCl_3$ without polymer, (C) THF without polymer, and (D) $H_2O$ (Pdots assembled with Pttc-TTQ/mPEG-DSPE) at concentration of 5 μM.

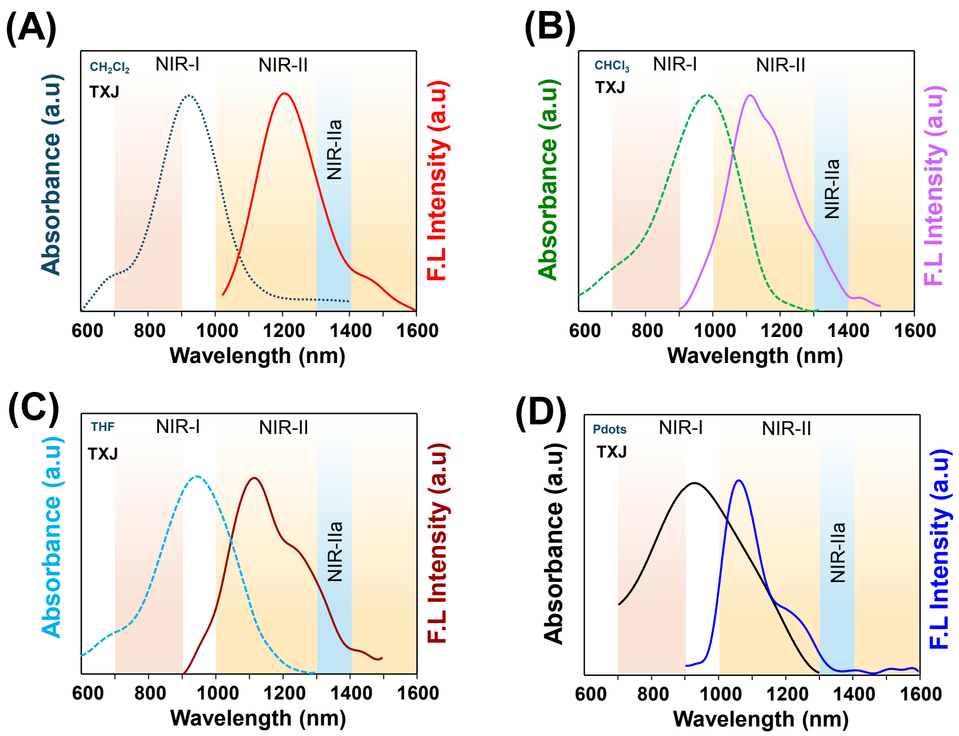


**Figure S16.** Absorption and emission spectra of **T-X-J** measured in (A) $CH_2Cl_2$ without polymer, (B) $CHCl_3$ without polymer, (C) THF without polymer, and (D) $H_2O$ (Pdots assembled with Pttc-TTQ/mPEG-DSPE) at concentration of 5 µM.

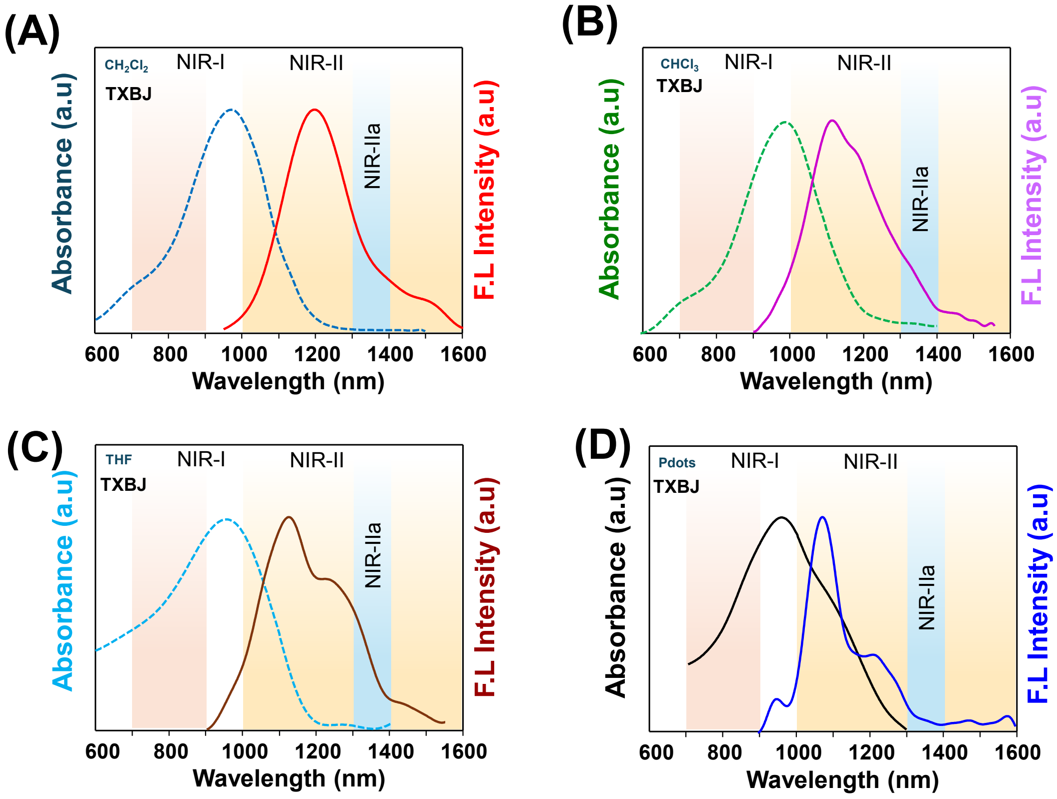


**Figure S17.** Absorption and emission spectra of **T-X-BJ** measured in (A) $CH_2Cl_2$ without polymer, (B) $CHCl_3$ without polymer, (C) THF without polymer, and (D) $H_2O$ (Pdots assembled with Pttc-TTQ/mPEG-DSPE) at concentration of 5 µM.

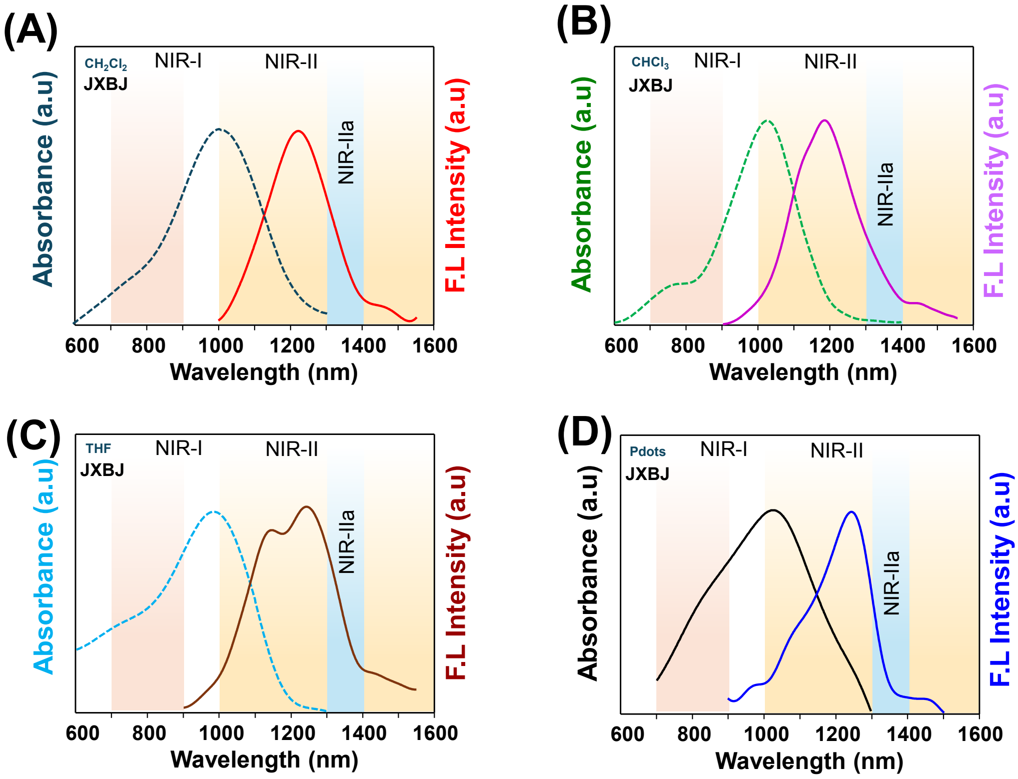


**Figure S18.** Absorption and emission spectra of **J-X-BJ** measured in (A) $CH_2Cl_2$ without polymer, (B) $CHCl_3$ without polymer, (C) THF without polymer, and (D) $H_2O$ (Pdots assembled with Pttc-TTQ/mPEG-DSPE) at concentration of 5 μM.

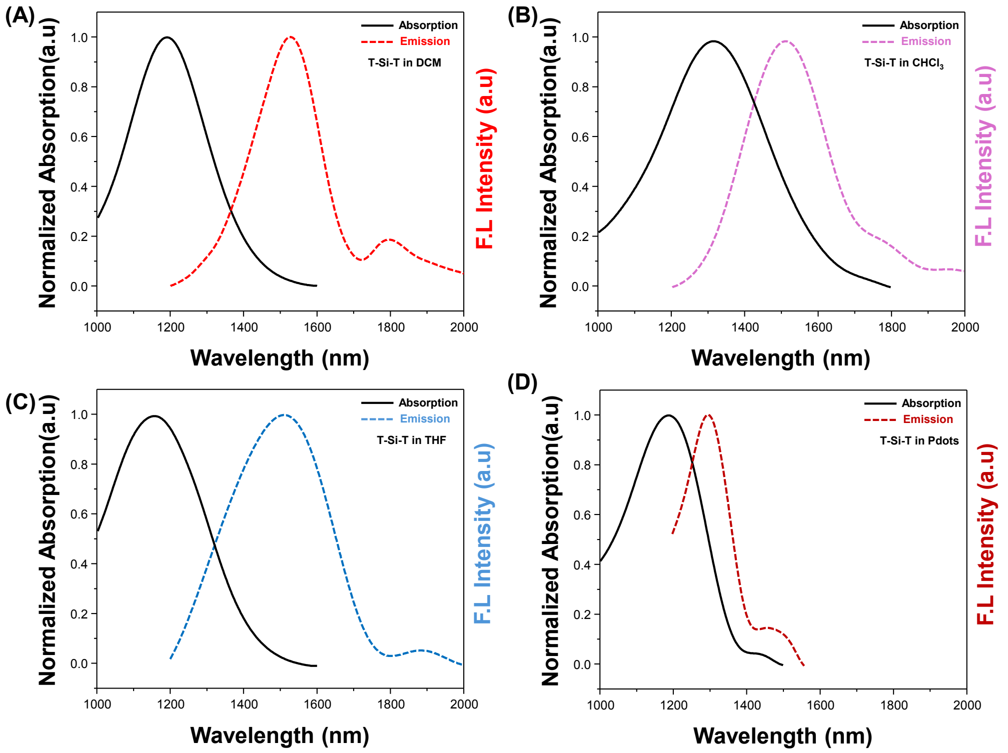


**Figure S19.** Absorption and emission spectra of **T-Si-T** measured in (A) $CH_2Cl_2$ without polymer, (B) $CHCl_3$ without polymer, (C) THF without polymer, and (D) $H_2O$ (Pdots assembled with Pttc-TTQ/mPEG-DSPE) at concentration of 5 μM.

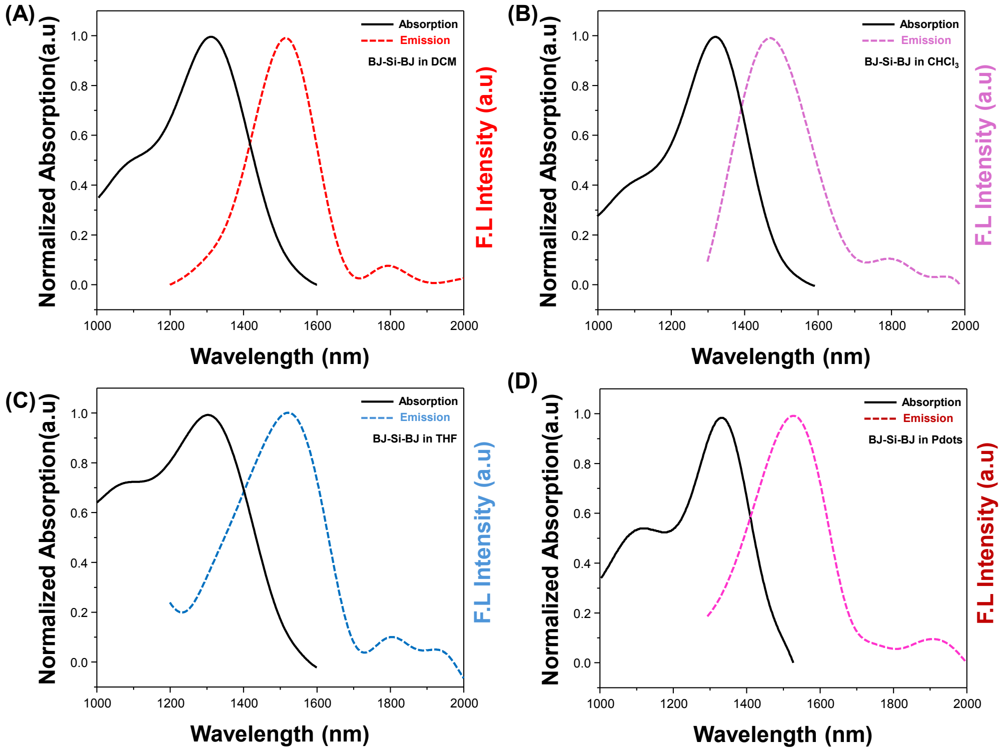


**Figure S20.** Absorption and emission spectra of **BJ-Si-BJ** measured in (A) $CH_2Cl_2$ without polymer, (B) $CHCl_3$ without polymer, (C) THF without polymer, and (D) $H_2O$ (Pdots assembled with Pttc-TTQ/mPEG-DSPE) at concentration of 5 μM.

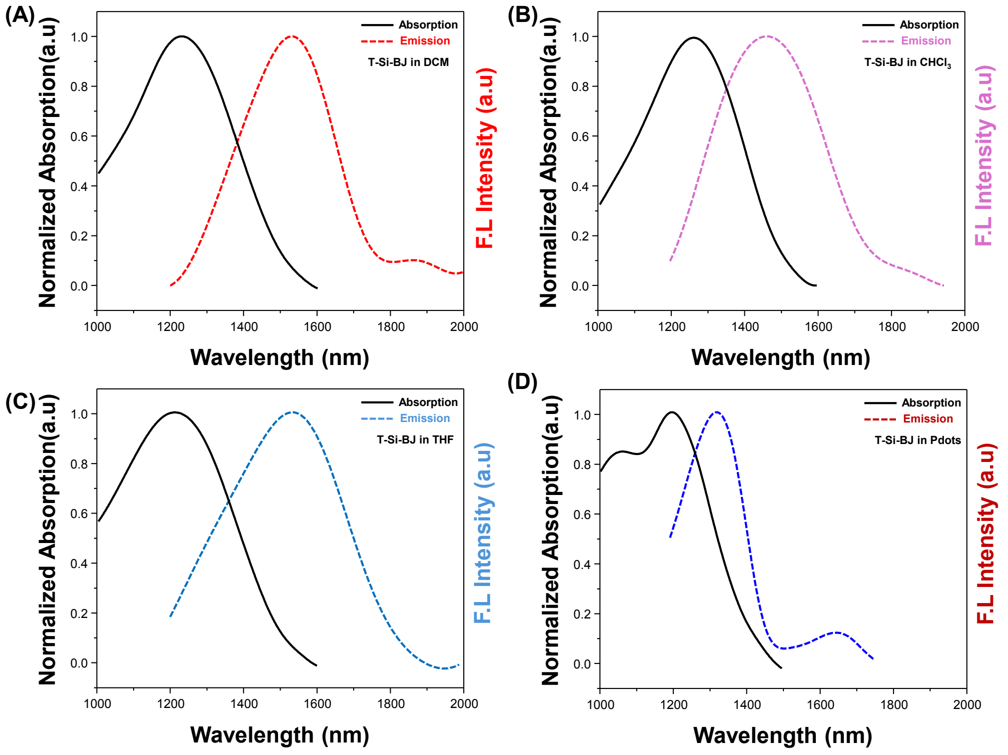


**Figure S21.** Absorption and emission spectra of **T-Si-BJ** measured in (A) $CH_2Cl_2$ without polymer, (B) $CHCl_3$ without polymer, (C) THF without polymer, and (D) $H_2O$ (Pdots assembled with Pttc-TTQ/mPEG-DSPE) at concentration of 5 μM.

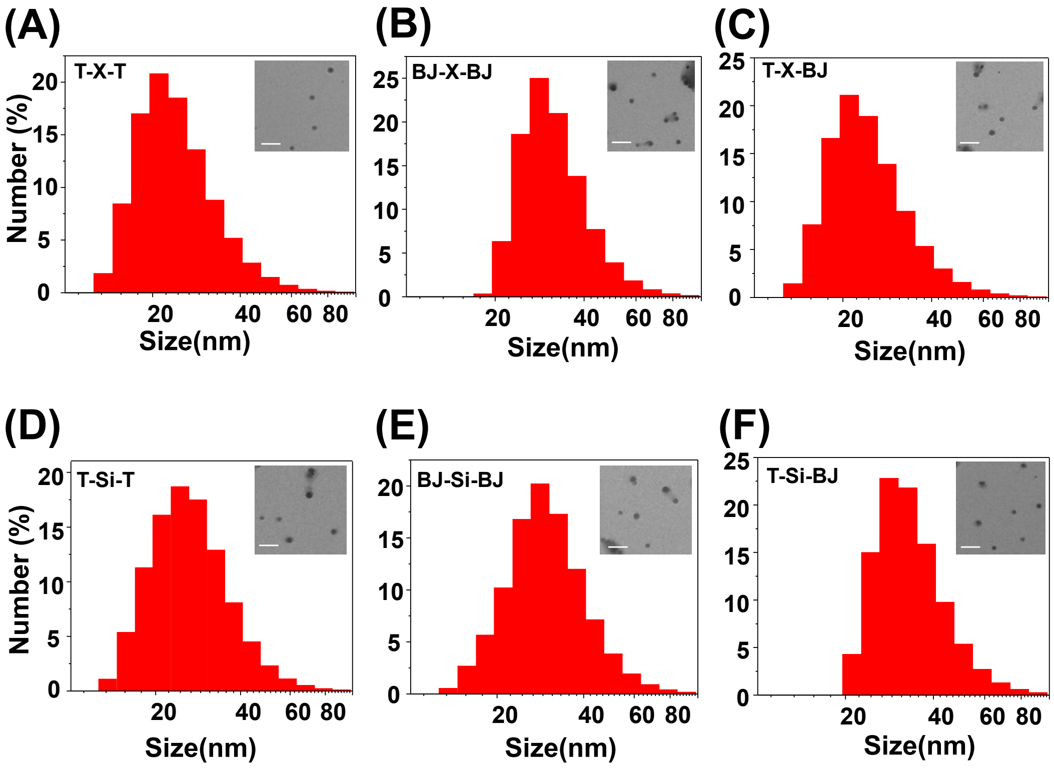


**Figure S22.** The hydrodynamic diameters of (A) **T-X-T**, (B) **BJ-X-BJ**, (C) **T-X-BJ**, (D) **T-Si-T**, (E) **BJ-Si-BJ** and (F) **T-Si-BJ** Pdots assembled with Pttc-TTQ/mPEG-DSPE were determined. The inset panels display the corresponding TEM images, each with a scale bar of 100 nm. The average particle sizes were 25 ± 9 nm, 32 ± 10 nm, 27 ± 8 nm, 29 ± 10 nm, 31 ± 11 nm and 34 ± 12 nm for **T-X-T**, **BJ-X-BJ**, **T-X-BJ**, **T-Si-T**, **BJ-Si-BJ**, and **T-Si-BJ** Pdots, respectively. Data from DLS measurements are presented as mean ± standard deviation, with each experiment performed in triplicate (n = 3).

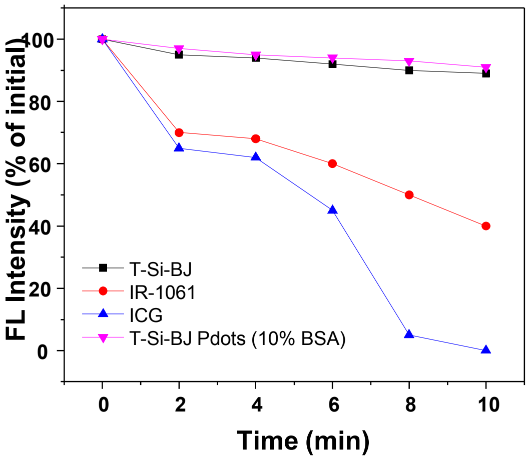


**Figure S23.** The comparison of photostability between reference **ICG**, **IR-1061** vs **T-Si-BJ** dissolved in $CH_2Cl_2$ and **T-Si-BJ Pdots** (10% BSA) under continuous 254 nm UV irradiation. The concentration is 0.01 mg mL−1 for all probes. The UV irradiation we used is 300 mW/cm$^2$.

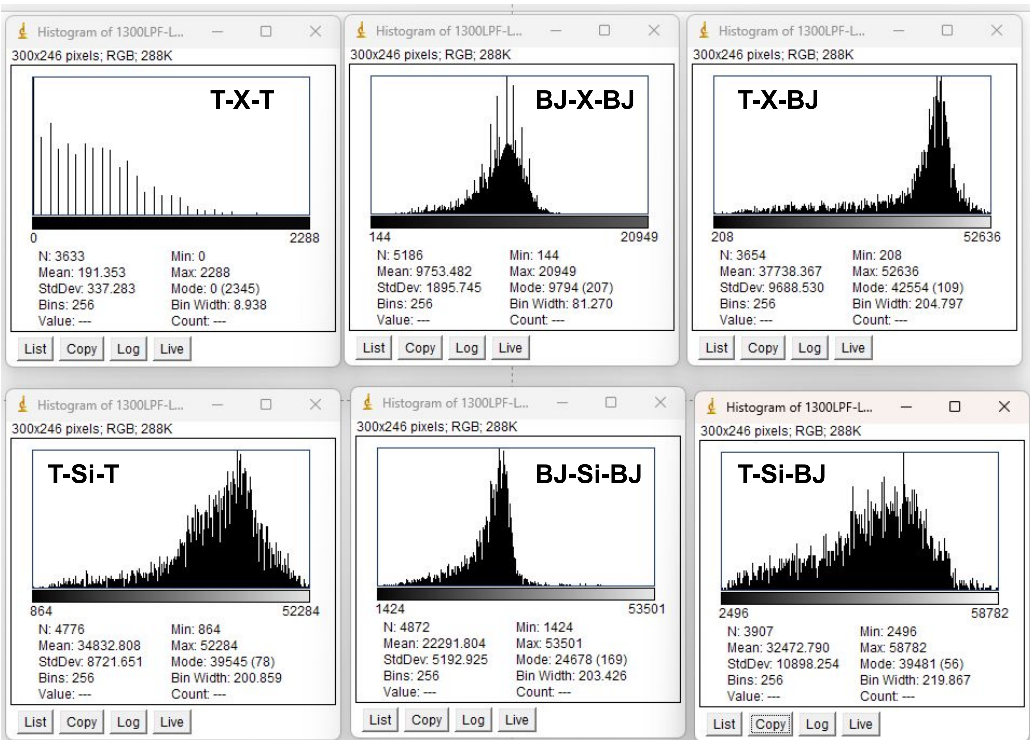


**Figure S24.** Real-time photon counts were recorded under 1064 nm laser excitation for **T-X-T**, **BJ-X-BJ**, **T-X-BJ**, **T-Si-T**, **BJ-Si-BJ**, and **T-Si-BJ** Pdots assembled with Pttc-TTQ/mPEG-DSPE. The data were plotted to compare their emission brightness under a 1300 LPF filter.

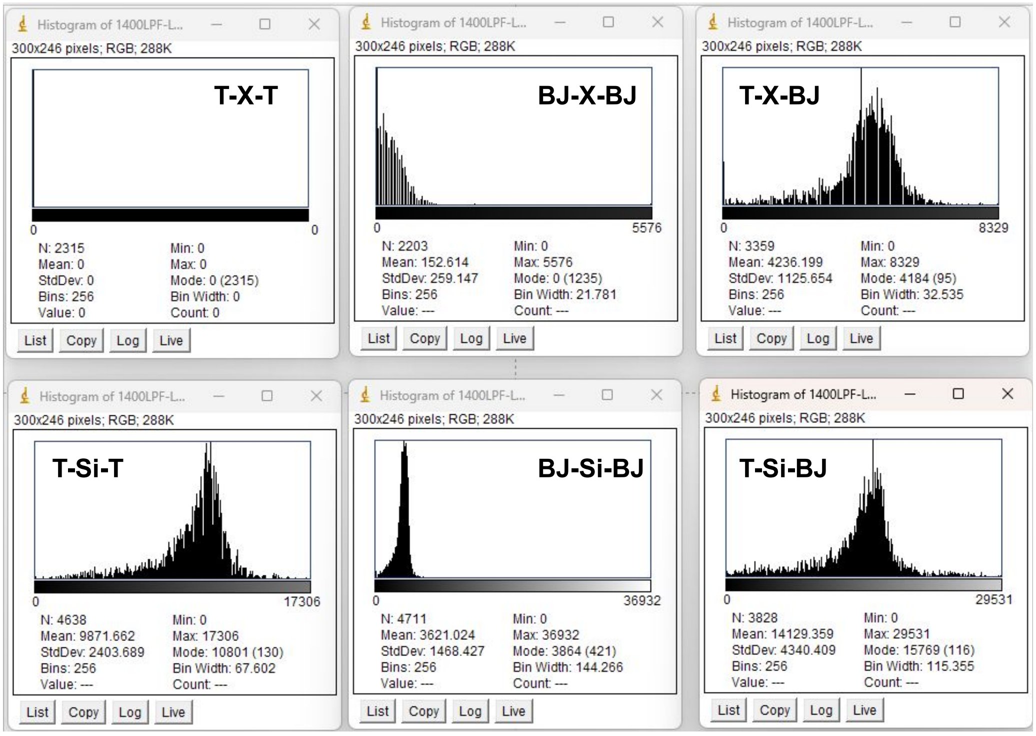


**Figure S25.** Real-time photon counts were recorded under 1064 nm laser excitation for **T-X-T**, **BJ-X-BJ**, **T-X-BJ**, **T-Si-T**, **BJ-Si-BJ**, and **T-Si-BJ** Pdots assembled with Pttc-TTQ/mPEG-DSPE. The data were plotted to compare their emission brightness under a 1400 LPF filter.

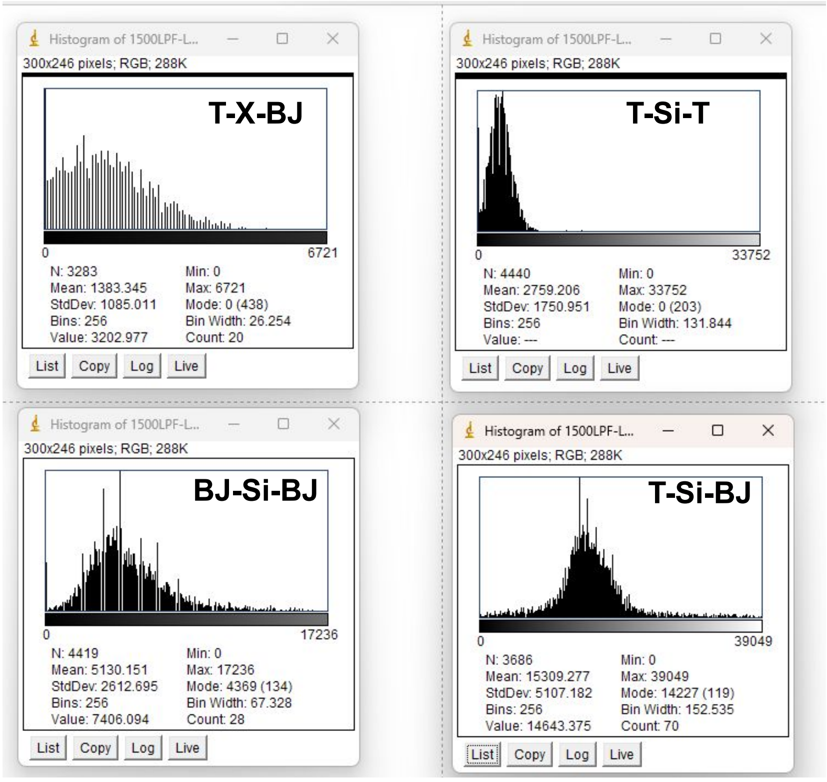


**Figure S26.** Real-time photon counts were recorded under 1064 nm laser excitation for **T-X-BJ**, **T-Si-T**, **BJ-Si-BJ**, and **T-Si-BJ** Pdots assembled with Pttc-TTQ/mPEG-DSPE. The data were plotted to compare their emission brightness under a 1500 LPF filter.

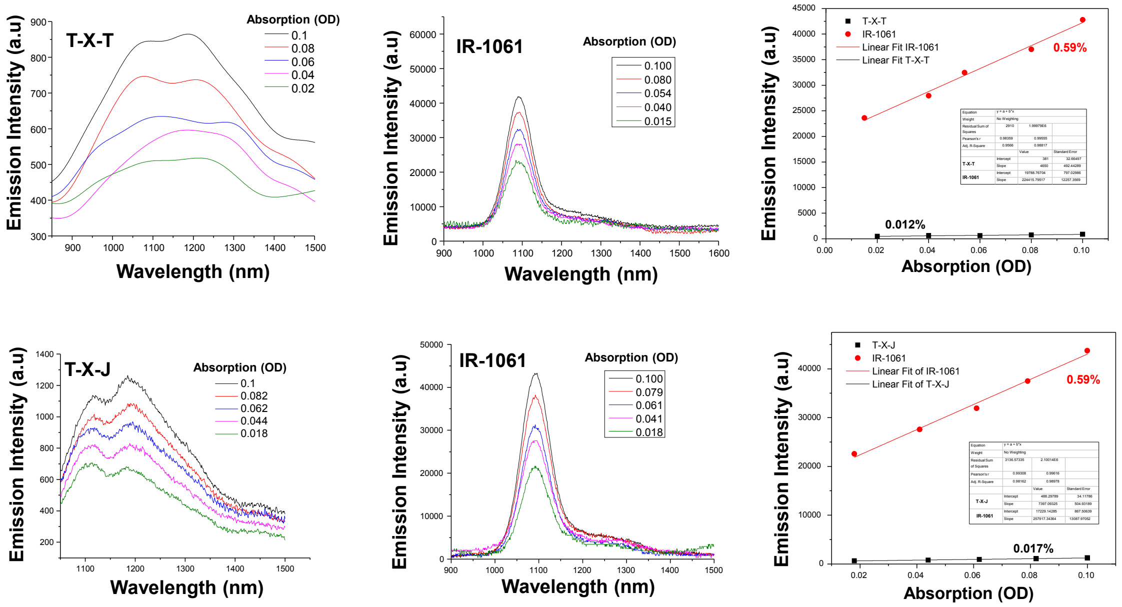


**Figure S27.** Data of fluorescence quantum yield measurements of **T-X-T** (upper panel) and **T-X-J** (bottom panel) in comparison to the reference dye, IR-1061.

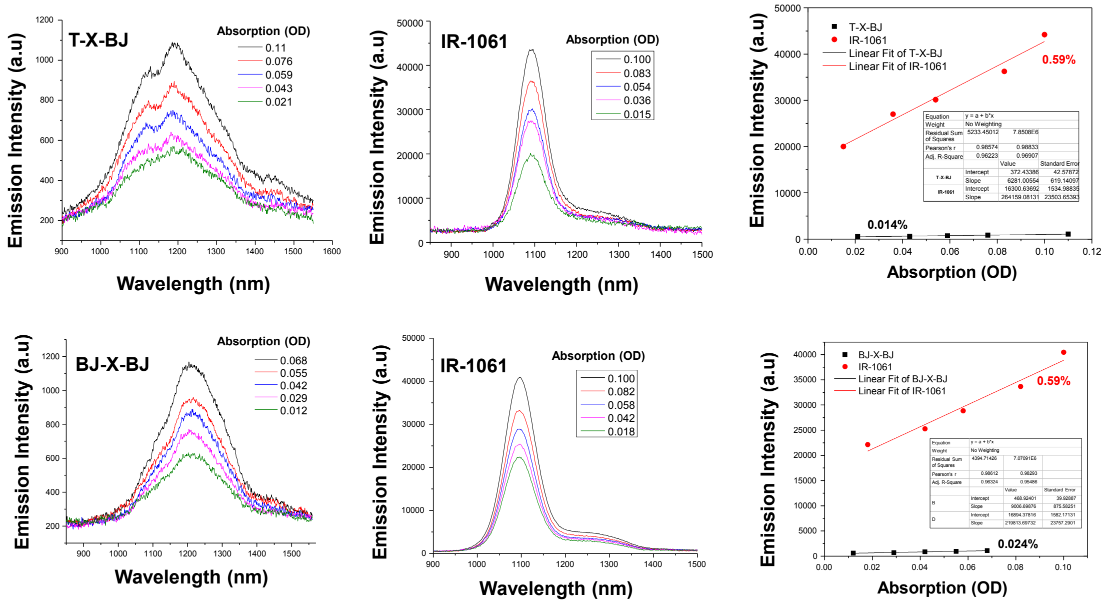


**Figure S28.** Data of fluorescence quantum yield measurements of **T-X-BJ** (upper panel) and **BJ-X-BJ** (bottom panel) in comparison to the reference dye, IR-1061.

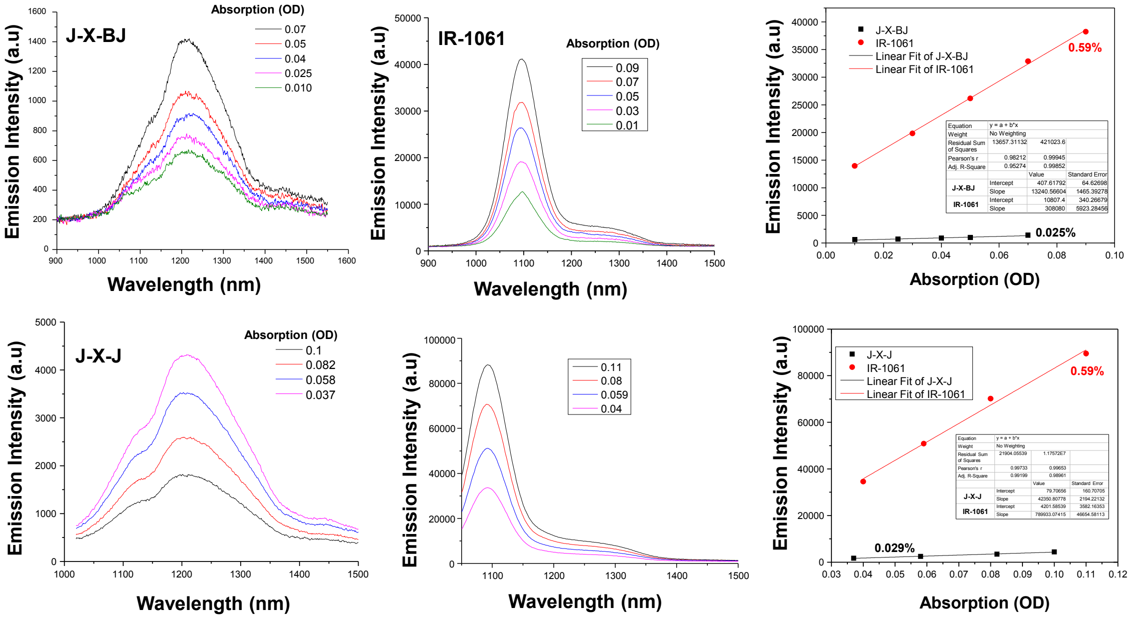


**Figure S29.** Data of fluorescence quantum yield measurements of **J-X-BJ** (upper panel) and **J-X-J** (bottom panel) in comparison to the reference dye, IR-1061.

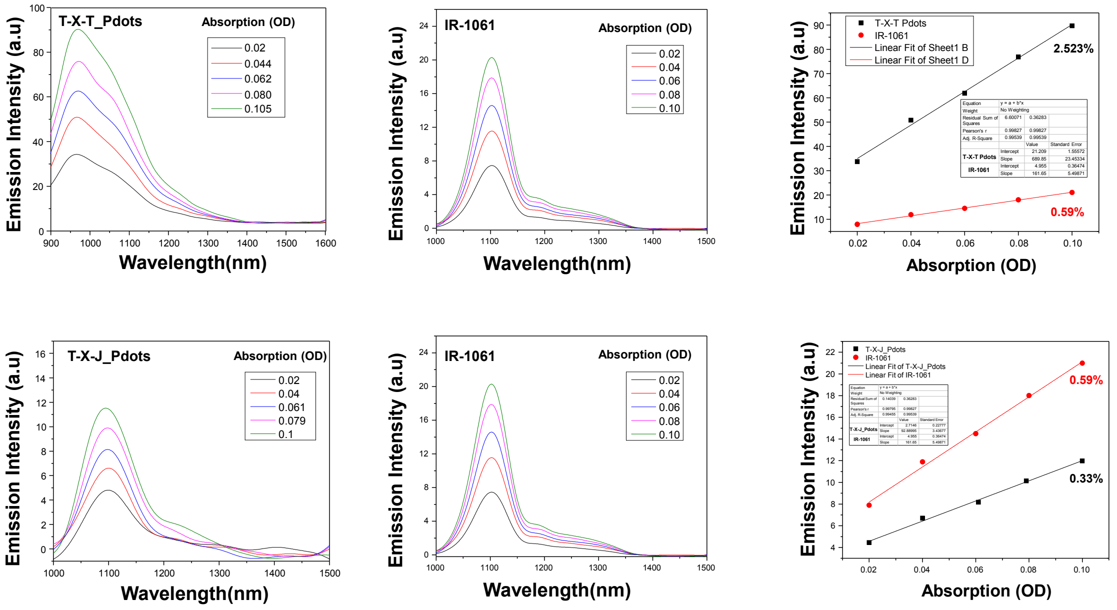


**Figure S30.** Data of fluorescence quantum yield measurements of **T-X-T** (upper panel) and **T-X-J** (bottom panel) Pdots in comparison to the reference dye, IR-1061.

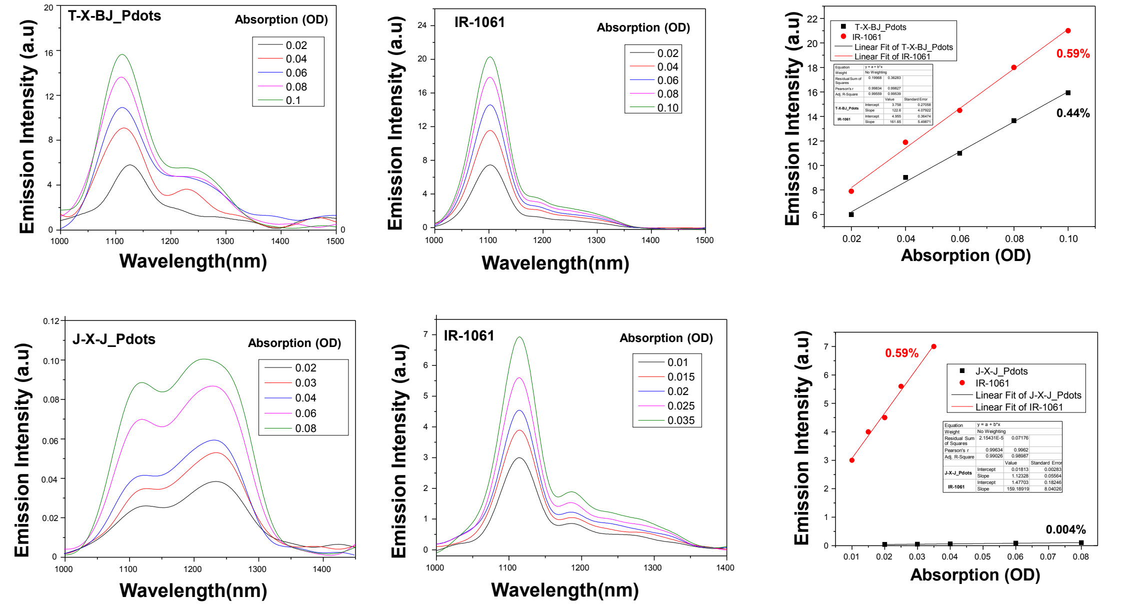


**Figure S31.** Data of fluorescence quantum yield measurements of **T-X-BJ** (upper panel) and **J-X-J** (bottom panel) Pdots in comparison to the reference dye, IR-1061.

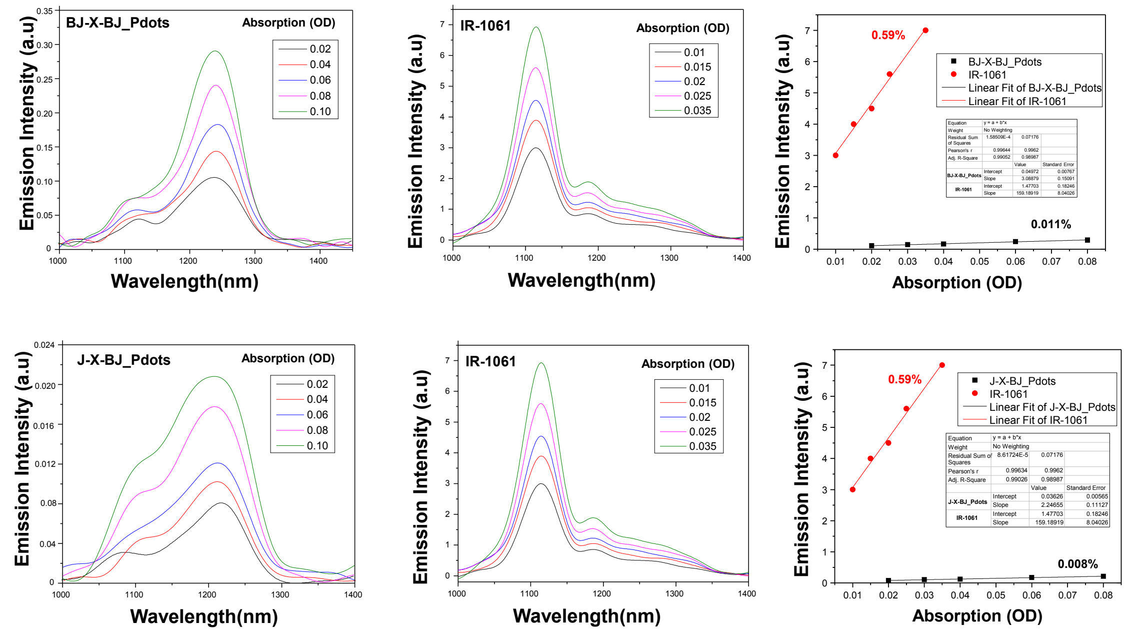


**Figure S32.** Data of fluorescence quantum yield measurements of **BJ-X-BJ** (upper panel) and **J-X-BJ** (bottom panel) Pdots in comparison to the reference dye, IR-1061.

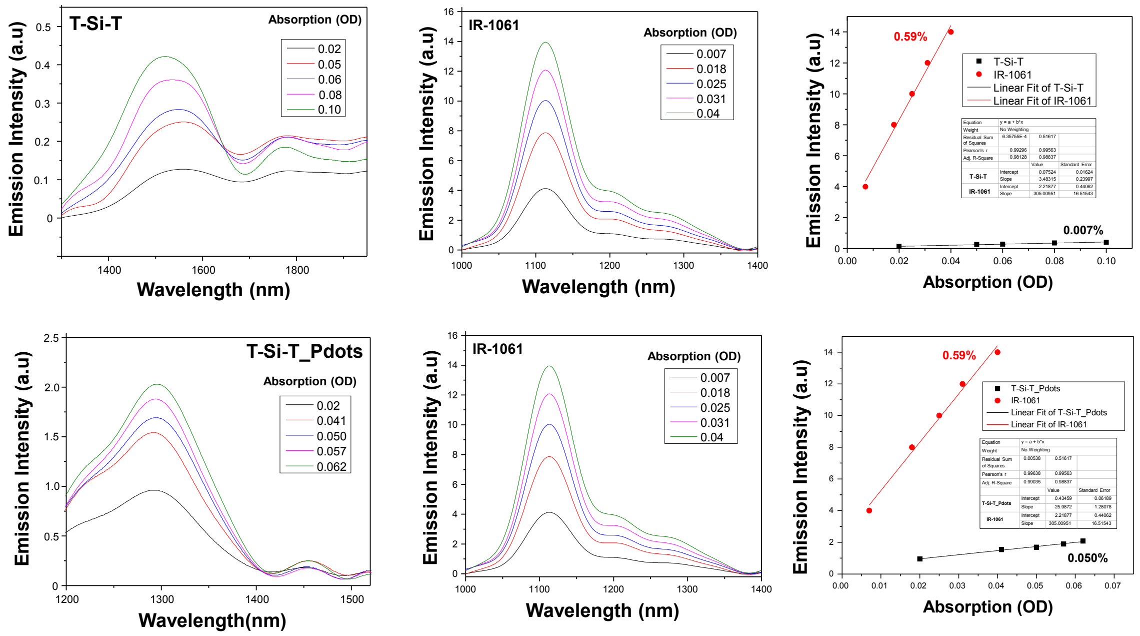


**Figure S33.** Data of fluorescence quantum yield measurements of **T-Si-T** (upper panel) and **T-Si-T** Pdots (bottom panel) in comparison to the reference dye, IR-1061.

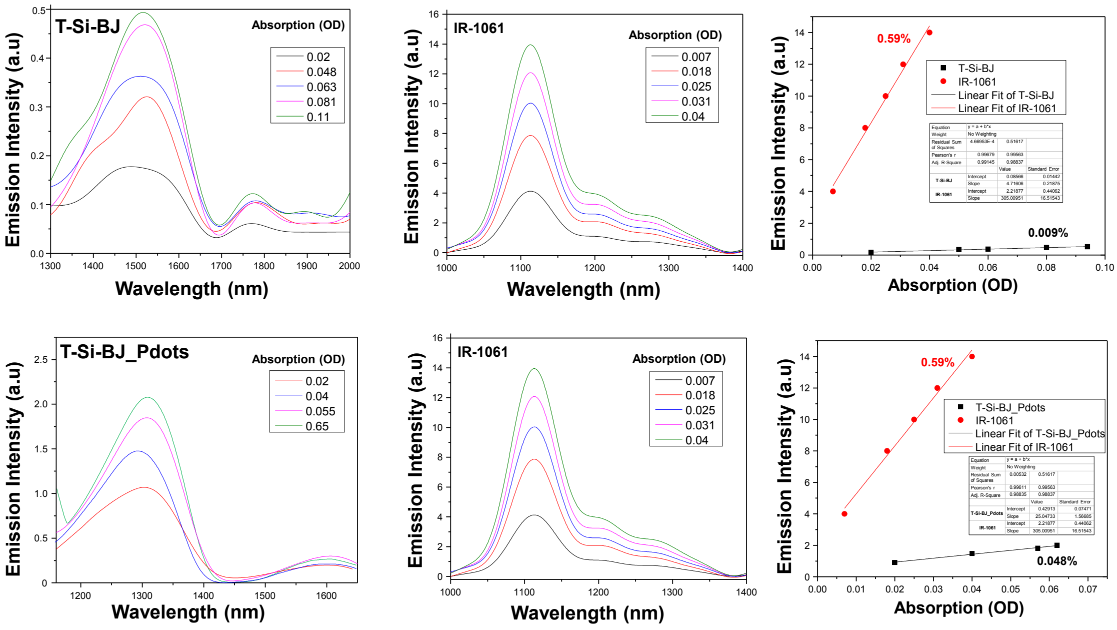


**Figure S34.** Data of fluorescence quantum yield measurements of **T-Si-BJ** (upper panel) and **T-Si-BJ** Pdots (bottom panel) in comparison to the reference dye, IR-1061.

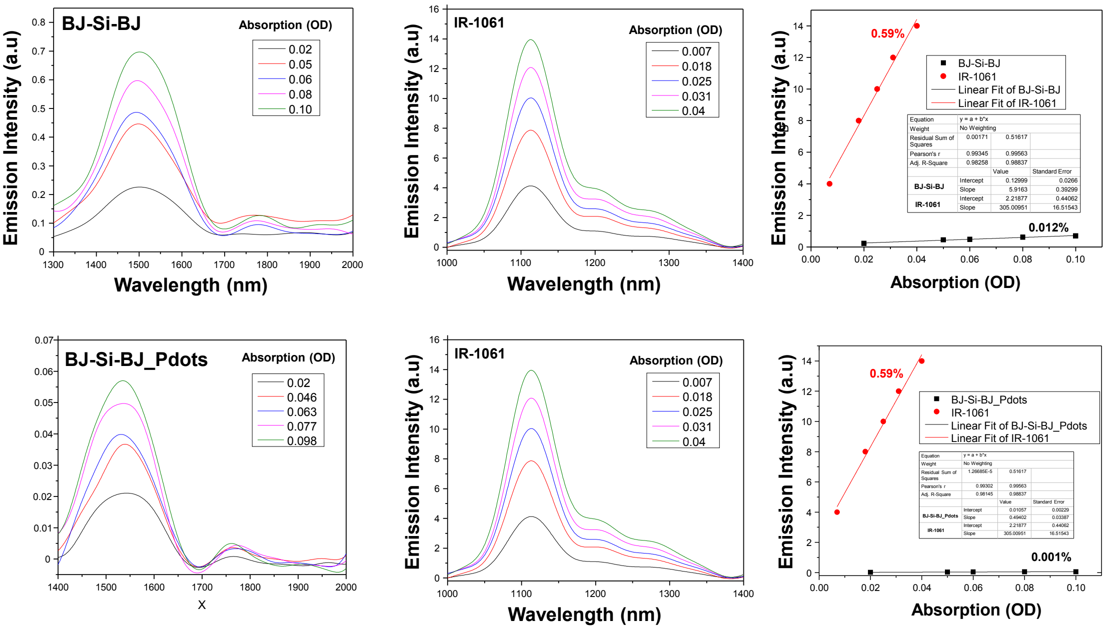


**Figure S35.** Data of fluorescence quantum yield measurements of **BJ-Si-BJ** (upper panel) and **BJ-Si-BJ** Pdots (bottom panel) in comparison to the reference dye, IR-1061.

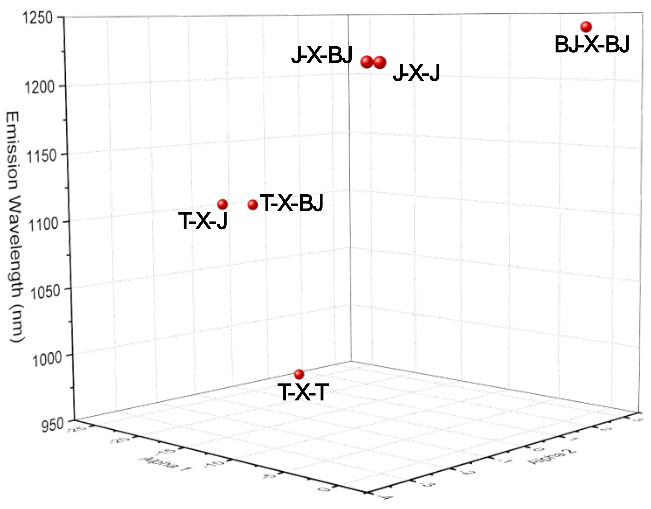


**Figure S36.** Quantitative correlation between dihedral angle of linker (C=C) relative to donor (**T**, **J**, and **BJ**) (□1 and □2) with emission wavelength for oxygen-containing xanthenes (**T-X-T**, **T-X-J**, **T-X-BJ**, **J-X-J**, **J-X-BJ**, and **BJ-X-BJ**).

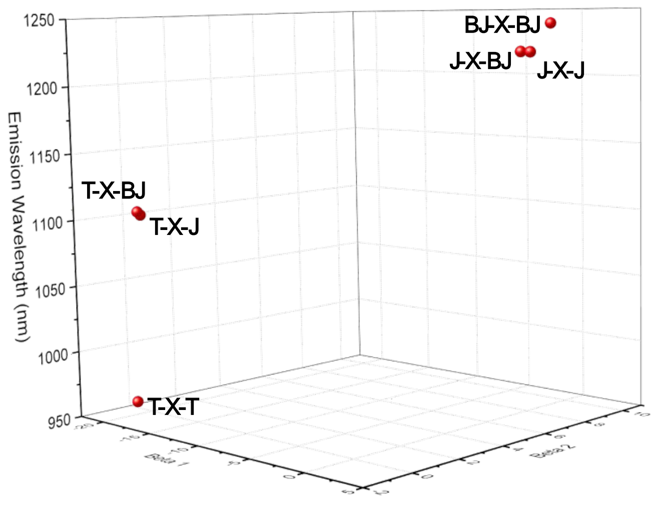


**Figure S37.** Quantitative correlation between dihedral angle of linker (C=C) relative to the xanthene core (□1 and □2) with emission wavelength for oxygen-containing xanthenes (**T-X-T**, **T-X-J**, **T-X-BJ**, **J-X-J**, **J-X-BJ**, and **BJ-X-BJ**).

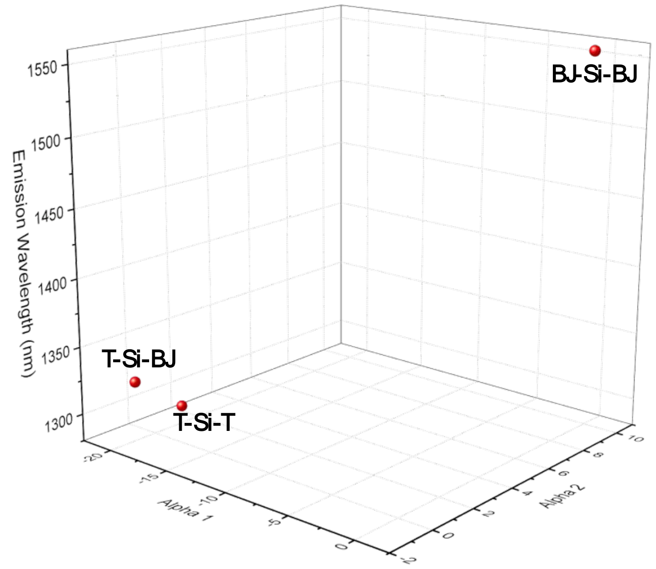


**Figure S38.** Quantitative correlation between dihedral angle of linker (C=C) relative to donor (**T**, **J**, and **BJ**) (□1 and □2) with emission wavelength for silicon-containing xanthenes (**T-Si-T**, **T-Si-BJ**, and **BJ-Si-BJ**).

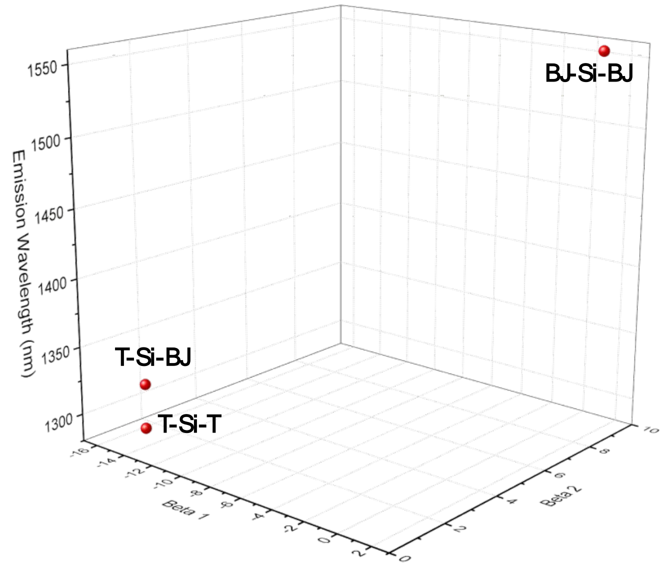


**Figure S39.** Quantitative correlation between dihedral angle of linker (C=C) relative to the xanthene core (□1 and □2) with emission wavelength for silicon-containing xanthenes (**T-Si-T**, **T-Si-BJ**, and **BJ-Si-BJ**).

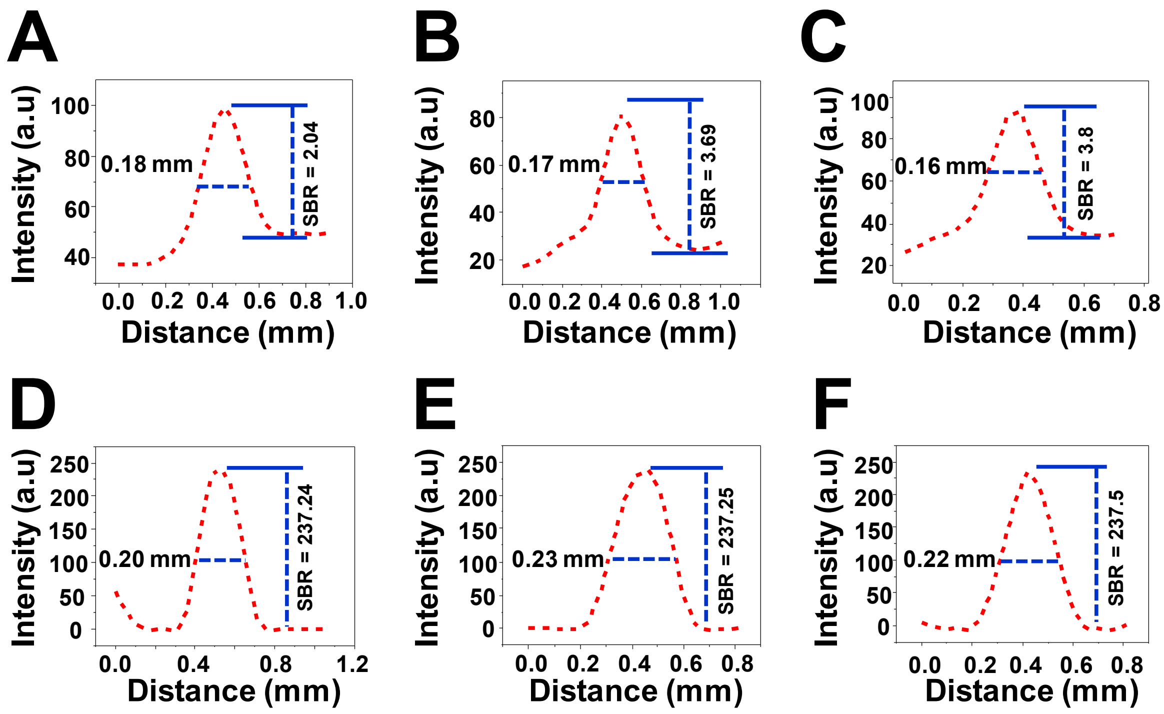


**Figure S40**. The corresponding cross-sectional fitting intensities in mice at the supine position injected by **T-Si-BJ** Pdots with (A)1300, (B)1400, and (C)1500 nm LPFs, along the red lines in **Figure 6** (top panel). (F-H) AI-enhanced corresponding cross-sectional fitting intensities along the red lines in at (D)1300, (E)1400, and (F)1500 nm LPFs (bottom panel).

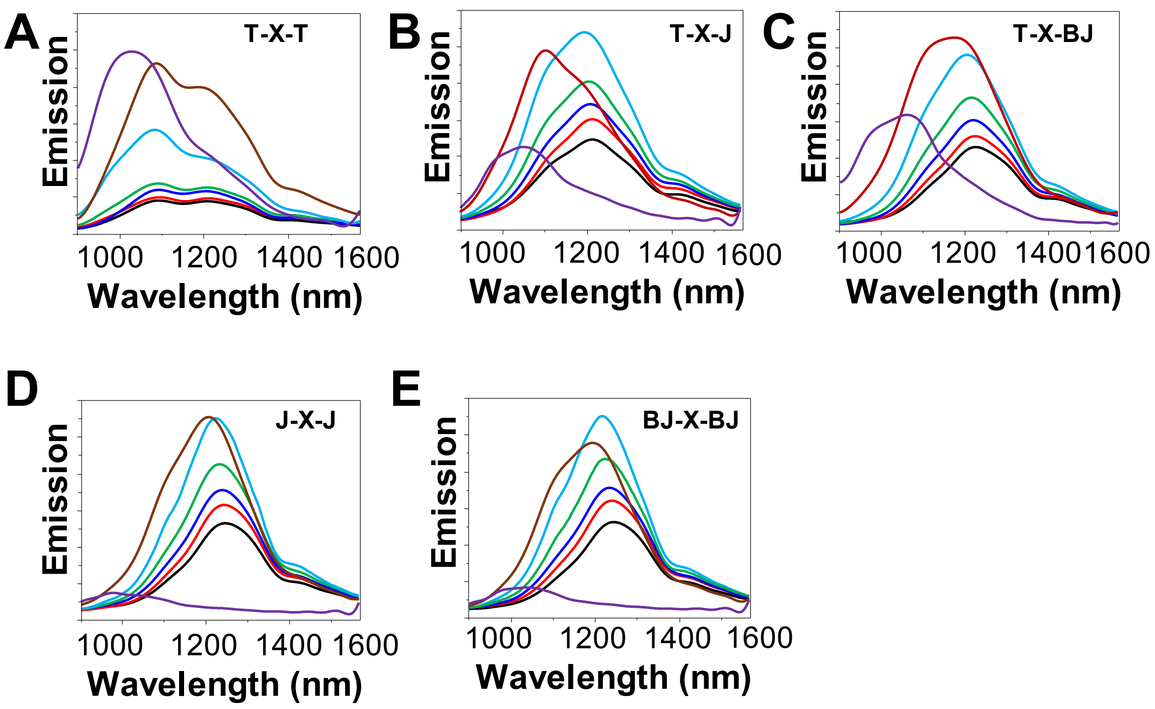


**Figure S41**. The FL intensity variation was monitored at increasing % toluene (v/v) in the DMSO/toluene mixture. (A) **T-X-T**, (B) **T-X-J**, (C) **T-X-BJ**, (D) **J-X-J**, and (E) **BJ-X-BJ**. Color in black, red, blue, green, sky blue, brown, and purple represent 0%, 20%, 40%, 60%, 80%, 90%, and 99% toluene fraction (v/v), respectively, in DMSO.

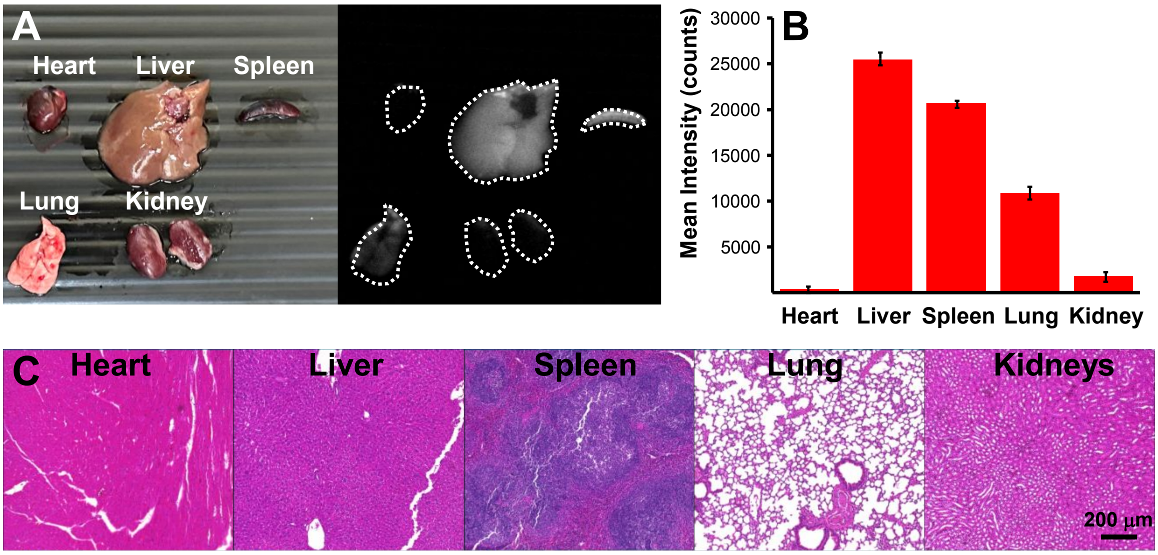


D

| Biochemical Tests | PBS | P-dots | Biochemical Tests | PBS | P-dots |
|---|---|---|---|---|---|
| ALanine aminotransferase (U/L) | 44 | 24 | ASpartate aminotransferase (U/L) | 77 | 72 |
| Liver and Gallbladder-Total Bilirubin (mg/dL) | 0.33 | 0.20 | Gamma Glutamyl Transpeptidase (U/L) | 3 | 3 |
| Blood Urea Nitrogen (mg/dL) | 23 | 22 | Alkaline Phosphatase (U/L) | 77 | 55 |
| Creatinine (mg/dL) | 0.2 | 0.3 | Calcium (mg/dL) | 10.8 | 9.5 |
| Phosphate (mg/dL) | 7 | 6.4 | Amylase (U/L) | 509 | 533 |
| Glucose (mg/dL) | 115 | 138 | Globulin (g/dL) | 2.9 | 2.7 |
| Total protein (g/dL) | 6.4 | 5 | Albumin/Globulin | 1 | 1.2 |
| Albumin (g/dL) | 2.9 | 2.7 | Blood Urea Nitrogen /Creatinine | 60 | 73.3 |

**Figure S42.** (A) Biodistribution of T-Si-BJ Pdots assembled with Pttc-TTQ/mPEG-DSPE in major excised organs at 24 h post-injection in which the bright-field image (left) and NIR-II fluorescence imaging (right) of different organs were shown. (B) The corresponding quantitative mean fluorescence intensities in organs. (C) H&E staining of organ sections from Pdot-treated mouse at 24 h post-injection. (D) Serum biochemical analysis of PBS-treated and Pdot-treated mice at 24 h post-injection.

**Table S1**. The vertical excitation energy ($E_{abs}$, eV) and absorption wavelength ($l_{abs}$, nm) of **T-X-T**, **J-X-J** and **BJ-X-BJ** at the TD-DFT($CH_2Cl_2$,CPCM)/6-31G* and SOS-CIS(D)($CH_2Cl_2$,CPCM)/VDZ.

| | Method | T-X-T | | J-X-J | | BJ-X-BJ | |
|---|---|---|---|---|---|---|---|
| Exp. | | $E_{abs}$ | $\lambda_{abs}$ | $E_{abs}$ | $\lambda_{abs}$ | $E_{abs}$ | $\lambda_{abs}$ |
| | | 1.4057 | 882 | 1.2227 | 1014 | 1.2096 | 1025 |
| | B3LYP | 1.3716 | 904 | 1.4476 | 856 | 1.4450 | 858 |
| | τHCTHhyb | 1.2884 | 962 | 1.3930 | 890 | 1.3897 | 892 |
| | PBE0 | 1.4517 | 854 | 1.4954 | 829 | 1.4912 | 831 |
| | PBE38 | 1.6838 | 736 | 1.6535 | 750 | 1.6472 | 753 |
| TD-DFT | MN15 | 1.7042 | 728 | 1.6554 | 749 | 1.6535 | 750 |
| | cam-B3LYP | 1.9538 | 635 | 1.8454 | 672 | 1.8384 | 674 |
| | ωB97-XD | 2.1076 | 588 | 1.9670 | 630 | 1.9577 | 633 |
| | LC-ωHPBE | 2.3374 | 530 | 2.1607 | 574 | 2.1470 | 577 |
| | BHHLYP | 1.9277 | 643 | 1.8456 | 672 | 1.8383 | 674 |
| SOS-CIS(D) | | 1.7410 | 712 | 1.4521 | 854 | 1.4177 | 875 |

**Table S2.** Optical properties of symmetric/asymmetric xanthenes in different conditions. $CH_2Cl_2$, $CHCl_3$, and THF: Organic solvents without polymer; $H_2O$: Pdots assembled with Pttc-TTQ/mPEG-DSPE.

| dye | solvents | $\lambda_{max}^{abs}$ (nm) | $\lambda_{max}^{em}$ (nm) | $\varepsilon_{max}$ ($M^{-1}$ $cm^{-1}$) | Φ (%) | brightness ($M^{-1}$ $cm^{-1}$) |
|---|---|---|---|---|---|---|
| **T-X-T** | $CH_2Cl_2$ | 884 | 1220 | $0.605 \times 10^5$ | 0.012 | 7.26 |
| | $CHCl_3$ | 905 | 1106 | -- | -- | -- |
| | THF | 850 | 1080 | -- | -- | -- |
| | Pdots | 826 | 960 | $0.003 \times 10^5$ | 2.523±0.3 | 7.569 |
| **T-X-J** | $CH_2Cl_2$ | 965 | 1192 | $1.720 \times 10^5$ | 0.017 | 29.24 |
| | $CHCl_3$ | 990 | 1126 | -- | -- | -- |
| | THF | 946 | 1120 | -- | -- | -- |
| | Pdots | 960 | 1102 | $0.013 \times 10^5$ | 0.333±0.05 | 4.33 |
| **T-X-BJ** | $CH_2Cl_2$ | 970 | 1200 | $1.680 \times 10^5$ | 0.014 | 23.52 |
| | $CHCl_3$ | 1000 | 1117 | -- | -- | -- |
| | THF | 952 | 1125 | -- | -- | -- |
| | Pdots | 968 | 1105 | $0.011 \times 10^5$ | 0.448±0.08 | 4.93 |
| **J-X-J** | $CH_2Cl_2$ | 1014 | 1210 | $2.190 \times 10^5$ | 0.029 | 63.51 |
| | $CHCl_3$ | 1028 | 1180 | -- | -- | -- |
| | THF | 990 | 1240 | -- | -- | -- |
| | Pdots | 1014 | 1220 | $0.053 \times 10^5$ | 0.004±0.001 | 0.212 |

| | | | | | | |
|---|---|---|---|---|---|---|
| | $CH_2Cl_2$ | 1016 | 1220 | $1.990 \times 10^5$ | 0.025 | 49.75 |
| | $CHCl_3$ | 1045 | 1185 | -- | -- | -- |
| **J-X-BJ** | THF | 1005 | 1250 | -- | -- | -- |
| | Pdots | 1017 | 1220 | $0.047 \times 10^5$ | 0.008±0.002 | 0.376 |
| | $CH_2Cl_2$ | 1025 | 1225 | $2.070 \times 10^5$ | 0.024 | 49.68 |
| | $CHCl_3$ | 1040 | 1183 | -- | -- | -- |
| **BJ-X-BJ** | THF | 1000 | 1246 | -- | -- | -- |
| | Pdots | 1025 | 1240 | $0.044 \times 10^5$ | 0.011±0.001 | 0.484 |
| | $CH_2Cl_2$ | 1195 | 1536 | $0.420 \times 10^5$ | 0.007 | 1.680 |
| | $CHCl_3$ | 1189 | 1510 | -- | -- | -- |
| **T-Si-T** | THF | 1152 | 1524 | -- | -- | -- |
| | Pdots | 1190 | 1290 | $0.110 \times 10^5$ | 0.050±0.005 | 5.577 |
| | $CH_2Cl_2$ | 1230 | 1520 | $0.910 \times 10^5$ | 0.009 | 4.823 |
| | $CHCl_3$ | 1262 | 1467 | -- | -- | -- |
| **T-Si-BJ** | THF | 1203 | 1553 | -- | -- | -- |
| | Pdots | 1220 | 1320 | $0.290 \times 10^5$ | 0.048±0.003 | 14.18 |
| | $CH_2Cl_2$ | 1312 | 1505 | $1.390 \times 10^5$ | 0.012 | 10.00 |
| | $CHCl_3$ | 1326 | 1500 | -- | -- | -- |
| **BJ-Si-BJ** | THF | 1302 | 1522 | -- | -- | -- |
| | Pdots | 1360 | 1555 | $0.310 \times 10^5$ | 0.001±0.0002 | 0.341 |

**Table S3.** Summary of lifetime measurements of T-X-T in $CH_2Cl_2$ solution and aggregate states.

| State | Lifetime (ps) | $k_r$ ($s^{-1}$) | k□r ($s^{-1}$) |
|---|---|---|---|
| $CH_2Cl_2$ | 1.48 | $8.1 \times 10^7$ | $6.8 \times 10^{11}$ |
| * | 1.6±0.3 | $6.32\times10^7$ ~$9.23\times10^7$ | $5.26\times10^{11}$ ~ $7.29\times10^{11}$ |
| Pdots (TTQ) | 8.91 | $2.8 \times 10^9$ | $1.1 \times 10^{11}$ |
| * | 8.80~9.34 | $2.70\times10^9$ ~$2.87\times10^9$ | $1.04\times10^{11}$ ~$1.11\times10^{11}$ |
| Short-range packing (Pdots) | 2.08 | $1.2 \times 10^{10}$ | $4.7 \times 10^{11}$ |
| * (Fast component) | 2.1±0.1 | $1.15\times10^{10}$~$1.26\times10^{10}$ | $4.43\times10^{11}$~ $4.87\times10^{11}$ |
| Long-range packing (Pdots) | 25.11 | $1.0 \times 10^9$ | $3.9 \times 10^{10}$ |
| * (Slow component) | 25.3±0.7 | $9.70\times10^8$ ~$1.03\times10^9$ | $3.75\times10^{10}$ ~$3.96\times10^{10}$ |

| | | | |
|---|---|---|---|
| Pdots (PMMA) | < 1 | - | - |

*using the Levenberg-Marquardt algorithm.

**Table S4**. Reported Photophysical Data for: Symmetric AIE and Symmetric ACQ measured in $H_2O$ (Pdots assembled with Pttc-TTQ/mPEG-DSPE).[4, 8]

Reported Photophysical Data for: Symmetric AIE and Symmetric ACQ (measured in $H_2O$)

| Symmetric AIE | Peak $\lambda_{abs}$ | Peak $\lambda_{em}$ | ϕ > 1500 nm | Imaging |
|---|---|---|---|---|
| 2TT-oC6B | 730 nm | 1034 nm | 0.009% | 1500-1700 nm |
| 2TToC26B | 730 nm | 1031 nm | 0.012% | 1500-1700 nm |
| 2TT-oC610B | 730 nm | 1029 nm | 0.011% | 1500-1700 nm |
| TT2-oCB | 752 nm | 1020 nm | 0.003% | 1500-1700 nm |
| TT3-oCB | 784 nm | 1062 nm | 0.003% | 1500-1700 nm |
| OTPA-BBT | 700 nm | 1000 nm | 0.012% | 1500-1700 nm |
| HQL2 | 720 nm | 993 nm | 0.0002% | 1500-1700 nm |
| HL3 | 750 nm | 1050 nm | 0.005% | 1500-1700 nm |

| Symmetric ACQ | Peak $\lambda_{abs}$ | Peak $\lambda_{em}$ | ϕ > 1500 nm | Imaging |
|---|---|---|---|---|
| SiRos 1700 | 1053 nm | 1500 nm | 0.0007% | - |

Our work- Photophysical Data for: Asymmetric AIE+ACQ (measured in $H_2O$)

| Asymmetric AIE | Peak $\lambda_{abs}$ | Peak $\lambda_{em}$ | ϕ > 1500 nm | Imaging |
|---|---|---|---|---|
| T-X-J | 960 nm | 1102 nm | 0.008% | - |
| T-X-BJ | 968 nm | 1105 nm | 0.009% | 1500-1700 nm |
| T-Si-BJ | 1220 nm | 1320 nm | 0.028% | 1500-1700 nm |

**Table S5:** The dihedral angle of linker (C=C) relative to chromophore (α1 and α2) or xanthene core (β1 and β2) of T-X-T monomer, and low-energy TXT dimer and T-X-T.TTQ complex using B3LYP-D3 and M06-2X optimized methods with 6-31G* basis sets, and BSSE and ZPE corrected binding energy (BE(0 K), in kcal/mol) of T-X-T dimer and T-X-T.TTQ complex at the M06-2X/6-31+G* level using different optimized methods.

| | | α1 | β1 | β2 | β2 | BE(0 K) |
|---|---|---|---|---|---|---|
| B3LYP-D3 | monomer | 1.5 | 0.2 | -0.2 | -1.5 | |
| | dimer | -14.1(-9.1) | -11.3(-1.4) | 4.8(-3.7) | -2.5(-6.1) | -3.81 |
| | T-X-T.TTQ | -23.4 | -19.9 | -0.6 | 0.9 | -25.26 |
| M06-2X | monomer | 1.7 | 0.2 | -0.2 | -1.7 | |
| | dimer | -14.5(-9.6) | -11.3(-3.0) | 4.3(-3.5) | 1.3(-6.2) | -5.32 |

| | | | | | |
|---|---|---|---|---|---|
| T-X-T.TTQ | -20.0 | -24.2 | 2.0 | 2.6 | -26.00 |

**Table S6:** Photophysical Properties of experimental results and the calculated vertical excitation data ($E_{abs}$ in eV and $\lambda_{abs}$ in nm) at the SOS-CIS(D)($CH_2Cl_2$, CPCM)/VDZ of symmetric and asymmetric xanthenes in $CH_2Cl_2$.

| | Exp. | CIS($CH_2Cl_2$, CPCM) | | SOS-CIS(D)($CH_2Cl_2$, CPCM) | | Difference between CIS and SOS-CIS(D) |
|---|---|---|---|---|---|---|
| dye | $\lambda_{max}^{abs}$ | $E_{abs}$ | $\lambda_{abs}$ | $E_{abs}$ | $\lambda_{abs}$ | eV |
| T-X-T | 884 | 2.792 | 444 | 1.741 | 742 | 1.051 |
| T-X-J | 865 | 2.705 | 458 | 1.551 | 800 | 1.154 |
| T-X-BJ | 970 | 2.694 | 460 | 1.522 | 815 | 1.172 |
| J-X-J | 1014 | 2.649 | 468 | 1.452 | 854 | 1.197 |
| J-X-BJ | 1016 | 2.641 | 469 | 1.434 | 865 | 1.207 |
| BJ-X-BJ | 1025 | 2.634 | 471 | 1.418 | 875 | 1.216 |
| T-Si-T | 1195 | 2.326 | 533 | 1.136 | 1091 | 1.190 |
| T-Si-BJ | 1230 | 2.241 | 553 | 0.963 | 1288 | 1.278 |
| BJ-X-BJ | 1312 | 2.185 | 568 | 0.856 | 1449 | 1.329 |

**Table S7**. Photophysical properties of experimental results and computational calculation of symmetric and asymmetric xanthenes in this study. ($E_{abs}$ is $E_{max}^{abs}$, $E_{em}$ is $E_{max}^{em}$, unit is $cm^{-1}$, A = Einstein's coefficient, A = $(E\_em^3)/E\_abs$, T=$(E\_em^3)/E\_abs$ ×OS×$n^2$×0.6671).

| DCM | $\lambda_{max}^{abs}$ | $\lambda_{max}^{em}$ | $E_{max}^{abs}$ | $E_{max}^{em}$ | A | T/$10^6$ | Φ(%) | | $\lambda_{max}^{abs}$ | $\lambda_{max}^{em}$ | $E_{max}^{abs}$ | $E_{max}^{em}$ | A | T/$10^6$ | Φ(%) |
|---|---|---|---|---|---|---|---|---|---|---|---|---|---|---|---|
| T-X-T | 884 | 1220 | 11312 | 8197 | 48682489 | 65.9 | 0.012 | T-X-T.TTQ | 826 | 960 | 12107 | 10417 | 93361183 | 141.6 | 2.523 |
| T-X-J | 965 | 1192 | 10363 | 8389 | 56976865 | 77.1 | 0.017 | T-X-J.TTQ | 960 | 1102 | 10417 | 9074 | 71734231 | 108.8 | 0.333 |
| T-X-BJ | 970 | 1200 | 10309 | 8333 | 56134259 | 75.9 | 0.014 | T-X-BJ.TTQ | 968 | 1105 | 10331 | 9050 | 71744485 | 108.8 | 0.448 |
| J-X-J | 1014 | 1210 | 9862 | 8264 | 57237657 | 77.4 | 0.029 | J-X-J.TTQ | 1014 | 1220 | 9862 | 8197 | 55841678 | 84.7 | 0.004 |
| J-X-BJ | 1016 | 1220 | 9843 | 8197 | 55951820 | 75.7 | 0.025 | J-X-BJ.TTQ | 1017 | 1220 | 9833 | 8197 | 56006890 | 84.9 | 0.008 |
| BJ-X-BJ | 1025 | 1225 | 9756 | 8163 | 55759080 | 75.4 | 0.024 | BJ-X-BJ.TTQ | 1025 | 1240 | 9756 | 8065 | 53759944 | 81.5 | 0.011 |
| T-Si-T | 1195 | 1536 | 8368 | 6510 | 32975718 | 44.6 | 0.007 | T-Si-T.TTQ | 1190 | 1290 | 8403 | 7752 | 55434206 | 84.1 | 0.050 |
| T-Si-BJ | 1230 | 1520 | 8130 | 6579 | 35024694 | 47.4 | 0.009 | T-Si-BJ.TTQ | 1220 | 1320 | 8197 | 7576 | 53044216 | 80.4 | 0.048 |
| BJ-Si-BJ | 1312 | 1505 | 7622 | 6645 | 38487911 | 52.1 | 0.012 | BJ-Si-BJ.TTQ | 1360 | 1555 | 7353 | 6431 | 36169935 | 54.8 | 0.001 |

# Appendix ($^1$H-NMR, $^{13}$C-NMR, HR-Mass, and ESI-HRMS)

$^1$H NMR of **Compound 1-I**

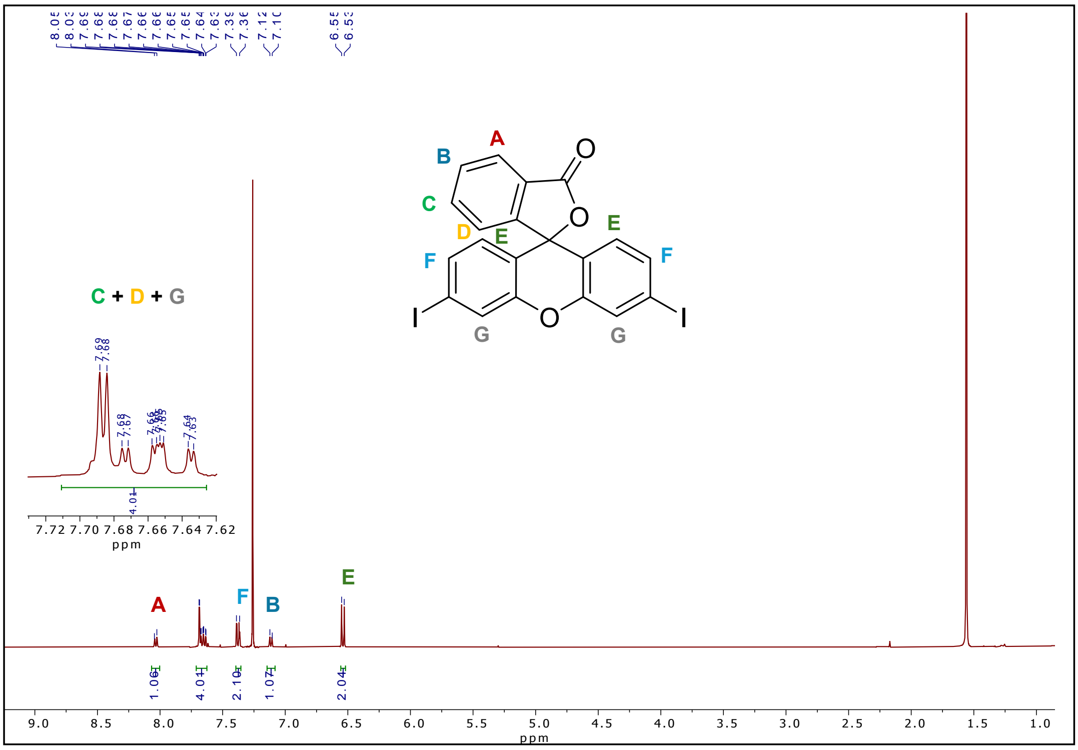


$^1$H NMR of **Compound 1-Br**

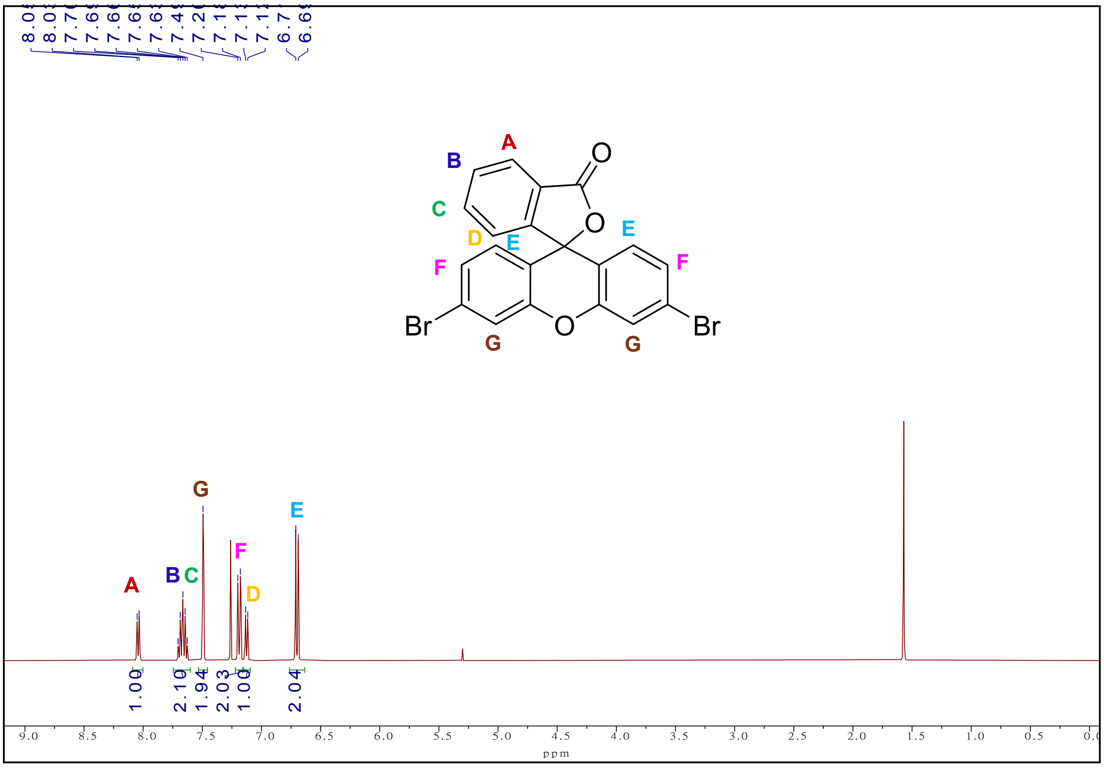

$^{1}$H NMR of **Compound 1-i**

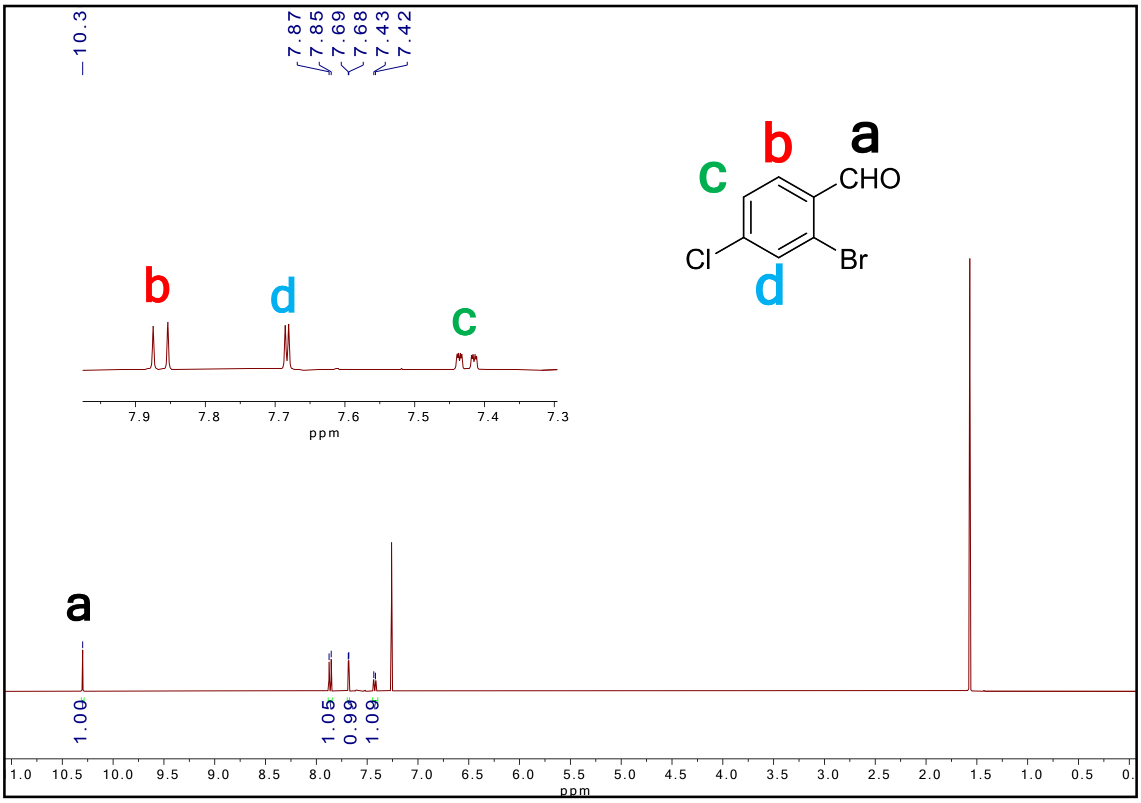


$^{1}$H NMR of **Compound 1-ii**

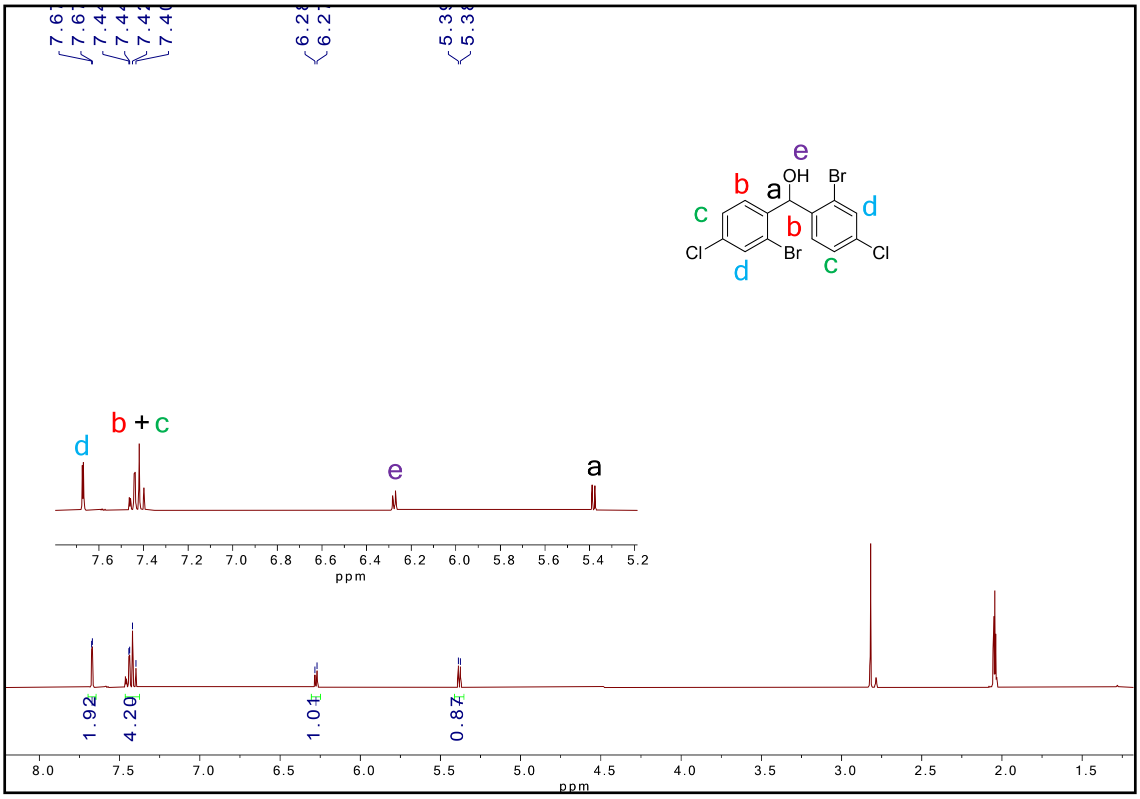

$^{1}$H NMR of **Compound 1-iii**

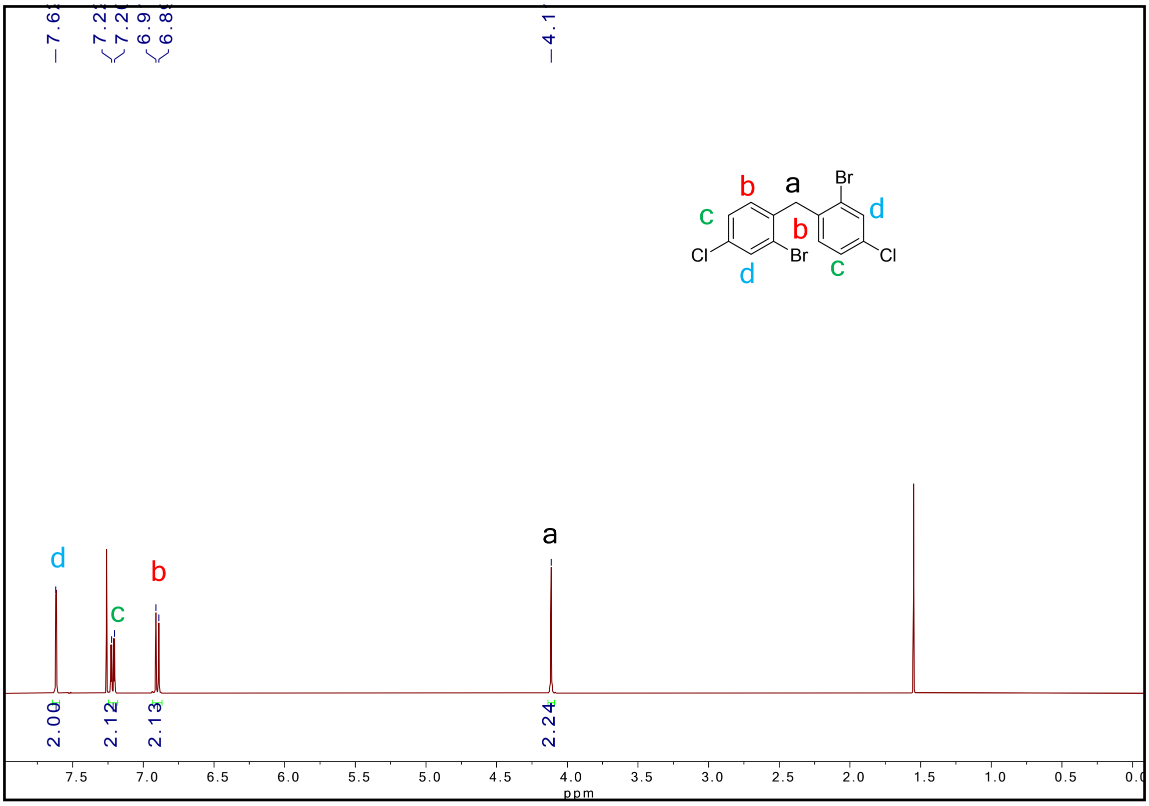


$^{1}$H NMR of **Compound 2**

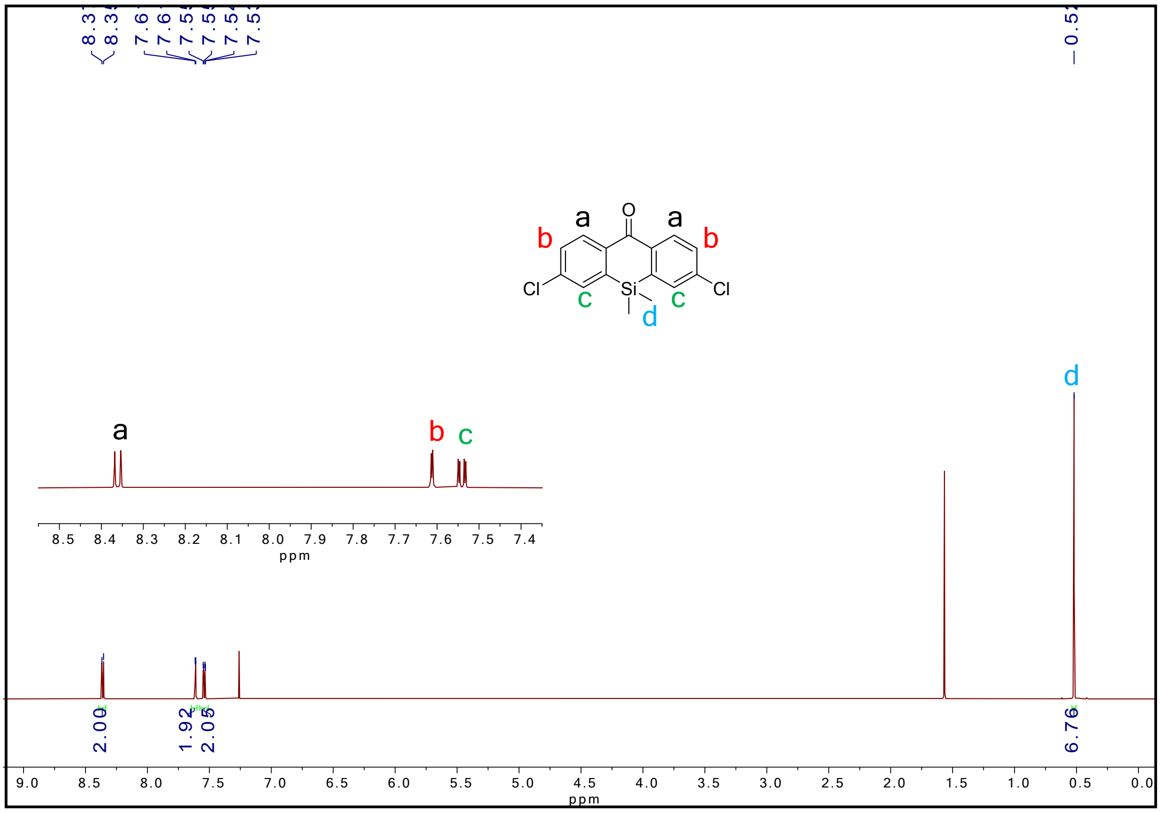

$^{1}$H NMR of **Compound 3-i**

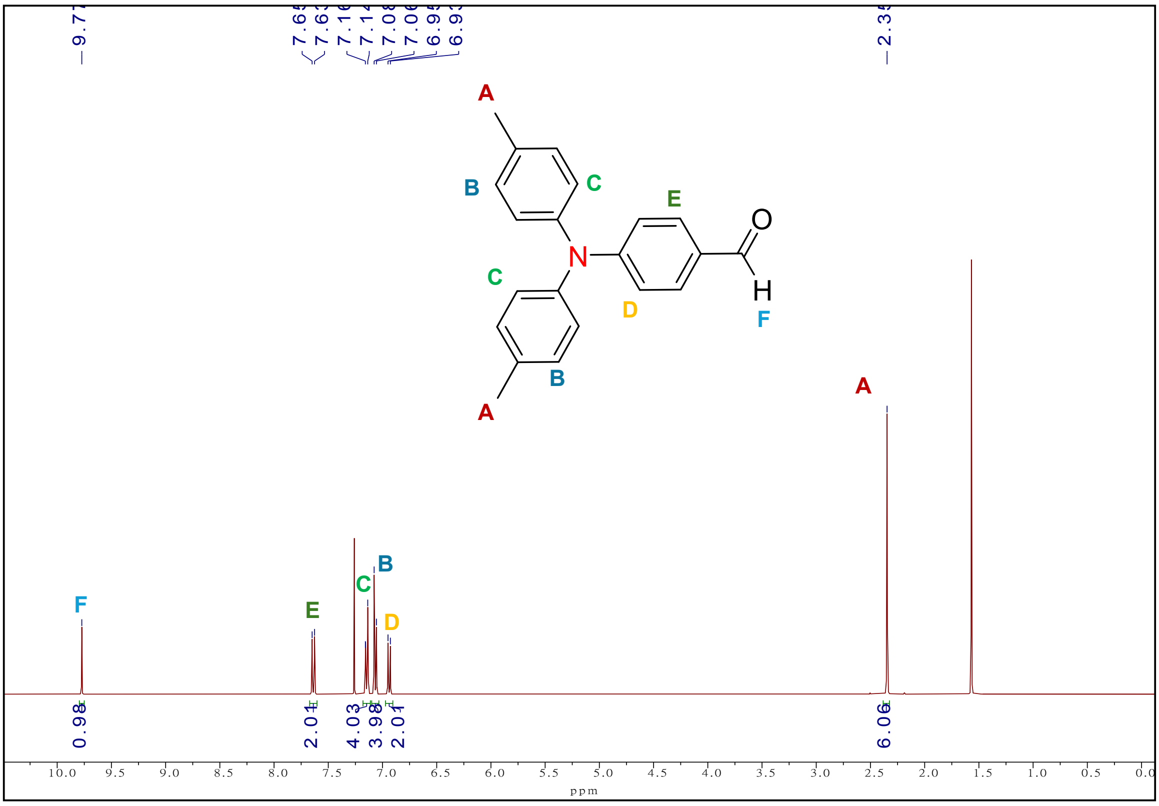


$^{1}$H NMR of **Compound 3-ii**

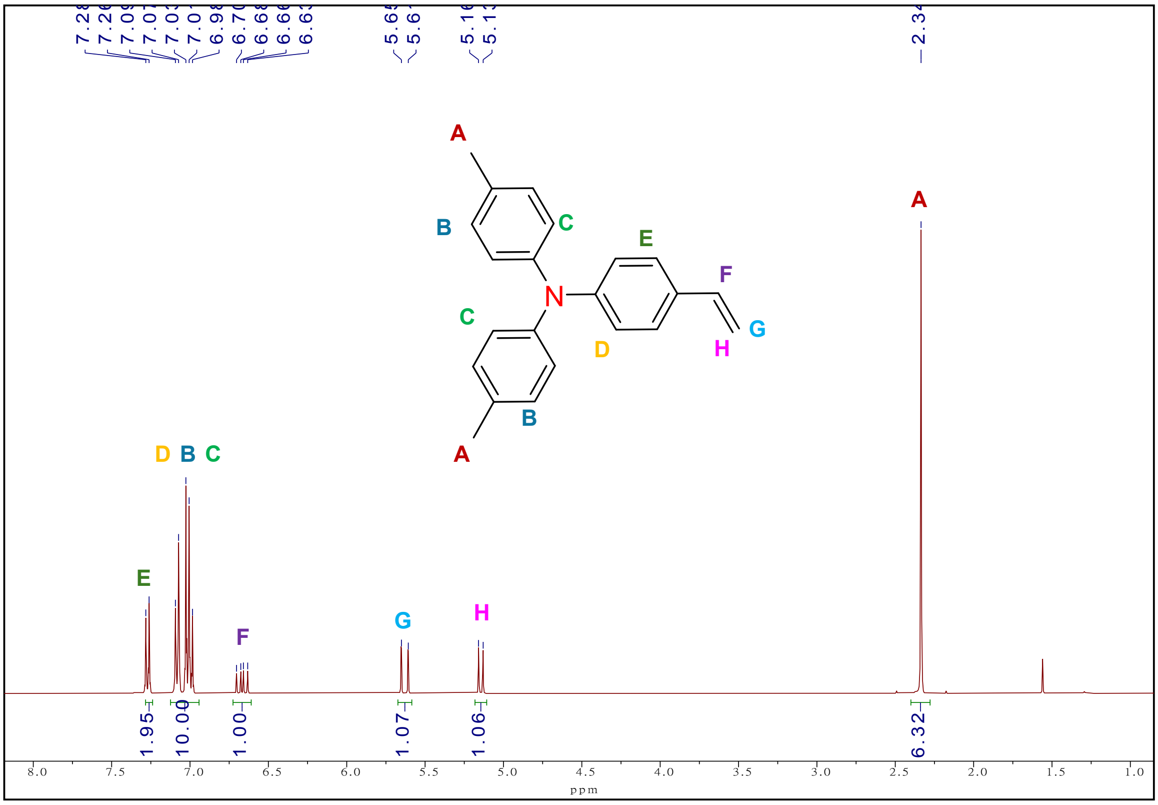

$^{1}$H NMR of **Compound 4-i**

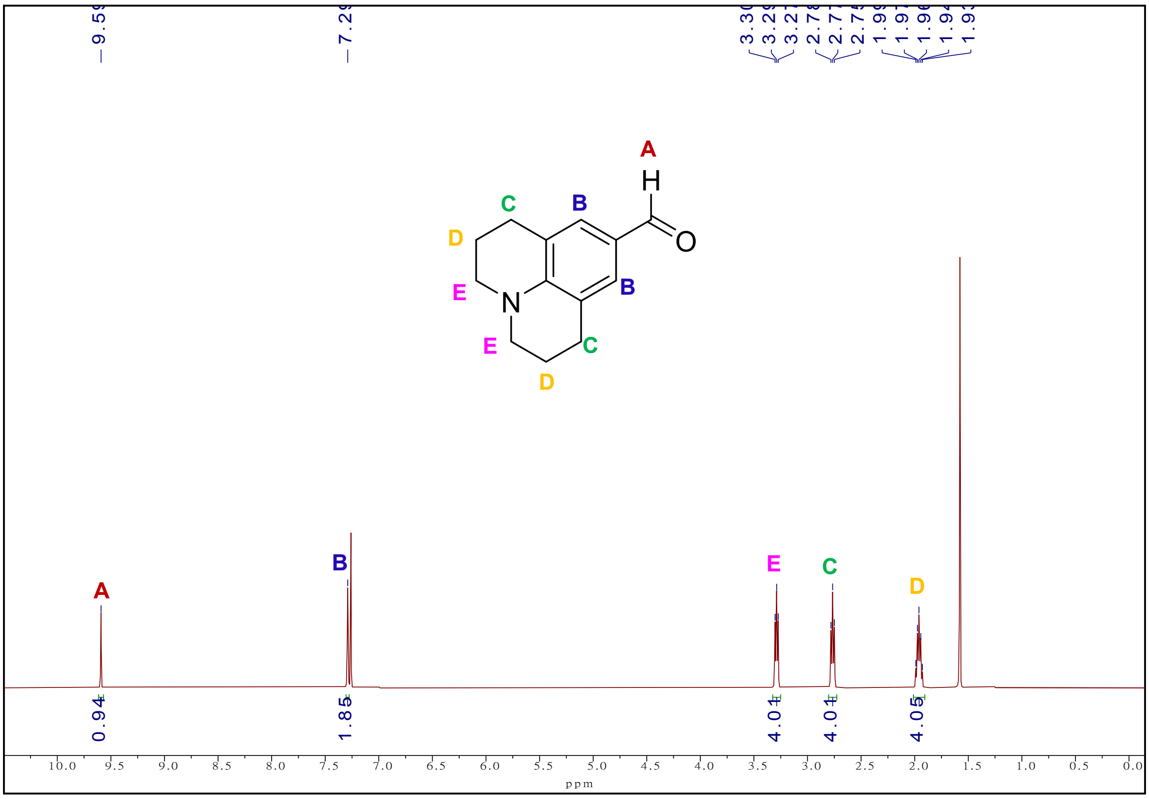

$^{1}$H NMR of **Compound 4-ii**

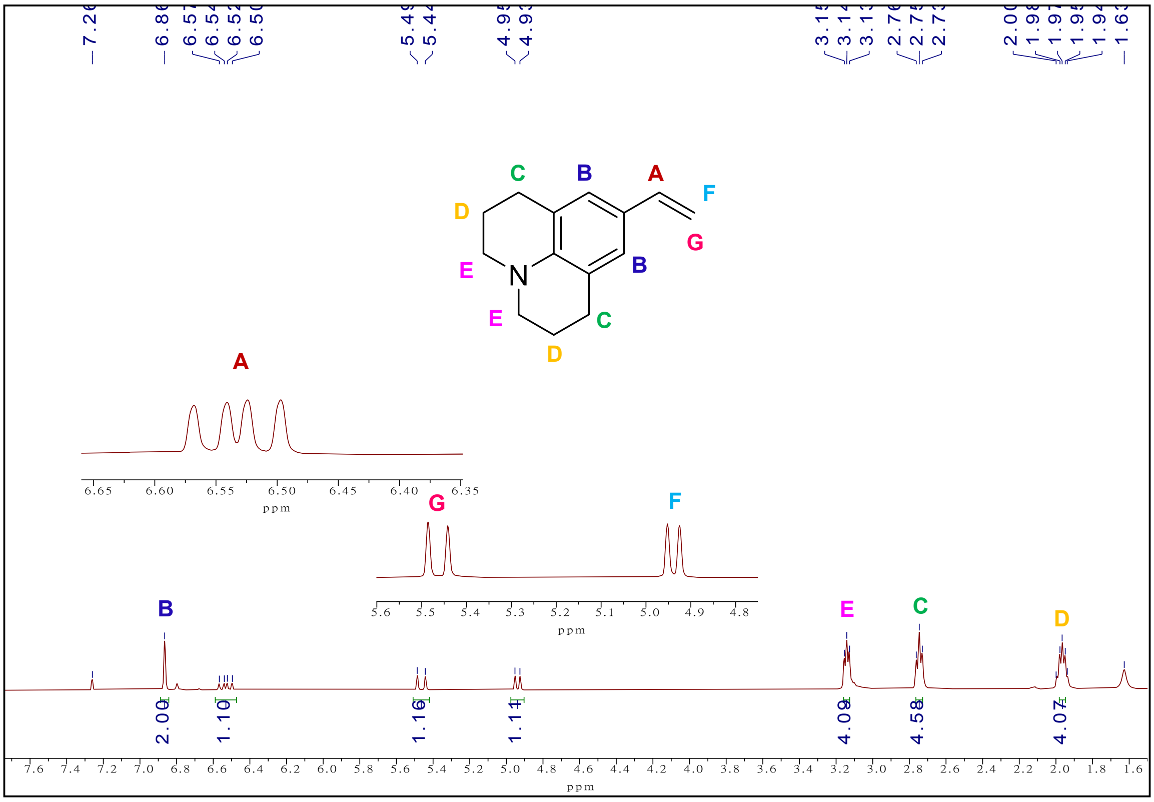

$^{1}$H NMR of **Compound 5'**

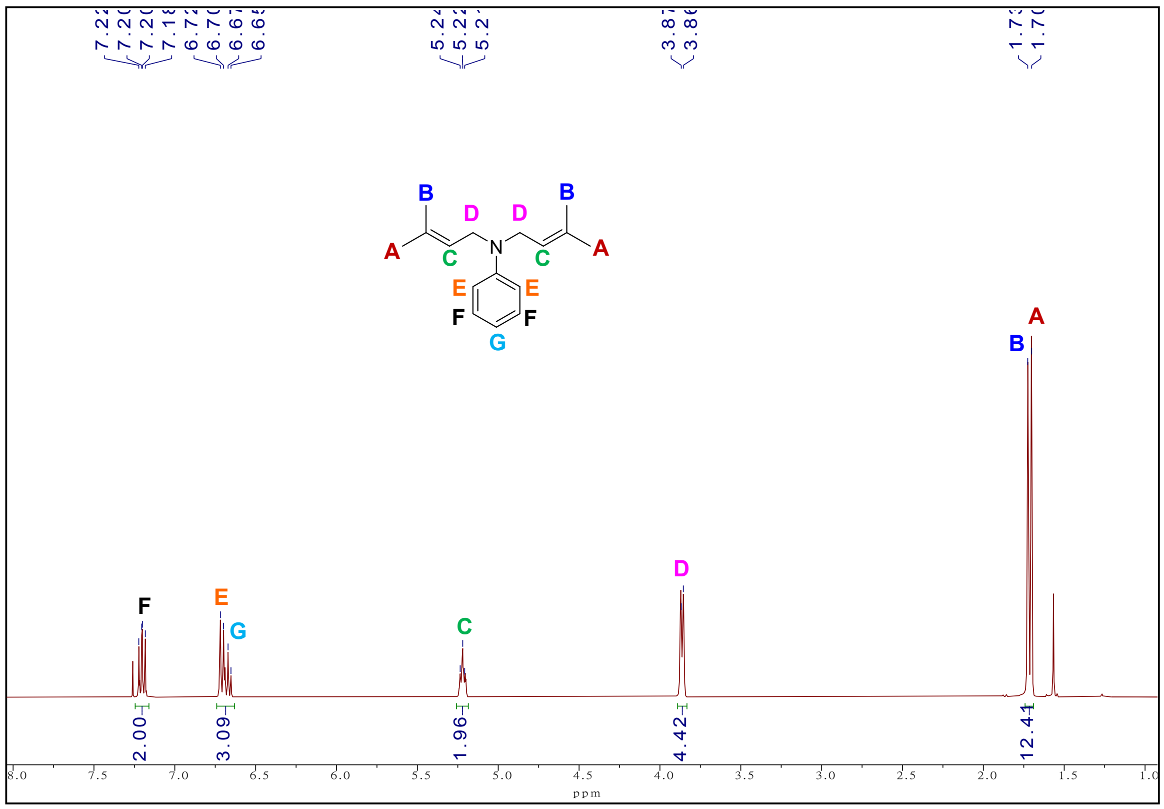

$^{1}$H NMR of **Compound 5**

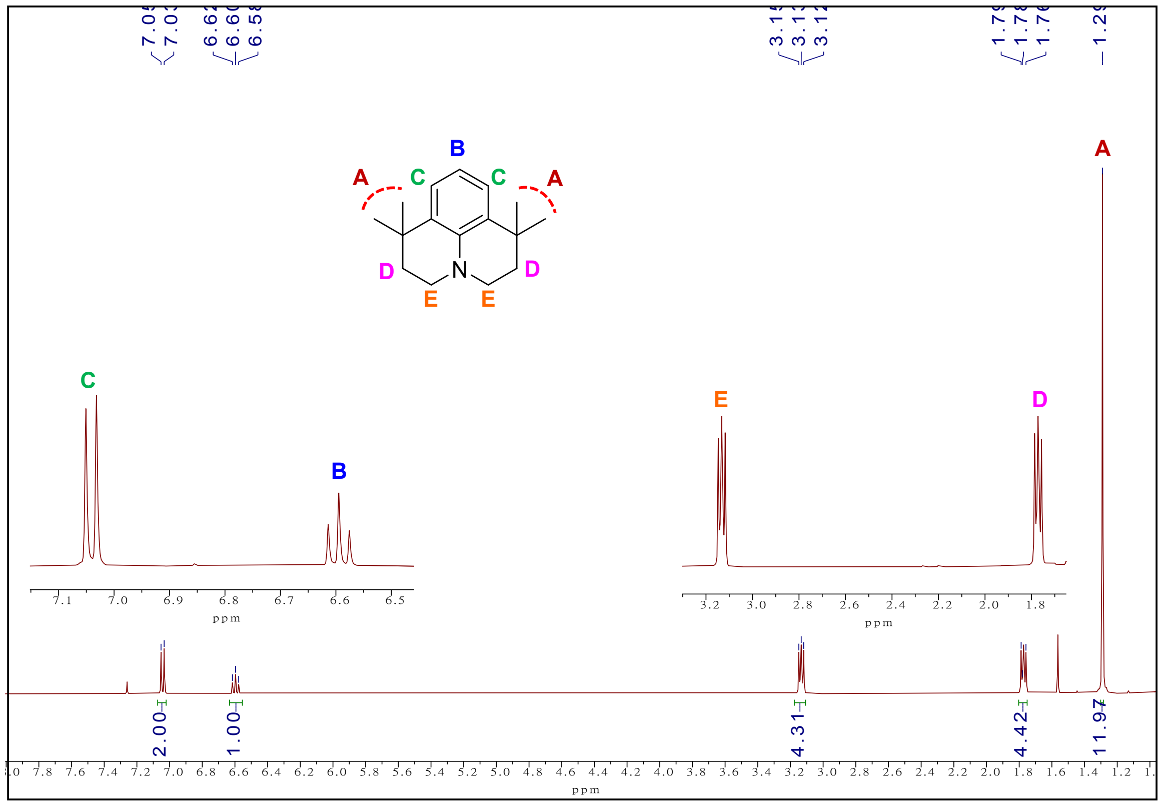

$^{1}$H NMR of **Compound 5-i**

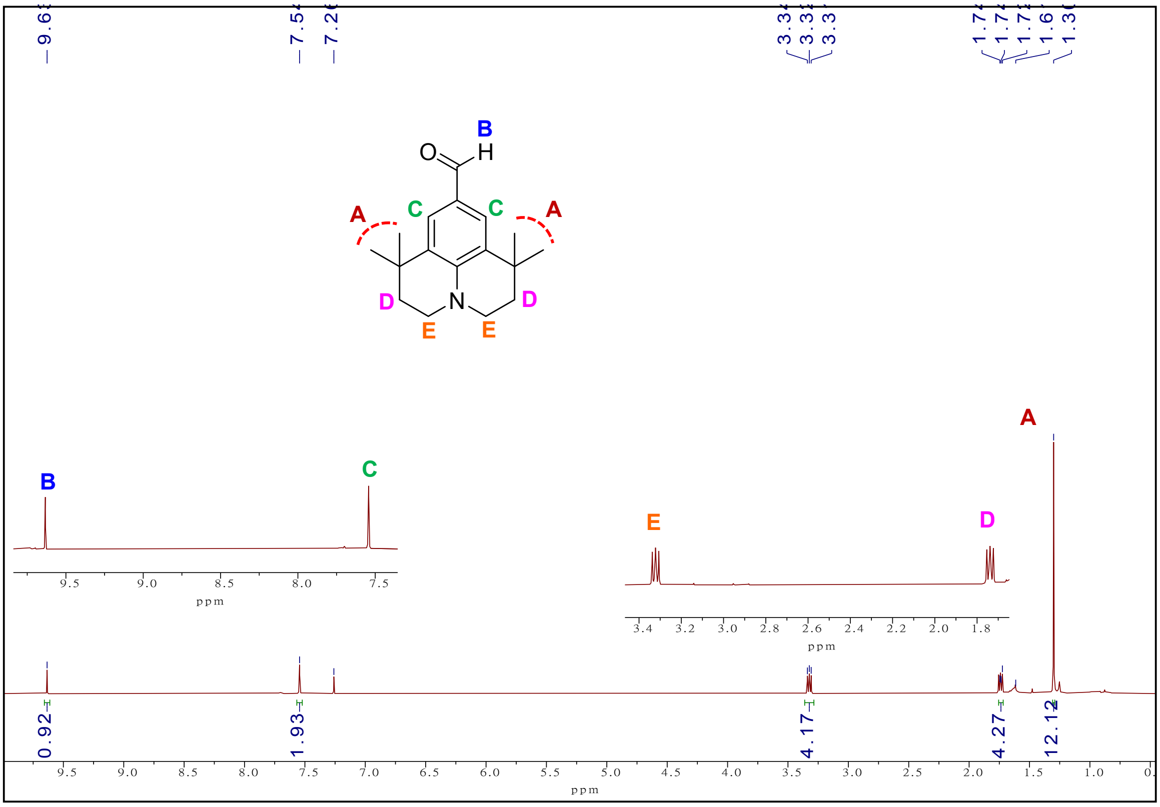

$^{1}$H NMR of **Compound 5-ii**

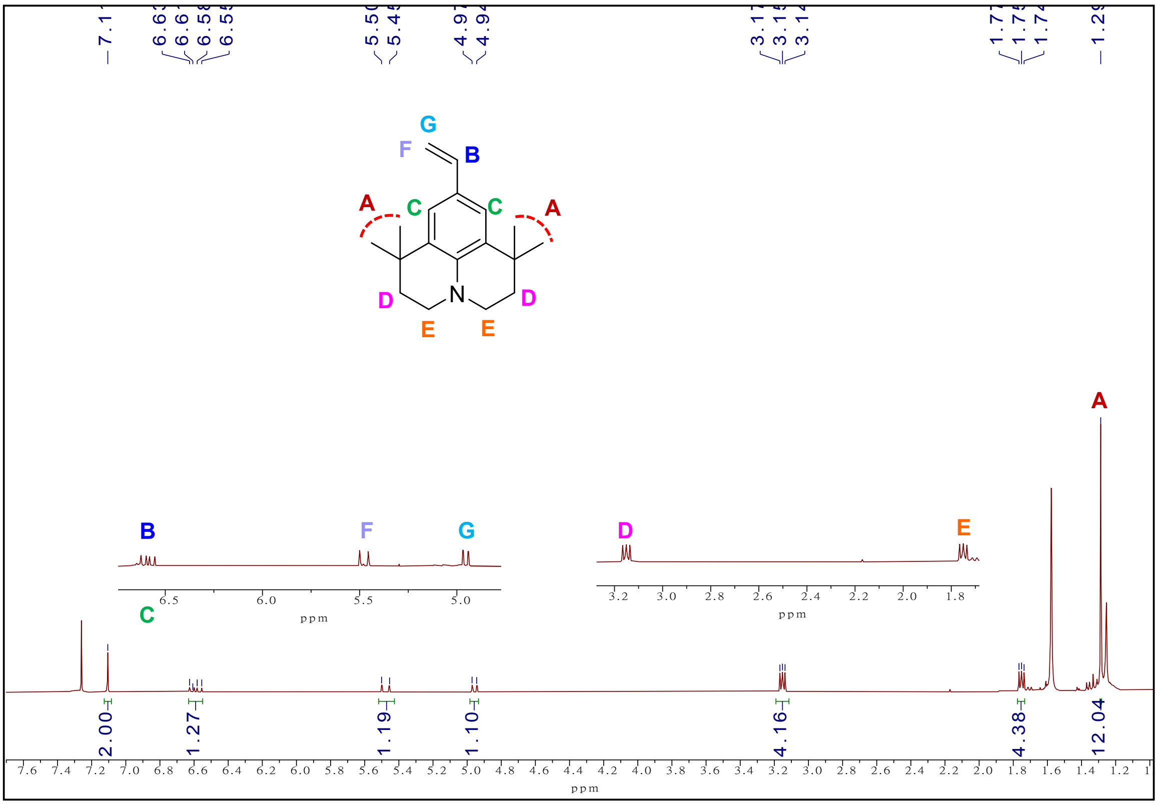

$^{1}$H NMR of **Compound 1a**

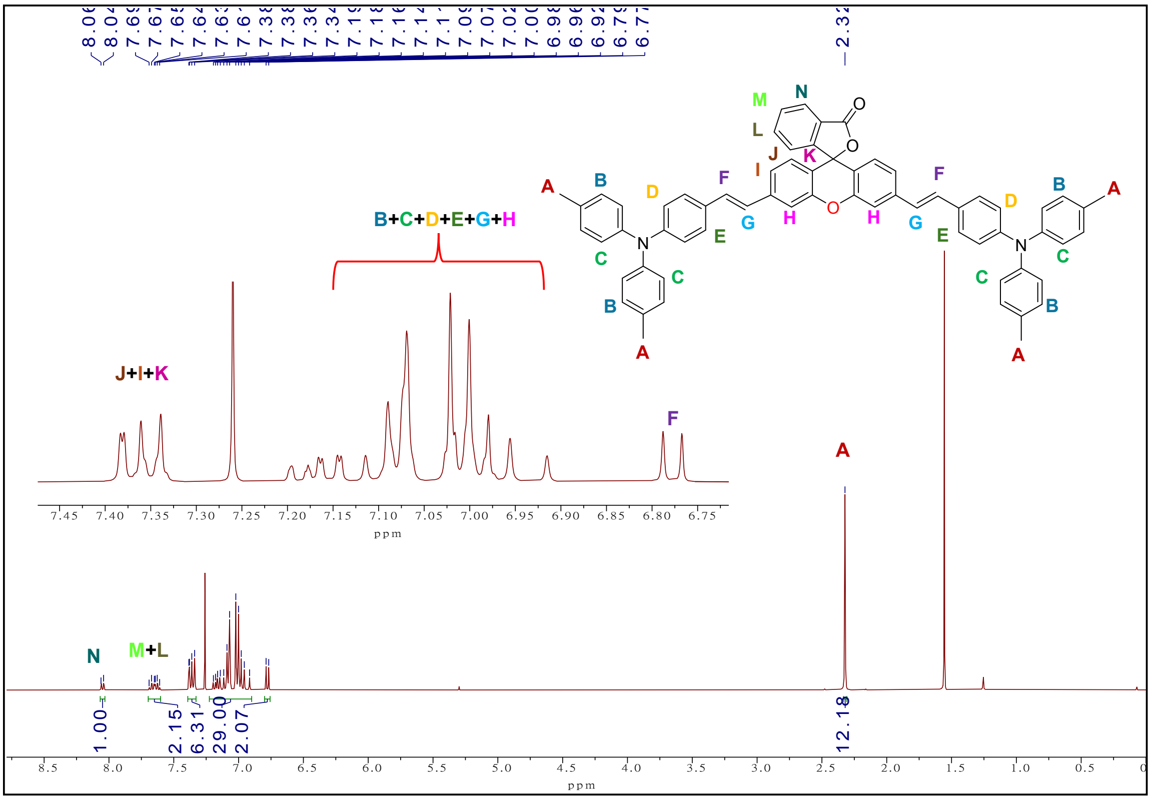

$^{13}$C NMR of **Compound 1a**

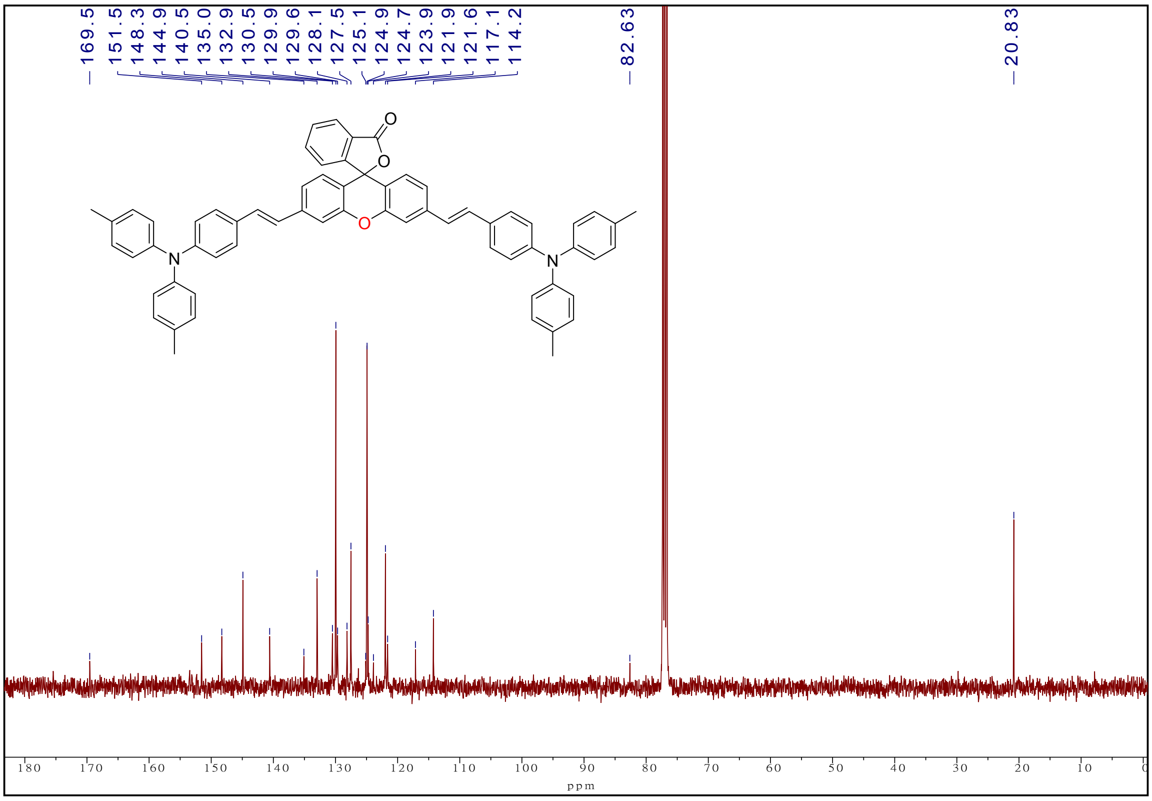

HRMS of **Compound 1a**

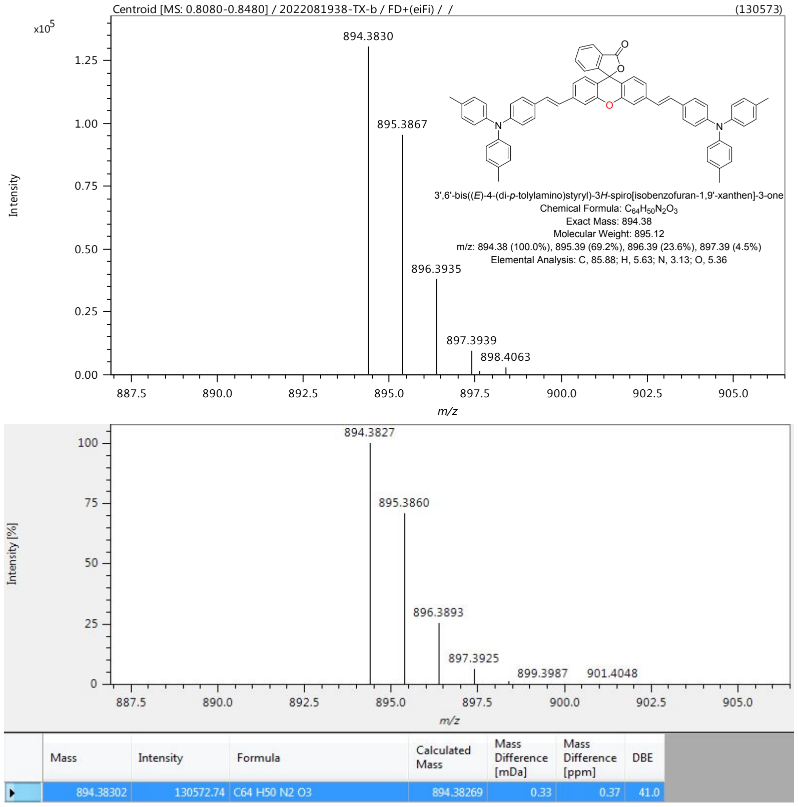


| Mass | Intensity | Formula | Calculated Mass | Mass Difference [mDa] | Mass Difference [ppm] | DBE |
|---|---|---|---|---|---|---|
| 894.38302 | 130572.74 | C64 H50 N2 O3 | 894.38269 | 0.33 | 0.37 | 41.0 |

¹H NMR of **Compound 1b**

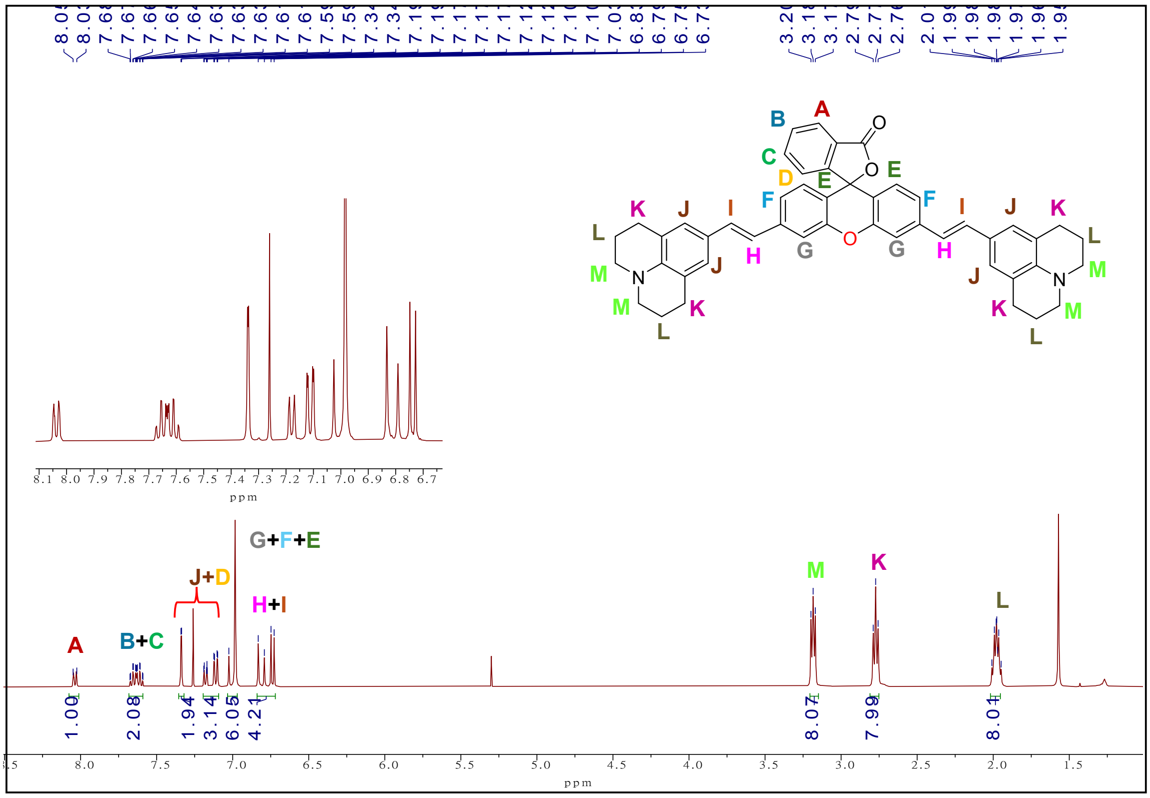


¹H NMR of **Compound 1c**

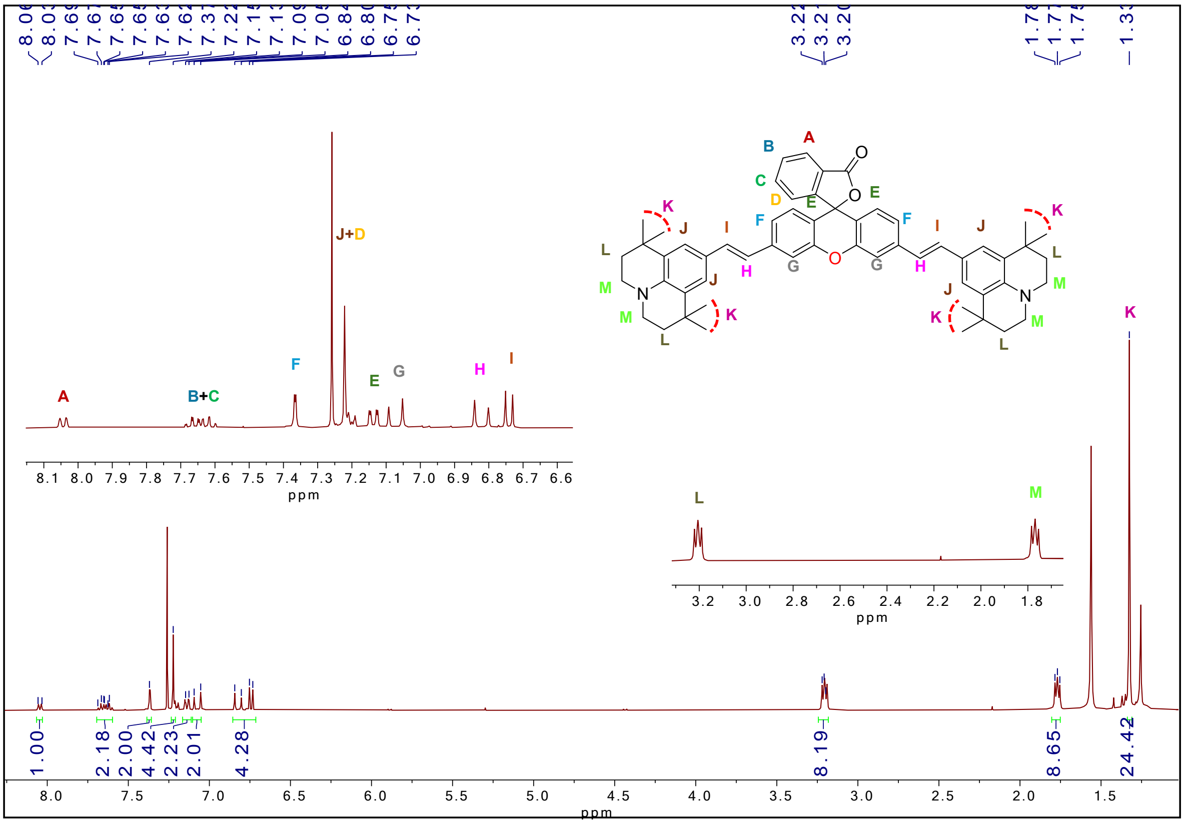

$^{13}$C NMR of **Compound 1c**

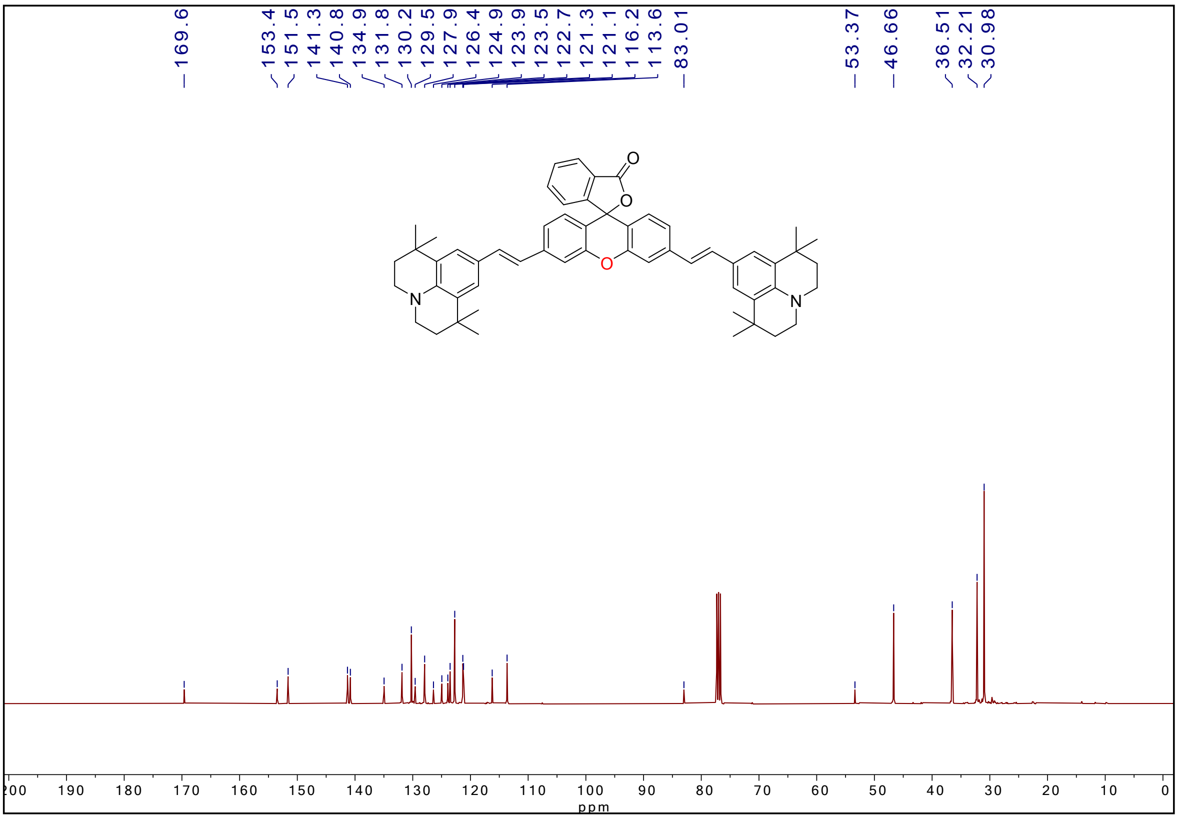

HRMS of **Compound 1c**

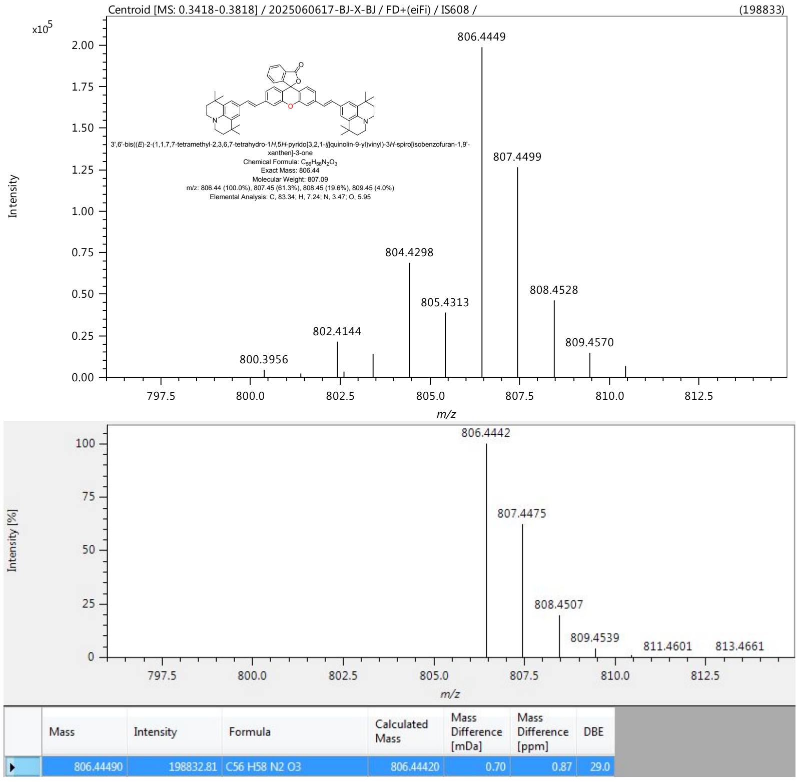


| Mass | Intensity | Formula | Calculated Mass | Mass Difference [mDa] | Mass Difference [ppm] | DBE |
|---|---|---|---|---|---|---|
| 806.44490 | 198832.81 | C56 H58 N2 O3 | 806.44420 | 0.70 | 0.87 | 29.0 |

$^{1}$H NMR of **Compound 1d**

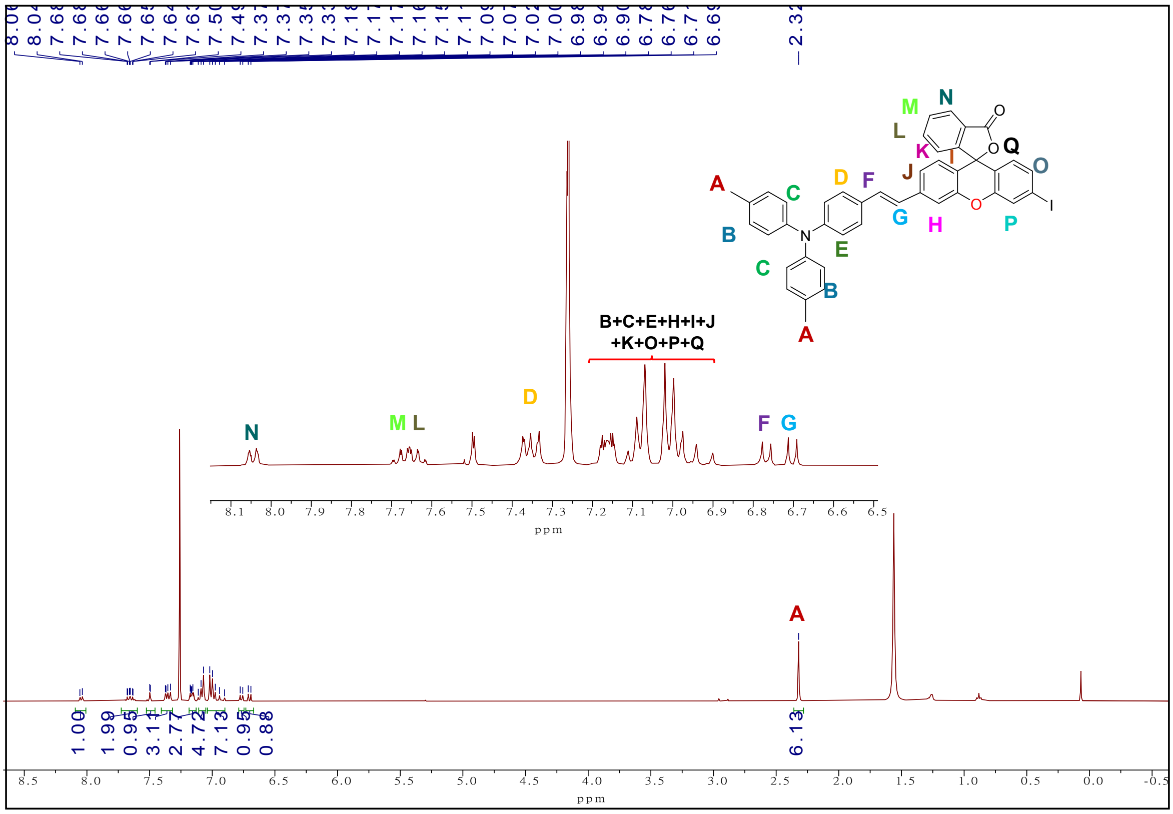


$^{13}$C NMR of **Compound 1d**

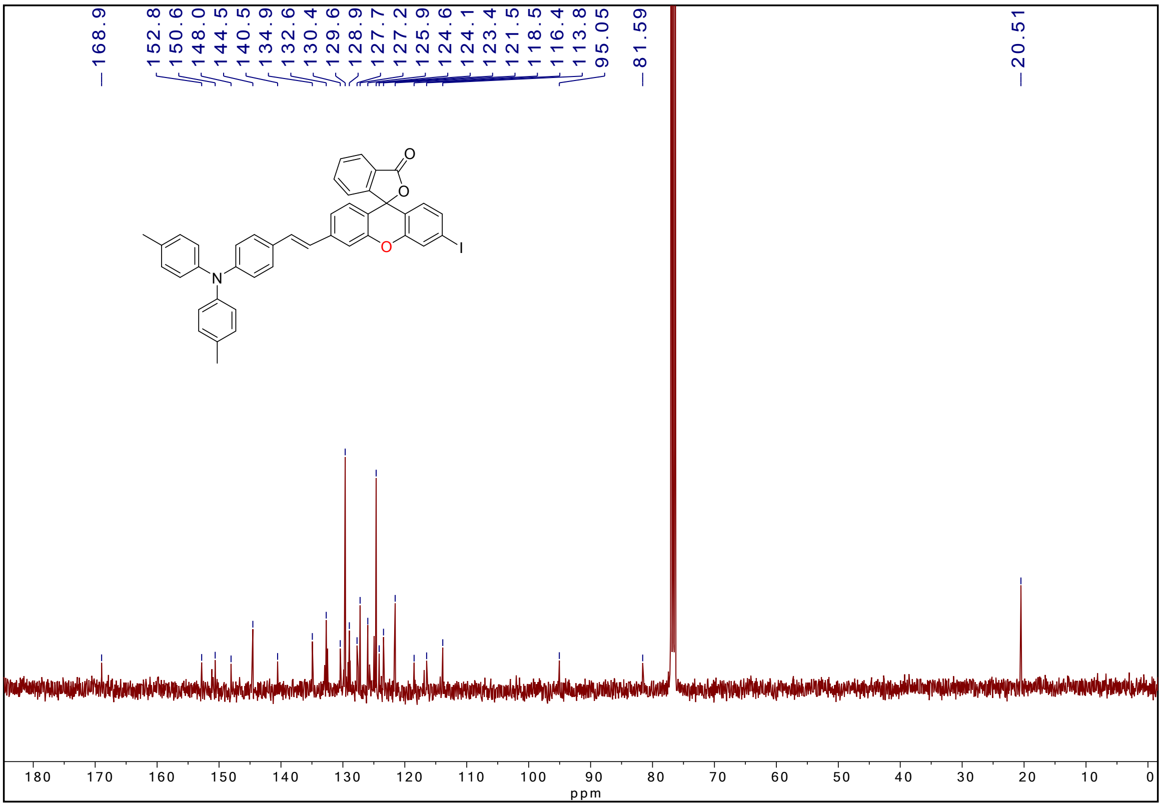


HRMS of **Compound 1d**

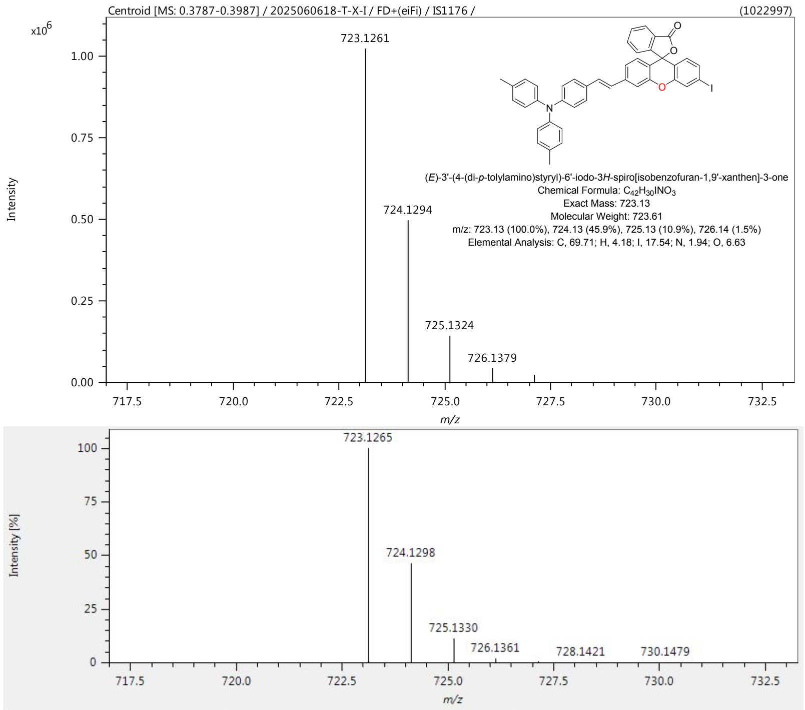


| | Mass | Intensity | Formula | Calculated Mass | Mass Difference [mDa] | Mass Difference [ppm] | DBE |
|---|---|---|---|---|---|---|---|
| ▸ | 723.12608 | 1022996.57 | C42 H30 N O3 I | 723.12649 | -0.41 | -0.57 | 28.0 |

$^{1}$H NMR of **Compound 1e**

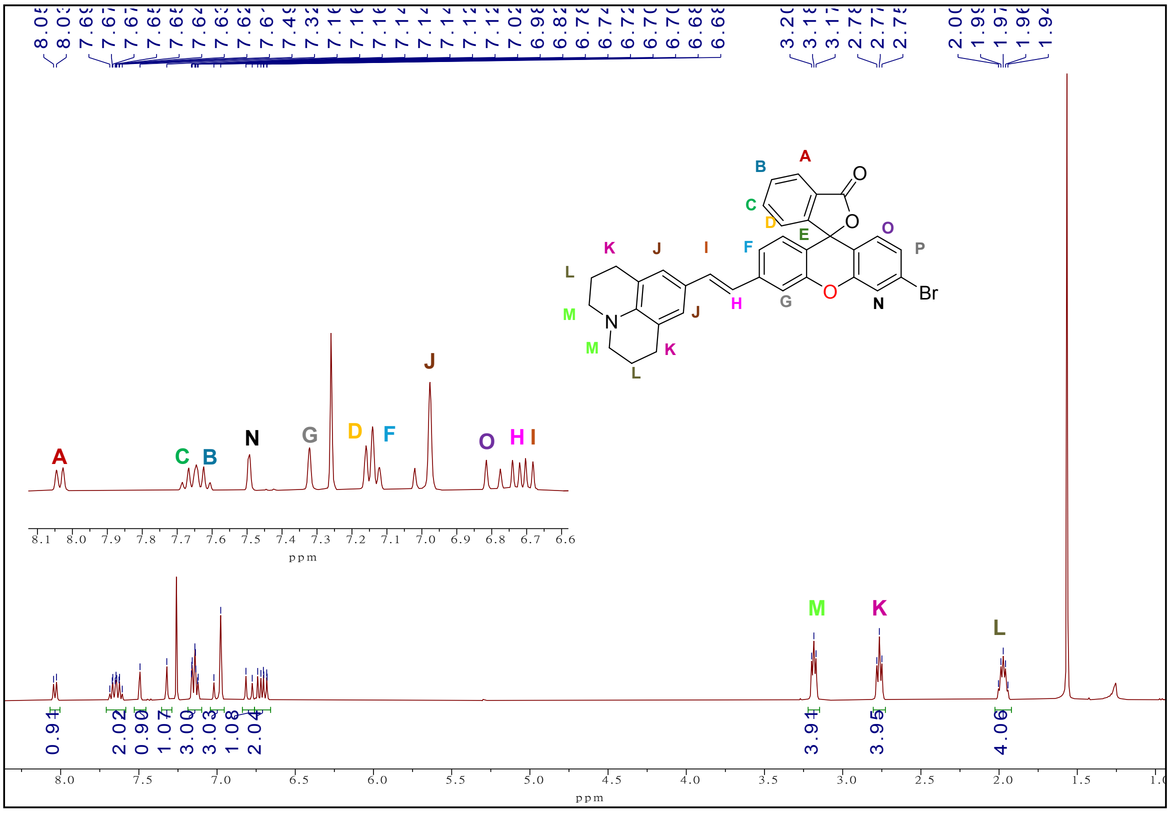


$^{13}$C NMR of **Compound 1e**

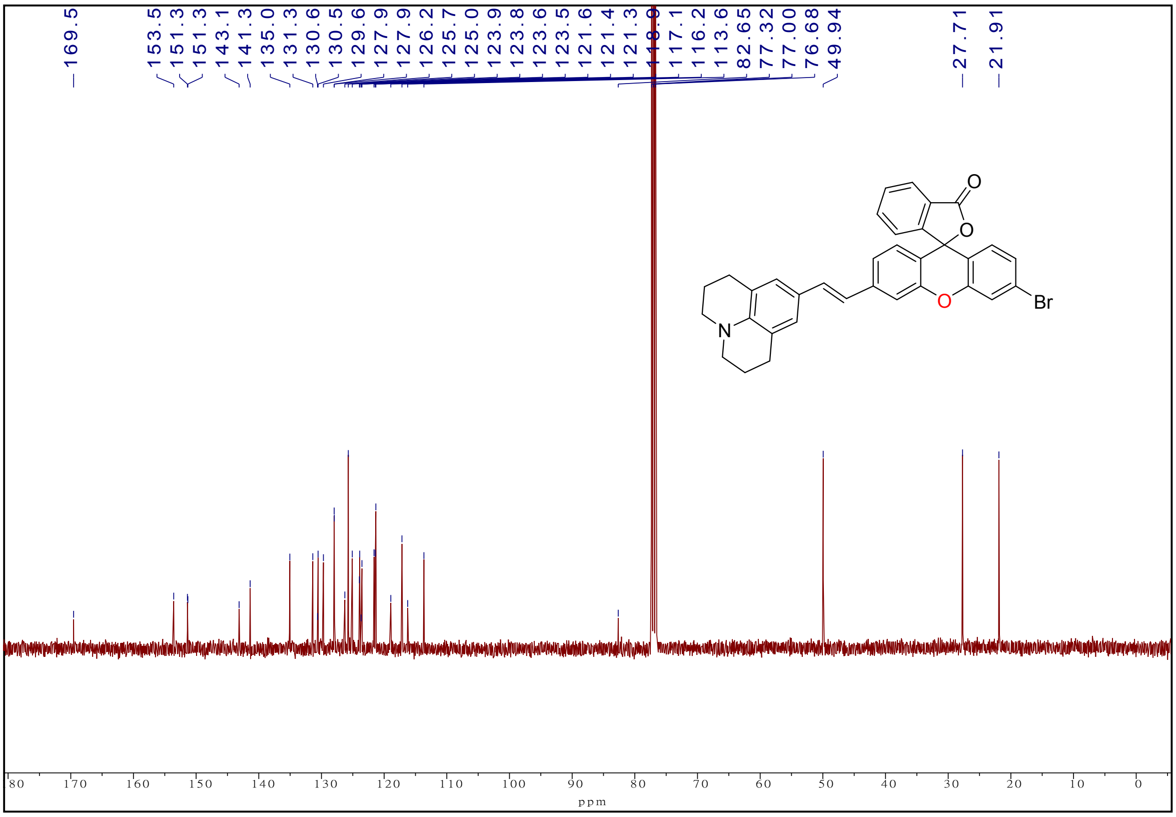


HRMS of **Compound 1e**

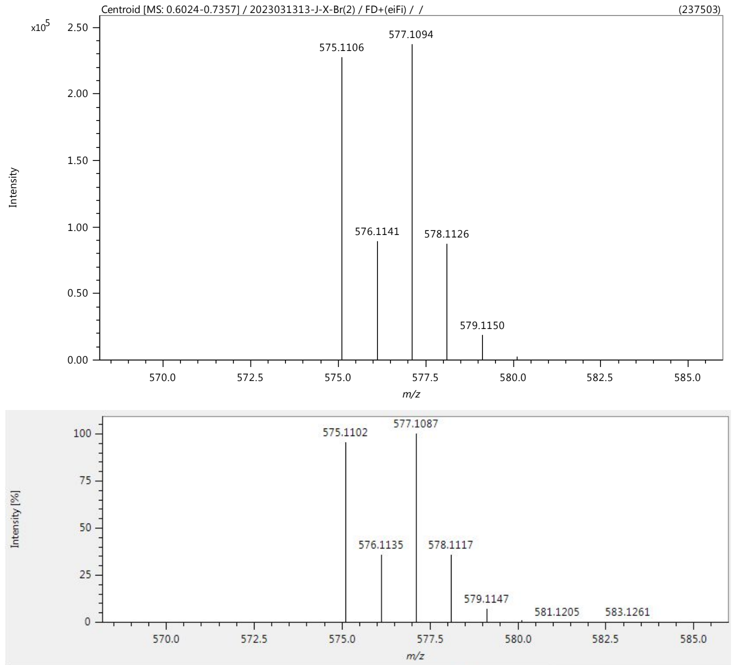


| | Mass | Intensity | Formula | Calculated Mass | Mass Difference [mDa] | Mass Difference [ppm] | DBE |
|---|---|---|---|---|---|---|---|
| ▸ | 575.11064 | 227659.92 | C34 H26 N O3 Br | 575.11015 | 0.49 | 0.85 | 22.0 |

$^{1}$H NMR of **Compound 1f**

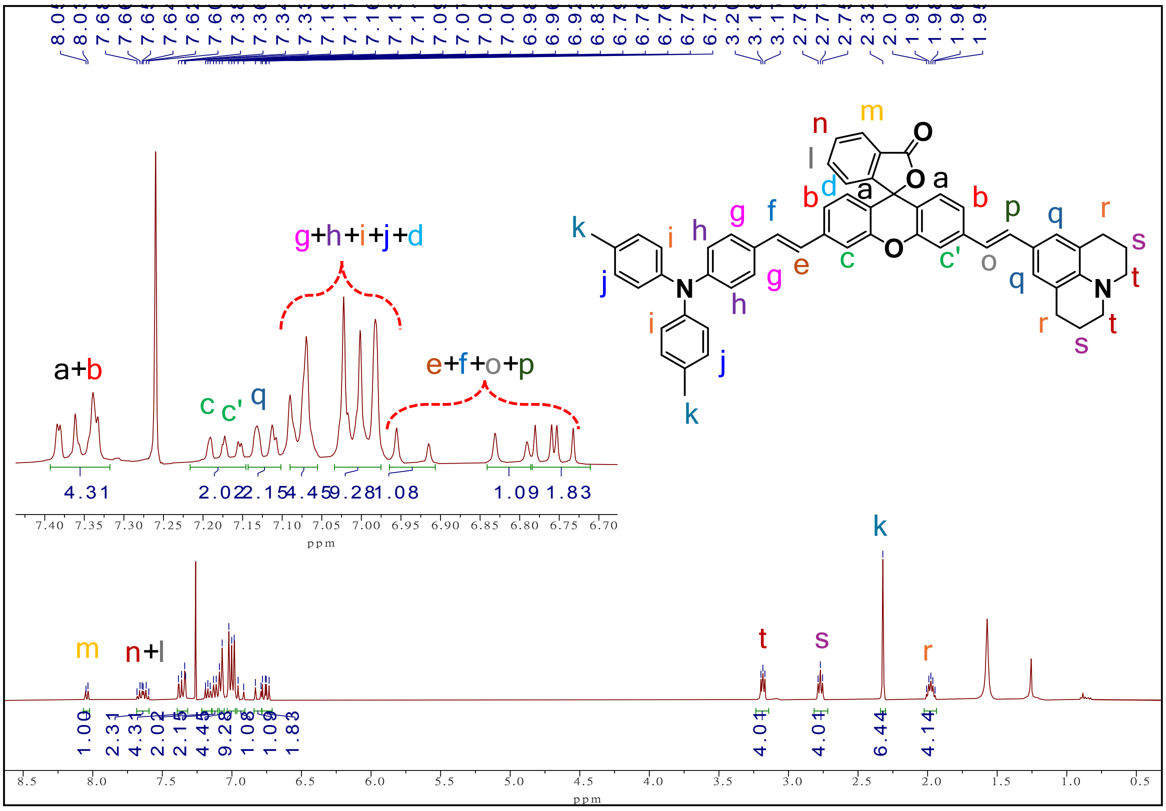


$^{13}$C NMR of **Compound 1f**

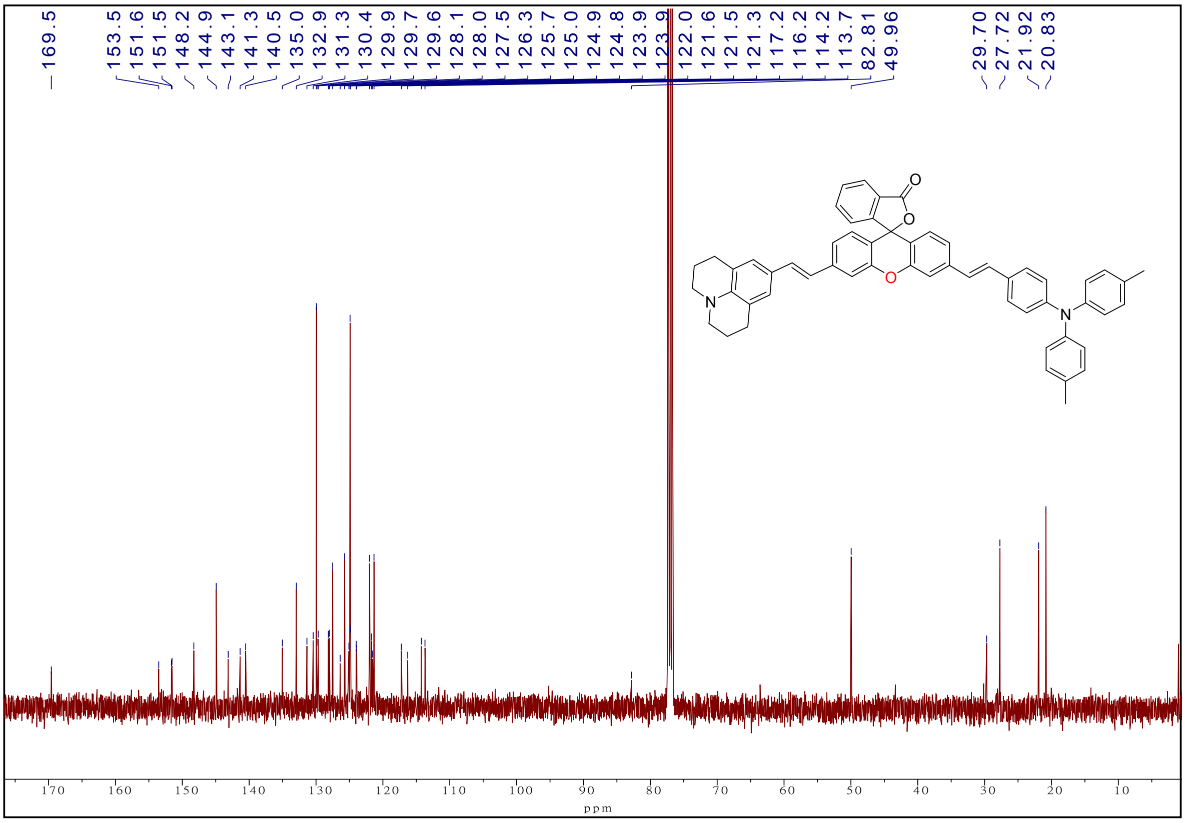


HRMS of **Compound 1f**

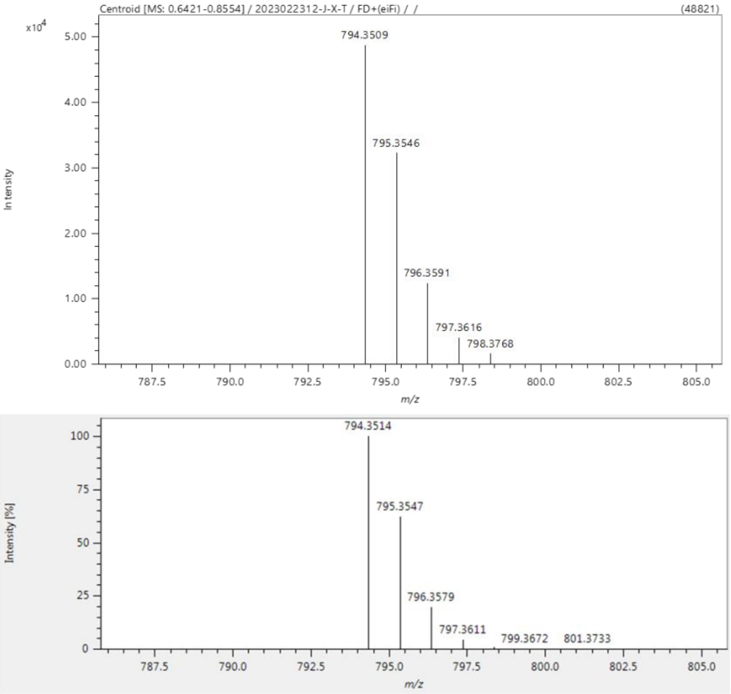


| Mass | Intensity | Formula | Calculated Mass | Mass Difference [mDa] | Mass Difference [ppm] | DBE |
|---|---|---|---|---|---|---|
| 794.35085 | 48820.99 | C56 H46 N2 O3 | 794.35139 | -0.54 | -0.68 | 35.0 |

$^{1}$H NMR of **Compound 1g**

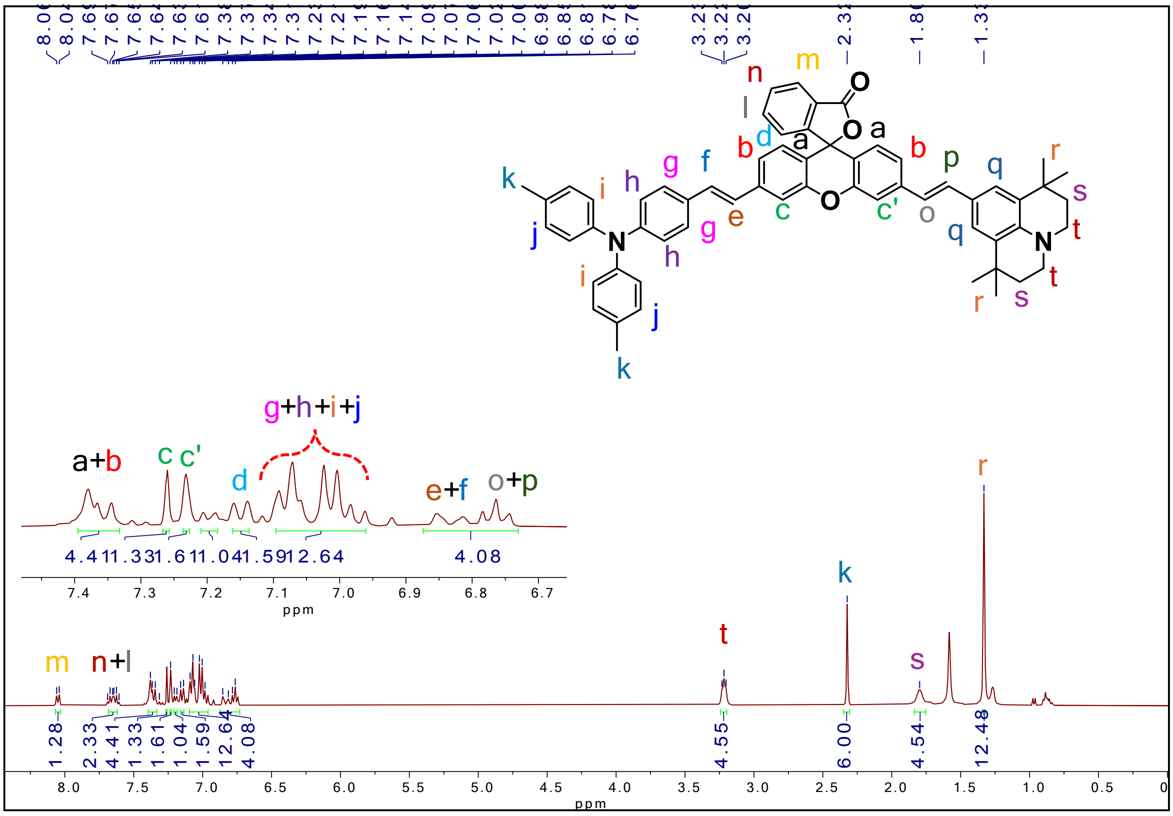

$^{13}C$ NMR of **Compound 1g**

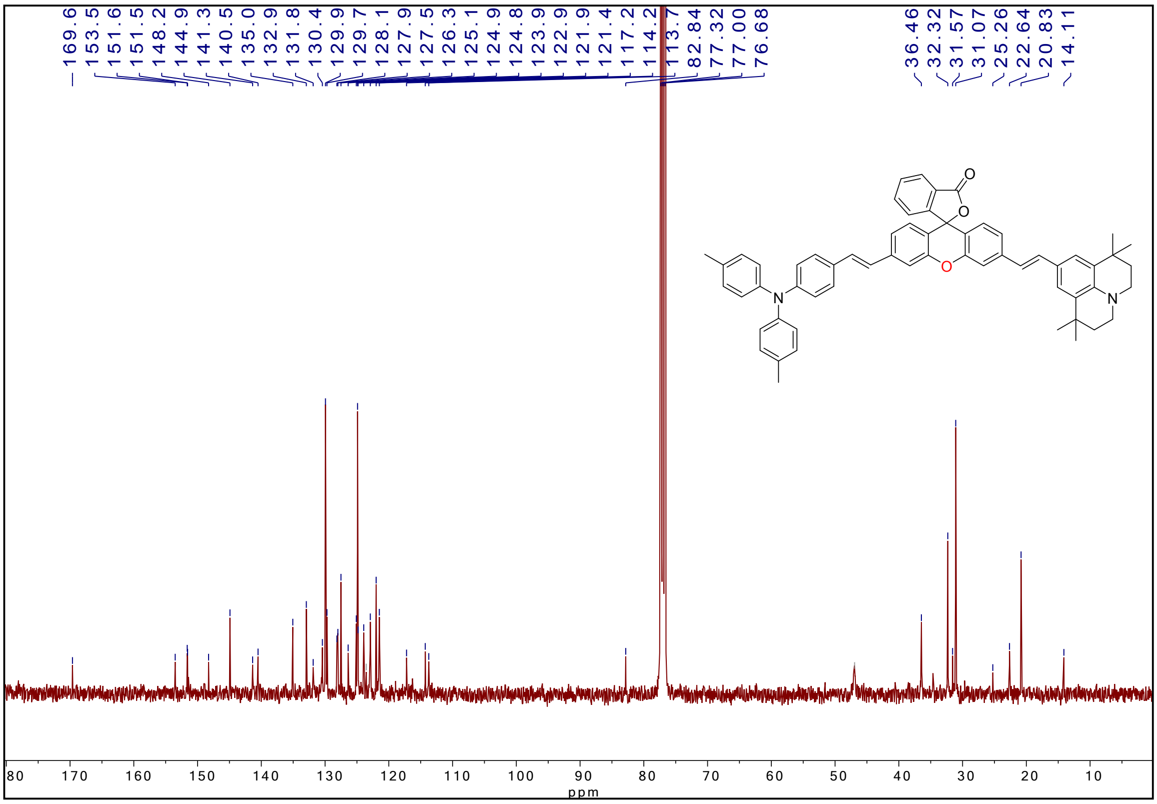

HRMS of **Compound 1g**

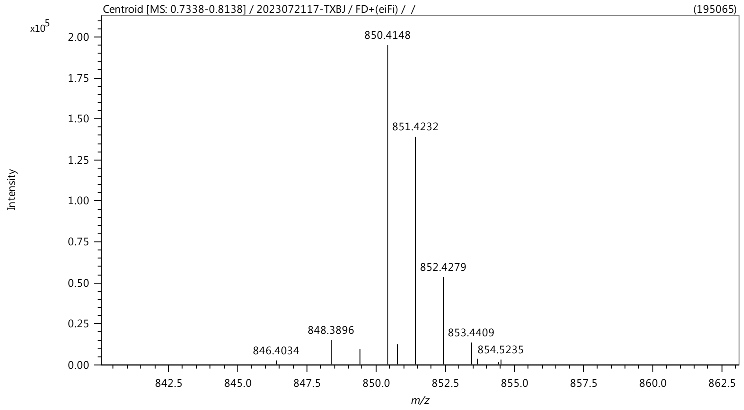


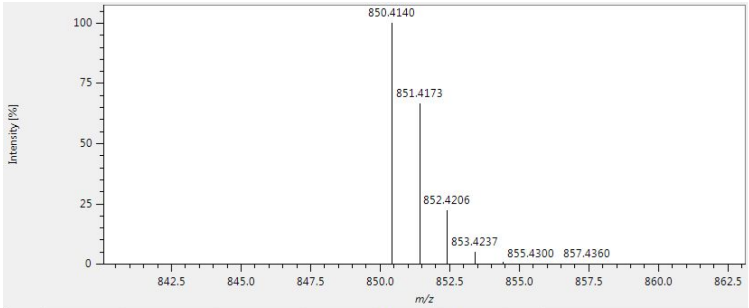


| Mass | Intensity | Formula | Calculated Mass | Mass Difference [mDa] | Mass Difference [ppm] | DBE |
|---|---|---|---|---|---|---|
| 850.41480 | 195065.03 | C60 H54 N2 O3 | 850.41399 | 0.81 | 0.95 | 35.0 |

$^{1}$H NMR of **Compound 1h**

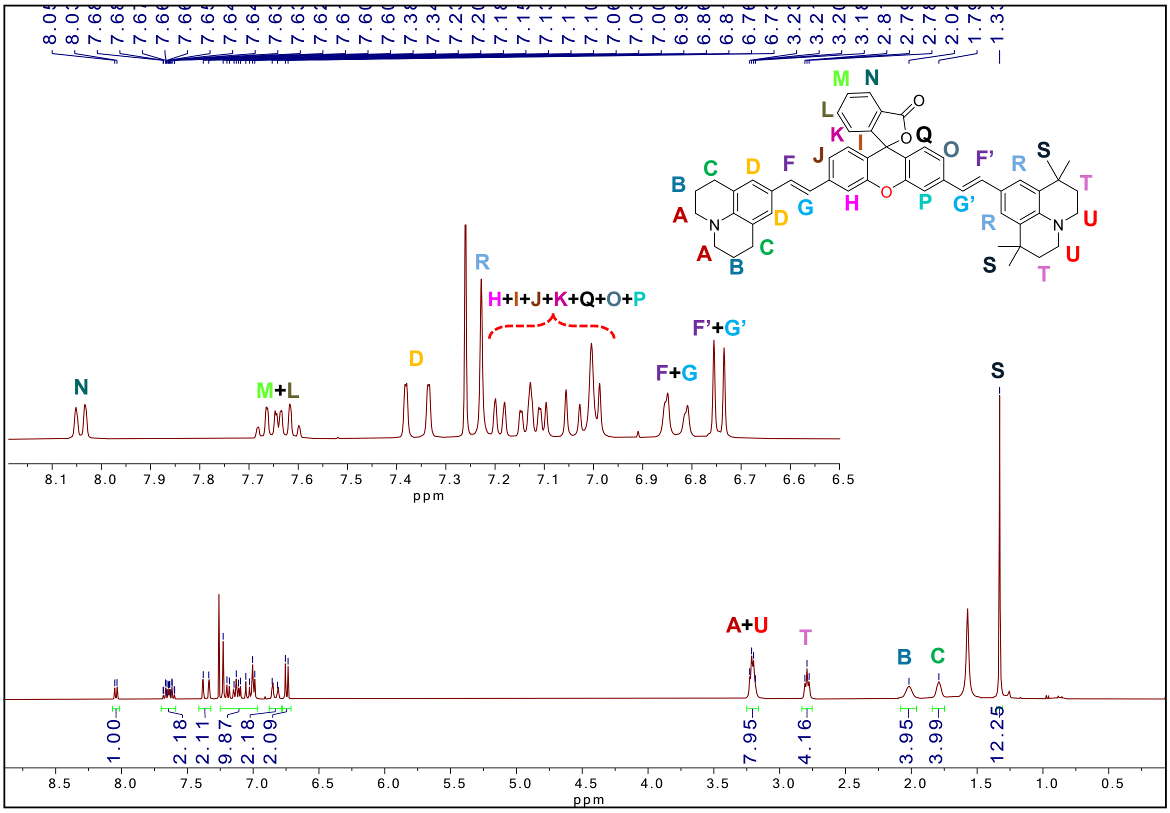


$^{13}$C NMR of **Compound 1h**

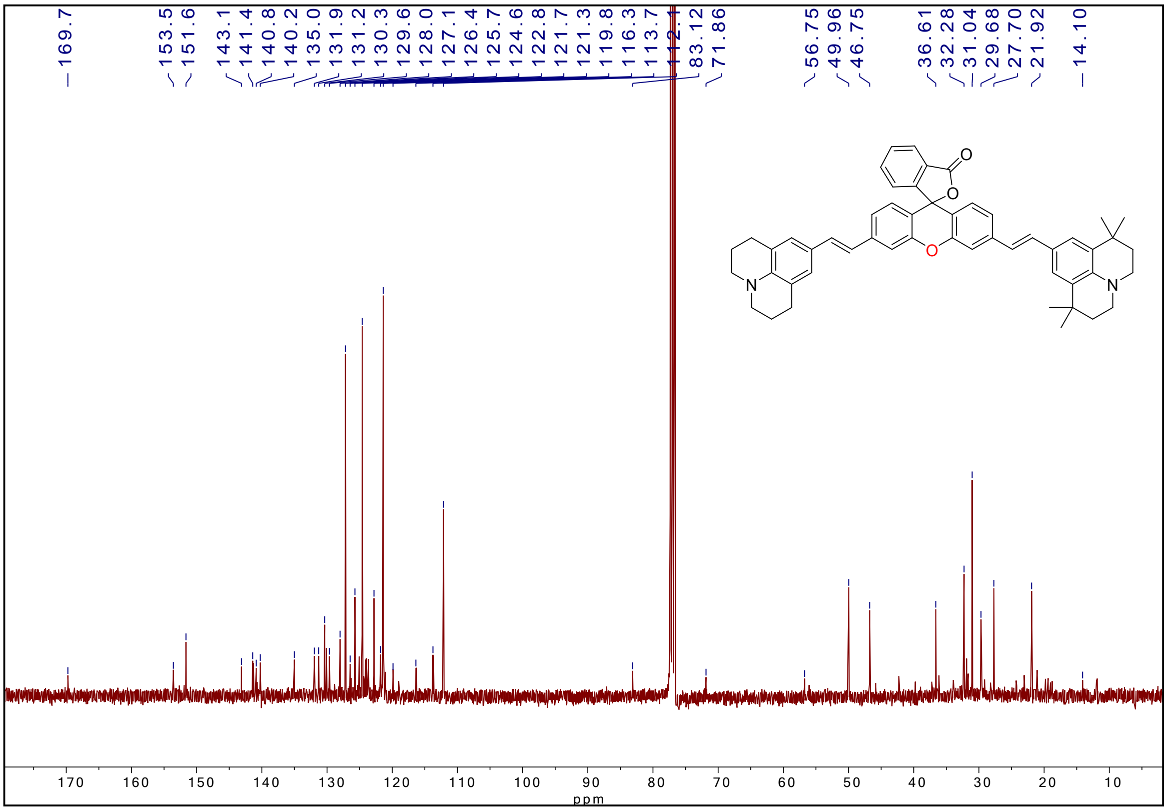


HRMS of **Compound 1h**

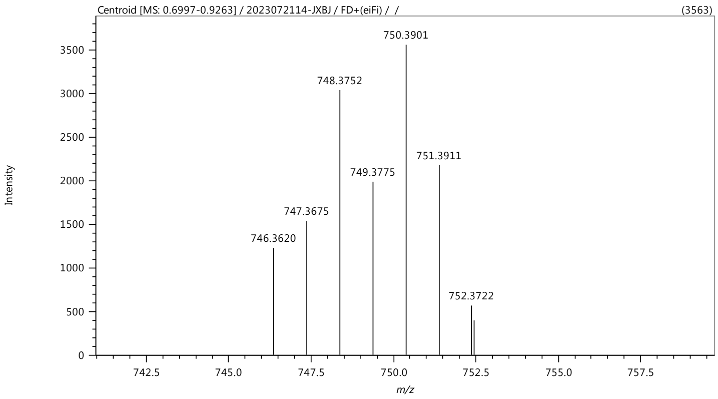


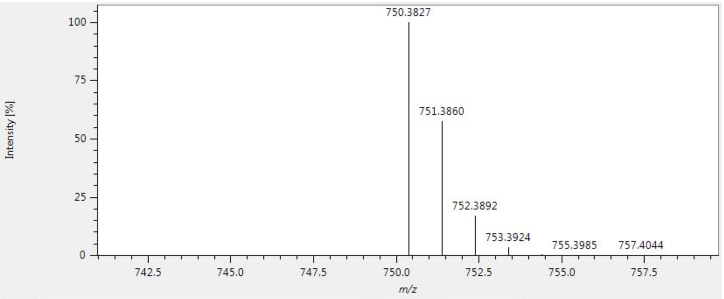


| Mass | Intensity | Formula | Calculated Mass | Mass Difference [mDa] | Mass Difference [ppm] | DBE |
|---|---|---|---|---|---|---|
| 750.39007 | 3562.73 | C52 H50 N2 O3 | 750.38269 | 7.38 | 9.84 | 29.0 |

$^{1}$H NMR of **Compound 2a**

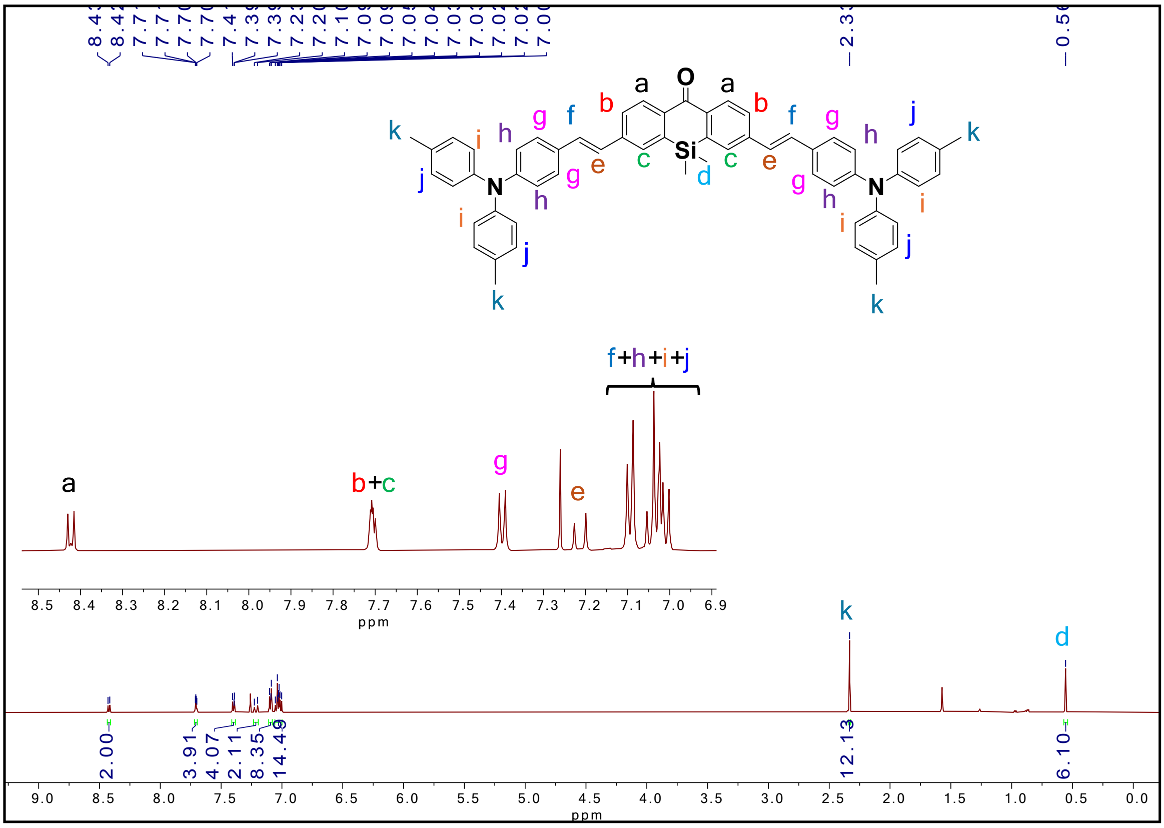



$^{13}$C NMR of **Compound 2a**

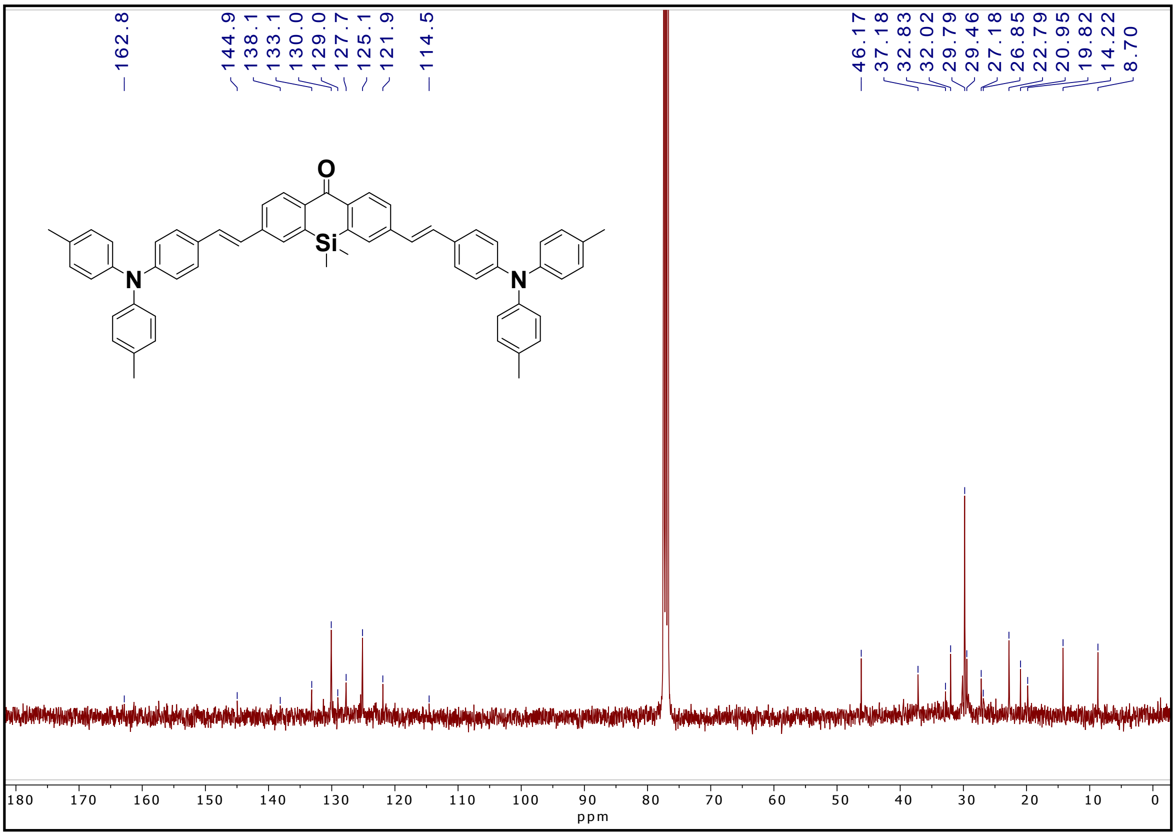



HRMS of **Compound 2a**

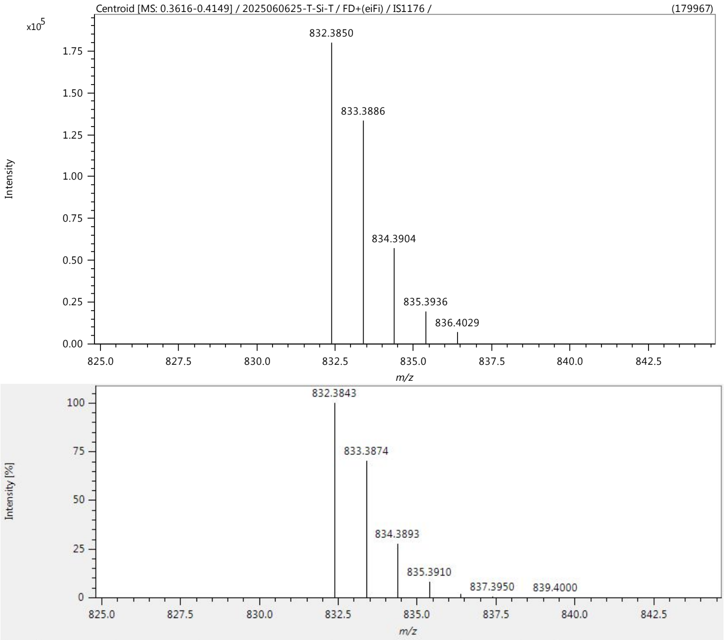


| | Mass | Intensity | Formula | Calculated Mass | Mass Difference [mDa] | Mass Difference [ppm] | DBE |
|---|---|---|---|---|---|---|---|
| ▸ | 832.38501 | 179967.01 | C59 H52 N2 O Si | 832.38434 | 0.67 | 0.80 | 36.0 |

$^{1}$H NMR of **Compound 2b**

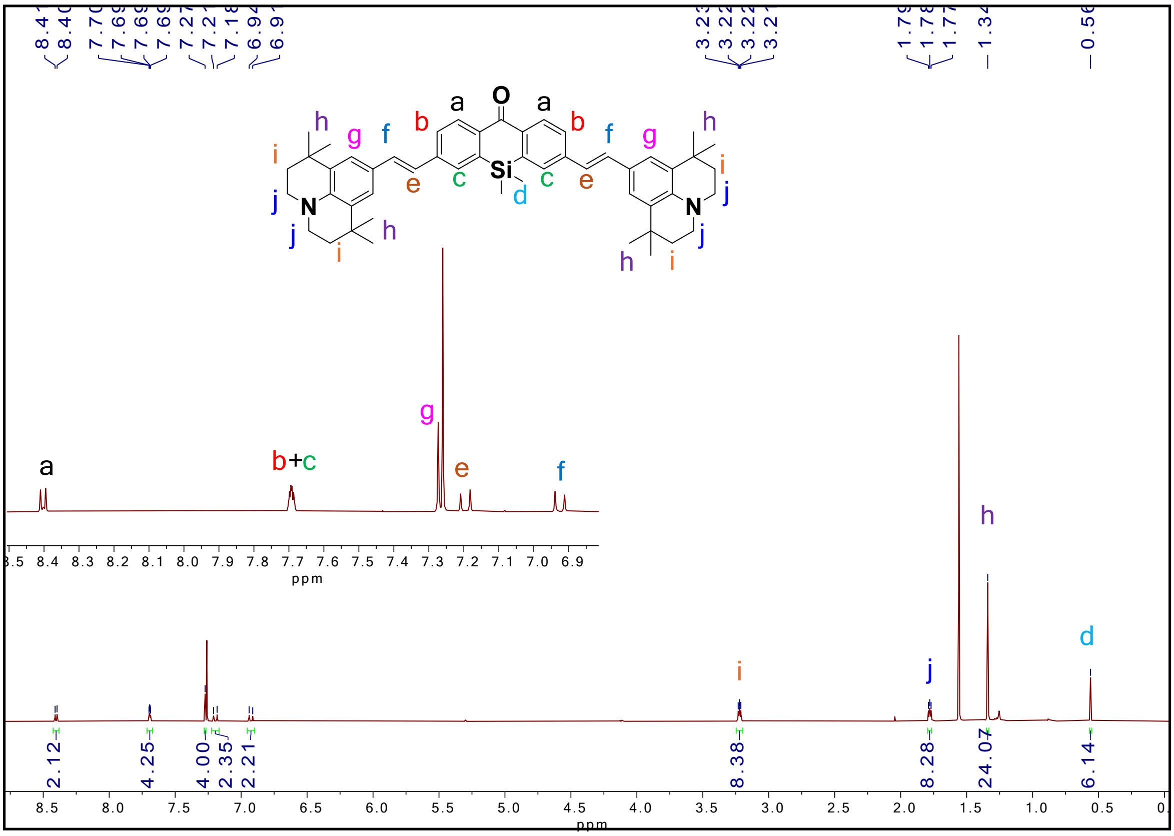


$^{13}$C NMR of **Compound 2b**

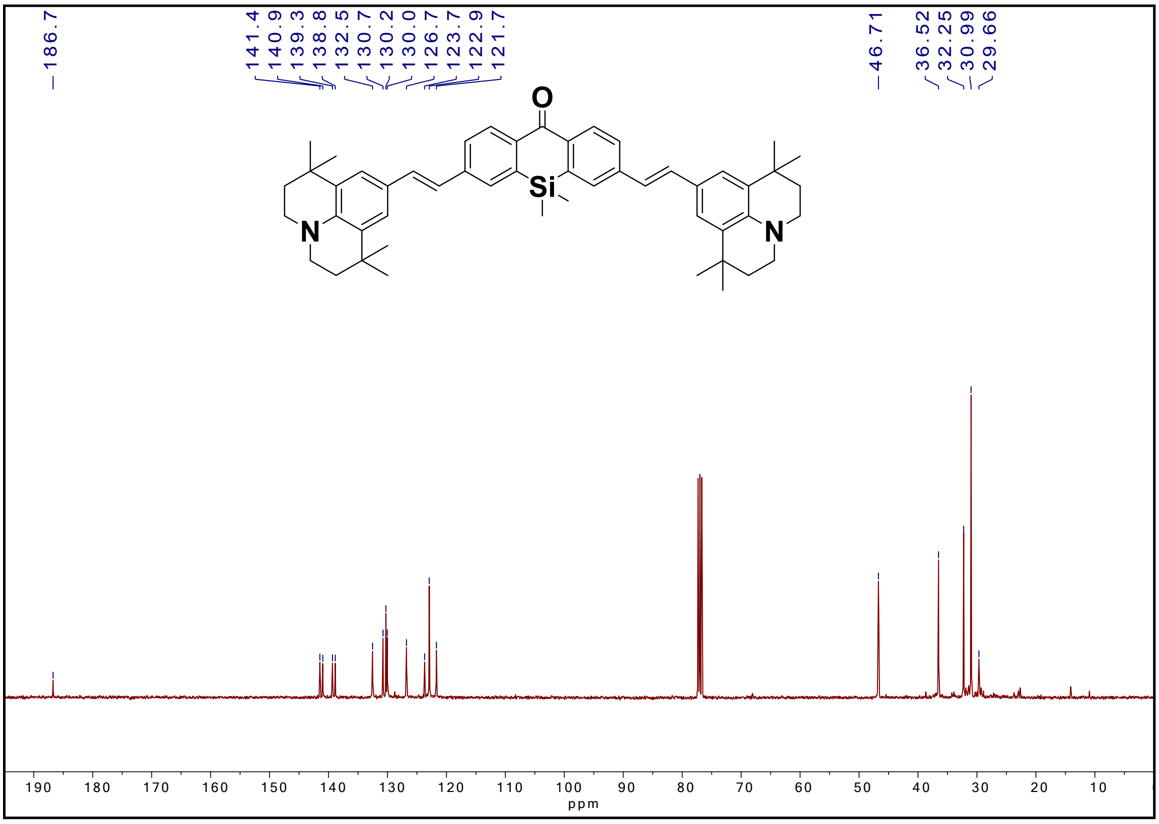


HRMS of **Compound 2b**

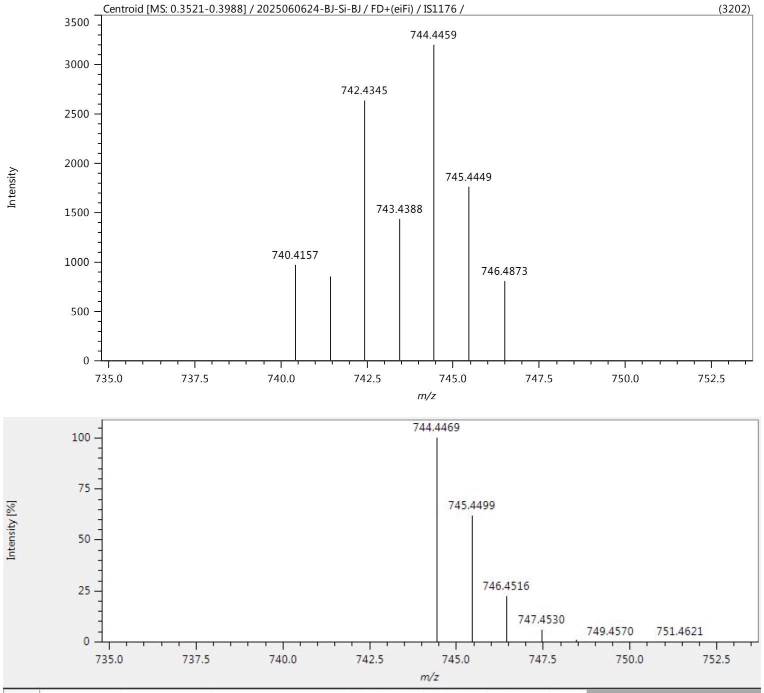


| | Mass | Intensity | Formula | Calculated Mass | Mass Difference [mDa] | Mass Difference [ppm] | DBE |
|---|---|---|---|---|---|---|---|
| ▸ | 744.44588 | 3202.07 | C51 H60 N2 O Si | 744.44694 | -1.06 | -1.43 | 24.0 |

$^{1}$H NMR of **Compound 2c**

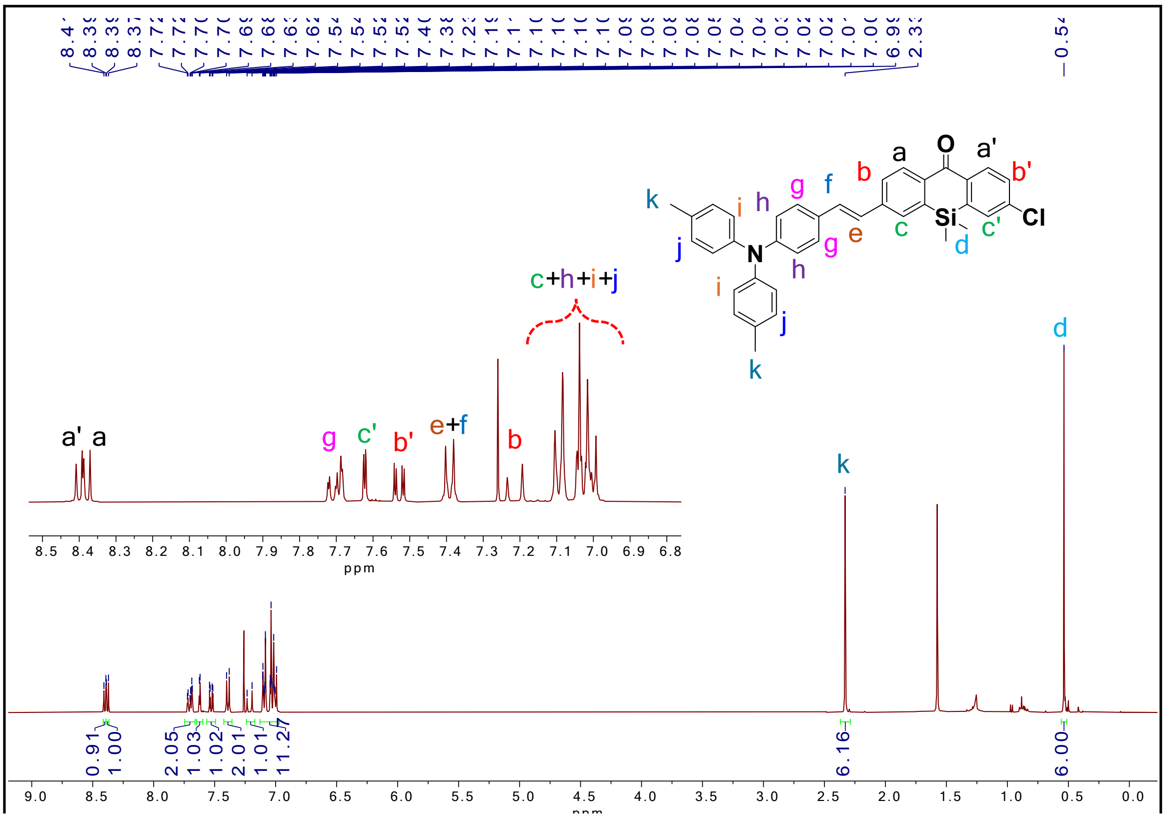

$^{13}$C NMR of **Compound 2c**

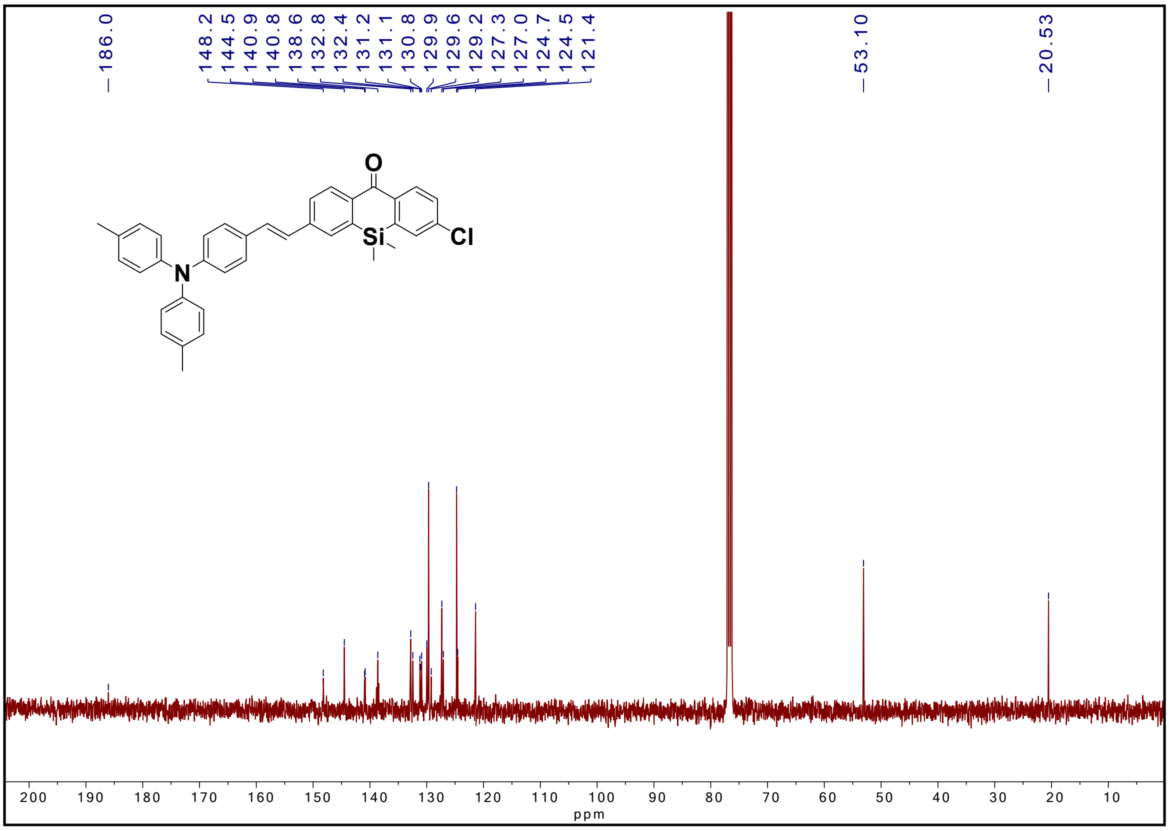

HRMS of **Compound 2c**

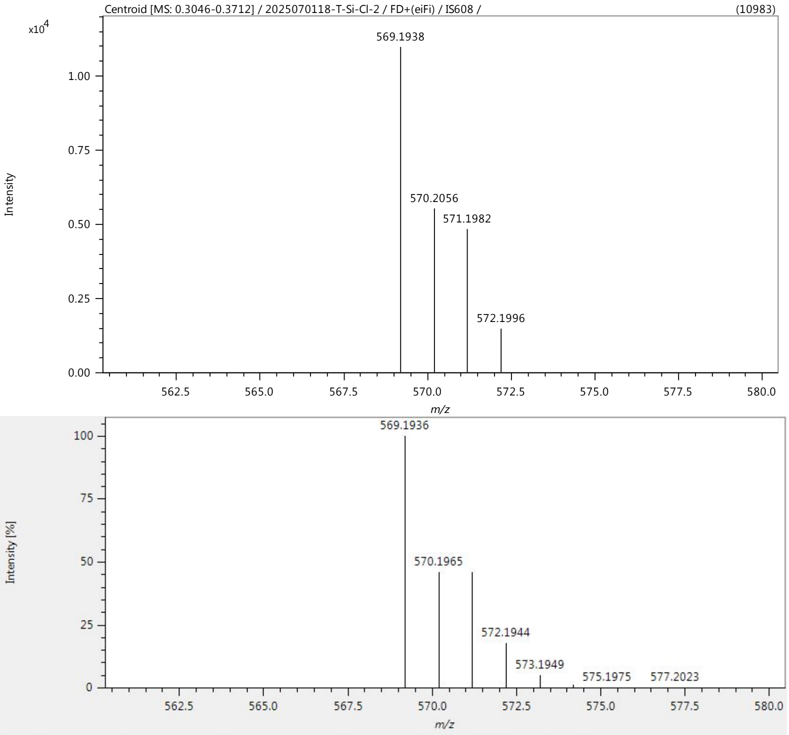


| | Mass | Intensity | Formula | Calculated Mass | Mass Difference [mDa] | Mass Difference [ppm] | DBE |
|---|---|---|---|---|---|---|---|
| ▸ | 569.19380 | 10982.54 | C37 H32 N O Si Cl | 569.19362 | 0.18 | 0.32 | 23.0 |

$^{1}$H NMR of **Compound 2d**

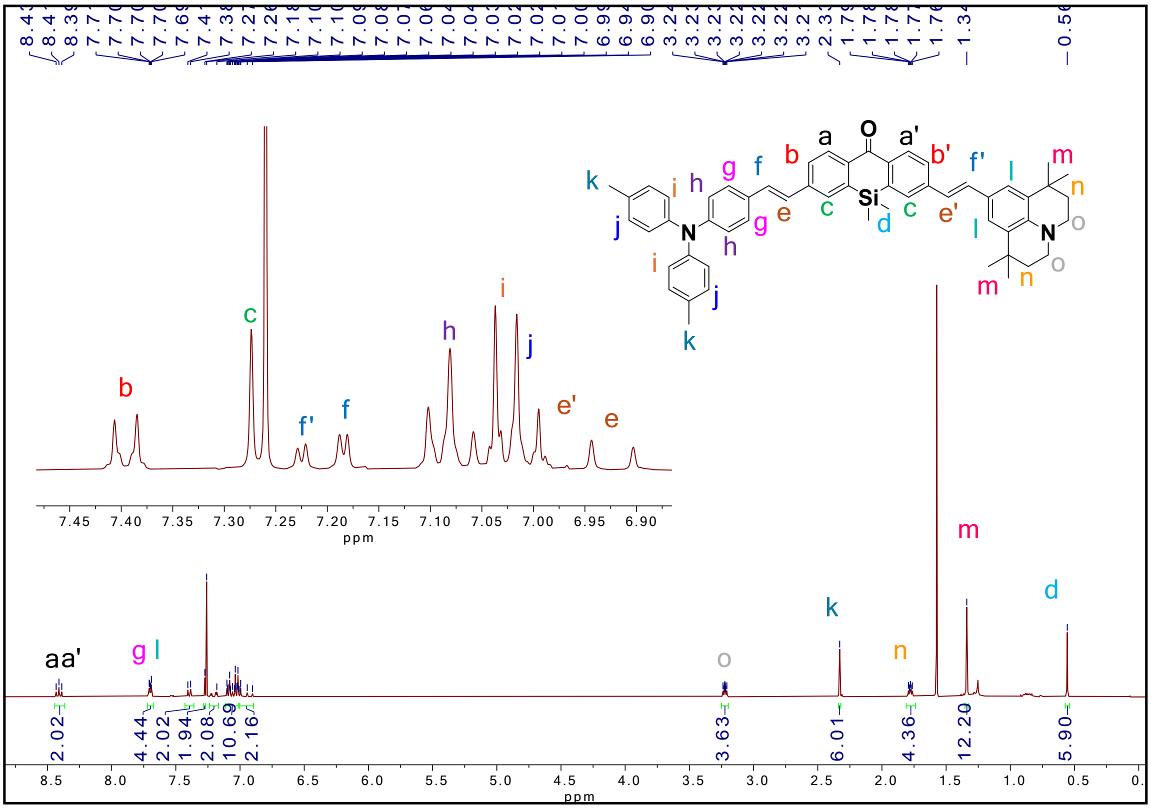


$^{13}$C NMR of **Compound 2d**

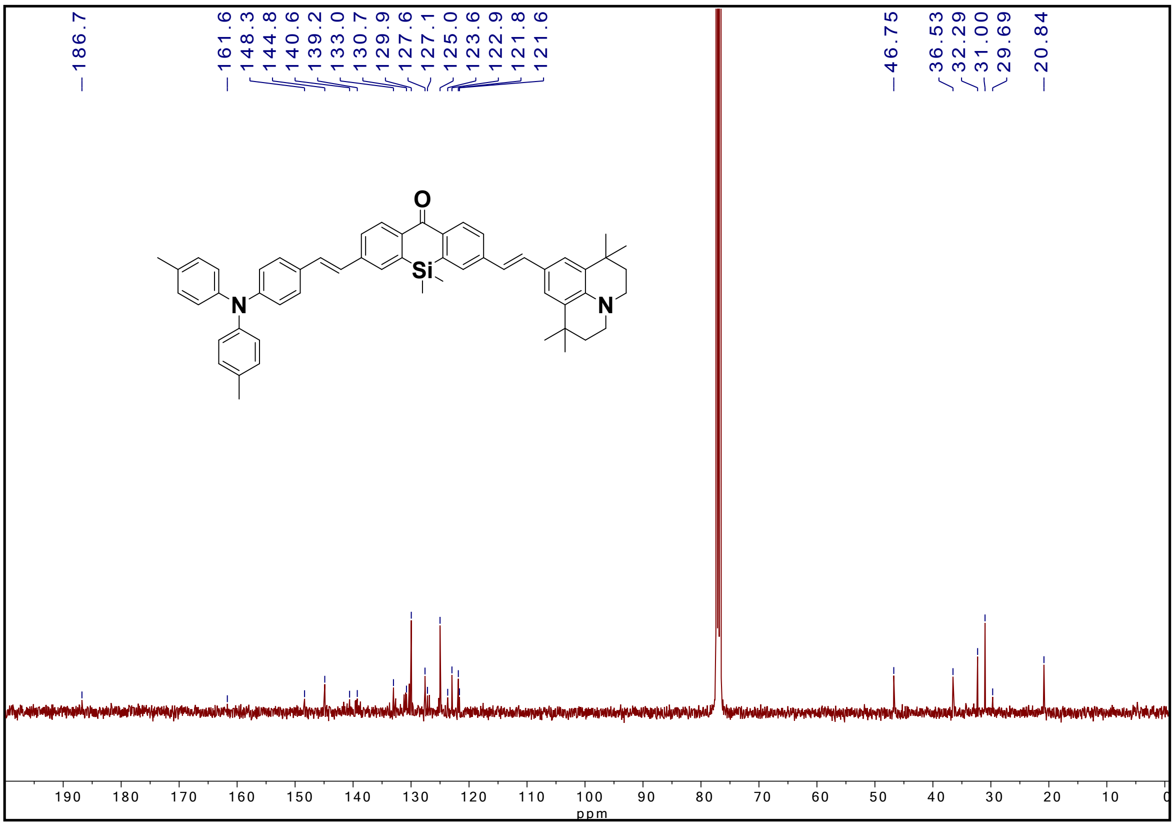


HRMS of **Compound 2d**

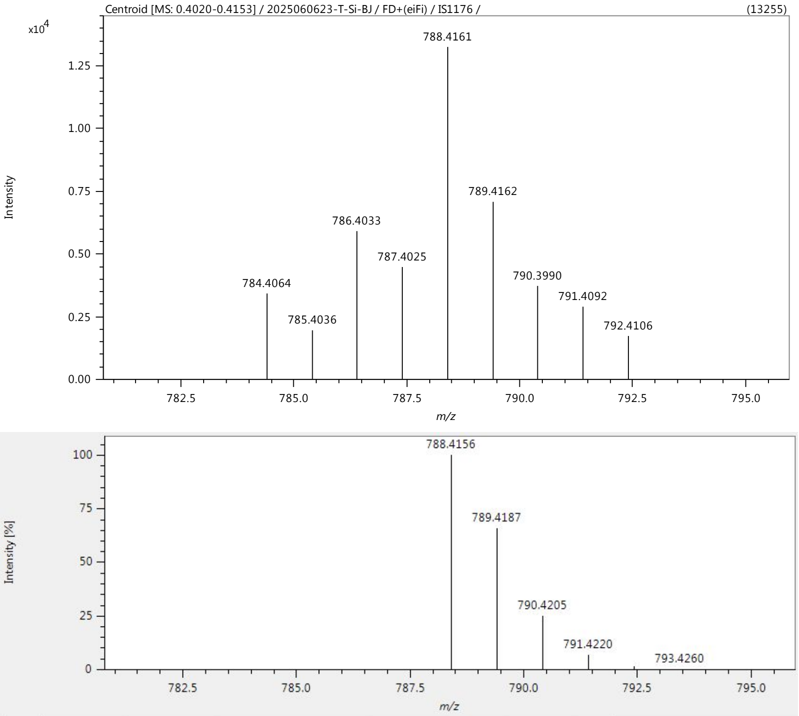


| | Mass | Intensity | Formula | Calculated Mass | Mass Difference [mDa] | Mass Difference [ppm] | DBE |
|---|---|---|---|---|---|---|---|
| ▸ | 788.41613 | 13255.45 | C55 H56 N2 O Si | 788.41564 | 0.49 | 0.62 | 30.0 |

$^{1}$H NMR of **Compound T-X-T**

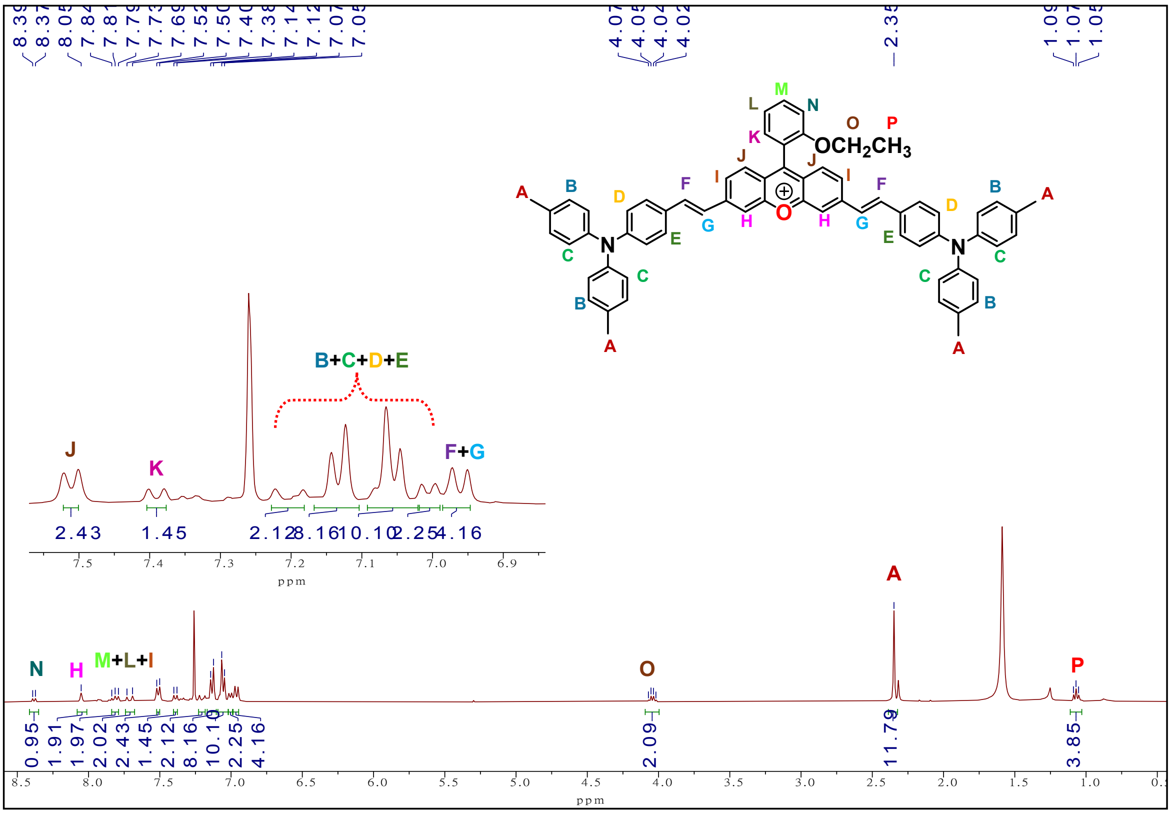


$^{13}$C NMR of **Compound T-X-T**

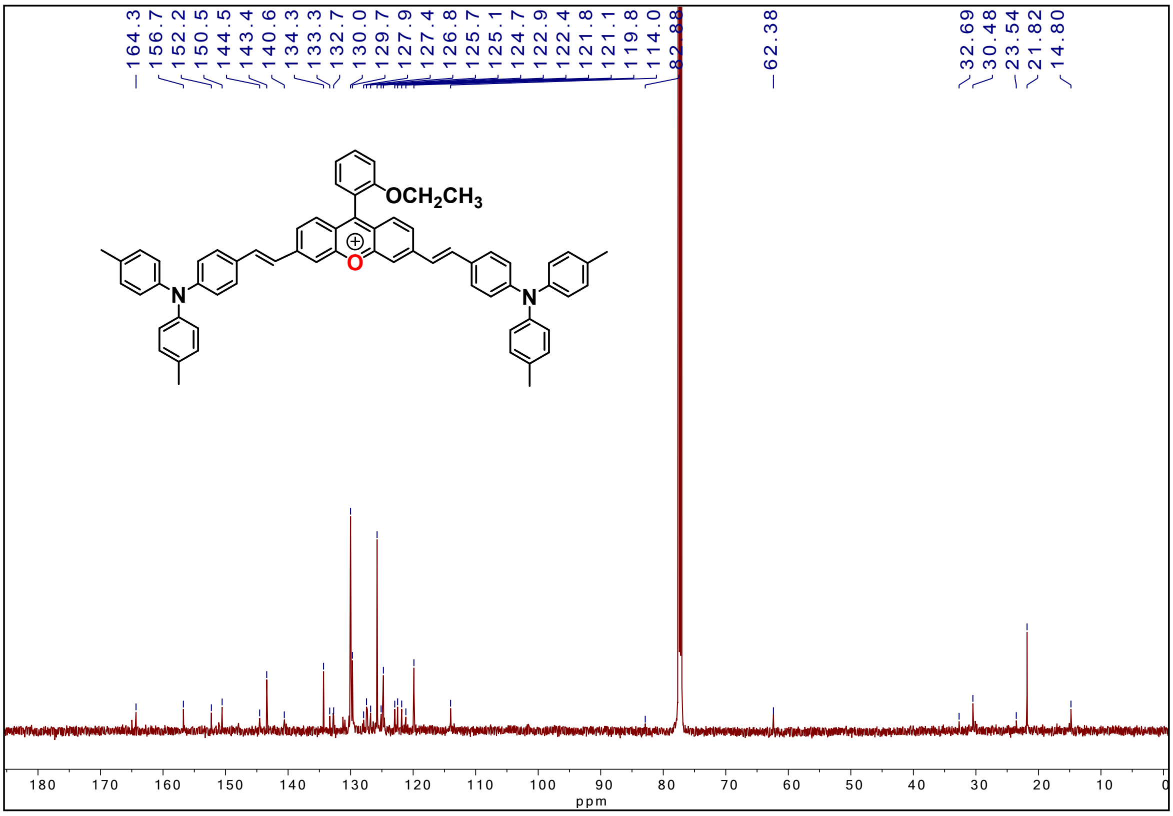


HRMS of **Compound T-X-T**

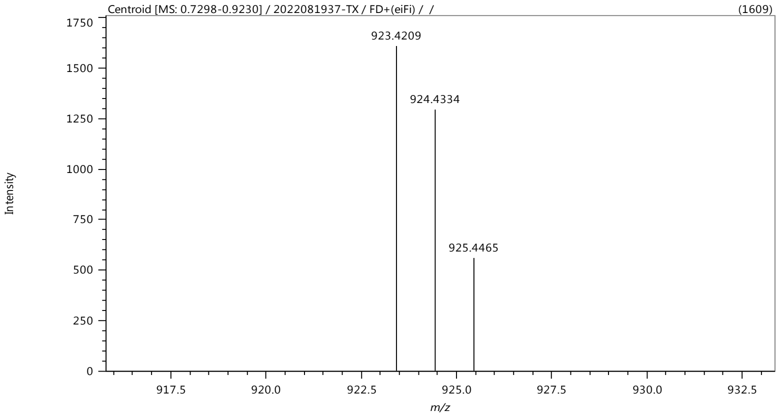


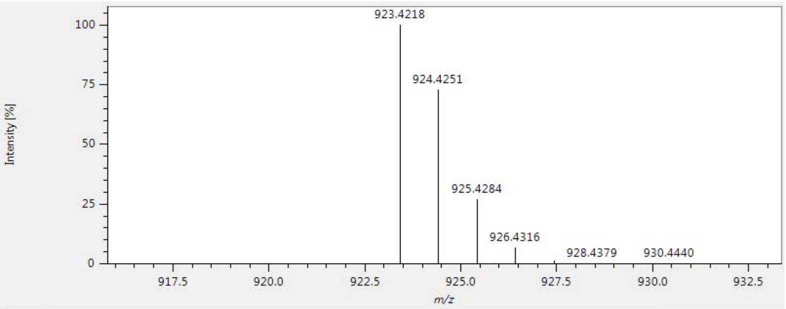


| Mass | Intensity | Formula | Calculated Mass | Mass Difference [mDa] | Mass Difference [ppm] | DBE |
|---|---|---|---|---|---|---|
| 923.42087 | 1609.17 | C66 H55 N2 O3 | 923.42182 | -0.95 | -1.03 | 40.5 |

$^{1}$H NMR of **Compound J-X-J**

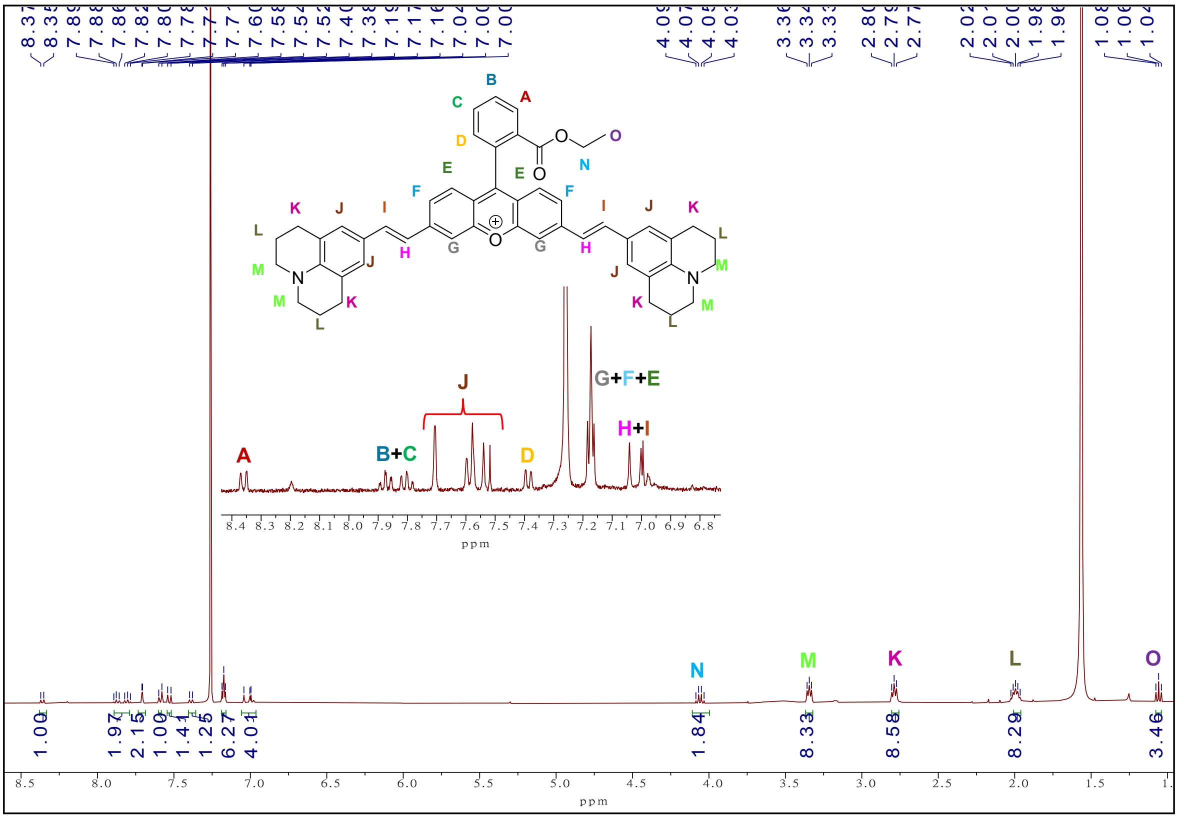


$^{13}$C NMR of **Compound J-X-J**

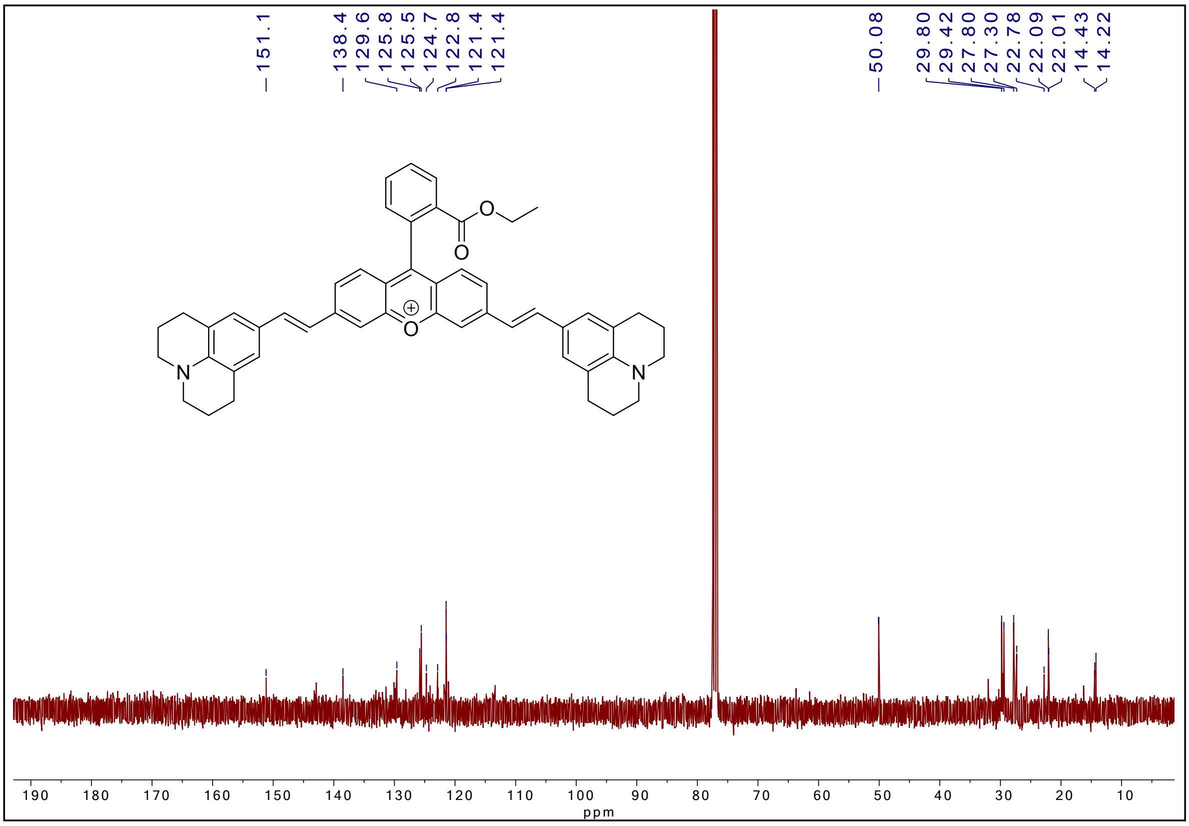


$^{1}$H NMR of **Compound BJ-X-BJ**

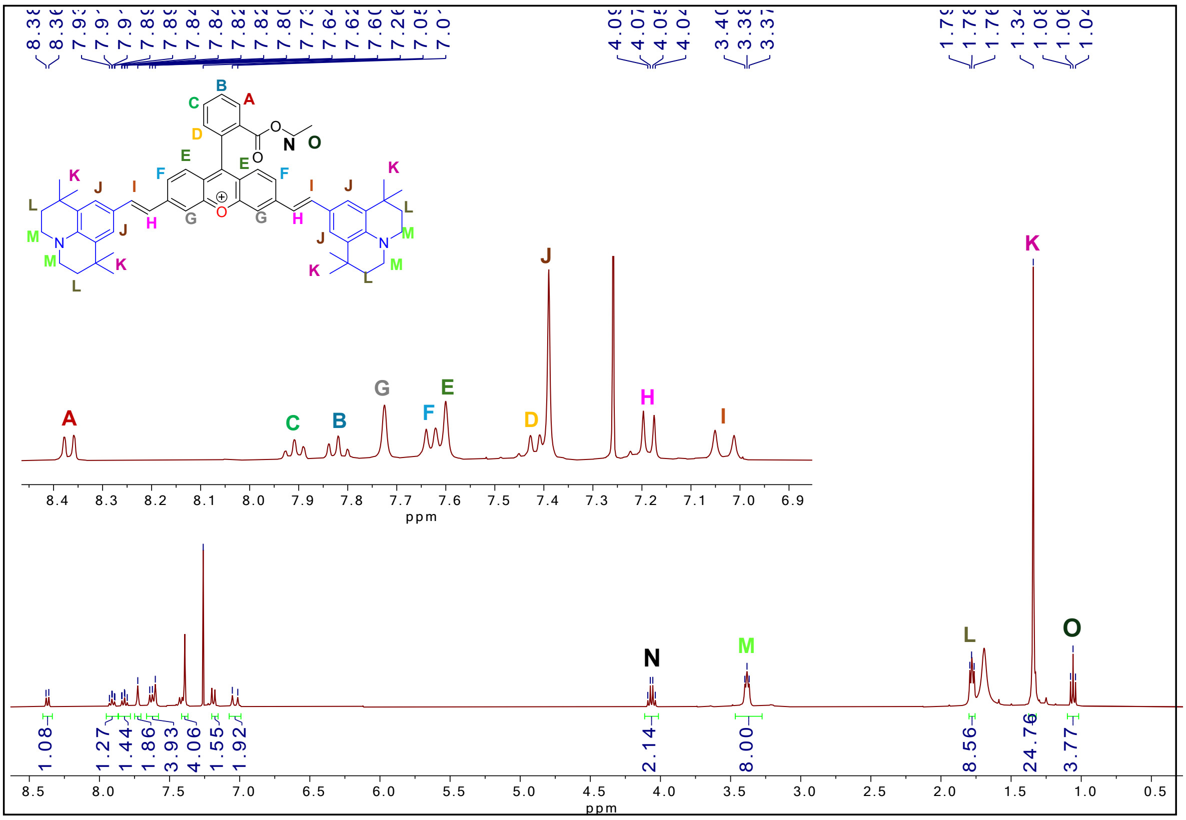


$^{13}$C NMR of **Compound BJ-X-BJ**

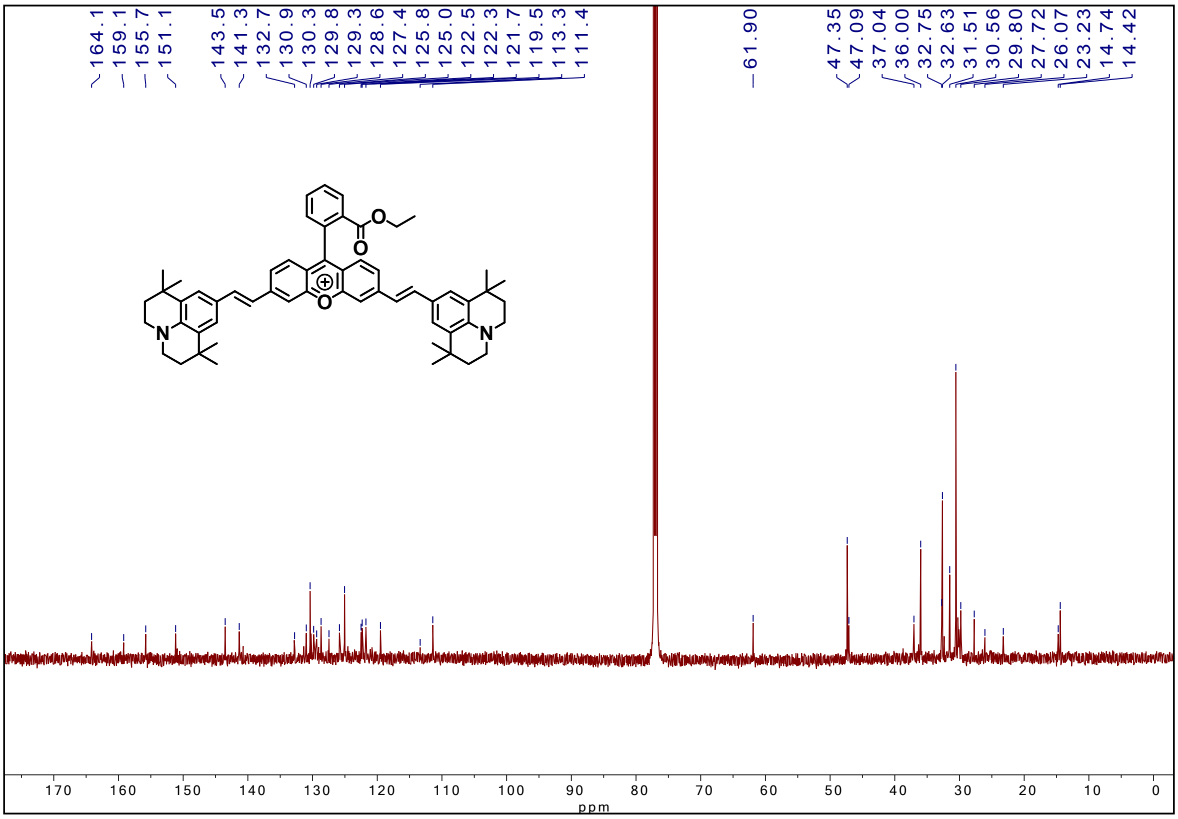


HRMS of **Compound BJ-X-BJ**

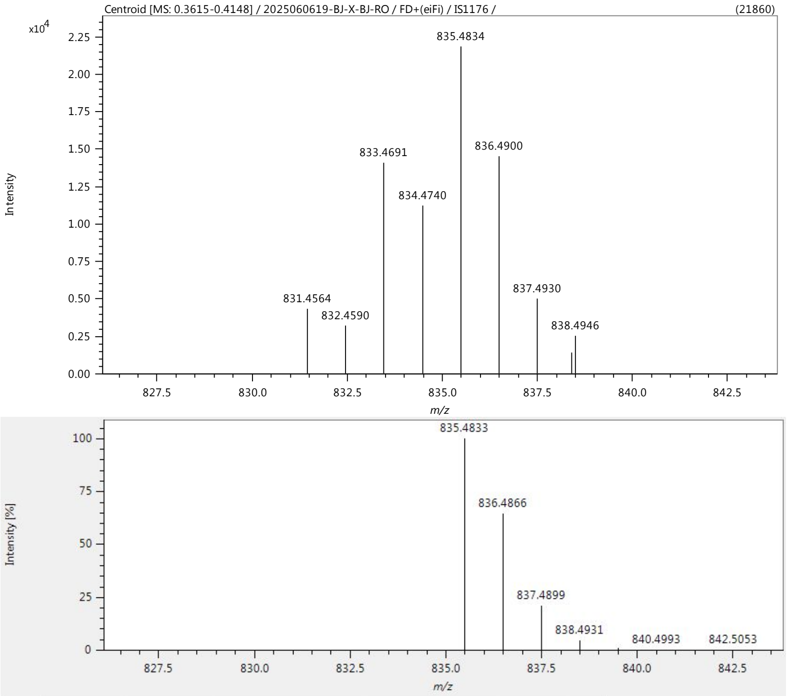


| | Mass | Intensity | Formula | Calculated Mass | Mass Difference [mDa] | Mass Difference [ppm] | DBE |
|---|---|---|---|---|---|---|---|
| ▸ | 835.48338 | 21860.23 | C58 H63 N2 O3 | 835.48332 | 0.06 | 0.08 | 28.5 |

$^{1}$H NMR of **Compound T-X-J**

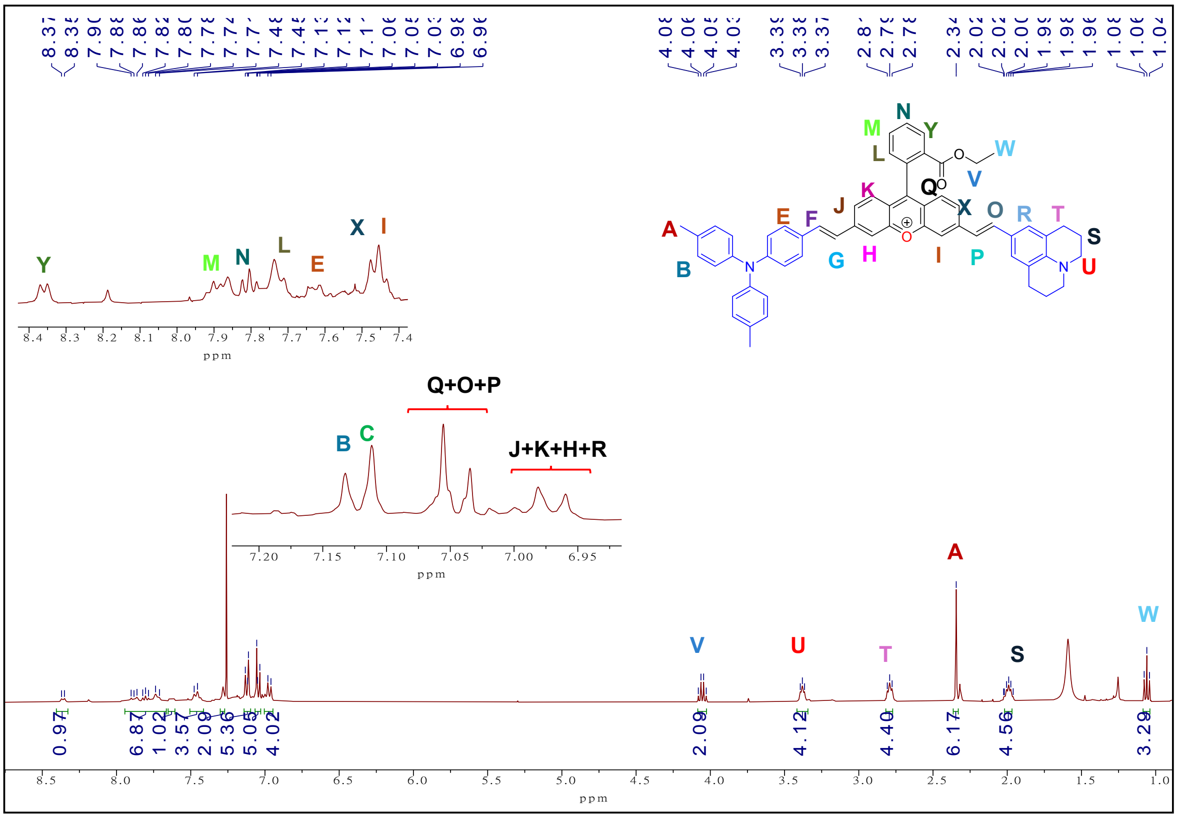


$^{13}$C NMR of **Compound T-X-J**

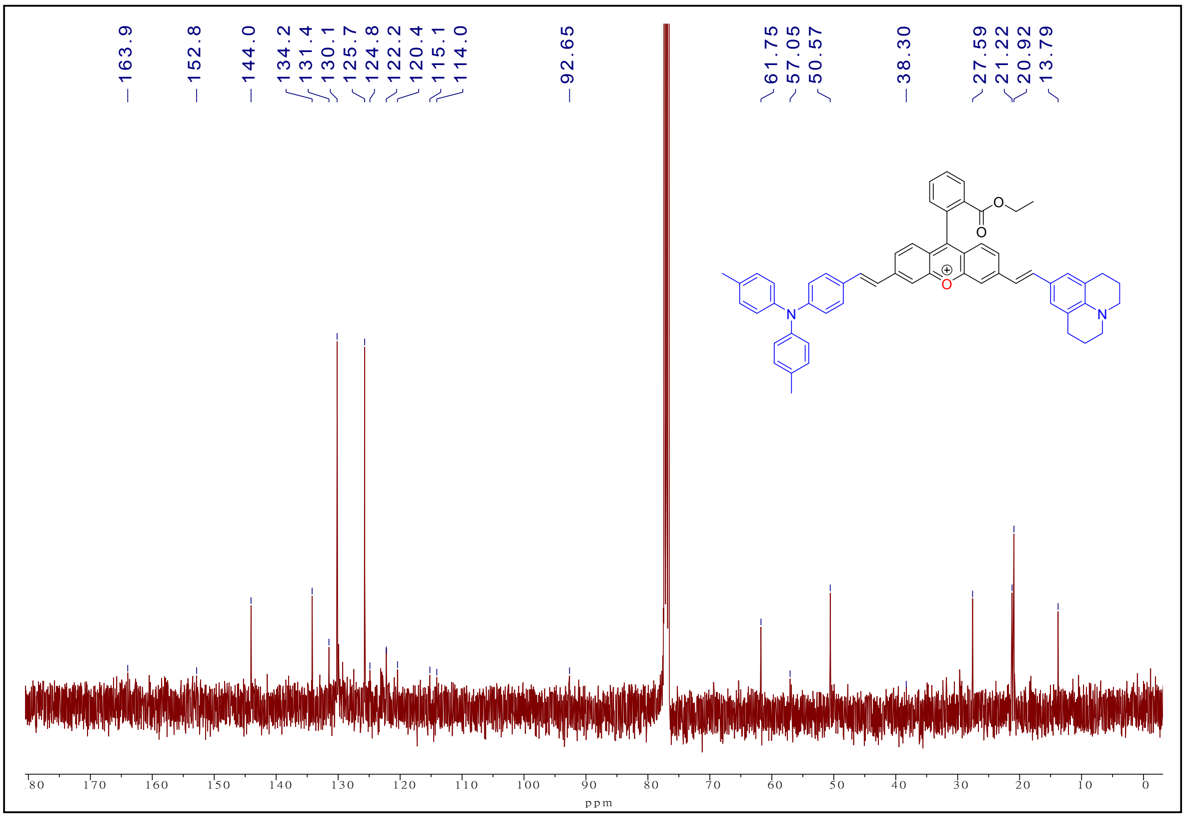


HRMS of **Compound T-X-J**

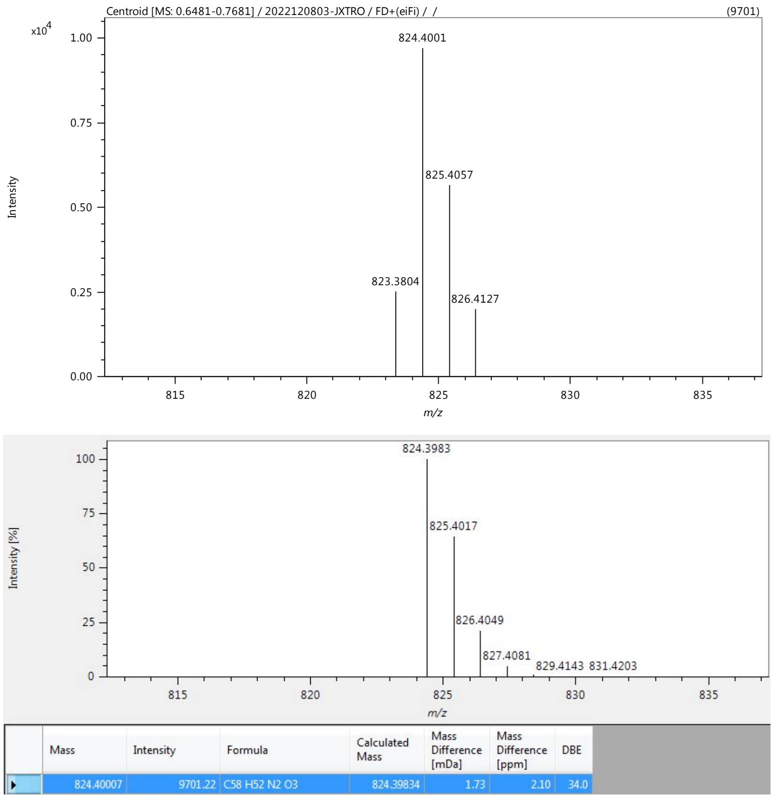

| Mass | Intensity | Formula | Calculated Mass | Mass Difference [mDa] | Mass Difference [ppm] | DBE |
|---|---|---|---|---|---|---|
| 824.40007 | 9701.22 | C58 H52 N2 O3 | 824.39834 | 1.73 | 2.10 | 34.0 |

$^{1}$H NMR of **Compound T-X-BJ**

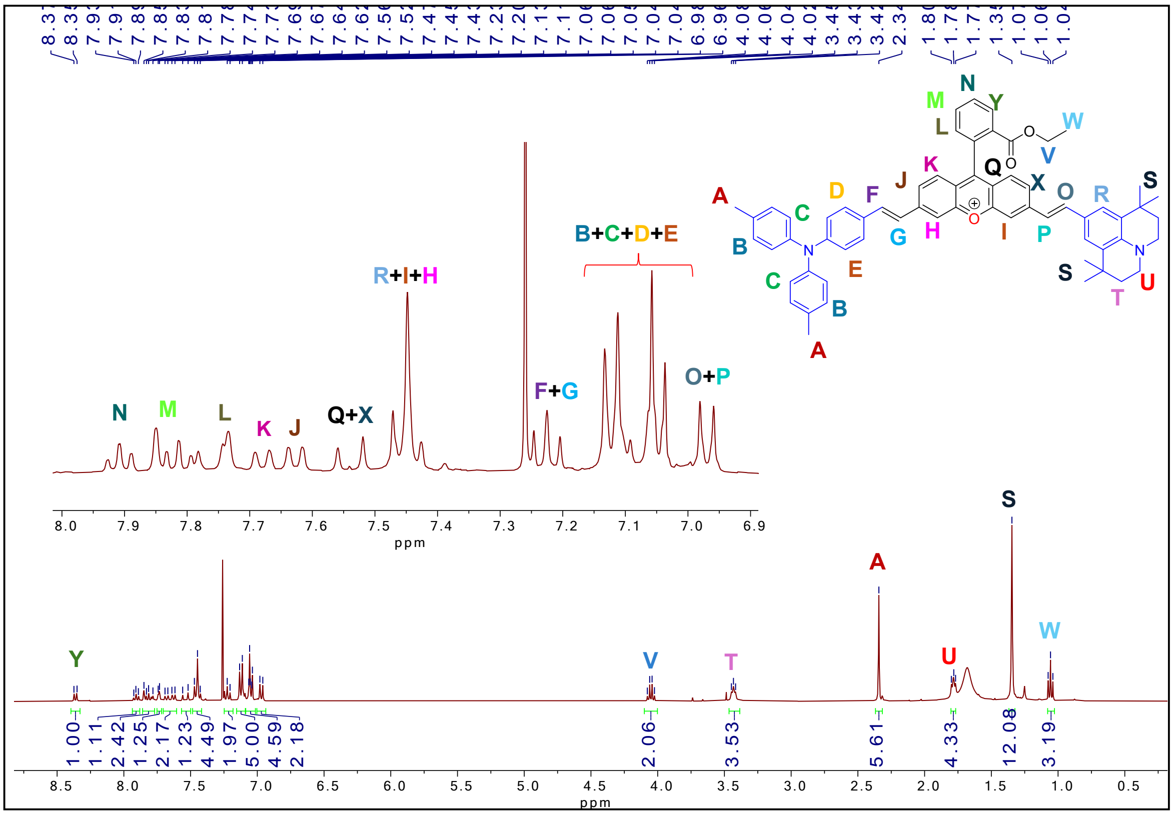

$^{13}$C NMR of **Compound T-X-BJ**

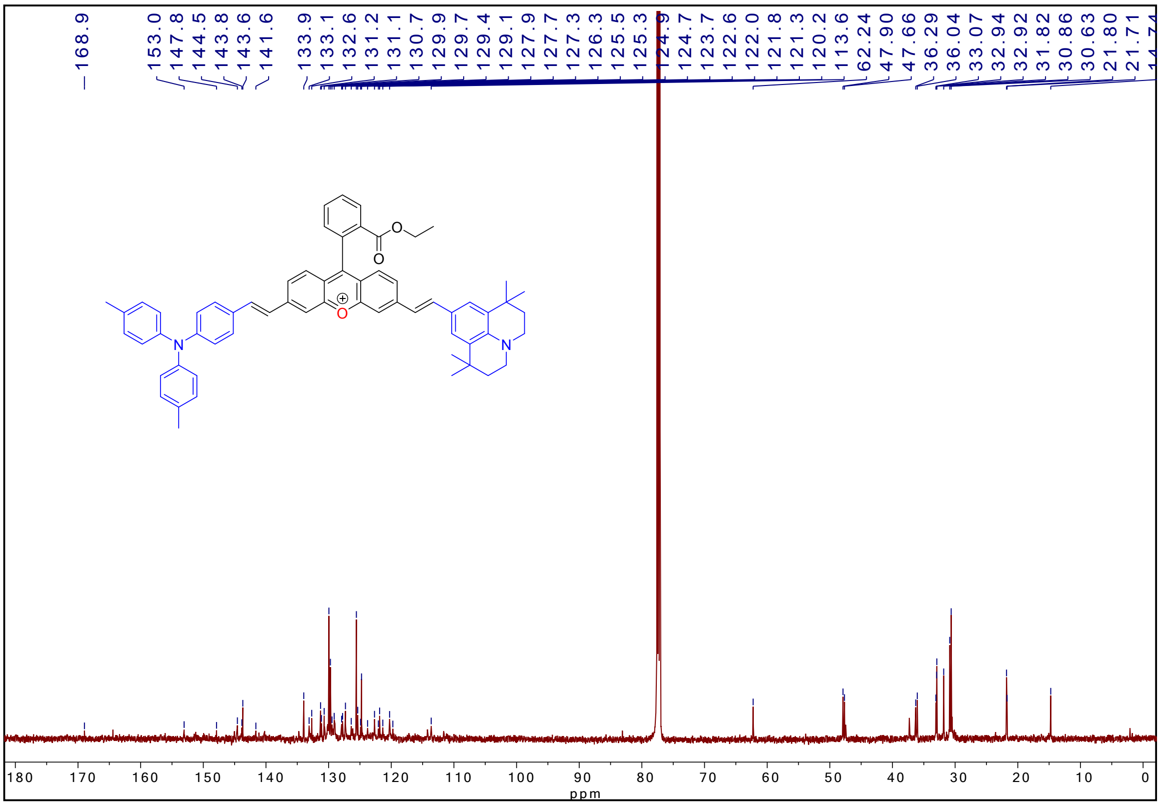


HRMS of **Compound T-X-BJ**

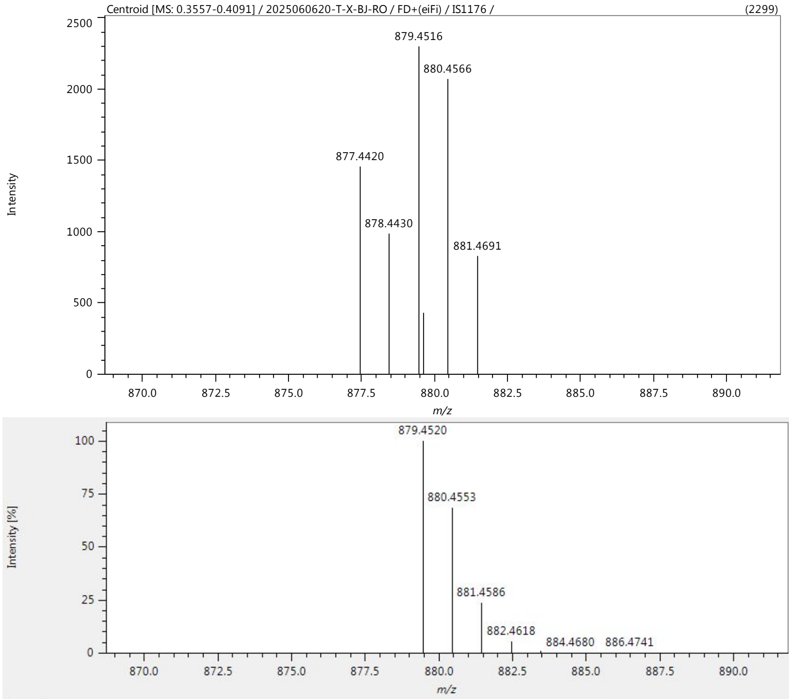


| | Mass | Intensity | Formula | Calculated Mass | Mass Difference [mDa] | Mass Difference [ppm] | DBE |
|---|---|---|---|---|---|---|---|
| ▸ | 879.45162 | 2299.31 | C62 H59 N2 O3 | 879.45202 | -0.40 | -0.45 | 34.5 |

$^{1}$H NMR of **Compound J-X-BJ**

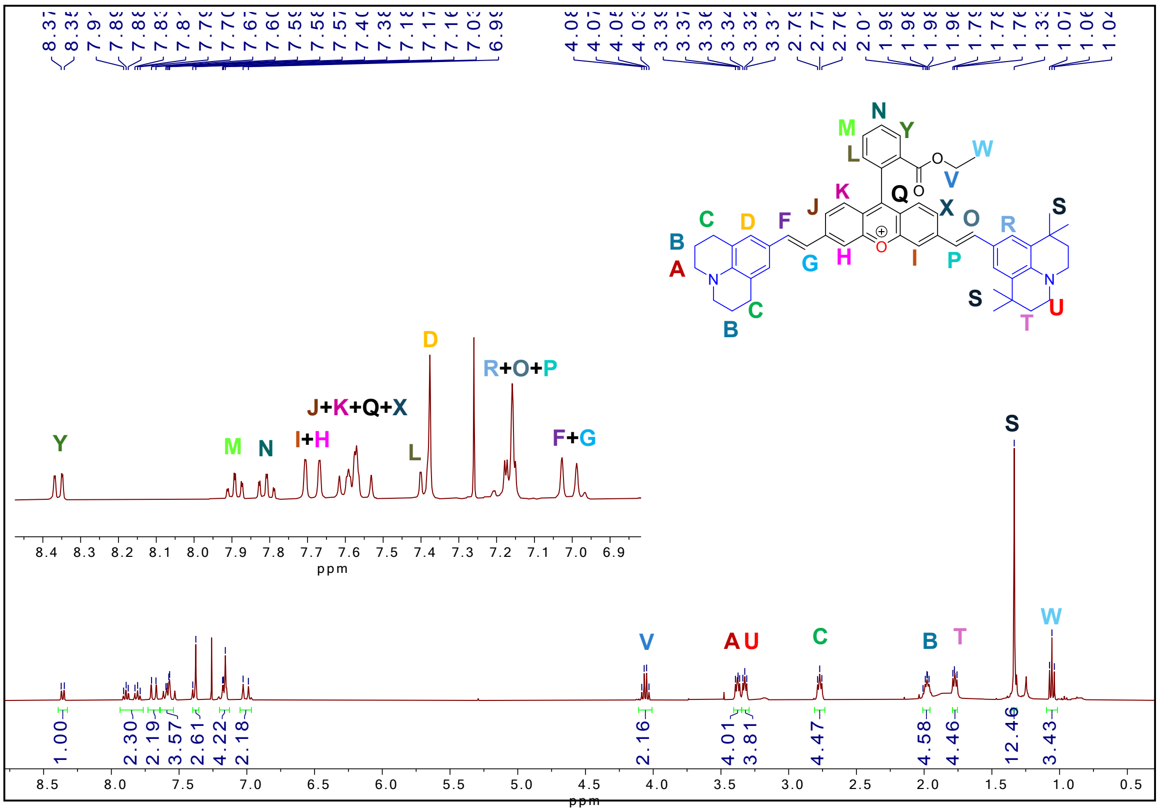

$^{13}$C NMR of **Compound J-X-BJ**

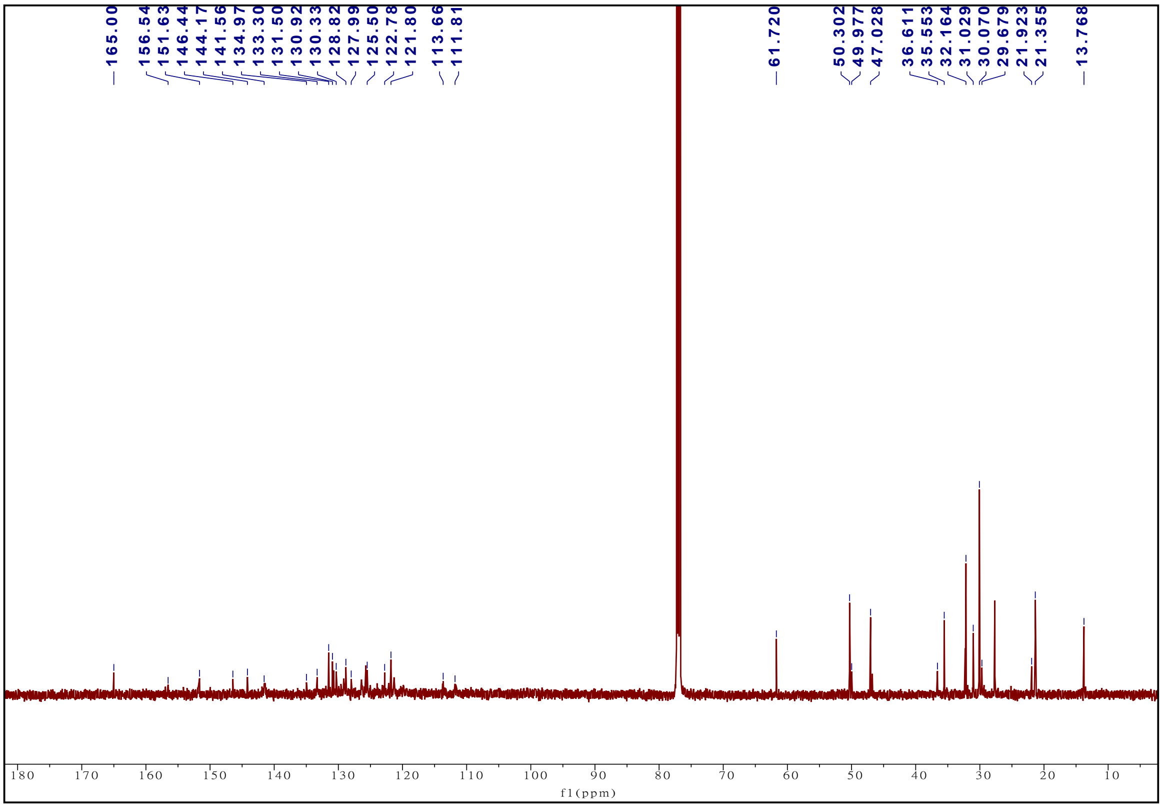

HRMS of **Compound J-X-BJ**

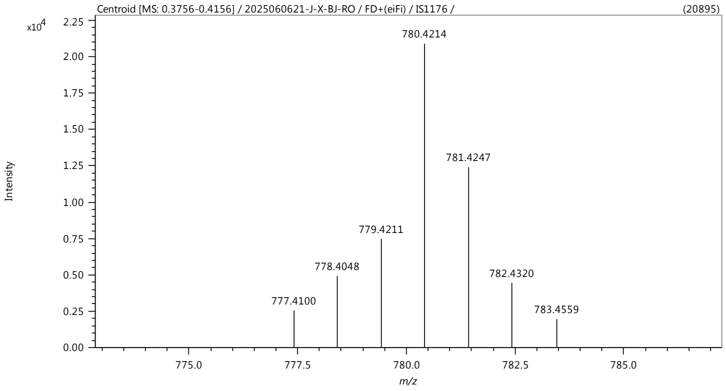


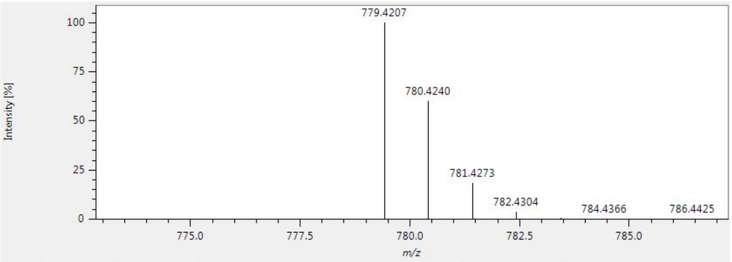


| | Mass | Intensity | Formula | Calculated Mass | Mass Difference [mDa] | Mass Difference [ppm] | DBE |
|---|---|---|---|---|---|---|---|
| ▸ | 779.42106 | 7485.44 | C54 H55 N2 O3 | 779.42072 | 0.34 | 0.44 | 28.5 |

$^{1}$H NMR of **Compound T-Si-T**

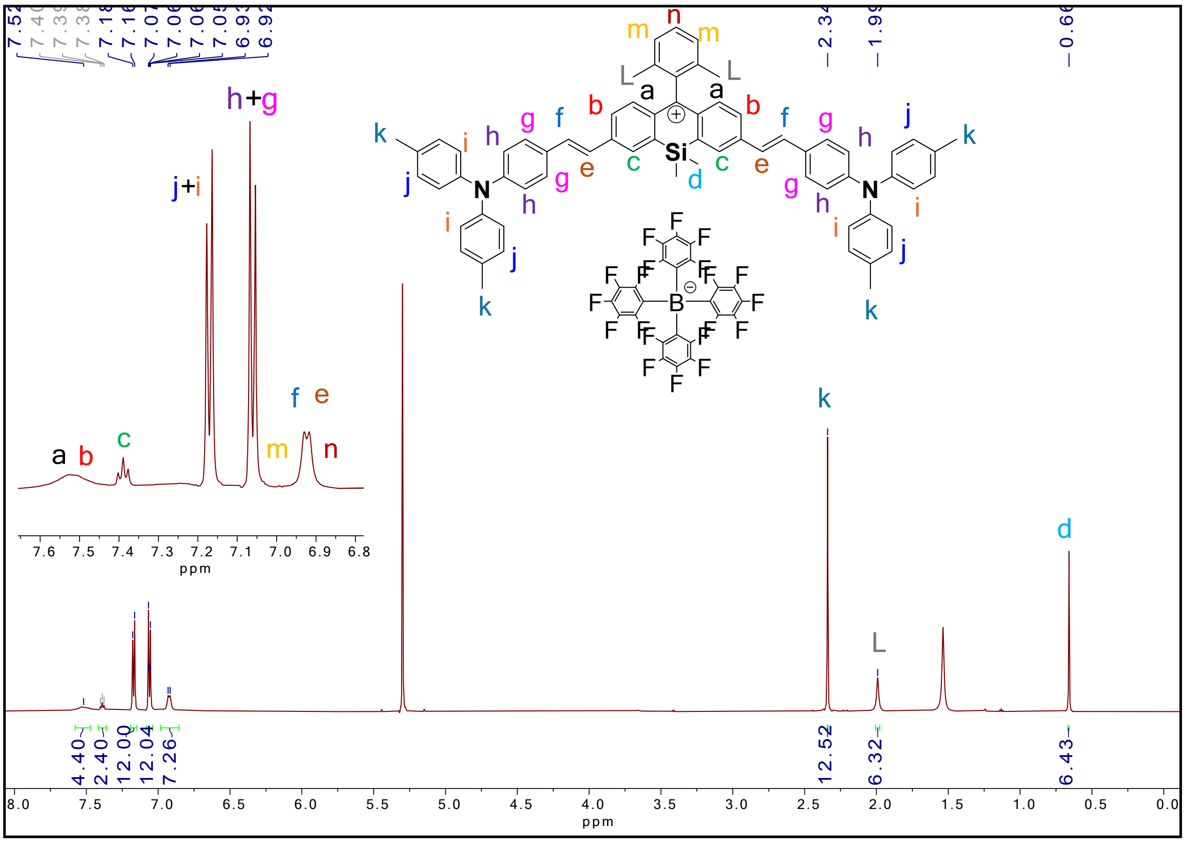


$^{13}$C NMR of **Compound T-Si-T**

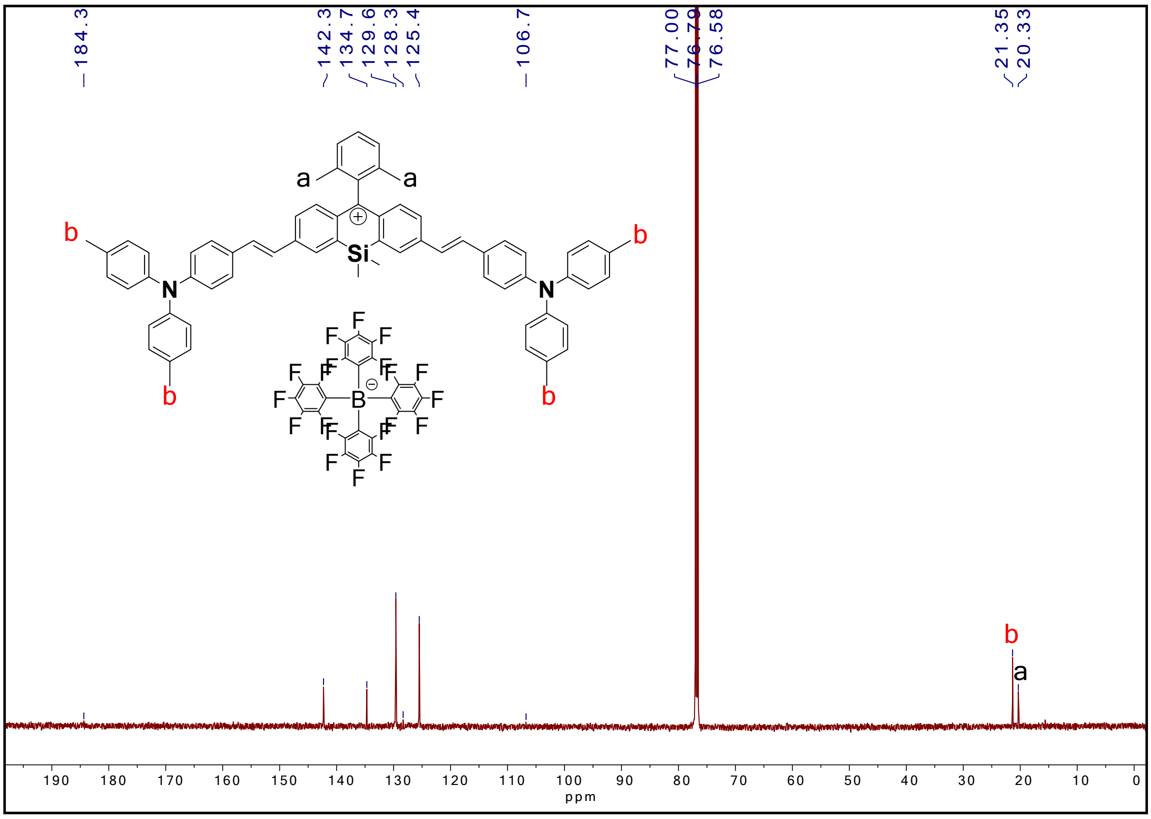


$^{19}$F NMR of **Compound T-Si-T**

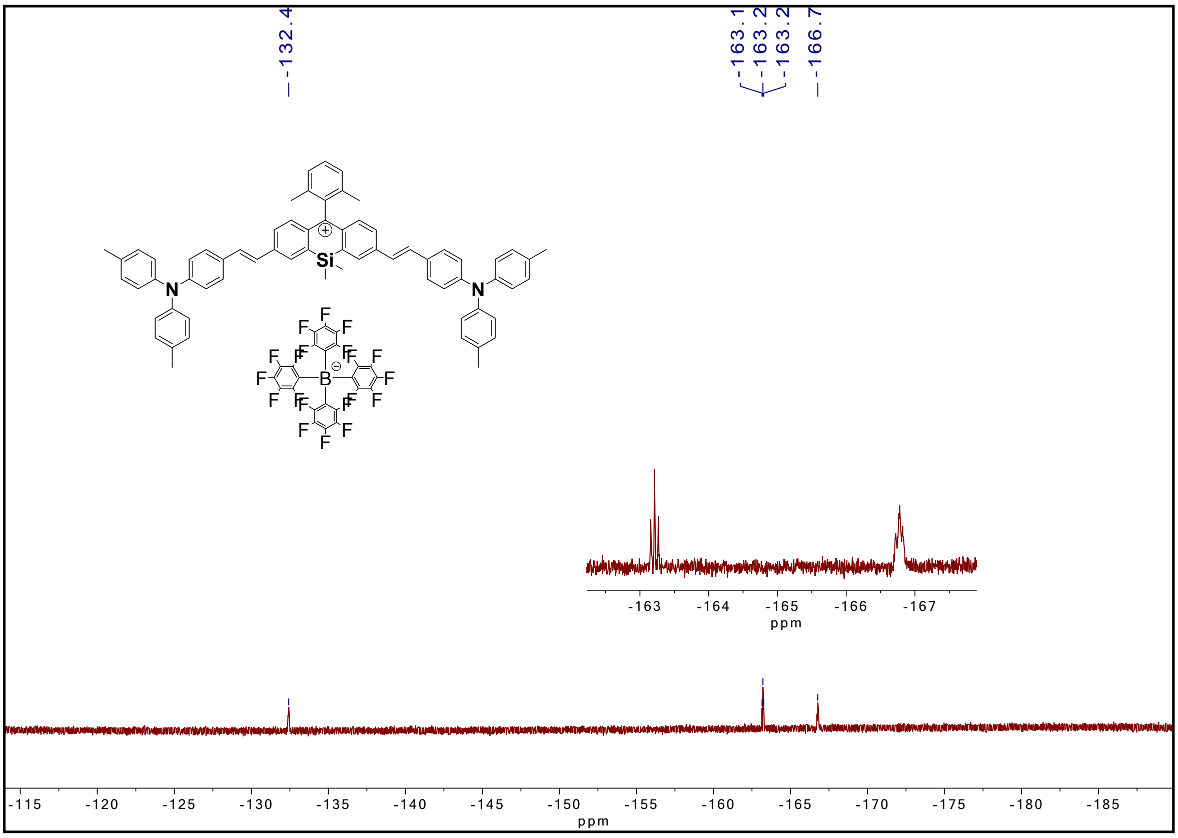


ESI-HRMS of **Compound T-Si-T**

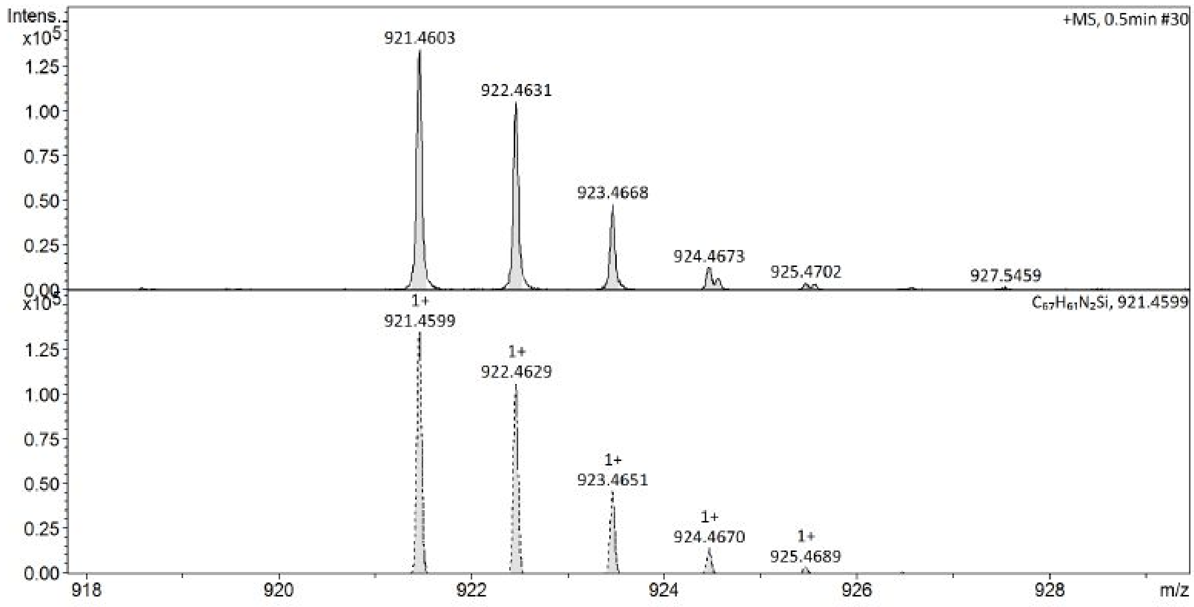


$^{1}$H NMR of **Compound BJ-Si-BJ**

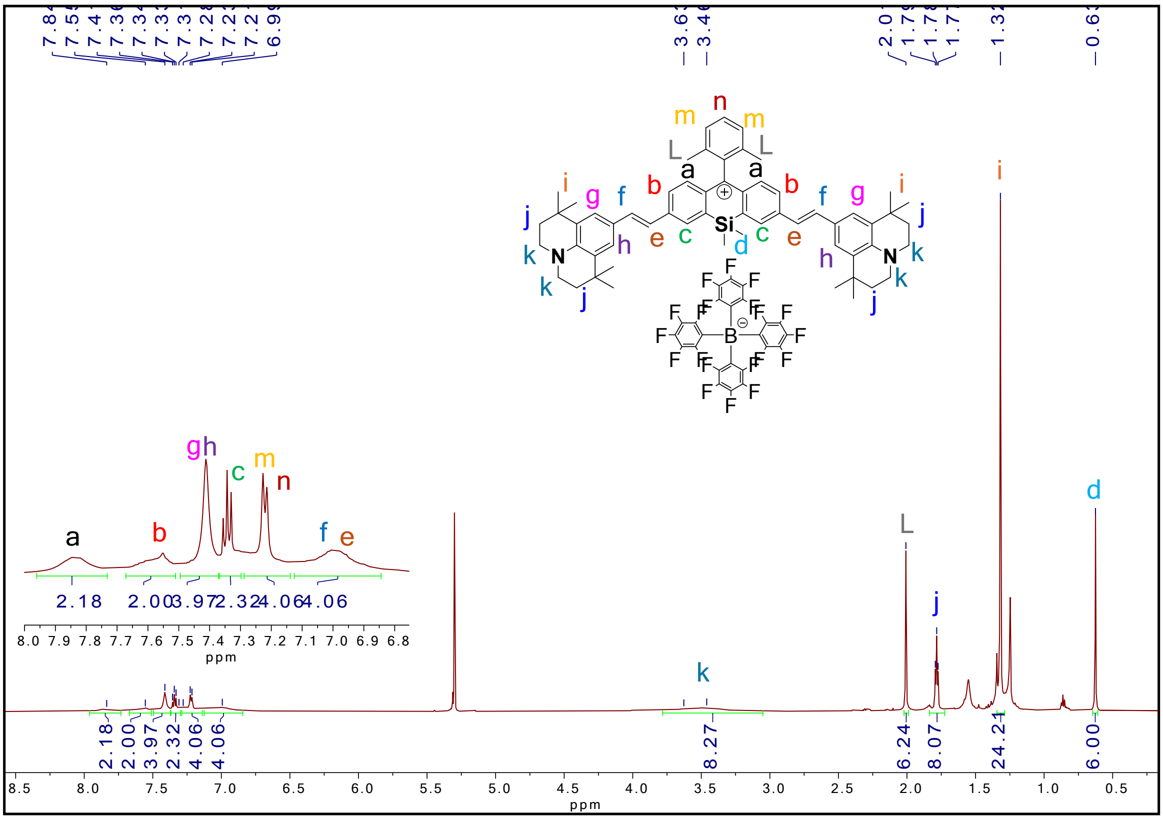


$^{13}$C NMR of **Compound BJ-Si-BJ**

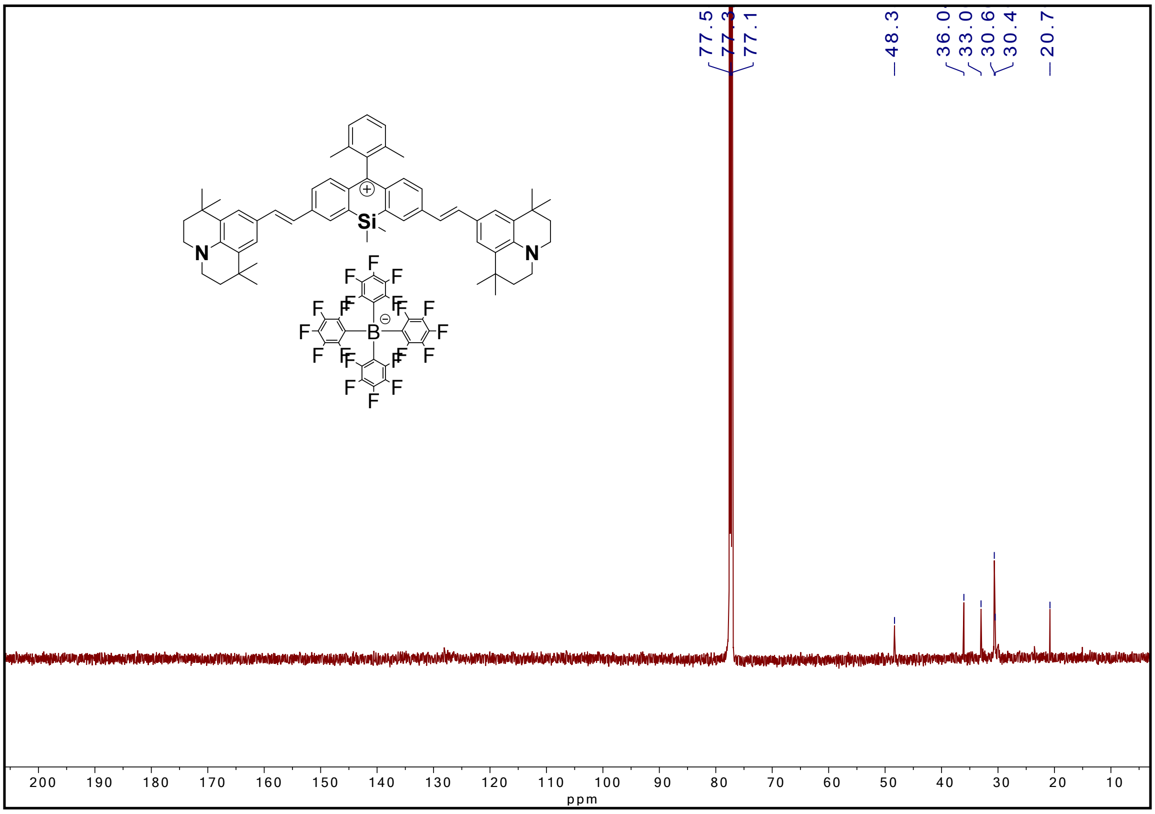


$^{19}$F NMR of **Compound BJ-Si-BJ**

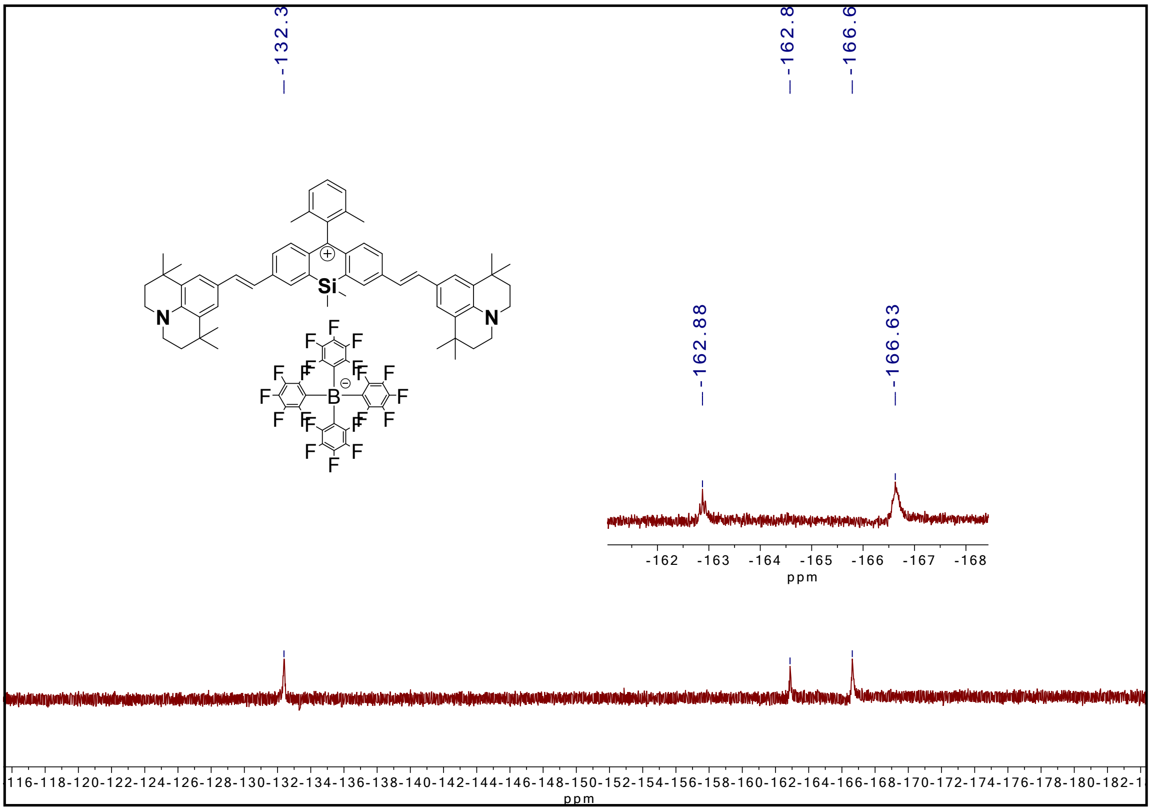


ESI-HRMS of **Compound BJ-Si-BJ**

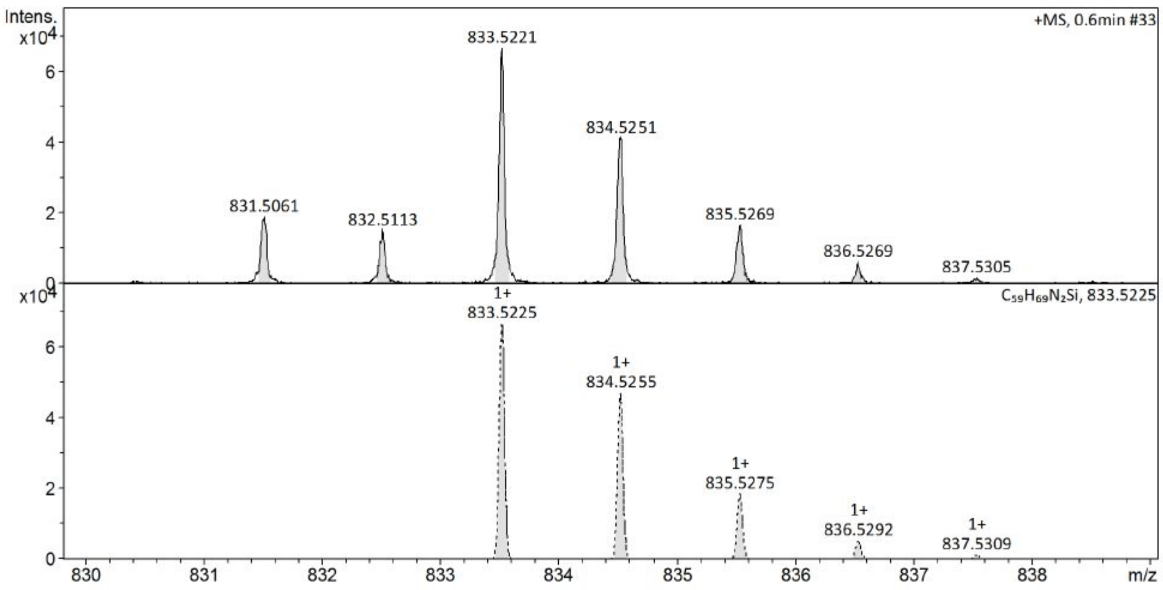


$^1$H NMR of **Compound T-Si-BJ**

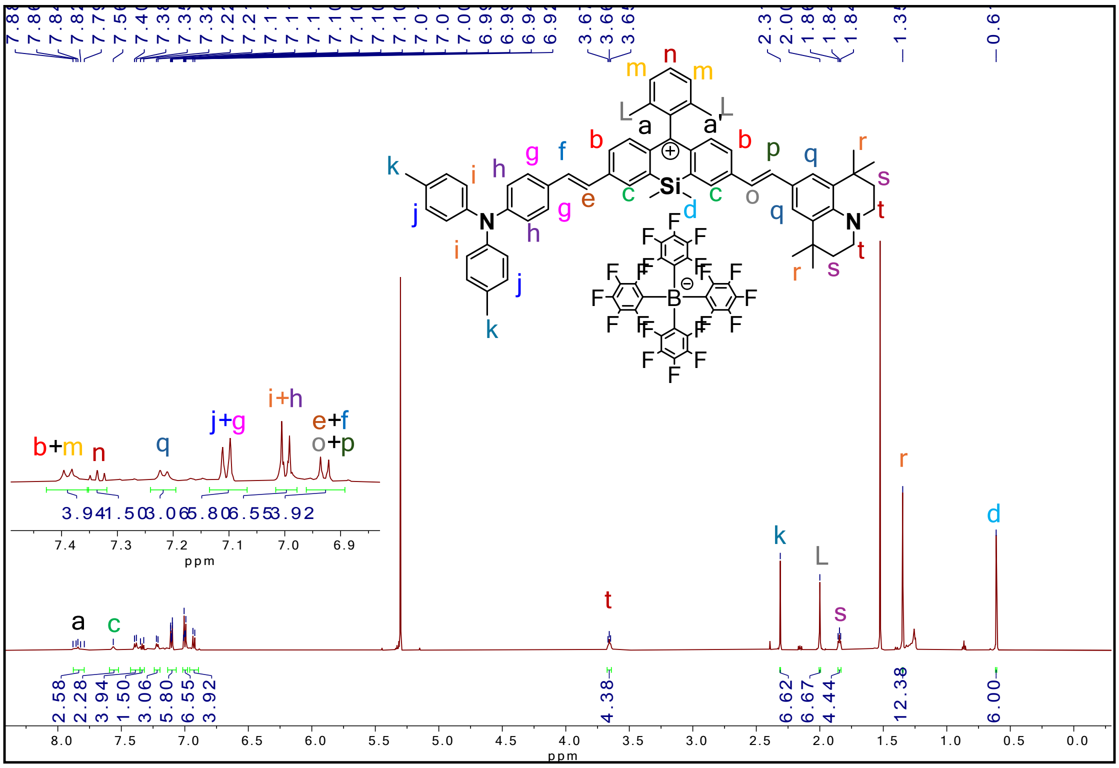


$^{13}$C NMR of **Compound T-Si-BJ**

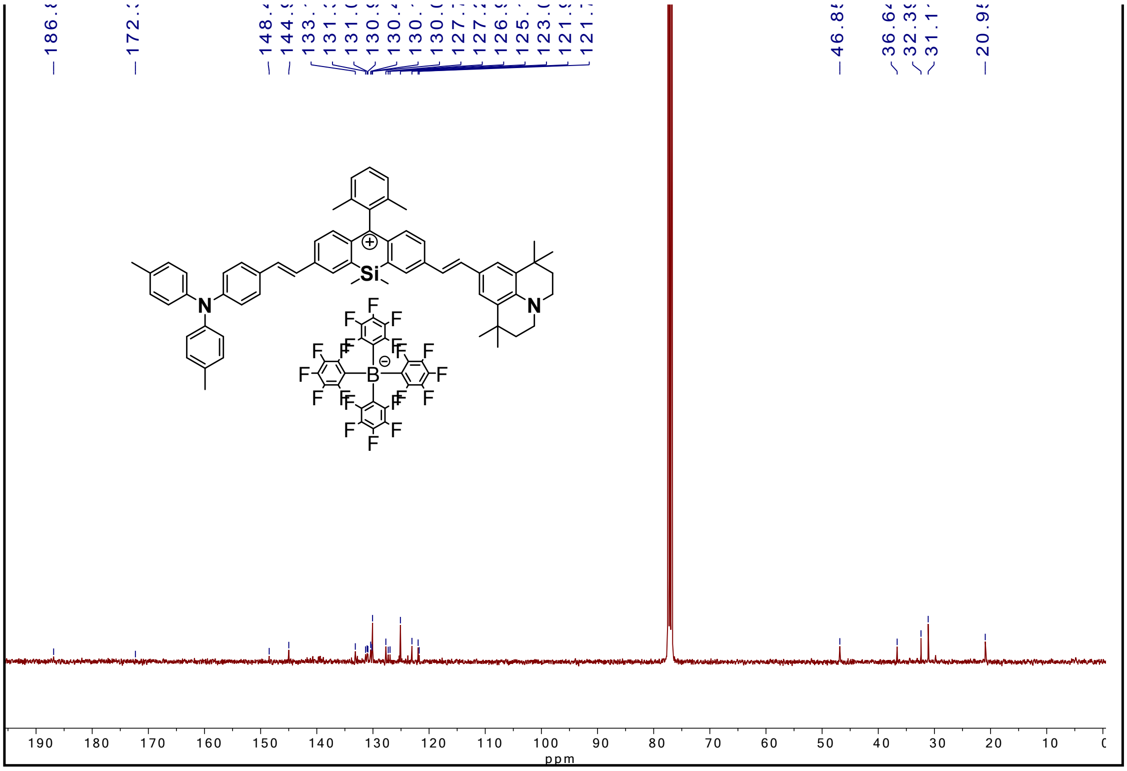


$^{19}$F NMR of **Compound T-Si-BJ**

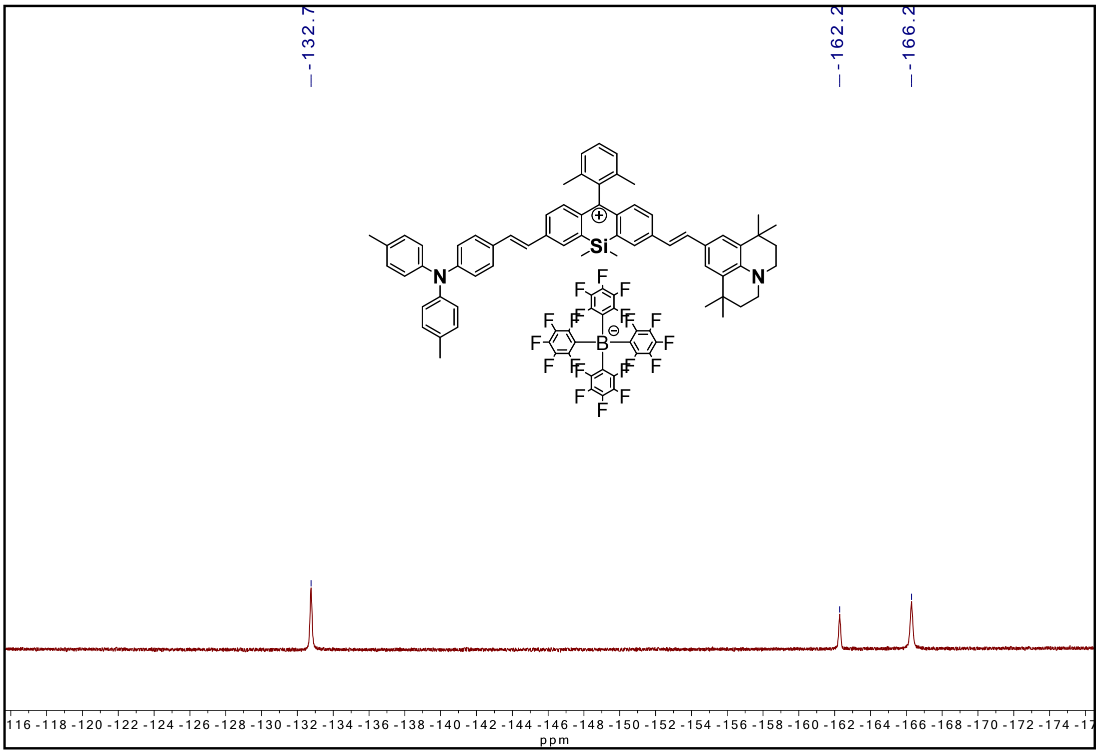


ESI-HRMS of **Compound T-Si-BJ**

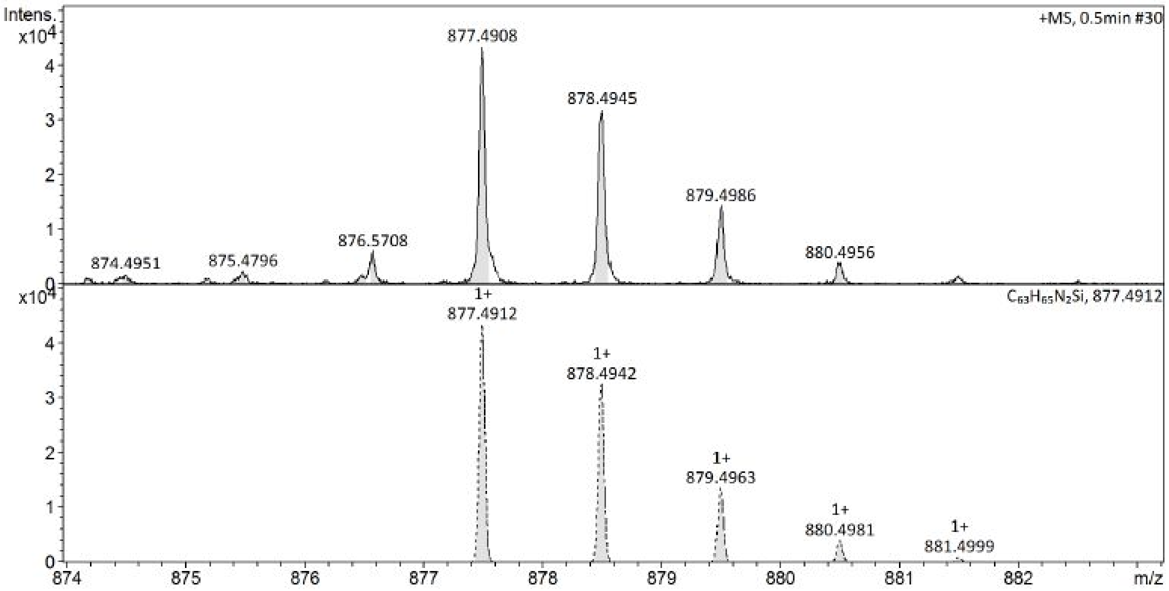


## References


(1) Chowdhury, P.; Lu, Z.-Y.; Su, S.-P.; Liu, M.-H.; Lin, C.-Y.; Wang, M.-W.; Luo, Y.-C.; Lee, Y.-J.; Chiang, H. K.; Chan, Y.-H. Ultrabright Dibenzofluoran-Based Polymer Dots with NIR-IIa Emission Maxima and Unusual Large Stokes Shifts for 3D Rotational Stereo Imaging. *Adv. Healthc. Mater.* **2024**, *13*, 2400606.

(2) Pan, H.-M.; Wu, C.-C.; Lin, C.-Y.; Hsu, C.-S.; Tsai, Y.-C.; Chowdhury, P.; Wang, C.-H.; Chang, K.-H.; Yang, C.-H.; Liu, M.-H.; et al. Rational Design of Asymmetric Polymethines to Attain NIR(II) Bioimaging at >1100 nm. *J. Am. Chem. Soc.* **2023**, *145*, 516-526.

(3) Wu, I. C.; Yu, J.; Ye, F.; Rong, Y.; Gallina, M. E.; Fujimoto, B. S.; Zhang, Y.; Chan, Y.-H.; Sun, W.; Zhou, X.-H.; et al. Squaraine-Based Polymer Dots with Narrow, Bright Near-Infrared Fluorescence for Biological Applications. *J. Am. Chem. Soc.* **2015**, *137*, 173-178.

(4) Meador, W. E.; Lin, E. Y.; Lim, I.; Friedman, H. C.; Ndaleh, D.; Shaik, A. K.; Hammer, N. I.; Yang, B.; Caram, J. R.; Sletten, E. M.; et al. Silicon-RosIndolizine Fluorophores with Shortwave Infrared Absorption and Emission Profiles Enable In Vivo Fluorescence Imaging. *Nat. Chem.* **2024**, *16*, 970-978.

(5) Meador, W. E.; Lewis, T. A.; Shaik, A. K.; Wijesinghe, K. H.; Yang, B.; Dass, A.; Hammer, N. I.; Delcamp, J. H. Molecular Engineering of Stabilized Silicon-Rosindolizine Shortwave Infrared Fluorophores. *J. Org. Chem.* **2024**, *89*, 2825-2839.

(6) Kaur, R.; Kruse, N. A.; Smith, C.; Hammer, N. I.; Delcamp, J. H. Comparison of Vinyldimethylaniline and Indolizine Donor Groups on Si-Substituted Xanthene Core Shortwave Infrared Fluorophores. *ChemPhotoChem* **2024**, *8*, e202400023.

(7) Hoshi, R.; Suzuki, K.; Hasebe, N.; Yoshihara, T.; Tobita, S. Absolute Quantum Yield Measurements of Near-Infrared Emission with Correction for Solvent Absorption. *Anal. Chem.* **2020**, *92*, 607-611.

(8) Feng, Z.; Li, Y.; Chen, S.; Li, J.; Wu, T.; Ying, Y.; Zheng, J.; Zhang, Y.; Zhang, J.; Fan, X.; et al. Engineered NIR-II Fluorophores with Ultralong-Distance Molecular Packing for High-Contrast Deep Lesion Identification. *Nat. Commun.* **2023**, *14*, 5017.
